# Discovery of an internal alkyne warhead scaffold for irreversible hTG2 inhibition

**DOI:** 10.1039/d5md00777a

**Published:** 2025-10-09

**Authors:** Lavleen K. Mader, Namita Maunick, Jessica E. Borean, Jeffrey W. Keillor

**Affiliations:** a Department of Chemistry and Biomolecular Sciences, University of Ottawa Ottawa Ontario K1N 6N5 Canada jkeillor@uottawa.ca

## Abstract

Human tissue transglutaminase (hTG2) is a multifunctional enzyme with both protein cross-linking and G-protein activity. Dysregulation of these functions has been implicated in diseases such as celiac disease and cancer, prompting the development of hTG2 inhibitors, many of which act covalently *via* a pendant electrophilic warhead. Most small molecule hTG2 inhibitors to date feature terminal, sterically minimal warheads, based on the assumption that bulkier electrophiles impair binding and reactivity. Here, we report structure–activity relationships (SAR) of a novel internal alkynyl warhead scaffold for irreversible inhibition of hTG2. This series includes one of the most potent non-peptidic hTG2 inhibitors reported to date. We demonstrate that this scaffold not only inhibits transamidase activity but also abolishes GTP binding, while exhibiting excellent isozyme selectivity. In addition, we investigate the tunability and stability of this warhead, providing insights into its broader applicability. Through detailed kinetic analysis, this study establishes a new scaffold for irreversible hTG2 inhibition and expands the design principles for covalent warheads beyond traditional terminal systems.

## Abstract 2

A unique internal alkynyl warhead scaffold irreversibly inhibits hTG2 by locking it in an open conformation, abolishing both transamidase and G-protein activities.

## Graphical abstract

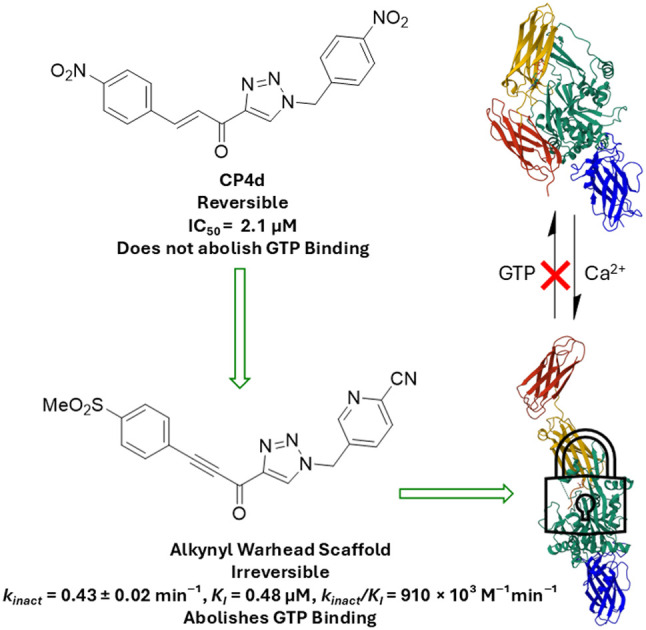

## Introduction

Human tissue transglutaminase (hTG2) is one of nine enzymes in the transglutaminase (TGase) family, characterized by their ability to catalyze protein cross-linking in the extracellular matrix *via* a transamidation reaction involving a catalytic cysteine (Cys277).^1,2^ Uniquely, hTG2 is multifunctional: in addition to transamidation, it can bind guanosine triphosphate (GTP) and act as a G-protein in intracellular signalling. These two functions are mutually exclusive; each is associated with a distinct conformational state.^1,3,4^ In the extracellular space, high calcium concentrations favour an extended “open” conformation that exposes the transamidase active site but abolishes the GTP binding pocket. Conversely, under intracellular conditions, low calcium concentrations favour a compact “closed” conformation, allowing the GTP binding site to form and occluding the transamidase site.^5–8^ Dysregulation of either function has been linked to pathologies including celiac diseases,^9^ fibrosis,^10^ and cancer,^11,12^ making hTG2 an attractive therapeutic target.

**Fig. 1:**
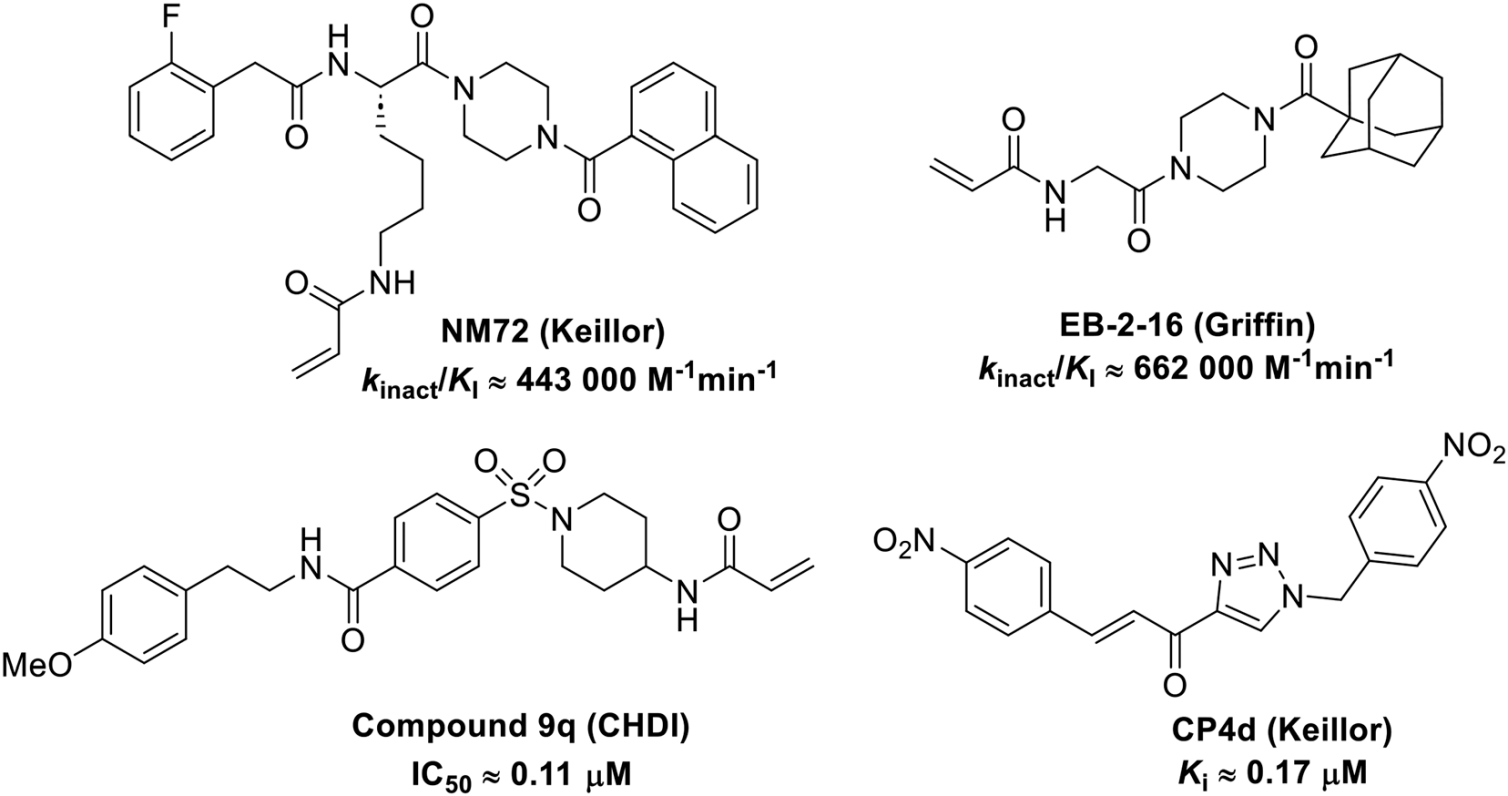
Representative small molecule hTG2 inhibitors.^15,17–19^

The conformationally-based exclusivity of the two functions enables a unique targeted covalent inhibition (TCI) strategy, whereby irreversible covalent modification of Cys277 in the transamidase site locks hTG2 in its open conformation, simultaneously blocking both of its functions.^13^ Several small molecule and peptidomimetic TCI scaffolds have been developed (Fig. 1),^14–19^ but peptidic inhibitors often suffer from poor pharmacokinetics, prompting increased interest in non-peptidic scaffolds.^14^

A key determinant of covalent inhibition is the nature of the electrophilic warhead. In a recent study, we evaluated multiple warheads on a scaffold (EB-2-16) originally reported by Badarau *et al.*^17,20^ We found that effective Cys277 engagement generally required “terminal” warheads (*i.e.*, minimally hindered electrophiles capable of accessing the active site tunnel). This is consistent with most reported irreversible hTG2 inhibitors, which typically feature a simple acrylamide warhead (Fig. 1). However, our group also previously identified CP4d,^19^ a structurally distinct inhibitor bearing a bulky, internal electrophilic warhead. Despite the presence of this warhead, CP4d acted reversibly and failed to react irreversibly with Cys277 or to lock hTG2 into the open state,^11,21^ and was not pursued further.

Here, we revisit CP4d with a library of alkynyl analogues, which interestingly, function as potent irreversible inhibitors that abolish GTP binding and exhibit high isozyme selectivity. These compounds feature an internal keto-alkyne-phenyl warhead, with tunable reactivity properties (*k*_inact_ parameter). Our findings challenge the prevailing view that hTG2 covalent inhibitors require terminal, sterically minimal warheads, and provide a novel scaffold for future structure-based inhibitor design.

## Results and discussion

### Discovery of alkynyl scaffold

CP4d represents a peculiar class of hTG2 inhibitors. It bears an obvious electrophilic group and is a competitive inhibitor with the acyl-donor substrate of our standard activity assay (as a surrogate for transamidase activity), but does not irreversibly modify Cys277 or lock hTG2 in an open conformation.^11,19,21^ Given the structure of its warhead, CP4d would be expected to react covalently, even if only through reversible covalent modification, due to the strong electron withdrawing nitrophenyl group.^22^ Reactivity studies with CP4d and glutathione in solution showed rapid and irreversible GSH adduct formation in a matter of minutes, highlighting the high intrinsic reactivity of the warhead.^23^ These findings suggest that the compound may bind in the active site but not in a conformation that allows reaction with Cys277, leading to reversible competitive binding.

Prior efforts in our group probed the reactivity of this scaffold with alkyne (KA22b) and bis-triazole derivatives.^24^ At the time it was assumed that these analogues were also reversible inhibitors, as kinetic analysis was performed using initial rate data over a very short period of time. However, an independent dialysis experiment later showed that no hTG2 activity was recovered after incubating with KA22b and dialyzing the inhibitor into buffer.^23^ Given the fact that the apparent IC_50_ and *K*_i_ values of this inhibitor were in the micromolar range, we ruled out “tight” binding as a cause of this result. This led us to our current hypothesis that the alkyne scaffold was in fact capable of irreversible inhibition and its evaluation and subsequent SAR analysis required rigorous irreversible inhibition kinetic analysis.

**Fig. 2:**
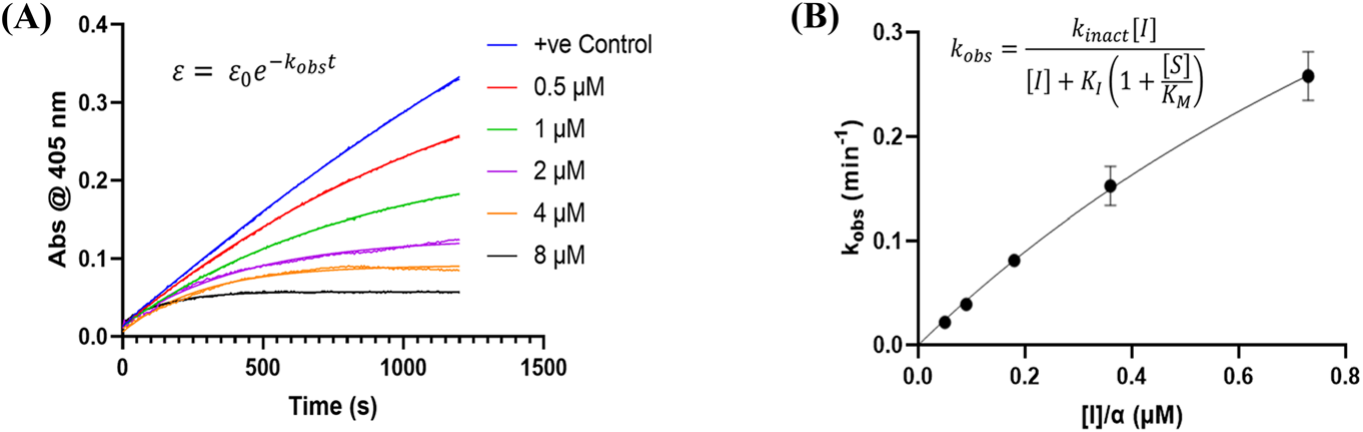
Representative hTG2 inhibition kinetic curves with inhibitor 8A. (A) Rate constants for the loss of activity (*k*_obs_) and (B) their hyperbolic fitting to saturation kinetic model (see Material and methods and SI).

We evaluated KA22b (also referred to as 8A in this work) following our standard AL5 assay protocol, carried out under Kitz and Wilson conditions, and indeed observed classic time-dependent inhibition as expected for an irreversible inhibitor.^25–27^ The observed rate constants of inhibition (*k*_obs_) acquired from fitting of curves of absorbance *vs.* time (Fig. 2A) were plotted against inhibitor concentration corrected for the presence of a competitive substrate. The resulting hyperbolic curve (Fig. 2B) was fitted to a saturation kinetics model to obtain *k*_inact_ = 0.90 ± 0.09 min^−1^ and *K*_I_ = 1.80 ± 0.24 μM with an overall inhibitor efficiency of *k*_inact_/*K*_I_ = 499 ± 0.85 × 10^3^ M^−1^ min^−1^. This represents a remarkably efficient inhibitor to identify early in a campaign, on par with the lead scaffolds shown in Fig. 1.

**Fig. 3:**
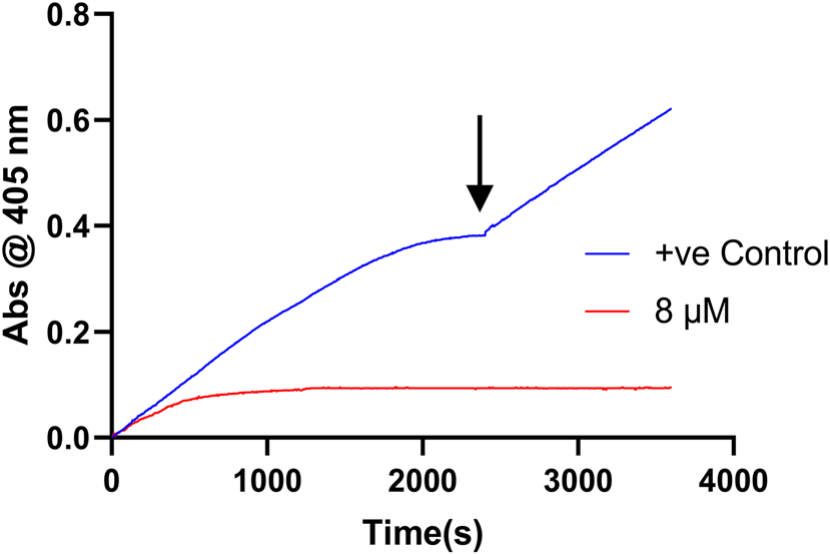
Substrate spike test showing irreversible nature of 8A inhibition.

We also demonstrated the irreversibility of inhibition by 8A by performing a substrate spike test as previously described,^25^ in which the assay is allowed to run until a plateau is reached in the uninhibited and inhibited reaction (Fig. 3). This plateau results from substrate depletion in the former, but complete enzyme inactivation in the latter. More substrate is then added (resulting in a concentration that is equivalent to its initial concentration in the assay), effectively diluting the reactions with substrate solution (Fig. 3, arrow). The activity of both reactions is then monitored again. The inhibition is confirmed to be irreversible as no activity is regained, whereas the activity of the uninhibited control returns to its initial activity, as observed in the first phase of the assay. The uninhibited control is crucial here to ensure that lack of activity is not due to enzyme degradation. Further validation of the irreversibility of this scaffold is provided by its ability to completely abolish GTP binding, unlike CP4d (see *Inhibition of GTP binding* below).

### Design and synthesis

Confirmation of 8A as a potent irreversible hTG2 inhibitor prompted us to design and synthesize various derivatives to give better understanding of the pharmacophore of this scaffold and a potential binding mode, as well as reactivity of the warhead. 8A, while potent, poses several challenges. It is a very symmetrical, flat molecule which imparts poor solubility, and it also contains nitro groups which have mutagenic/toxicity potential.^28^ Moreover, the activated keto-alkyne warhead is very intrinsically reactive and has poor stability in GSH reactivity assays (see *Intrinsic reactivity* below).^23^ Therefore, prominent goals were to increase solubility and replace the aryl nitro groups, while tuning the warhead for stability.

**Scheme 1:**
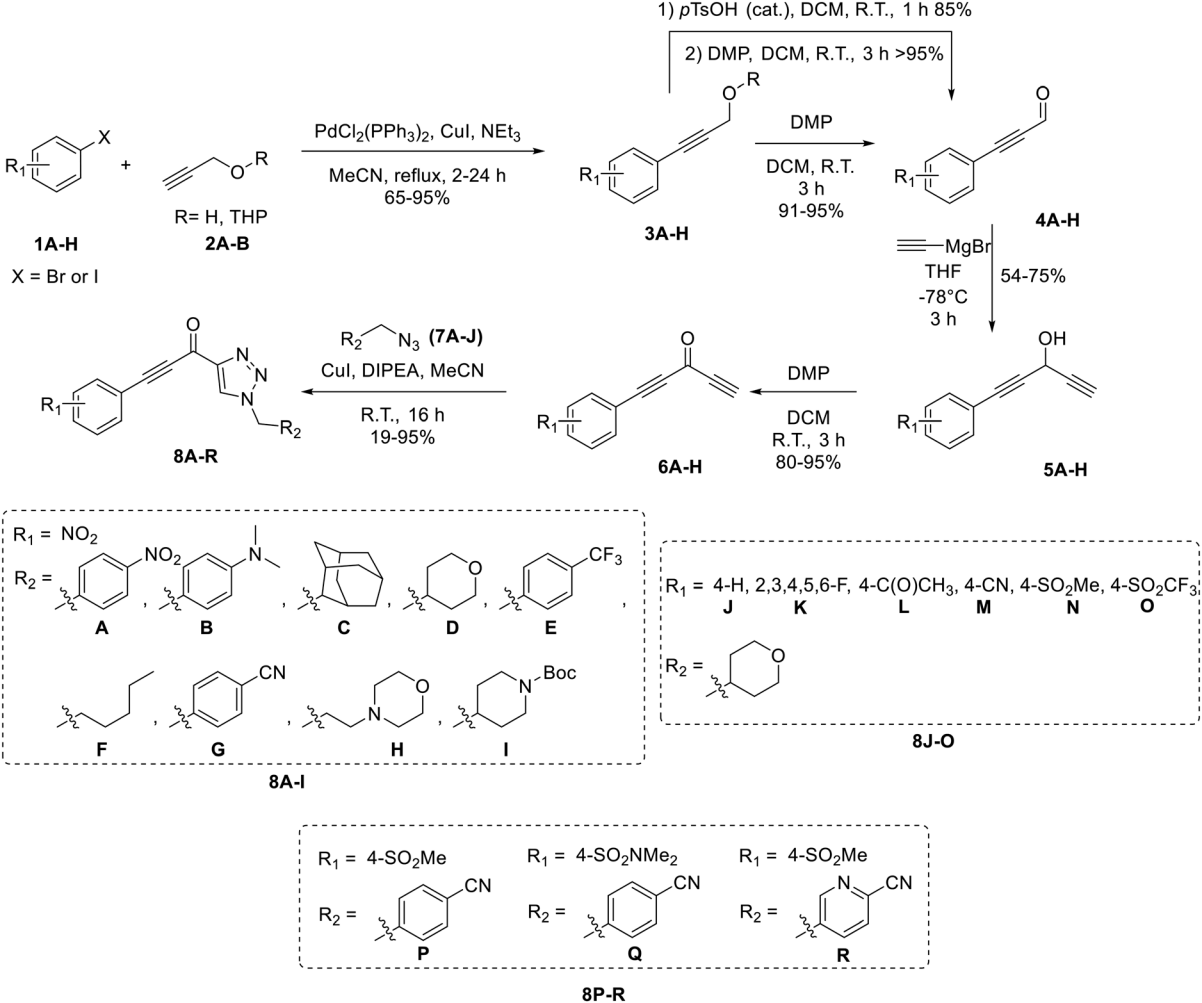
Synthesis of inhibitors 8A–R.

**Scheme 2:**
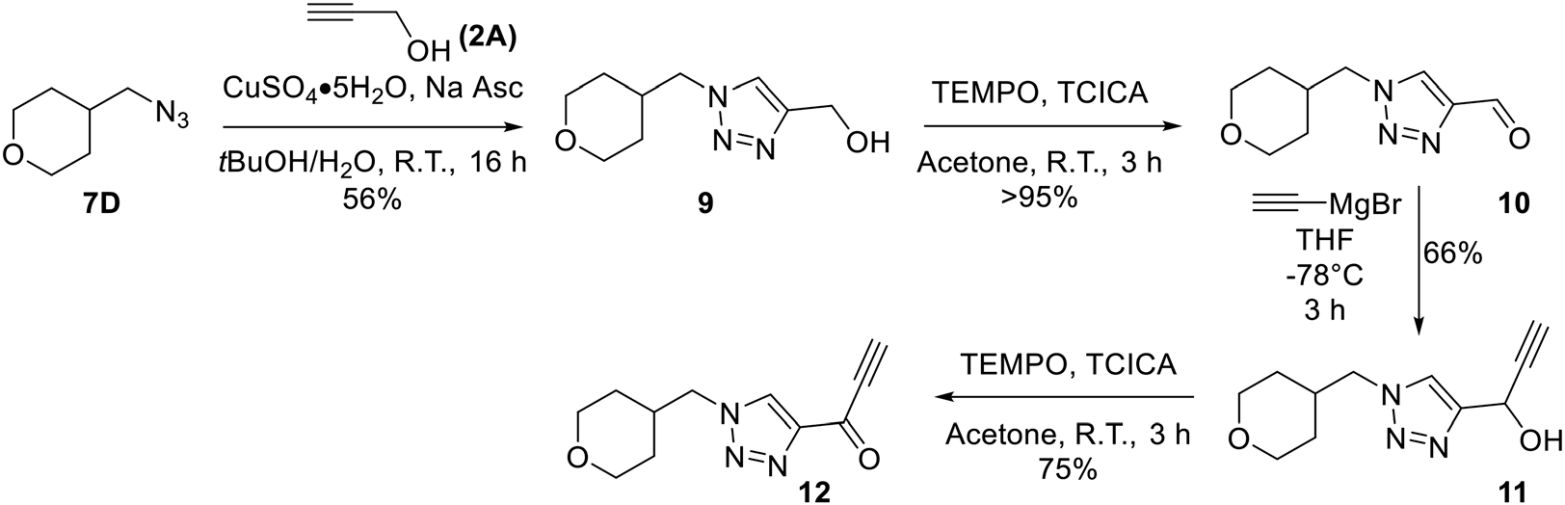
Synthesis of inhibitor 12.

The synthesis of most analogues follows our previously reported procedure (Scheme 1).^23,24^ Aryl iodides 1A–H were either commercially available or synthesized according to known procedures (see *Materials and methods*). THP-protected propargyl alcohol (2B) was synthesized as previously described and azides 7A–J were synthesized by standard literature procedures. In the case of the pentafluorobenzene analogue (8K), additional protection and deprotection steps were required as side products from an S_N_Ar reaction were observed when the free alcohol was used in the first Sonogashira reaction. Compound 12, bearing only a hydrogen at the terminal end of the alkyne, was also synthesized by a slightly modified route (Scheme 2), since working with a terminally unsubstituted alkyne (*i.e.*, without a phenyl ring) would likely lead to highly reactive and volatile intermediates, making the original route challenging. Here, TEMPO/TCICA was used as the oxidant, since DMP resulted in poor yields.

In the first series of compounds (8A–I), we altered the 1-position of the 1,4-substituted triazole with a variety of alkyl, cyclic/heterocyclic, and substituted phenyl moieties. When a group that replaced the nitro group while retaining potency and improving solubility was identified (see *Kinetic evaluation* below), that substituent was retained in the second series of compounds (8J–O, 12) to allow kinetic analysis without solubility as a barrier. 8J–O, and 12 probe the stereo electronic effects on the terminal phenyl ring attached to the alkyne warhead. Based on the results from the first two series (see *Kinetic evaluation* below), the elements that increased potency, solubility, and replaced nitro groups were combined to form compounds 8P–R.

**Scheme 3:**
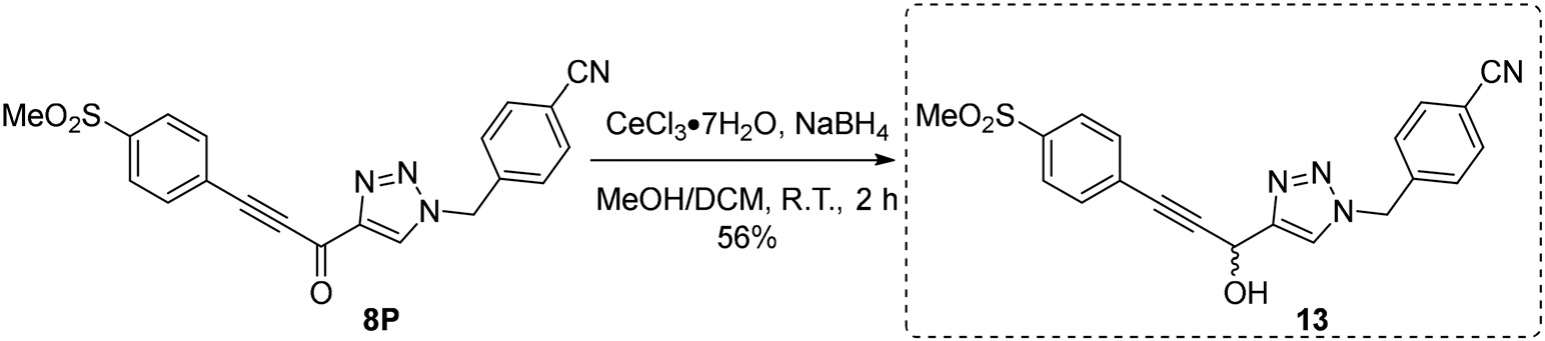
Synthesis of inhibitor 13.

Next, we made a series of derivatives (13, 18, 23A–E) to probe and attempt to improve the stability (*i.e.*, lower intrinsic reactivity) of the alkynyl warhead while retaining sufficient reactivity and binding affinity to inhibit hTG2. Compound 13, synthesized *via* a Luche reduction (Scheme 3), lowers the electrophilicity of the warhead by removing electron withdrawing affects from the adjacent carbonyl.

**Scheme 4:**
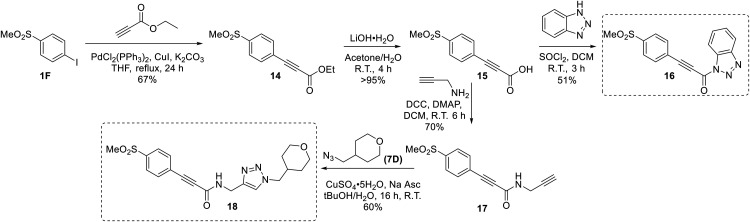
Synthesis of inhibitors 16 and 18.

**Scheme 5:**
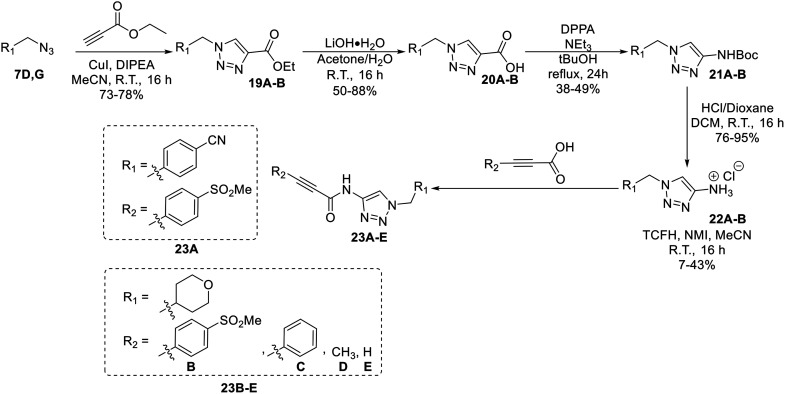
Synthesis of compounds 23A–E.

We also synthesized propiolamide derivatives 16, 18, 23A–E (Schemes 4 and 5), as the electron donation of the amine to the carbonyl is known to attenuate the intrinsic reactivity of α,β-unsaturated carbonyls.^29^ Some additional modifications were also made to the terminal substituent of the alkyne within the propiolamide series, to probe the effects of sterics and electronics, at the end of the alkyne, on intrinsic reactivity (see *Intrinsic reactivity* below).

Compound 16 was synthesized in similar fashion to a previously described protocol,^24^ and compound 18 was obtained by an amide coupling of intermediate 15 with propargyl amine, followed by a copper-catalysed click reaction.

Synthesis of compounds 23A–E features a click reaction with ethyl propiolate and subsequent hydrolysis to produce intermediate 20. This intermediate was then subjected to a Curtius rearrangement and deprotection to install a free amine at the 4-position of the triazole (22). This intermediate was then coupled with various synthesized or commercially available propiolic acids using TCFH/NMI, which is known to facilitate challenging amide couplings with electron deficient amines.^30^

**Scheme 6:**
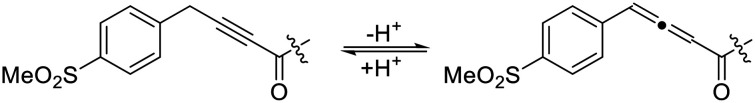
Rearrangement from alkyne to allene.

Given the stability that a terminal methyl group is known to provide to alkynone warheads (see *Intrinsic reactivity*), attempts were made to synthesize an analogue with a methylene unit between the alkynone and the phenyl group, to disrupt any conjugation that may contribute to increased electrophilicity of the warhead. However, attempts to synthesize this type of compound yielded rearrangement to an allene in mildly basic aqueous conditions (Scheme 6, data not shown), so this was not pursued further.

**Fig. 4:**
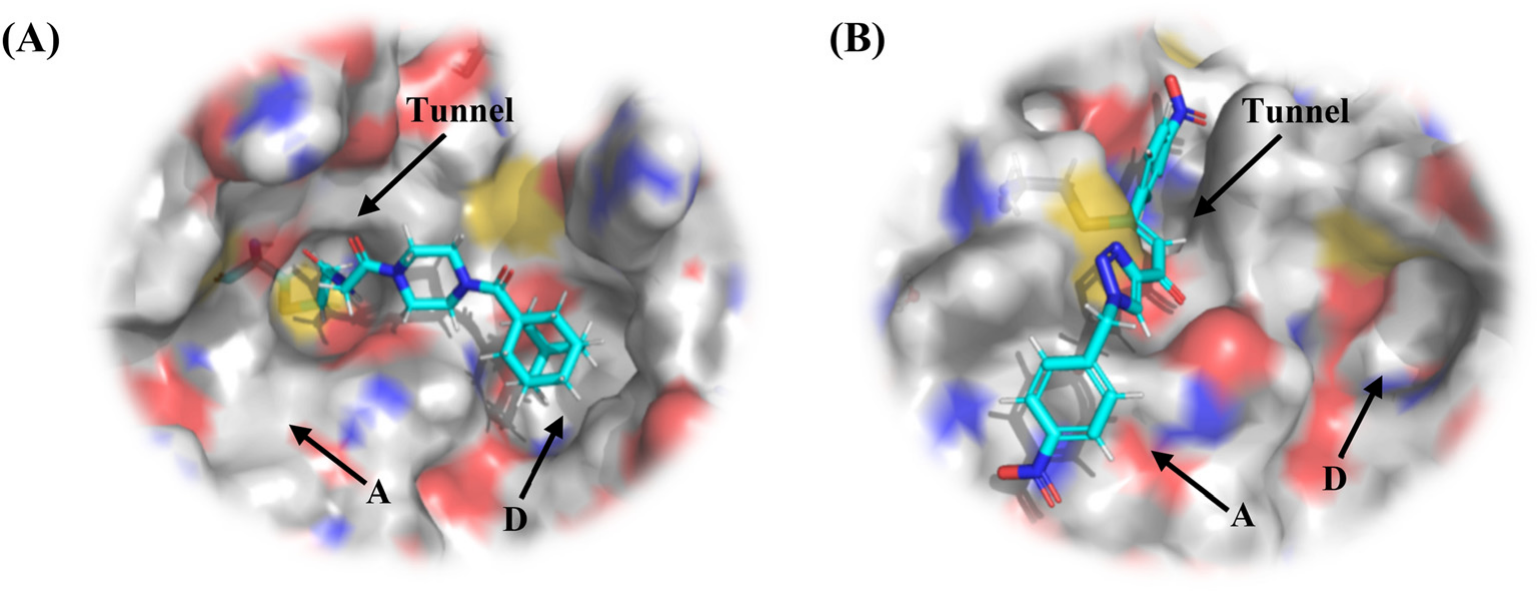
Molecular modelling of (A) EB-2-16 in crystal structure PDB: 2Q3Z and (B) 8A in crystal structure PDB: 3S3S. Tunnel, D-site, and A-site are shown by arrows (see Materials and methods).

We also found it peculiar that hTG2 could not tolerate steric bulk around the warhead of a scaffold like EB-2-16, but apparently could do so for 8A, calling into question whether different small molecule scaffolds bind at all similarly to hTG2. No crystal structures are known of hTG2 in complex with a non-peptidic small molecule inhibitor; however, a structure obtained with Ac-P(DON)LPF-NH_2_ (PDB: 2Q3Z) is widely used for structure-based design,^6^ which features three distinct pockets: an A-site, the active site tunnel, and a hydrophobic D-site. This crystallographic structure features a distinct active site ‘tunnel’, in which it appears the warhead must fit to access Cys277. This was used to rationalize the need for ‘terminal’ warheads in EB-2-16.^17^ However, in another structure of hTG2 (PDB: 3S3S), obtained with an analogue of **ZED013**,^31^ which does feature a small but internal warhead, the upper wall of this tunnel is absent in the crystal structure, suggesting that it is dynamic in nature. Perhaps surpassing a certain threshold of steric bulk forces the tunnel open, to accommodate a larger warhead. Interestingly, molecular docking of these inhibitors in the 2Q3Z*vs.*3S3S structures produced significantly different poses. While EB-2-16 could be modelled in 2Q3Z (Fig. 4A), 8A could not, as none of the resulting poses placed the warhead in the tunnel near Cys277 (data not shown). Docking of 8A in 3S3S, however, resulted in a plausible binding mode shown in Fig. 4B. Given its more rigid and linear structure, it is unlikely that 8A could both occupy the D-site and wrap around the adjacent groove to access Cys277. However, binding in the A-site would allow the warhead direct access to Cys277 without compromising its extended conformation.

To investigate these differences experimentally, we designed a series of ‘hybrid’ compounds (28, 33, 36, 38, 40) that combined elements of EB-2-16 with elements of 8A. If these two inhibitors bind similarly to TG2, some degree of cooperativity between their structural features would be expected, whereas the absence of cooperativity would suggest they adopt different binding poses.

**Scheme 7:**
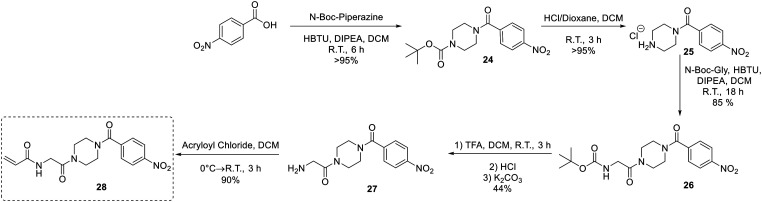
Synthesis of inhibitor 28.

An analogue of EB-2-16 bearing a *para*-nitrophenyl group in the place of the adamantane group (28) was synthesized using our previously established protocol (Scheme 7),^20^ beginning with *para*-nitrobenzoic acid.

**Scheme 8:**
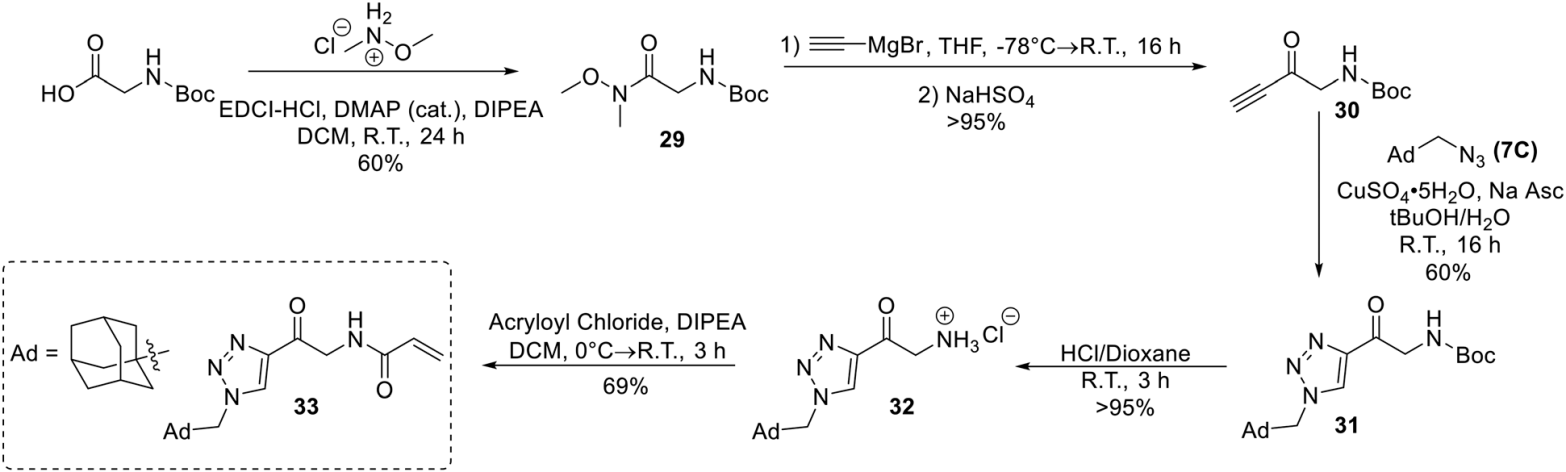
Synthesis of inhibitor 33.

Next, an analogue replacing the piperazine group of EB-2-16 with a triazole was synthesized (33). This was achieved by accessing an alkynone derivative of Boc-glycine (30) *via* a Weinreb amide and subsequent Grignard reaction with ethynylmagnesium bromide. 30 was then clicked to 1-(azidomethyl)adamantane (7C), followed by deprotection of the amine and coupling to acryloyl chloride to obtain inhibitor 33 (Scheme 8).

**Scheme 9:**
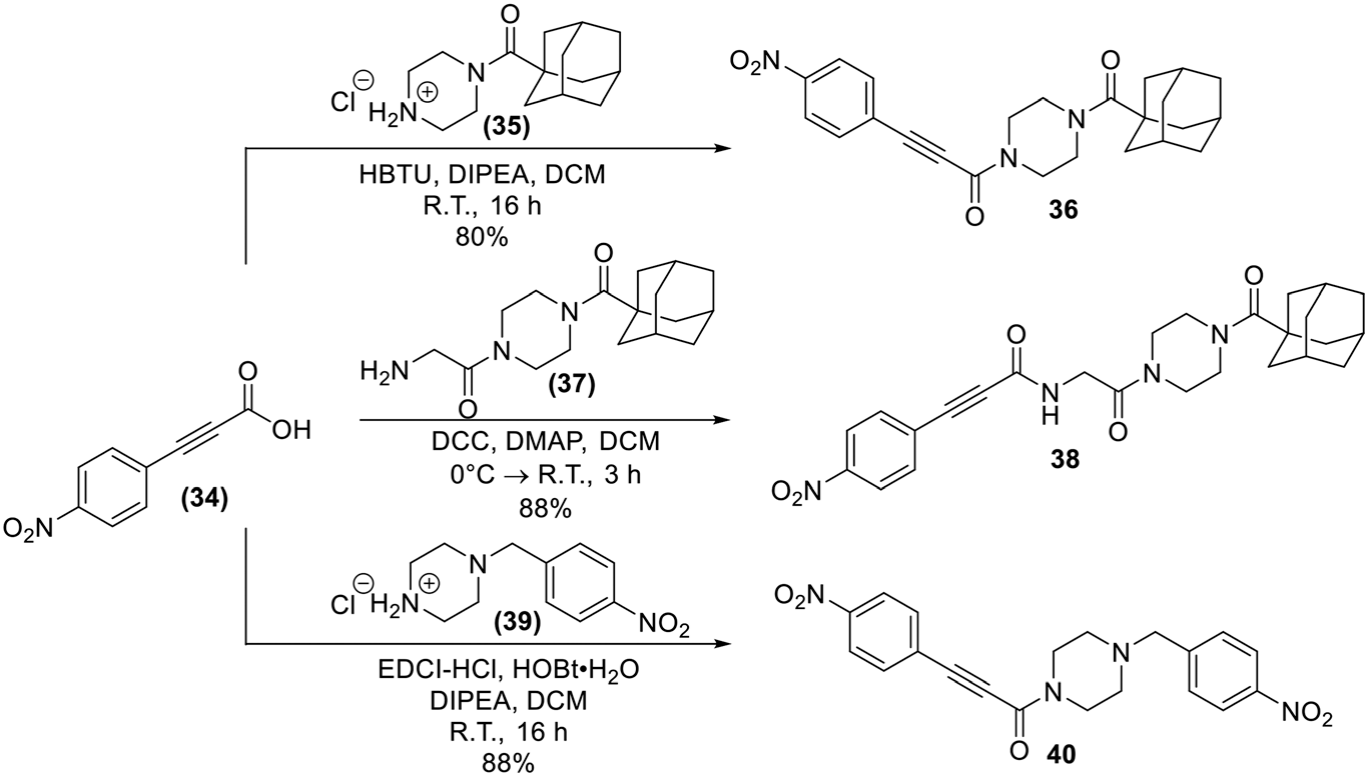
Synthesis of inhibitors 36, 38, and 40.

Lastly, analogues that combined the warhead of 8A with the scaffold of EB-2-16, with and without the glycine spacer were synthesized (36 and 38), as absence of the linker more closely resembles the size of 8A, but including the glycine linker is more consistent with the original EB-2-16 scaffold. We also included a compound with a *para*-nitrobenzyl replacing the adamantane carbonyl moiety (40), to resemble the rotatable bond and overall compound symmetry found in 8A. These compounds were obtained through amide couplings of intermediates we have previously synthesized (Scheme 9).^20^

### Structure–activity relationships

All compounds were tested as described above for 8A. Whenever possible, individual *k*_inact_ and *K*_I_ parameters were determined using nonlinear regression. However, in cases where inhibitors showed poor solubility and/or poor inhibition, such that they could not be tested at concentrations high enough to produce a saturation plot, double reciprocal fitting was used to estimate these parameters. However, it should be noted that this double reciprocal approach often produces high errors in the fitting of individual rate and inhibition constants.^25^ In these cases, the ratio of *k*_inact_/*K*_I_ is taken to be the more reliable measure of potency. All results were interpreted relative to benchmark values for 8A (*k*_inact_ = 0.90 ± 0.09 min^−1^, *K*_I_ = 1.80 ± 0.24 μM, *k*_inact_/*K*_I_ = 499 ± 0.85 × 10^3^ M^−1^ min^−1^) and EB-2-16 (*k*_inact_ = 2.64 ± 0.39 min^−1^, *K*_I_ = 3.98 ± 0.79 μM, *k*_inact_/*K*_I_ = 662 ± 164 × 10^3^ M^−1^ min^−1^).^20^

### 1-substituted triazole variants

We first started by altering the substituent at the 1-position of the triazole ring, with the goal of replacing the nitro group on the phenyl rings and increasing solubility, while retaining potency. We kept any substituents on the phenyl ring in the *para* position, as it was previously observed in CP4d and 8A derivatives that *ortho* and *meta* substituents led to a decrease in potency.^19,23^

In general, we found that a variety of groups ranging from simple alkyl chains to more complex aromatic and aliphatic heterocycles could be tolerated at this position, while maintaining overall inhibitor efficiencies of *k*_inact_/*K*_I_ ∼ 300–500 × 10^3^ M^−1^ min^−1^ (Table 1). *K*_I_ values in this series of compounds were around 1–2 μM and *k*_inact_ ranged from about 0.5–1 min^−1^, diverging very little from parent compound 8A. However, it should be noted that moieties with a hydrogen bond acceptor showed slightly increased inhibition efficiency.

**Table 1:** Kinetic parameters of triazole variants

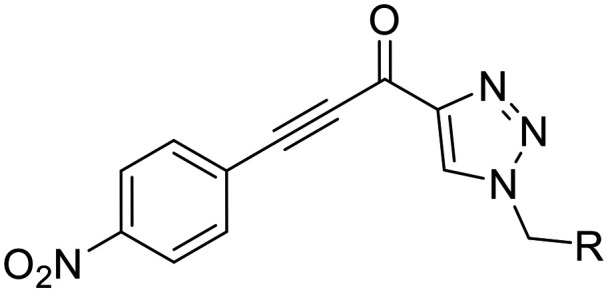				
Inh.	R	*k*_inact_ (min^−1^)	*K*_I_ (μM)	*k*_inact_/*K*_I_ (×10^3^ M^−1^ min^−1^)
8A	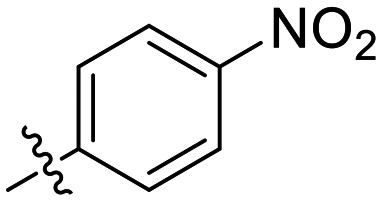	0.90 ± 0.09	1.80 ± 0.24	499 ± 85
8B^a^	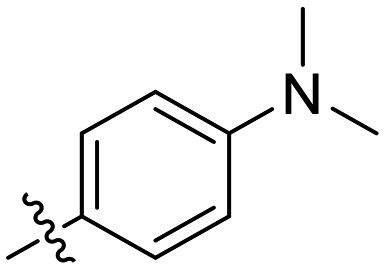	0.54 ± 0.38	1.03 ± 0.74	536 ± 27
8C^b^	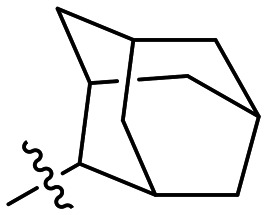	n.d.	n.d.	n.d.
8D	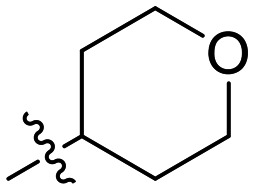	1.24 ± 0.19	2.30 ± 0.56	538 ± 156
8E^a^	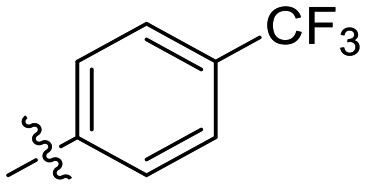	0.62 ± 0.29	1.75 ± 0.39	353 ± 136
8F^a^	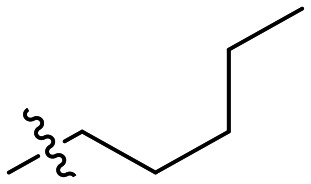	0.89 ± 0.12	2.43 ± 0.33	368 ± 3
8G	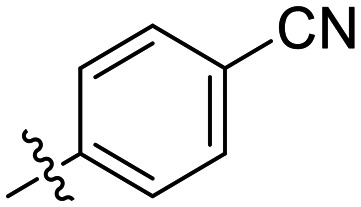	0.77 ± 0.07	0.96 ± 0.13	805 ± 128
8H	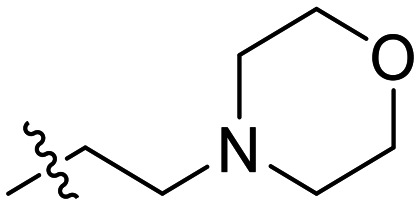	1.21 ± 0.14	2.84 ± 0.46	425 ± 84
8I^a^	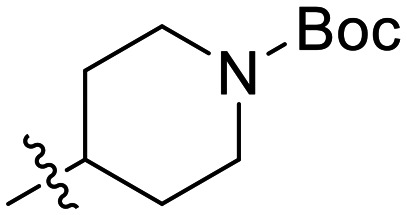	0.20 ± 0.02	0.40 ± 0.03	511 ± 15

a Determined by double reciprocal fitting.

b No inhibition detected up to solubility limit of compound (1 μM).

The most efficient compounds from this series were the tetrahydropyranyl derivative (8D) and the *para*-cyanobenzyl derivative (8G). The latter showed roughly 2-fold increase in potency compared to parent compound 8A, owing largely to an increase in binding affinity (decrease in *K*_I_), suggesting that the presence of a cyano group as a hydrogen bond acceptor in this position was superior to a nitro (8A), amine (8B), ether (8D/8H), or ester (8I). The least efficient compound in this series was the *para*-trifluoromethyl derivative (8E). Fluorine is known to have variable effects on binding affinity with hTG2 depending on the scaffold. In the case of NM72 (Fig. 1) it provided a notable increase in binding affinity for this peptidomimetic scaffold.^15^ However, in the EB-2-16 scaffold (Fig. 1), the addition of a fluorine, particularly near the warhead, resulted in a significant loss of binding affinity.^20^ Here, it seems to not drastically reduce affinity but is not particularly desirable to include either. Note that 8C is not necessarily a poor inhibitor, but it displayed very poor solubility and could not be sufficiently tested. However, it was synthesized to serve as an interesting comparison to the ‘hybrid’ compound series discussed below.

Although 8G displayed the highest overall efficiency, for the majority of the subsequent series of compounds we chose to move forward with a tetrahydropyranyl group (8D) at the 4-position as it provided higher solubility and allowed us to test compounds at higher concentrations.

### Warhead variants

Next, we probed the effects of modifications to the terminal alkynyl substituent of the warhead on reactivity and binding affinity with hTG2 (Table 2). Surprisingly, completely removing the phenyl ring (12) or removing just its *para* substituent (8J) resulted in equally poor binding affinity. While both modifications also reduced reactivity *k*_inact_, retaining the phenyl ring resulted in 3-fold higher reactivity than having no phenyl ring at all. This suggests that the phenyl ring is not providing potency by way of binding affinity, but its conjugation to the alkyne is increasing the reactivity of the warhead. This is corroborated by the intrinsic reactivity of the warheads with glutathione (see *Intrinsic reactivity*).

**Table 2:** Kinetic parameters of warhead variants

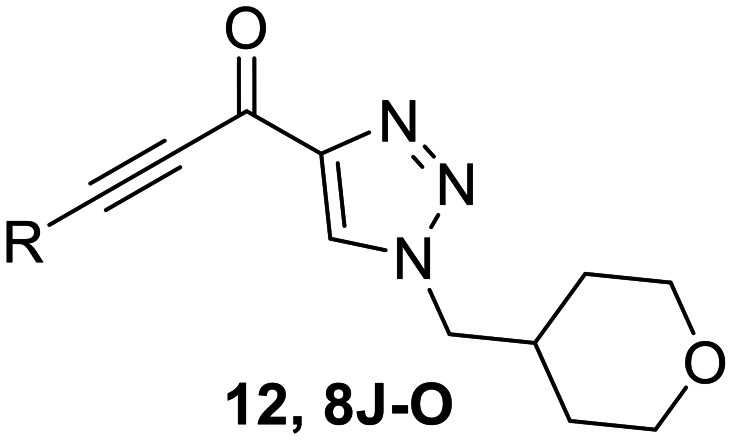				
Inh.	R/Structure	*k*_inact_ (min^−1^)	*K*_I_ or *K*_i_ (μM)	*k*_inact_/*K*_I_ (×10^3^ M^−1^ min^−1^)
12	H	0.12 ± 0.05	19.4 ± 9.1	6.30 ± 1.00
8J	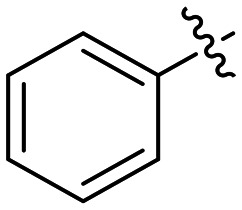	0.36 ± 0.02	23.9 ± 4.1	15.0 ± 3.0
8K	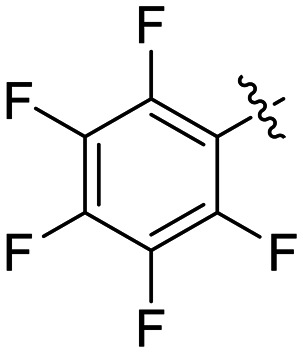	0.25 ± 0.02	7.30 ± 0.80	33.8 ± 4.3
8L	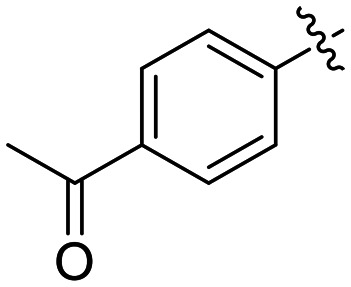	0.36 ± 0.05	1.36 ± 0.36	267 ± 81
8M	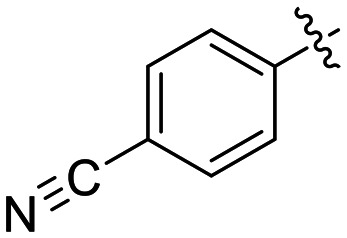	1.04 ± 0.18	2.99 ± 0.77	349 ± 109
8N	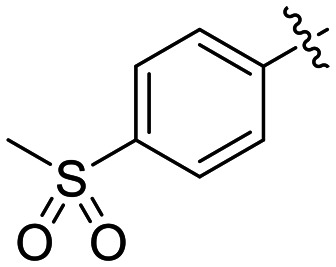	0.98 ± 0.14	2.05 ± 0.48	479 ± 131
8O	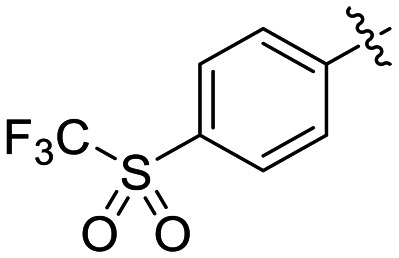	0.90 ± 0.13	3.19 ± 0.68	282 ± 74
13^a^	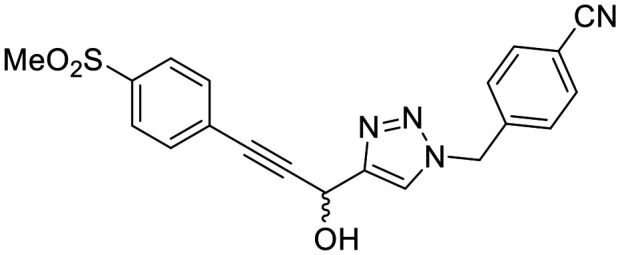	0.06 ± 0.01	18.9 ± 2.8	3.33 ± 0.54
16^b^	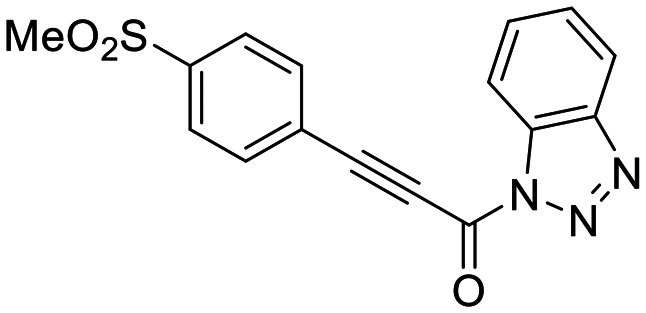	—	0.19 ± 0.02	—
18^c^	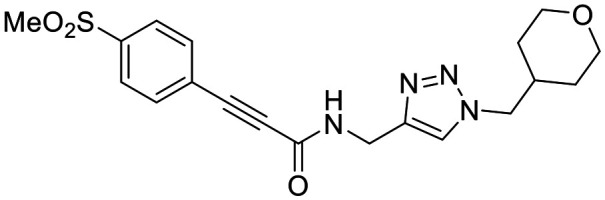	n.d.	n.d.	n.d.
23A^a^	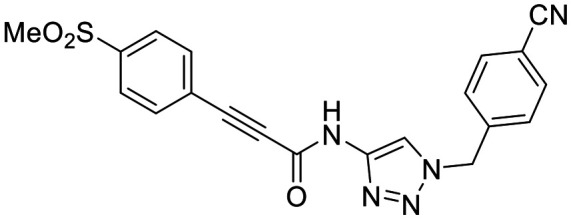	0.59 ± 0.13	11.4 ± 2.9	52.0 ± 2.0
23B	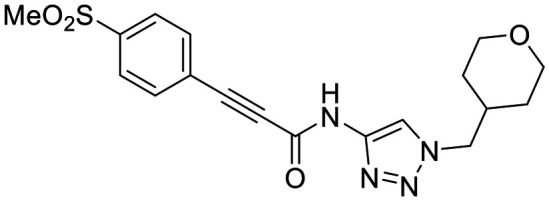	0.48 ± 0.06	21.5 ± 4.3	22.0 ± 4.0

a Determined by double reciprocal fitting.

b Reversible inhibitor.

c No inhibition detected up to solubility limit of compound (100 μM).

Preliminary results previously showed that electron donating groups on this portion of the scaffold resulted in poor potency.^23^ Thus, we opted to keep electron withdrawing groups on the phenyl ring and investigate how the strength of the electron withdrawing group affects reactivity. Starting with a perfluorobenzene (8K), it seems that inductively withdrawing groups do not increase *k*_inact_ at all, and fluorine at this position significantly decreases binding affinity, similar to what was observed with an α-fluoroacrylamide warhead on the EB-2-16 scaffold.^20^ Interestingly, while introducing and increasing the strength of a conjugated electron withdrawing group does increase *k*_inact_, this effect is not as strong as expected (*e.g.*, 8L*vs.*8M), and *k*_inact_ seems to plateau around a value of 1 min^−1^, regardless of electron withdrawing group strength beyond a cyano group. However, again it seems that the presence of an electron withdrawing group containing a hydrogen bond acceptor, specifically one that contains a double-bonded oxygen, is required for optimal binding affinity (*e.g.*, 8L and 8N).

Previously we showed that a propiolamide warhead on the EB-2-16 scaffold had a *k*_inact_ value of 6.83 ± 1.75 min^−1^.^20^ Compounds 8A–O are not propiolamides, as their keto-alkyne moiety is directly attached to the triazole ring, so there is very little attenuation of electrophilicity due to resonance from this side of the molecule. What is peculiar here, is that despite the high intrinsic reactivity of these compounds (see *Intrinsic reactivity*), their *k*_inact_ values are relatively low. Given the generally higher binding affinity that this scaffold shows compared to EB-2-16, it seems that it is able to bind tightly to hTG2, but this bound conformation does not optimally position the warhead relative to Cys277 for reaction, resulting in lower *k*_inact_ values. This could be due to improper positioning of these sterically hindered warheads in the active site tunnel, but it could also suggest a binding mode entirely different from the one proposed in (Fig. 4), perhaps accessing a pocket that has not yet been discovered crytallographically, since all crystal structures have been obtained with peptidomimetic inhibitors. The best inhibitor of this series was the methylsulfone derivative 8N, which allowed us to retain excellent inhibitor efficiency while replacing the second nitro group.

We also evaluated a variety of compounds with attenuated warhead reactivity. Inhibitor 13 features an internal propargyl alcohol rather than a keto-alkyne, in which the alkyne is much less activated (less electron deficient). Surprisingly, this inhibitor did still display time-dependent irreversible inhibition with hTG2, albeit with a very low *k*_inact_ value of 0.06 ± 0.01 min^−1^ and lower binding affinity. Next, we investigated a variety of propiolamide derivatives, starting with a compound (16) inspired from a benzotriazole-cinnamoyl adduct (CP15n), from which CP4d was first discovered.^19^ Interestingly, this compound was a very potent but reversible inhibitor with a *K*_i_ of 190 nM (see SI). This suggests that having the triazole group and phenyl groups closer to the warhead in a fused ring structure impacted binding affinity very positively, but disrupted the positioning of the warhead such that it could no longer react with Cys277 at all. Strangely, it seems that minor structural changes can have significant impact on the exact binding site/pose of these compounds, without perturbing the overall binding affinity.

Other, more classic propiolamide derivatives 18 and 23A–B showed substantially reduced potency, largely due to a decrease in binding affinity, suggesting that more distance between the triazole and warhead leads to poorer binding. With the 2-atom spacer between the triazole and the warhead in compound 18, no significant inhibition was detected. However, with the 1-atom spacer in 23A–B, appreciable binding affinity had returned. The only difference between 23A and 23B is the substituent at the 1-position of the triazole. The *para*-cyanobenzyl derivative showed roughly 2-fold higher potency than the tetrahydropyranyl derivative. This is consistent the previous findings with compounds 8D and 8G (Table 1), confirming that there were no unusual cooperativity effects taking place when altering substituents at the 1 and 4-positions of the triazole together.

### EB-2-16 and 8A hybrids

Compounds bearing elements of both the EB-2-16 and 8A scaffold were evaluated to determine if any cooperativity existed between their binding modes (Table 3). Inhibitor 28 features a *para*-nitrophenyl group in the place of the adamantane of EB-2-16. This derivative showed ∼50-fold reduction in binding affinity compared to EB-2-16. This suggests that the substituent at the 1-position of the triazole in 8A is not similar in its binding pose/interactions as the hydrophobic unit of EB-2-16.

**Table 3:** Kinetic parameters of EB-2-16 and 8A hybrids

Inh.	Structure	*k*_inact_ (min^−1^)	*K*_I_ (μM)	*k*_inact_/*K*_I_ (×10^3^ M^−1^ min^−1^)
28	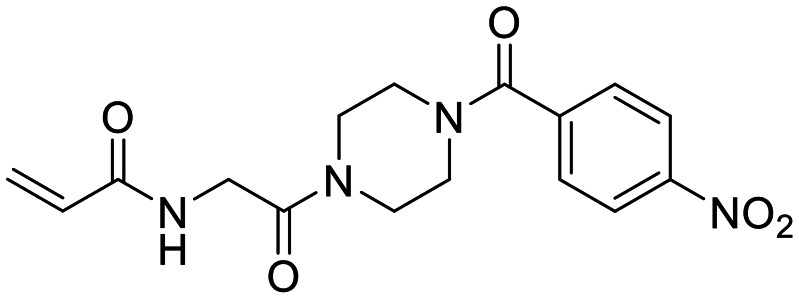	2.17 ± 0.45	181 ± 54	12.0 ± 4.3
33	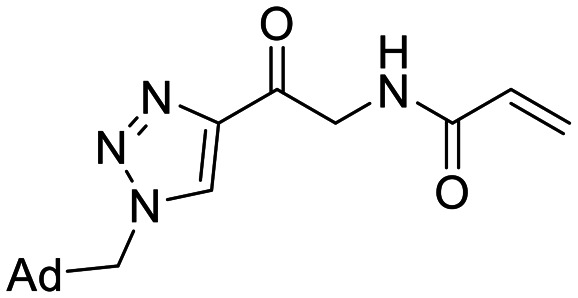	2.24 ± 0.56	16.9 ± 5.5	133 ± 54
36^a^	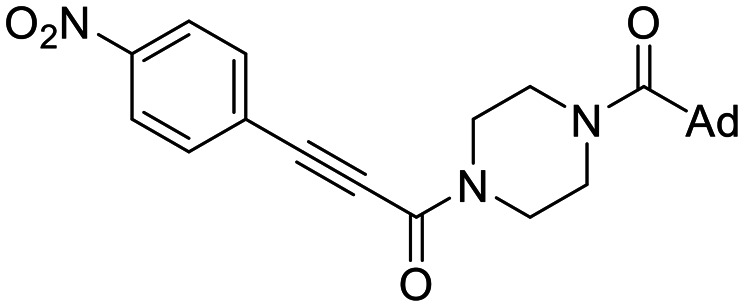	0.40 ± 0.22	17.7 ± 12.5	22.7 ± 3.8
38^b^	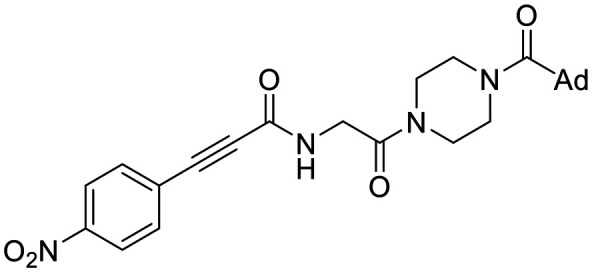	n.d.	n.d.	n.d.
40^b^	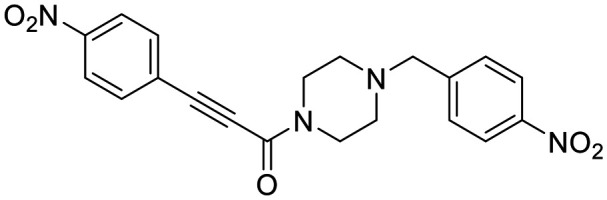	n.d.	n.d.	n.d.

a Determined by double reciprocal fitting.

b No inhibition detected up to solubility limit of compound (50 μM).

Inhibitor 33 contains a triazole ‘spacer’ unit in between the hydrophobic moiety and glycine linker/warhead component, instead of a piperazine. It should be noted, however, that the hydrophobic moiety does not contain a carbonyl linkage to the triazole like it does to piperazine in the parent compound. This is due to the fact that a click reaction with an acyl azide precursor would be needed to retain the carbonyl, and because this is synthetically impractical, we carried forward with a methylene linkage instead. Inhibitor 33 showed ∼4-fold decrease in binding affinity, but it was still a relatively efficient inhibitor. It is unclear whether the reduction in binding affinity came solely from replacing the triazole with the piperazine and/or removing the carbonyl linkage. However, given the fact that compound 40, in which the triazole of 8A was replaced with a piperazine, showed no significant inhibition at all, there seems to be little cooperativity between these ‘spacer’ units either.

Compounds 36 and 38 contain the warhead of 8A, but with the rest of the EB-2-16 scaffold, without and with the glycine linker respectively. Again, these compounds display significantly reduced potency compared to the parent scaffolds. However, it is interesting to note that the smaller size/length of 36, which is more similar to the size of 8A, shows greater potency than 38.

Taken together, these hybrid compounds reveal that the binding pose of these two small molecule scaffolds is not likely to be similar and that their optimization will require significantly different considerations when considering the types of interactions different structural elements may be engaged in. This is notably very different from traditional peptidic inhibitors that contain very similar structural features and binding modes.

### Optimization of inhibitor potency

The results of the aforementioned SAR analyses suggested that the nitro group on the terminal phenyl group of the warhead could be replaced with a methylsulfone and that a *para*-cyanobenzyl group at the 1-position of the triazole increased binding affinity. Therefore, we combined these elements together to give inhibitor 8P. The synergy of these two changes pushed the inactivation constant (*K*_I_) down into the nanomolar range, while retaining a modest *k*_inact_ (Table 4). We also synthesized and evaluated a derivative with a dimethylsulfonamide instead of a methylsulfone (8Q); however, this compound showed relatively poor solubility in our kinetic assay, and its overall inhibition efficiency was also lower. To achieve solubilizing effects similar to those of the tetrahydropyranyl moiety of 8D, we replaced the simple benzene ring with a pyridine in inhibitor 8R. This compound displayed the highest inhibitor efficiency among all compounds (*k*_inact_ = 0.43 ± 0.02 min^−1^, *K*_I_ = 0.48 ± 0.05 μM, *k*_inact_/*K*_I_ = 910 ± 113 × 10^3^ M^−1^ min^−1^), representing one of the most potent non-peptidic small molecule hTG2 inhibitors to date.

**Table 4:** Kinetic parameters of the most potent inhibitors

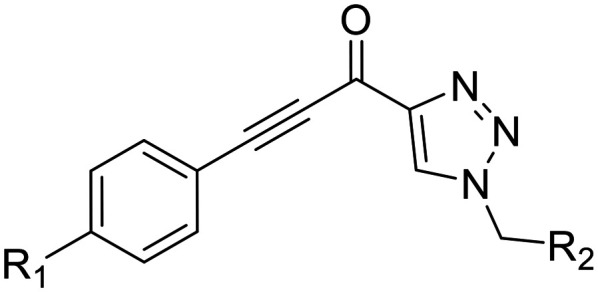					
Inh.	R1	R2	*k*_inact_ (min^−1^)	*K*_I_ (μM)	*k*_inact_/*K*_I_ (×10^3^ M^−1^ min^−1^)
8P	SO_2_Me	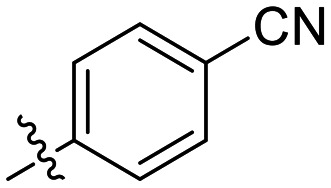	0.42 ± 0.01	0.52 ± 0.03	802 ± 50
8Q	SO_2_NMe_2_	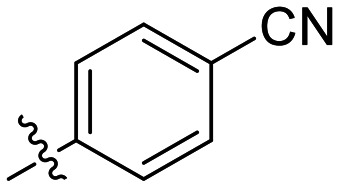	0.14 ± 0.01	0.22 ± 0.03	604 ± 82
8R	SO_2_Me	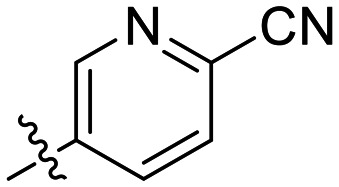	0.43 ± 0.02	0.48 ± 0.05	910 ± 113

**Fig. 5:**
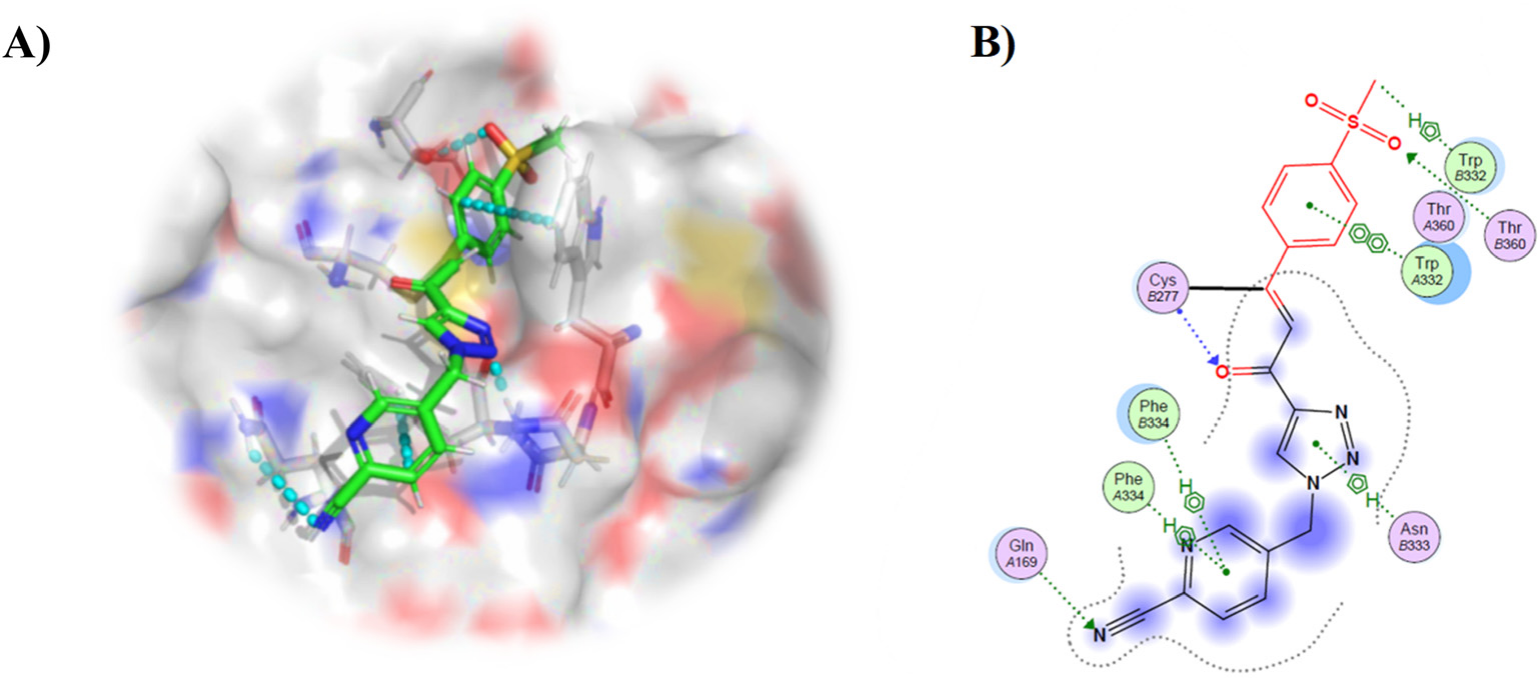
Molecular modelling of 8R in crystal structure PDB: 3S3S. (A) Molecular model with key ligand-protein interactions shown by cyan dotted lines. (B) Ligand interaction map (see Materials and methods).

Repeating molecular modelling with the most potent inhibitor 8R revealed some interesting interactions that may contribute to increased potency (and general affinity of this scaffold). In the binding pose shown in Fig. 5, the sulfonylphenyl group is engaged in pi-pi stacking with Trp332 and hydrogen bonding with Thr360, the triazole forms a hydrogen bond with Asn333, and the cyanopyridine group is positioned to engage in a pi-pi stacking interaction with Phe334 and a hydrogen bond with Gln169 *via* the nitrile group. The increased affinity observed with 8P and 8R compared to 8A may be due to the better positioning of the nitrile group, relative to the nitro group, to act as a hydrogen bond acceptor.

### Isozyme selectivity

**Fig. 6:**
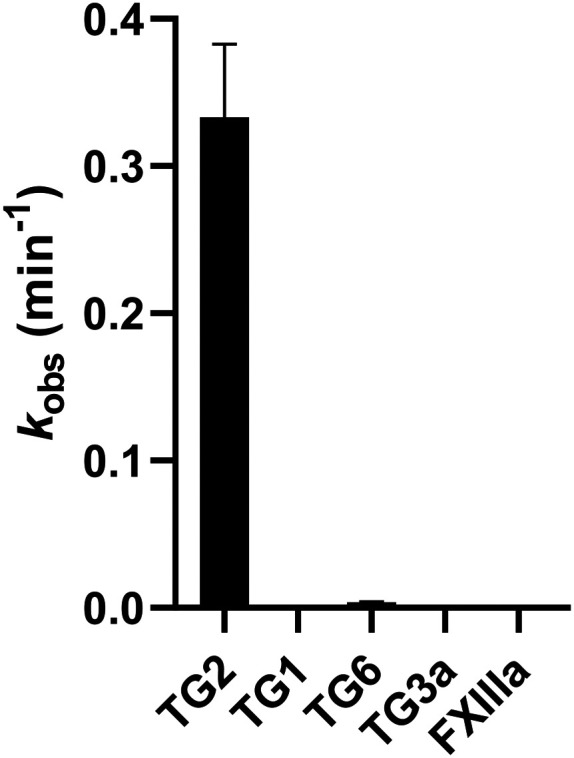
Isozyme selectivity of 8R at [I]/*α* = 0.73 μM with each isozyme.

Since hTG2 is part of a family of TGases it is always important to establish the selectivity of new scaffolds against a panel of other, therapeutically relevant TGases, namely TG1, TG6, TG3a, and FXIIIa. The AL5 assay described above for hTG2 inhibition was also used to monitor hTG1 and hTG6 inhibition,^16^ whereas for hTG3a and hFXIIIa, a commercially available continuous fluorescence assay that uses a peptidic FRET-quenched substrate was used.^32,33^ In each case the same effective inhibitor concentration ([I]/*α*, where *α* = (1 + ([S]/*K*_M_)), to account for competition with substrate) was used for each enzyme/substrate pair in the assays, in order to permit comparison between isozymes. A single *k*_obs_ value was determined, in triplicate, for each isozyme with inhibitor 8R (see SI).^16^ As shown in Fig. 6 and the SI (page S12), 8R does not show significant inhibition of any of the other isozymes. In fact, this scaffold shows better selectivity than EB-2-16,^20^ and our other previously reported peptidomimetic scaffolds,^14^ making 8R one of the most selective non-peptidic hTG2 inhibitor scaffolds reported to date.

### Inhibition of GTP binding

**Fig. 7:**
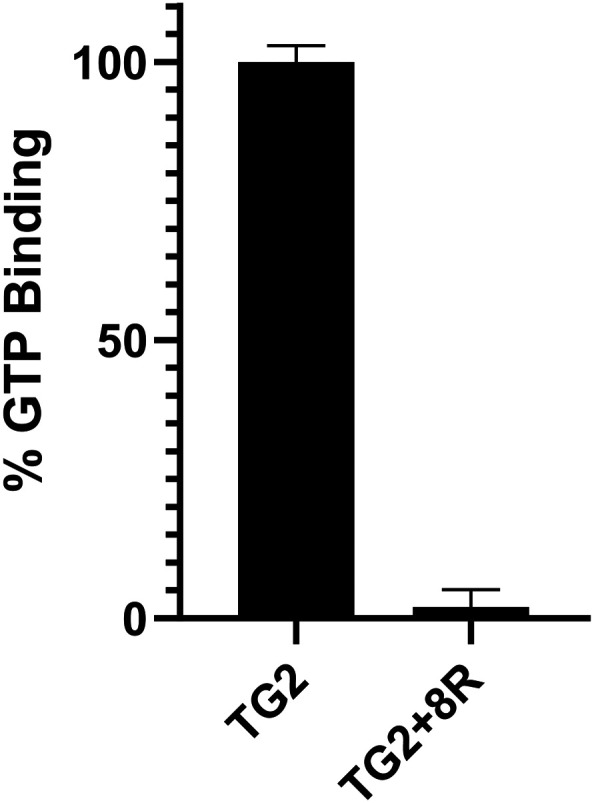
Inhibition of GTP binding by 8R at [I] = 0.92 μM.

One of the key advantages of covalent modification of Cys277 of hTG2 is that it locks hTG2 in its open conformation and thereby abolishes its ability to bind GTP. We have recently shown that molecules as small as acrylamide itself completely lock hTG2 in its open conformation, even if they have poor binding affinity.^34^ Therefore, it appears that any compound covalently modifying Cys277 will abolish GTP binding. Compound 8R was evaluated for its impact on GTP binding using our previously reported assay.^34^ The enzyme was incubated with and without inhibitor and then subjected to dialysis to wash out any unbound inhibitor. Both samples were then mixed with a nonhydrolyzable GTP analogue (GTPγS FL BODIPY) whose fluorescence increases when bound to hTG2. As shown in Fig. 7, enzyme incubated with inhibitor showed negligible GTP binding compared to the uninhibited control. This provides further validation that the alkynyl scaffold is in fact irreversible and likely reacting with Cys277.

### Intrinsic reactivity of alkynyl warheads

Although none of the warheads with attenuated reactivity were able to react/bind as efficiently with hTG2 as 8A, this type of internal warhead is underexplored in the field of TCIs. Therefore, we further investigated the intrinsic reactivity of different alkynyl warhead analogues by measuring the corrected second order rate constants (*k*^corr^_2_) of their reaction with glutathione (GSH), in solution, under pseudo-first order conditions, at room temperature (Table 5). Reactions were carried out at pH 7.4 (phosphate buffer) or pH 10.4 (CAPS buffer) depending on how reactive the compound was and if rate acceleration was needed by increasing the pH to observe the reaction over a reasonable time frame. Corrected second order rate constants (*i.e.*, corrected for pH and accounting for [GS^−^] in solution) provide a much more robust picture of reactivity than half-lives or pseudo-first order rate constants alone, as they are not dependent on assay conditions and allow direct comparison between literature values.^35^

**Table 5:** Intrinsic reactivity of representative alkynyl warheads and their enzymatic efficiency ratios

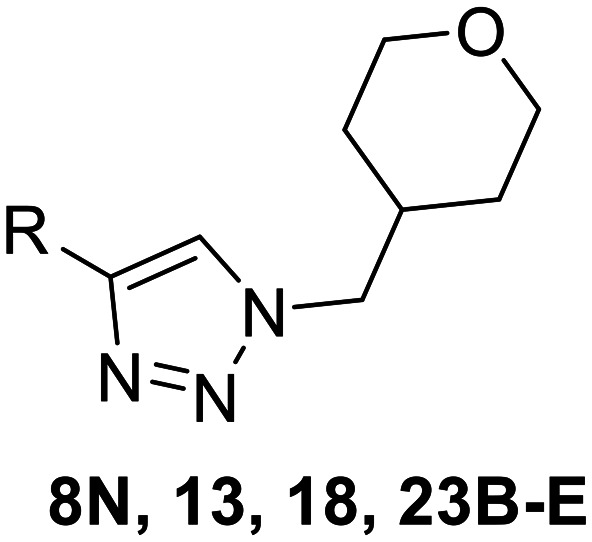			
Cpd.	R/Structure	*k* ^corr^_2_ (M^−1^ min^−1^)	∼(*k*_inact_/*K*_I_)/*k*^corr^_2_
8N	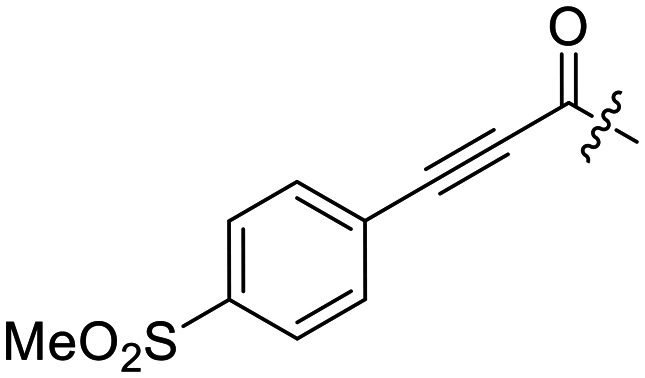	>100	—
13	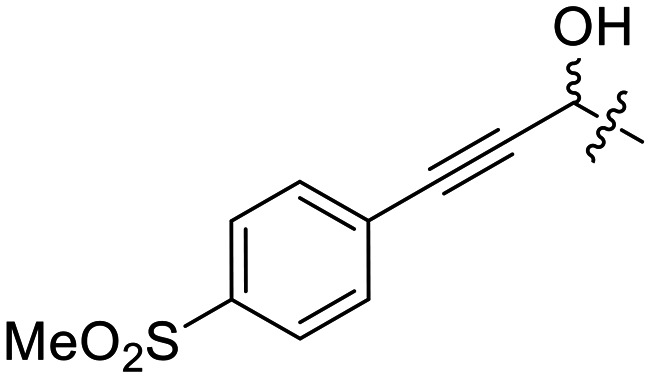	0.12 ± 0.01	28 000
16	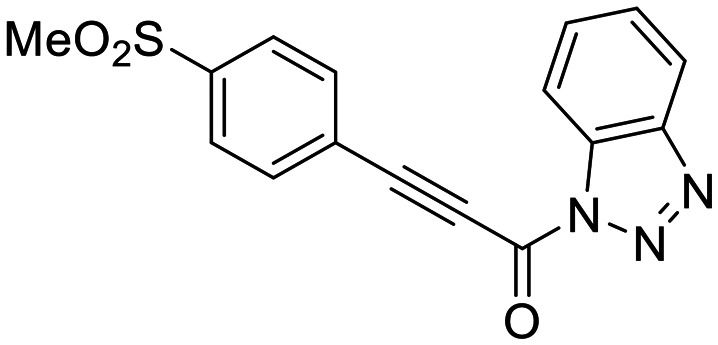	>100	—
18	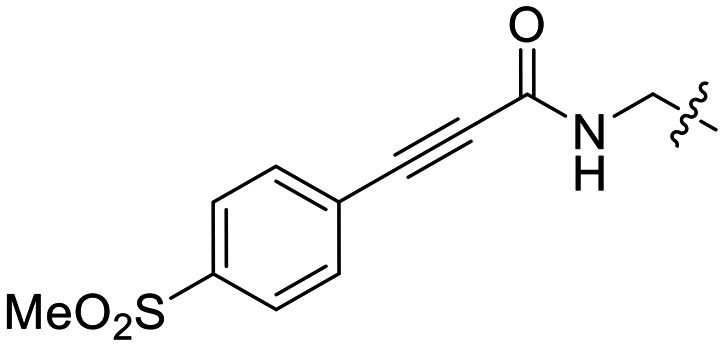	4.85 ± 0.19	—
23B	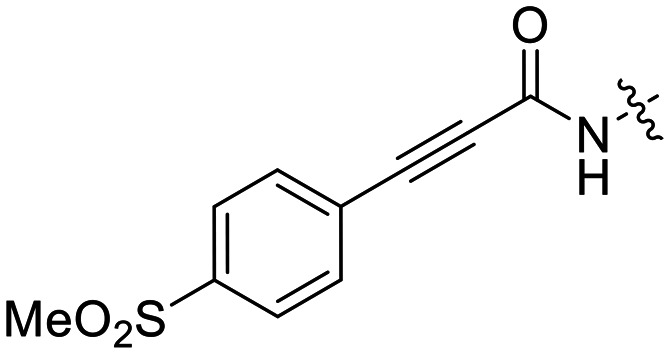	36.7 ± 1.0	600
23C	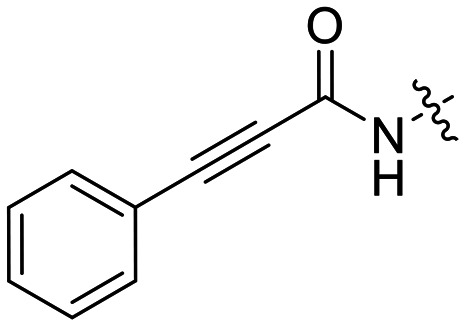	11.2 ± 0.31	—
23D	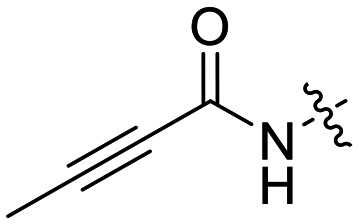	0.51 ± 0.08	—
23E	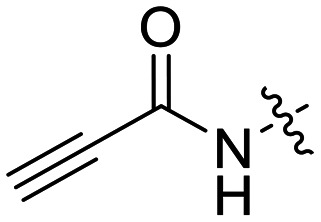	69.5 ± 4.81	—

We started with compound 8N as a representative of the potent alkynyl hTG2 inhibition scaffold reported herein. Unfortunately, this compound showed rapid reaction with GSH and was highly unstable (*t*_1/2_ ≤ 10 min at pH 7.4). This is likely due to the fact there are two strong electron withdrawing groups attached directly to the alkyne (*para*-methylsulfone phenyl and ketone) making it a highly activated electrophile. Compound 16, a potent reversible inhibitor, also showed rapid consumption and instability in this assay. Due to its poor solubility, its reactivity with GSH was measured by ^1^H NMR in DMSO-*d*_6_ (see SI, page S13). Even in non-aqueous conditions, its half-life was under 10 minutes. With this compound, it is plausible that along with the alkyne, the amide bond could be cleaved by glutathione (or in solution during kinetic assays) due to the fact that benzotriazole is a good leaving group. For this reason, we also tested benzotriazole itself and intermediate 15 (Scheme 4) in our inhibition assay to see if inhibition actually resulted from one of these individual precursors rather than the compound itself due to hydrolysis. However, none of these precursors showed any inhibition of hTG2 on their own (see SI, page S9), suggesting that the inhibition observed in our assay was not due to degradation products. The evaluation of 8N and 16 provided half-lives that were less than 10 min, allowing us to set a limit of their *k*^corr^_2_ values as being >100 M^−1^ min^−1^ (see *Materials and methods* for related equations).

The alkyne in compound 13 is not conjugated to a carbonyl, which explains why it is the least intrinsically reactive compound in this series with a *k*^corr^_2_ of 0.12 ± 0.01 M^−1^ min^−1^. However, the conjugated methylsulfonylbenzene ring still provides significant activation, such that this compound is only about 4–8 fold less reactive than a standard *N*-alkyl acrylamide warhead, which typically have *k*^corr^_2_ values of ∼0.4–0.8 M^−1^ min^−1^ at room temperature,^20,29,36^ suggesting that it is still a viable warhead, especially if it offers greater binding affinity with specific targets.

We also tested a variety of propiolamide derivatives with different terminal alkyne substituents. Compound 18, featuring an *N*-alkyl amide linkage showed a *k*^corr^_2_ = 4.85 ± 0.19 M^−1^ min^−1^, whereas an analogue with an *N*-triazole amide linkage (23B) has a *k*^corr^_2_ = 36.7 ± 1.0 M^−1^ min^−1^. This is likely because the electron donating capability of the nitrogen is stronger in the *N*-alkyl linkage, whereas the *N*-triazole linkage splits the electron donation through resonance between the adjacent carbonyl and the triazole, making the warhead overall about 10-fold more reactive. This is very similar to the difference in reactivity between *N*-alkyl and *N*-phenyl acrylamides.^35,36^

Next, we explored the effect of the methylsulfone group on the terminal phenyl ring. Removing this electron withdrawing group in compound 23C decreased the *k*^corr^_2_ ∼ 4-fold to 11.2 ± 0.08 M^−1^ min^−1^ compared to 23B, revealing the significant contribution of this electron withdrawing group to warhead reactivity. Removing the phenyl group and replacing it with a methyl group (23D) resulted in a significant increase in stability, with a *k*^corr^_2_ = 0.52 ± 0.08 M^−1^ min^−1^. This is an interesting result, as it seems that the steric hindrance conferred by the methyl group far outweighs any steric hindrance effects imparted by the phenyl group, and that the electronic effects of the phenyl group dominate. It is likely that, when directly attached to the alkyne, the planar phenyl ring is orthogonal to the anti-bonding pi-orbitals of the α,β-unsaturated carbonyl system. However, a tetrahedral methyl group is free to rotate and partially block attack from the incoming nucleophile. The stabilizing effects of the methyl group are also seen in compound 23E, where replacing the methyl group with a hydrogen (*i.e.*, effectively removing the terminal substituent) resulted in significant loss of stability (∼140-fold) with a *k*^corr^_2_ = 69.5 ± 5.81 M^−1^ min^−1^.

The *k*_inact_/*K*_I_ ratio represents the second order rate constant for reaction between an inhibitor and enzyme, taking into account both binding affinity and reactivity. On the other hand, *k*^corr^_2_ represents the second order rate constant for the reaction of inhibitor with glutathione thiolate (GS^−^). We divided (*k*_inact_/*K*_I_) by *k*^corr^_2_ for every inhibitor for which these values were measured, to provide an ‘enzymatic efficiency ratio’ (EER), as we have previously described.^20^ This ratio gives information about how well the enzyme is able to facilitate thiol addition to the inhibitor, compared to GSH in solution. A higher ratio indicates higher expected on-target reactivity and selectivity. Interestingly, compound 13, which is a less efficient inhibitor than 23B, shows an EER of ∼28 000 (Table 5), roughly 50-fold higher than the EER obtained with 23B (∼600), meaning the enzyme is much more selective in facilitating the reaction with the intrinsically low reactivity warhead, likely due to binding affinity and/or conformational effects. This is generally true and consistent with what we found in our previous warhead screening study on EB-2-16.^20^ However, for good lead candidates this ratio should be on the order of millions,^20^ which is far above the EER values of our current series.

These results point to some routes that may be explored to improve the stability and efficiency of these inhibitors. While it seems that propiolamide derivatives show significant decrease in binding affinity, and that the pharmacophore is very sensitive to changes at the 4-position of the triazole, attempts can be made to further improve the binding affinity of analogues like 13 and 23A/B, as they show modest inhibition efficiency and stability. Additionally, although attempts to synthesize a propargyl derivative of our scaffold (Scheme 6) by introducing a methylene unit between the alkyne and the phenyl ring were unsuccessful, it is likely that this type of modification, in principle, will increase stability similarly to a terminal methyl group. However, this type of modification will likely require more than one methylene unit to prevent allene formation, as well as more complex synthetic routes.

## Conclusions

In this study, we revisited our previously reported reversible hTG2 inhibitor scaffold CP4d and discovered a unique and highly potent internal alkynyl scaffold (8A) that irreversibly inhibits hTG2. Comprehensive kinetic SAR analysis revealed how modifications to the triazole 1-substituent, warhead reactivity, and incorporation of elements from the EB-2-16 scaffold affect both binding affinity (*K*_I_) and reactivity (*k*_inact_). These insights guided the design of inhibitor 8R, in which potentially toxic nitrophenyl groups were replaced with methylsulfonyl-phenyl and cyano-pyridinyl substituents, while improving solubility and inhibitor efficiency substantially. With a *k*_inact_ of 0.43 ± 0.02 min^−1^, a *K*_I_ of 0.48 ± 0.05 μM, and a *k*_inact_/*K*_I_ of 910 ± 113 × 10^3^ M^−1^ min^−1^, 8R ranks among the most effective non-peptidic hTG2 inhibitors reported to date. It also demonstrates excellent isozyme selectivity and fully abolishes GTP binding.

Hybrid derivatives combining the scaffolds of 8A and EB-2-16 showed no cooperative inhibition, suggesting distinct binding poses and underscoring the need for structural elucidation of non-peptidic hTG2 inhibitors *via* crystallography or cryo-EM to enhance rational structure-based design. Investigation into the intrinsic reactivity of various internal alkynyl warheads highlighted the stabilizing effect of terminal methyl groups and the significant influence of extended conjugation and electron-withdrawing groups on reactivity. Although the most potent inhibitors have limited stability, promising strategies such as propiolamide and propargyl derivatives have been identified for future optimization.

Overall, this work establishes a novel internal alkynyl warhead scaffold for irreversible hTG2 inhibition that not only provides a valuable chemical biology tool but also expands the scope of covalent warheads beyond traditional terminal electrophiles, with potential applications extending beyond hTG2.

## Materials and methods

### Synthesis

#### General remarks

All reagents and solvents were purchased from commercial sources and used without further purification. Anhydrous solvents were obtained using a Phoenix Solvent Dispensing System (JC Meyer Solvent Systems) with neutral alumina-packed columns. Thin layer chromatography was performed using EMD aluminum-backed silica 60 F254-coated plates and were visualized using either UV-light (254 nm), KMnO_4_, Hanessian's stain, or ninhydrin stain. Column chromatography was carried out using standard flash technique with silica (Siliaflash-P60, 230–400 mesh Silicycle) under compressed air pressure. ^1^H-NMR spectra were obtained at 300, 400, 500, or 600 MHz, ^13^C-NMR spectra were obtained at 75, 100, 125, or 150 MHz, and ^19^F-NMR spectra were obtained at 283 MHz on Bruker instruments. NMR chemical shifts (*δ*) are reported in ppm and are calibrated against residual solvent signals of CDCl_3_, DMSO-*d*_6_, or CD_3_OD. Spectra were processed using MNOVA 14.1.2 (Mestrelab Research S.L., Santiago de Compostela, Spain). Fourier transform was conducted using a linear phase shift group delay, an exponential-fit 1 Hz apodization, and a zero-filling linear prediction of 65 536 points. An automatic phase correction was applied and manually refined to provide a flatter baseline. A baseline correction, employing a Whittaker smoothing function with a 2.60 Hz filter and a smoothing factor of 16 384 was employed on the spectrum between −2.00 and 15.00 ppm. Integration was measured using the manual integration tool and referenced to a relevant proton. Multiplet analysis was conducted using the algorithm as implemented in the software, confirmed by manual analysis. High resolution mass spectrometry (HRMS) was performed using a quadrupole time-of-flight (QTOF) or magnetic sector analyzer and electrospray ionization (ESI) or electron impact (EI), respectively. All measured *m*/*z* values were within 3 mmu of the calculated value (generally < 10 ppm discrepancy) unless otherwise noted. High performance liquid chromatography (HPLC) purity traces were collected on a Gilson-Mandel GXP271 with UV detection at 214 and 254 nm (Phenomenex Luna, 150 mm × 4.6 mm, 30 min, 1.5 mL min^−1^ flow rate, 5–95% or 20–80%, CH_3_CN with 0.1% TFA in H_2_O with 0.1% TFA, 30 min method).

### 1-Iodo-4-(methylsulfonyl)benzene (1F)

To a solution of 4-(methylsulfonyl)aniline (2.00 g, 11.68 mmol, 1.0 eq.) in H_2_O (10 mL) and conc. HCl (2.5 mL) at 0 °C was added a solution of NaNO_2_ (0.88 g, 12.84 mmol, 1.1 eq.) in H_2_O (2 mL) dropwise. The reaction was stirred for 1 h and then a solution of KI (2.12 g, 12.84 mmol, 1.2 eq.) in H_2_O (2 mL) was added dropwise at 0 °C. The reaction was allowed to warm to R.T. and then refluxed for 1 h. The reaction mixture was then cooled to 0 °C prior to addition of sat. NaHSO_3_ (100 mL). The aqueous solution was extracted with Et_2_O (3 × 50 mL) and then washed with brine (100 mL). The organic layer was then dried over MgSO_4_ and concentrated *in vacuo* to give a yellow solid which was purified by flash column chromatography (30% EtOAc/Hex *R*_f_ = 0.47) to give a white powder (1.05 g, 35%). ^**1**^**H NMR** (500 MHz, CDCl_3_) *δ* 7.99 (d, *J* = 8.6 Hz, 2H), 7.70 (d, = 8.6 Hz, 2H), 3.09 (s, 3H). ^**13**^**C NMR** (125 MHz, CDCl_3_) *δ* 140.4, 138.8, 128.9, 101.7, 44.6. Characterization data are consistent with previously reported values.^37^

### 1-Bromo-4-((trifluoromethyl)sulfonyl)benzene (1G)

To a solution of 4-bromophenyl trifluoromethyl sulfide (0.30 mL, 1.94 mmol, 1.0 eq.) in DCM (20 mL) at 0 °C was added *m*CPBA (1.67 g, 9.70 mmol, 5.0 eq.) portion wise. The reaction mixture was allowed to warm to R.T while stirring overnight. The reaction mixture was then diluted with DCM (100 mL) and washed with sat. NaHCO_3_ (2 × 100 mL) and then brine (100 mL) followed by drying over MgSO_4_ and concentrating *in vacuo*. The residue was purified by flash column chromatography (5% EtOAc/Hex, *R*_f_ = 0.45, dry loading using celite) to give a white solid (0.50 g, 89%). ^**1**^**H NMR** (300 MHz, CDCl_3_) *δ* 7.90 (d, *J* = 9.0 Hz, 2H), 7.83 (d, *J* = 9.0 Hz, 2H). ^**13**^**C NMR** (75 MHz, CDCl_3_) *δ* 119.8 (q, *J*_C,F_ = 324.3 Hz), 130.4, 132.3, 133.0, 133.4. ^**19**^**F NMR** (282 MHz, CDCl_3_) *δ* –78.25. Characterization data are consistent with previously reported values.^38^

### 2-(Prop-2-yn-1-yloxy)tetrahydro-2*H*-pyran (2B)

To a solution of propargyl alcohol (1.10 mL, 17.84 mmol, 1.0 eq.) in DCM (15 mL) were added dihydropyran (2.50 mL, 26.76 mmol, 1.5 eq.) and 4 drops of 12.5 M HCl. The reaction mixture was stirred vigorously and R.T. for 24 h, after which it was quenched with sat. NaHCO_3_ (50 mL). The organic layer was separated, and the aqueous layer was further extracted using DCM (3 × 50 mL). The combined organic layer was washed with brine (150 mL) and dried over MgSO_4_ before being concentrated *in vacuo* to give a colourless oil (2.14 g, 86%) ^**1**^**H NMR** (300 MHz, CDCl_3_) *δ* 4.83 (t, *J* = 3.0 Hz, 1H), 4.27 (dd, *J* = 15.6, 2.4 Hz, 2H), 3.90–3.74 (m, 1H), 3.60–3.47 (m, 1H), 2.42 (t, *J* = 2.4 Hz, 1H), 1.92–1.44 (m, 6H, overlapping with H_2_O). ^**13**^**C NMR** (75 MHz, CDCl_3_) *δ* 96.8, 79.7, 73.9, 61.9, 53.9, 30.2, 25.3, 19.0. Characterization data are consistent with previously reported values.^39^

### General procedure 1 (**GP**1): Sonogashira reaction^24^

In a flame-dried round-bottom flask filled with N_2_ were added *para* substituted aryl iodide or aryl bromide (1A–H) (1.0 eq., commercially available unless indicated otherwise), PdCl_2_(PPh_3_)_2_ (2 mol%) and CuI (2 mol%). The solids were dissolved in anhydrous MeCN (∼1 M) followed by the addition of NEt_3_ (4.0 eq.). The reaction mixture was stirred for 10 min at R.T. followed by dropwise addition of propargyl alcohol (2A) or THP-protected propargyl alcohol (2B) (1.2 eq.). The reaction was heated to reflux and stirred for 2–24 h. The reaction was concentrated *in vacuo* and then diluted with H_2_O (100 mL) and EtOAc (100 mL) and filtered through celite. The layers of the filtrate were separated, and the aqueous layer was extracted with EtOAc (2 × 100 mL). The combined organic layer was washed with sat. NH_4_OH (100 mL) and then water (100 mL) followed by drying over MgSO_4_ and concentrating *in vacuo*. The residue was purified by flash column chromatography as described below.

### 3-(4-Nitrophenyl)prop-2-yn-1-ol (3A)

Synthesized according to **GP**1 using 4-iodonitrobenzene (1A) (1.24 g, 5.00 mmol) and purified by flash column chromatography (50% EtOAc/Hexanes, *R*_f_ = 0.61, dry loading using celite) to give an orange solid (0.74 g, 84%). ^**1**^**H NMR** (300 MHz, CDCl_3_) *δ* 8.19 (d, *J* = 8.7 Hz, 2H), 7.58 (d, *J* = 8.7 Hz, 2H), 4.54 (s, 2H), 1.70 (br s, overlapping with H_2_O, 1H). ^**13**^**C NMR** (75 MHz, CDCl_3_) *δ* 147.3, 132.4, 129.4, 123.6, 92.4, 83.8, 51.5. Characterization data are consistent with previously reported values.^24^

### 3-Phenylprop-2-yn-1-ol (3B)

Synthesized according to **GP**1 using 4-iodobenzene (1B) (1.00 g, 4.90 mmol) and purified by flash column chromatography (30% EtOAc/Hex, *R*_f_ = 0.39, dry loading using celite) to give a yellow solid (0.68 g, >95%). ^**1**^**H NMR** (400 MHz, CDCl_3_) *δ* 7.46–7.43 (m, 2H), 7.35–7.31 (m, 3H), 4.52 (s, 2H), 1.72 (br d, *J* = 2.0 Hz, 1H). ^**13**^**C NMR** (100 MHz, CDCl_3_) *δ* 131.7, 128.5, 128.3, 122.5, 87.7, 85.7, 51.6. Characterization data are consistent with previously reported values.^23^

### 2-((3-(Perfluorophenyl)prop-2-yn-1-yl)oxy)tetrahydro-2*H*-pyran (3C1)

Synthesized according to **GP**1 using iodopentafluorobenzene (1C) (1.00 mL, 7.50 mmol), 2B (0.87 g, 6.45 mmol), and purified by flash column chromatography (10% EtOAc/Hex, *R*_f_ = 0.53, dry loading using celite) to give a colourless oil (1.50 g, 65%). ^**1**^**H NMR** (300 MHz, CDCl_3_) *δ* 4.88 (t, *J* = 3.1 Hz, 1H), 4.54 (s, 2H), 4.01–3.77 (m, 1H), 3.61–3.54 (m, 1H), 1.97–1.41 (m, 6H). ^**13**^**C NMR** (75 MHz, CDCl_3_) *δ* 148.4–148.3 (m), 146.8–146.6 (m), 142.6–142.4 (m), 140.9–140.7 (m), 138.5–138.3 (m), 136.9–136.6 (m), 99.7–99.5 (m), 98.2, 97.1, 69.8, 62.1, 54.5, 30.2, 25.3, 18.9. ^**19**^**F NMR** (282 MHz, CDCl_3_) *δ* −135.79–−135.92 (m, 2F), −152.17–−152.34 (m, 1F), −161.66–−161.86 (m, 2F). **HRMS (EI)** calc'd for C_14_H_11_F_5_O_2_ [M˙]^+^: 306.0679, found: 306.0702.

### 3-(Perfluorophenyl)prop-2-yn-1-ol (3C2)

To a solution of 3C1 (1.40 g, 4.90 mmol, 1.0 eq.) in MeOH (15 mL) was added *p*TsOH·H_2_O (0.10 g, 0.49 mmol, 0.1 eq.). The reaction was stirred for 1 h at R.T. at which point it was confirmed complete by TLC analysis. The reaction was concentrated *in vacuo*, and the residue was purified by flash column chromatography (10% EtOAc/Hex, *R*_f_ = 0.16, dry loading using celite) to give a white crystalline solid (0.92 g, 85%). ^**1**^**H NMR** (300 MHz, CDCl_3_) *δ* 4.60 (s, 2H), 1.93 (br s, 1H). ^**13**^**C NMR** (75 MHz, CDCl_3_) *δ* 148.4–148.2 (m), 146.7–146.5 (m), 142.7–142.5 (m), 140.9–140.7 (m), 138.5–138.3 (m), 136.9–136.6 (m), 99.8, 99.5–99.2 (m), 69.7, 51.5. ^**19**^**F NMR** (282 MHz, CDCl_3_) *δ* −135.90–−136.03 (m, 2F), −151.82–−151.98 (m, 1F), −161.46–−161.66 (m, 2F). **HRMS (EI)** calc'd for C_9_H_3_F_5_O [M˙]^+^: 222.0104, found: 222.0091.

### 1-(4-(3-Hydroxyprop-1-yn-1-yl)phenyl)ethan-1-one (3D)

Synthesized according to **GP**1 using 4-iodoacetophenone (1D) (1.00 g, 4.06 mmol) and purified by flash column chromatography (10% EtOAc/DCM, *R*_f_ = 0.43, dry loading using celite) to give a yellow solid (0.65 g, 92%). ^**1**^**H NMR** (300 MHz, CDCl_3_) *δ* 7.92 (d, *J* = 8.5 Hz, 2H), 7.53 (d, *J* = 8.5 Hz, 2H), 4.54 (s, 2H), 2.61 (s, 3H). ^**13**^**C NMR** (75 MHz, CDCl_3_) *δ* 197.3, 136.5, 131.8, 128.2, 127.4, 90.4, 84.9, 51.6, 26.6. Characterization data are consistent with previously reported values.^23^

### 4-(3-Hydroxyprop-1-yn-1-yl)benzonitrile (3E)

Synthesized according to **GP**1 using 4-iodobenzonitrile (1E) (1.00 g, 4.37 mmol) and purified by flash column chromatography (50% EtOAc/Hex, *R*_f_ = 0.63, dry loading using celite) to give a yellow solid (0.60 g, 87%). ^**1**^**H NMR** (400 MHz, CDCl_3_) *δ* 7.59 (d, *J* = 8.4 Hz, 2H), 7.50 (d, *J* = 8.4 Hz, 2H), 4.51 (s, 2H). ^**13**^**C NMR** (100 MHz, CDCl_3_) *δ* 132.1, 132.0, 127.5, 118.3, 111.8, 91.7, 83.9, 51.4. Characterization data are consistent with previously reported values.^40^

### 3-(4-(Methylsulfonyl)phenyl)prop-2-yn-1-ol (3F)

Synthesized according to **GP**1 using 1F (0.60 g, 2.12 mmol) and purified by flash column chromatography (20% EtOAc/Hex, *R*_f_ = 0.37, dry loading using celite) to give an off-white solid (0.35 g, 80%). ^**1**^**H NMR** (300 MHz, CDCl_3_) *δ* 7.90 (d, *J* = 8.6 Hz, 2H), 7.61 (d, *J* = 8.6 Hz, 2H), 4.53 (s, 2H), 3.06 (s, 3H), 1.76 (br s, 1H). ^**13**^**C NMR** (75 MHz, CDCl_3_) *δ* 44.6, 51.5, 83.9, 91.9, 127.5, 128.8, 132.6, 140.0. Characterization data are consistent with previously reported values.^41^

### 3-(4-((Trifluoromethyl)sulfonyl)phenyl)prop-2-yn-1-ol (3G)

Synthesized according to **GP**1 using 1G (0.50 g, 1.73 mmol) and purified by flash column chromatography (5% EtOAc/DCM, *R*_f_ = 0.47, dry loading using celite) to give a brown oil (0.34 g, 73%). ^**1**^**H NMR** (600 MHz, CDCl_3_) *δ* 8.00 (d, *J* = 8.6 Hz, 2H), 7.70 (d, *J* = 8.6 Hz, 2H), 4.57 (s, 2H), 1.98 (br s, 1H). ^**13**^**C NMR** (150 MHz, CDCl_3_) *δ* 132.7, 131.5, 130.7, 130.4, 119.7 (q, *J*_C,F_ = 324.3 Hz), 93.7, 83.5, 51.5. ^**19**^**F NMR** (282 MHz, CDCl_3_) *δ* −78.25. **HRMS (EI)** calc'd for C_10_H_6_F_3_O_3_S [M − H]˙^+^: 262.9990, found: 262.9950 (note: 5 mmu (15 ppm) discrepancy).

### 4-(3-Hydroxyprop-1-yn-1-yl)-*N*,*N*-dimethylbenzenesulfonamide (3H)

Synthesized according to **GP**1 using 4-bromo-*N*,*N*-dimethylbenzenesulfonamide (1H) (1.00 g, 3.79 mmol) and purified by flash column chromatography (70% EtOAc/Hex, *R*_f_ = 0.41, dry loading using celite) to give a pale-yellow solid (0.77 g, 84%). ^**1**^**H NMR** (600 MHz, CDCl_3_) *δ* 7.72 (d, *J* = 8.6 Hz, 2H), 7.57 (d, *J* = 8.6 Hz, 2H), 2.71 (s, 6H). ^**13**^**C NMR** (150 MHz, CDCl_3_) *δ* 135.3, 132.2, 127.8, 127.5, 91.0, 84.2, 51.7, 38.0. **HRMS (EI)** calc'd for C_11_H_13_NO_3_S [M˙]^+^: 239.0616, found: 239.0636.

### General procedure 2 (**GP**2): DMP oxidation^23^

To a solution of alcohol (3A–H and 5A–H) (1.0 eq.) in DCM (∼0.15 M) at 0 °C was added DMP (1.2 eq.) portion wise. The ice bath was then removed, and the reaction was stirred at R.T. for 3 h. The reaction was concentrated *in vacuo* and diluted with EtOAc (100 mL). Sat. Na_2_S_2_O_3_ and NaHCO_3_ (150 mL : 150 mL) were added to the reaction mixture and the solution was stirred vigorously until the precipitate dissolved. The layers were separated, and the aqueous layer was extracted with EtOAc (2 × 100 mL). The organic layer was washed with sat. NaHCO_3_ (100 mL), brine (200 mL), and then dried over MgSO_4_. After concentrating *in vacuo*, the residue was purified by flash column chromatography or carried forward without further purification as described below.

### 3-(4-Nitrophenyl)propiolaldehyde (4A)

Synthesized according to **GP**2 using 3A (0.74 g, 4.15 mmol) and purified by flash column chromatography (30% EtOAc/Hexanes, *R*_f_ = 0.57, dry loading using celite) to give a yellow solid (0.67 g, 92%). ^**1**^**H NMR** (300 MHz, CDCl_3_) *δ* 9.46 (s, 1H), 8.28 (d, *J* = 9.0 Hz, 2H), 7.78 (d, *J* = 9.0 Hz, 2H). ^**13**^**C NMR** (75 MHz, CDCl_3_) *δ* 176.1, 184.8, 133.9, 126.0, 123.9, 90.8, 90.6. Characterization data are consistent with previously reported values.^23^

### 3-Phenylpropiolaldehyde (4B)

Synthesized according to **GP**2 using 3B (0.63 g, 4.70 mmol) and the resulting solid was carried forward without further purification (5% EtOAc/DCM, *R*_f_ = 0.71) (0.60 g, >95%). ^**1**^**H NMR** (400 MHz, CDCl_3_) *δ* 9.44 (s, 1H), 7.64–7.61 (m, 2H), 7.53–7.48 (m, 1H), 7.44–7.40 (m, 2H). ^**13**^**C NMR** (100 MHz, CDCl_3_) *δ* 176.8, 133.3, 131.3, 128.7, 119.4, 95.1, 88.4. Characterization data are consistent with previously reported values.^23^

### 3-(Perfluorophenyl)propiolaldehyde (4C)

Synthesized according to modified procedure **GP**2 using 3C2 (0.30 g, 1.40 mmol). NaHCO_3_ (0.47 g, 4.60 mmol, 4.0 eq.) was added to the reaction to neutralize the AcOH in the reaction mixture. The resulting white powder (0.28 g, >95%) was carried forward without further purification (10% EtOAc/Hex, *R*_f_ = 0.41). ^**1**^**H NMR** (300 MHz, CDCl_3_) *δ* 9.41 (s, 1H). ^**13**^**C NMR** (75 MHz, CDCl_3_) *δ* 175.2, 149.9–149.7 (m), 146.5–146.3 (m), 145.6–145.2 (m), 142.1–141.7 (m), 139.4–139.2 (m), 136.3–136.0 (m), 97.3–97.7 (m, 2C overlapping), 60.7. ^**19**^**F NMR** (282 MHz, CDCl_3_) *δ* −132.68–−132.82 (m, 2F), −146.49–−146.65 (m, 1F), −160.17–−160.41 (m, 2F). **HRMS (EI)** calc'd for C_9_HF_5_O [M˙]^+^: 219.9948, found: 219.9937.

### 3-(4-Acetylphenyl)propiolaldehyde (4D)

Synthesized according to **GP**2 using 3D (0.65 g, 3.73 mmol) and purified by flash column chromatography (10% EtOAc/DCM, *R*_f_ = 0.67, dry loading using celite) to give a yellow solid (0.60 g, 93%). ^**1**^**H NMR** (300 MHz, CDCl_3_) *δ* 9.46 (s, 1H), 7.99 (d, *J* = 8.7 Hz, 2H), 7.71 (d, *J* = 8.7 Hz, 2H), 2.64 (s, 3H). ^**13**^**C NMR** (75 MHz, CDCl_3_) *δ* 196.9, 176.4, 138.5, 133.3, 128.4, 123.9, 92.9, 89.8, 26.7. Characterization data are consistent with previously reported values.^23^

### 4-(3-Oxoprop-1-yn-1-yl)benzonitrile (4E)

Synthesized according to **GP**2 using 3E (0.60 g, 3.75 mmol) and the resulting yellow solid (0.59 g, >95%) was carried forward without further purification (50% EtOAc/Hex, *R*_f_ = 0.56). ^**1**^**H NMR** (300 MHz, CDCl_3_) *δ* 9.43 (s, 1H), 7.71–7.66 (m, 4H). ^**13**^**C NMR** (75 MHz, CDCl_3_) *δ* 176.2, 133.5, 132.4, 124.2, 117.8, 114.6, 91.3, 90.3. Characterization data are consistent with previously reported values.^42^

### 3-(4-(Methylsulfonyl)phenyl)propiolaldehyde (4F)

Synthesized according to **GP**2 using 3F (0.35 g, 1.68 mmol) and purified by flash column chromatography (5% EtOAc/DCM, *R*_f_ = 0.55, dry loading using celite) to give an off-white solid (0.31 g, 91%). ^**1**^**H NMR** (300 MHz, CDCl_3_) *δ* 9.46 (s, 1H), 8.00 (d, *J* = 8.7 Hz, 2H), 7.79 (d, *J* = 8.7 Hz, 2H), 3.08 (3H, s) ^**13**^**C NMR** (75 MHz, CDCl_3_) *δ* 176.2, 142.5, 133.8, 127.7, 125.1, 91.2, 89.2, 44.4. **HRMS (EI)** calc'd for C_10_H_8_O_3_S [M˙]^+^: 208.0194, found: 208.0181.

### 3-(4-((Trifluoromethyl)sulfonyl)phenyl)propiolaldehyde (4G)

Synthesized according to **GP**2 using 3G (0.30 g, 0.76 mmol) and the resulting orange oil (0.30 g, >95%) was carried forward without further purification (5% EtOAc/DCM = 0.91). ^**1**^**H NMR** (300 MHz, CDCl_3_) *δ* 9.47 (s, 1H), 8.09 (d, *J* = 8.3 Hz, 2H), 7.87 (d, *J* = 8.3 Hz, 2H). ^**13**^**C NMR** (600 MHz, CDCl_3_) *δ* 175.9, 133.9, 133.2, 130.9, 128.1, 119.7 (q, *J*_C,F_ = 324.3 Hz), 91.0, 89.9. ^**19**^**F NMR** (282 MHz, CDCl_3_) *δ* –77.89. **HRMS (EI)** calc'd for C_10_H_5_F_3_O_3_S [M˙]^+^: 261.9911, found: 261.9912.

### *N*,*N*-Dimethyl-4-(3-oxoprop-1-yn-1-yl)benzenesulfonamide (4H)

Synthesized according to **GP**2 using 3H (0.75 g, 3.12 mmol) and the resulting yellow solid (0.73 g, >95%) was carried forward without further purification (70% EtOAc/Hex, *R*_f_ = 0.67). ^**1**^**H NMR** (600 MHz, CDCl_3_) *δ* 9.47 (s, 1H), 7.84 (d, *J* = 8.0 Hz, 2H), 7.78 (d, *J* = 8.0 Hz, 2H), 2.77 (s, 6H). ^**13**^**C NMR** (150 MHz, CDCl_3_) *δ* 176.3, 138.0, 133.5, 127.9, 123.9, 91.8, 89.8, 37.9. **HRMS (EI)** calc'd for C_11_H_11_NO_3_S [M˙]^+^: 237.0460, found: 237.0473.

### General procedure 3 (**GP**3): Grignard reaction with ethynyl magnesium bromide^24^

In a flamed dried flask under N_2_, 4A–H (1.0 eq.) was dissolved in anhydrous THF (∼0.1 M) and the solution was cooled to −78 °C. Ethynylmagnesium bromide (2.0 eq.) was then added dropwise. The reaction was stirred at this temperature for 3 h and then quenched with H_2_O (10 mL) and sat. NH_4_Cl (5 mL), and then extracted with EtOAc (3 × 50 mL). The organic layer was dried over MgSO_4_ and concentrated *in vacuo*. The resulting solid was purified by flash column chromatography as described below.

### 1-(4-Nitrophenyl)penta-1,4-diyn-3-ol (5A)

Synthesized according to **GP**3 using 4A (0.20 g, 1.14 mmol) and purified by flash column chromatography (30% EtOAc/hexanes, *R*_f_ = 0.27, dry loading using celite) to give a yellow solid (0.12 g, 54%). ^**1**^**H NMR** (300 MHz, CDCl_3_) *δ* 8.21 (d, *J* = 8.9 Hz, 2H), 7.62 (d, *J* = 8.9 Hz, 2H), 5.38 (dd, *J* = 7.5, 2.4 Hz, 1H), 2.67 (d, *J* = 2.4 Hz, 1H), 2.42 (br d, *J* = 7.5 Hz, 1H). ^**13**^**C NMR** (75 MHz, CDCl_3_) *δ* 147.6, 132.7, 128.5, 123.6, 90.3, 82.5, 80.0, 73.6, 52.4. Characterization data are consistent with previously reported values.^24^

### 1-Phenylpenta-1,4-diyn-3-ol (5B)

Synthesized according to **GP**3 using 4B (0.24 g, 1.84 mmol) and purified by flash column chromatography (30% EtOAc/Hexanes, *R*_f_ = 0.51, dry loading using celite) to give a yellow oil (0.25 g, 87%). ^**1**^**H NMR** (400 MHz, CDCl_3_) *δ* 7.50–7.47 (m, 2H), 7.38–7.31 (m, 3H), 5.37 (d, *J* = 2.4 Hz, 1H), 2.63 (d, *J* = 2.4 Hz, 1H), 2.44 (br s, 1H). ^**13**^**C NMR** (100 MHz, CDCl_3_) *δ* 131.8, 128.9, 128.3, 121.7, 85.4, 84.7, 80.8, 72.9, 52.5. Characterization data are consistent with previously reported values.^23^

### 1-(Perfluorophenyl)penta-1,4-diyn-3-ol (5C)

Synthesized according to **GP**3 using 4C (0.20 g, 0.90 mmol) and purified by flash column chromatography (10% EtOAc/hexanes, *R*_f_ = 0.21, dry loading using celite) to give a pale-yellow powder (0.12 g, 56%). ^**1**^**H NMR** (600 MHz, CDCl_3_) *δ* 5.40 (dd, *J* = 8.0, 2.3 Hz, 1H), 2.66 (d, *J* = 2.3 Hz, 1H), 2.53 (d, *J* = 8.0 Hz, 1H).^**13**^**C NMR** (125 MHz, CDCl_3_) *δ* 148.6–148.5 (m), 146.9–146.8 (m), 143.2–142.9 (m), 141.5–141.3 (m), 138.7–138.5 (m), 137.0–136.8 (m), 99.01–98.8 (m), 97.8, 79.6, 69.0, 52.6. ^**19**^**F NMR** (282 MHz, CDCl_3_) *δ* −135.15–−135.29 (m, 2F), −150.69–−150.86 (m, 1F), −161.12–−161.32 (m, 2F). **HRMS (EI)** calc'd for C_11_H_3_F_5_O [M˙]^+^: 246.0104, found: 246.0111.

### 1-(4-(3-Hydroxypenta-1,4-diyn-1-yl)phenyl)ethan-1-one (5D)

Synthesized according to **GP**3 using 4D (0.20 g, 1.16 mmol) and purified by flash column chromatography (30% EtOAc/hexanes, *R*_f_ = 0.27, dry loading using celite) to give a yellow oil (0.20 g, 86%). ^**1**^**H NMR** (300 MHz, CDCl_3_) *δ* 7.92 (d, *J* = 8.5 Hz, 2H), 7.53 (d, *J* = 8.5 Hz, 2H), 4.54 (s, 2H), 2.61 (s, 3H). ^**13**^**C NMR** (75 MHz, CDCl_3_) *δ* 197.3, 136.5, 131.8, 128.2, 127.4, 90.4, 84.9, 51.6, 26.6. Characterization data are consistent with previously reported values.^23^

### 4-(3-Hydroxypenta-1,4-diyn-1-yl)benzonitrile (5E)

Synthesized according to **GP**3 using 4E (0.20 g, 1.30 mmol) and purified by flash column chromatography (30% EtOAc/hexanes, *R*_f_ = 0.31, dry loading using celite) to give a yellow solid (0.14 g, 61%). ^**1**^**H NMR** (300 MHz, CDCl_3_) *δ* 1H NMR (300 MHz, CDCl3) *δ* 7.62 (d, *J* = 8.8 Hz, 2H), 7.55 (d, *J* = 8.8 Hz, 2H), 5.37 (dd, *J* = 7.3, 2.3 Hz, 1H), 2.65 (d, *J* = 2.3 Hz, 1H), 2.44 (br d, *J* = 7.3 Hz, 1H). ^**13**^**C NMR** (75 MHz, CDCl_3_) *δ* 132.3, 132.0, 126.6, 118.2, 112.4, 89.5, 82.8, 80.1, 73.5, 52.4. **HRMS (EI)** calc'd for C_12_H_7_NO [M˙]^+^: 181.0528, found: 181.0516.

### 1-(4-(Methylsulfonyl)phenyl)penta-1,4-diyn-3-ol (5F)

Synthesized according to **GP**3 using 4F (0.20 g, 0.96 mmol) and purified by flash column chromatography (15% EtOAc/DCM, *R*_f_ = 0.41, dry loading using celite) to give a yellow solid (0.15 g, 66%). ^**1**^**H NMR** (300 MHz, CDCl_3_) *δ* 7.89 (d, *J* = 8.7 Hz, 2H), 7.62 (d, *J* = 8.7 Hz, 2H), 5.37 (d, *J* = 2.3 Hz, 1H), 3.06 (s, 3H), 2.65 (d, *J* = 2.3 Hz, 1H). ^**13**^**C NMR** (75 MHz, CDCl_3_) *δ* 140.3, 132.6, 127.6, 127.4, 89.4, 82.6, 80.2, 73.5, 52.4, 44.4. **HRMS (EI)** calc'd for C_12_H_10_O_3_S [M˙]^+^: 234.0351, found: 234.0325.

### 1-(4-((Trifluoromethyl)sulfonyl)phenyl)penta-1,4-diyn-3-ol (5G)

Synthesized according to **GP**3 using 4G (0.30 g, 1.14 mmol) and purified by flash column chromatography (8% EtOAc/DCM, *R*_f_ = 0.65, dry loading using celite) to give a dark orange oil (0.18 g, 54%). ^**1**^**H NMR** (300 MHz, CDCl_3_) *δ* 8.01 (d, *J* = 8.7 Hz, 2H), 7.73 (d, *J* = 8.7 Hz, 2H), 5.39 (d, *J* = 2.3 Hz, 1H), 2.66 (d, *J* = 2.3 Hz, 1H). ^**13**^**C NMR** (150 MHz, CDCl_3_) *δ* 133.0, 131.0, 130.7, 130.6, 119.7 (q, *J*_C,F_ = 324.3 Hz), 91.4, 82.2, 79.9, 73.8, 52.4. ^**19**^**F NMR** (282 MHz, CDCl_3_) *δ* –78.16. **HRMS (EI)** calc'd for C_12_H_7_F_3_O_3_S [M˙]^+^: 288.0068, found: 288.0055.

### 4-(3-Hydroxypenta-1,4-diyn-1-yl)-*N*,*N*-dimethylbenzenesulfonamide (5H)

Synthesized according to **GP**3 using 4H (0.30 g, 1.25 mmol) and the resulting yellow solid (0.27 g, 75%) was carried forward without further purification (70% EtOAc/Hex, *R*_f_ = 0.49). ^**1**^**H NMR** (600 MHz, CDCl_3_) *δ* 7.76 (d, *J* = 8.7 Hz, 2H), 7.64 (d, *J* = 8.7 Hz, 2H), 5.39 (dd, *J* = 7.6, 2.3 Hz, 1H), 2.73 (s, 6H), 2.67 (d, *J* = 2.3 Hz, 1H), 2.50 (d, *J* = 7.6 Hz, 1H). ^**13**^**C NMR** (150 MHz, CDCl_3_) *δ* 135.5, 132.4, 127.9, 127.7, 88.9, 82.9, 80.3, 73.5, 52.5, 37.9. **HRMS (EI)** calc'd for C_13_H_13_NO_3_S [M˙]^+^: 263.0616, found: 263.0639.

### 1-(4-Nitrophenyl)penta-1,4-diyn-3-one (6A)

Synthesized according to **GP**2 using 5A (0.12 g, 0.60 mmol) and purified by flash column chromatography (30% EtOAc/hexanes, *R*_f_ = 0.50, dry loading using celite) to give a yellow solid (0.11 g, 88%). ^**1**^**H NMR** (300 MHz, CDCl_3_) *δ* 8.28 (d, *J* = 8.9 Hz, 2H), 7.79 (d, *J* = 8.9 Hz, 2H), 3.46 (s, 1H). ^**13**^**C NMR** (75 MHz, CDCl_3_) *δ* 159.4, 148.9, 134.0, 125.7, 123.8, 91.1, 88.0, 81.8, 80.3. Characterization data are consistent with previously reported values.^24^

### 1-Phenylpenta-1,4-diyn-3-one (6B)

Synthesized according to **GP**2 using 5B (0.25 g, 1.60 mmol) and the resulting solid (0.24 g, >95%) was carried forward without further purification (30% EtOAc/hexanes, *R*_f_ = 0.77). ^**1**^**H NMR** (400 MHz, CDCl_3_) *δ* 7.65–7.63 (m, 2H), 7.54–7.49 (m, 1H), 3.80 (s, 1H). ^**13**^**C NMR** (75 MHz, CDCl_3_) *δ* 160.1, 133.5, 131.5, 128.7, 119.1, 92.5, 89.0, 82.2, 79.0. Characterization data are consistent with previously reported values.^23^

### 1-(Perfluorophenyl)penta-1,4-diyn-3-one (6C)

Synthesized according to modified **GP**2 using 5C (0.12 g, 0.49 mmol) and the resulting white powder (0.11 g, >95%) was carried forward without further purification (10% EtOAc/Hex, *R*_f_ = 0.53). ^**1**^**H NMR** (600 MHz, CDCl_3_) *δ* 3.49 (s, 1H). ^**13**^**C NMR** (150 MHz, CDCl_3_) *δ* 158.7, 149.2–149.1 (m), 147.5–147.4 (m), 144.9–144.6 (m), 143.1–142.9 (m), 138.7–138.5 (m), 137.0–136.8 (m), 97.6, 97.0–96.7 (m), 81.5, 81.1, 74.5. ^**19**^**F NMR** (282 MHz, CDCl_3_) *δ* −131.87–−132.00 (m, 2F), −145.45–−145.62 (m, 1F), −159.49–−159.76 (m, 2F). **HRMS (EI)** calc'd for C_11_HF_5_O [M˙]^+^: 243.9948, found: 243.9922.

### 1-(4-Acetylphenyl)penta-1,4-diyn-3-one (6D)

Synthesized according to **GP**2 using 5D (0.20 g, 1.0 mmol) and purified by flash column chromatography (30% EtOAc/hexanes, *R*_f_ = 0.43, dry loading using celite) to give a grey solid (0.18 g, 92%). ^**1**^**H NMR** (300 MHz, CDCl_3_) *δ* 7.99 (d, *J* = 8.7 Hz, 2H), 7.73 (d, *J* = 8.7 Hz, 2H), 3.43 (s, 1H), 2.64 (s, 3H). ^**13**^**C NMR** (75 MHz, CDCl_3_) *δ* 196.9, 159.8, 138.6, 133.5, 128.4, 123.6, 90.4, 90.2, 82.0, 79.7, 26.7. Characterization data are consistent with previously reported values.^23^

### 4-(3-Oxopenta-1,4-diyn-1-yl)benzonitrile (6E)

Synthesized according to **GP**2 using 5E (0.14 g, 0.77 mmol) and purified by flash column chromatography (30% EtOAc/Hexanes, R_f_ = 0.48, dry loading using celite) to give a yellow solid (0.12 g, 88%). ^**1**^**H NMR** (300 MHz, CDCl_3_) *δ* 7.71 (s, 4H), 3.44 (s, 1H). ^**13**^**C NMR** (75 MHz, CDCl_3_) *δ* 159.7, 133.7, 132.5, 124.0, 117.8, 114.9, 90.9, 88.7, 81.9, 80.4. **HRMS (EI)** calc'd for C_12_H_5_NO [M˙]^+^: 179.0371, found: 179.0362.

### 1-(4-(Methylsulfonyl)phenyl)penta-1,4-diyn-3-one (6F)

Synthesized according to **GP**2 using 5F (0.14 g, 0.60 mmol) and the resulting yellow solid (0.14 g, >95%) was used without further purification (15% EtOAc/DCM, *R*_f_ = 0.69). ^**1**^**H NMR** (600 MHz, CDCl_3_) *δ* 8.00 (d, *J* = 8.6 Hz, 2H), 7.81 (d, *J* = 8.7 Hz, 2H), 3.45 (s, 1H), 3.06 (s, 3H). ^**13**^**C NMR** (150 MHz, CDCl_3_) *δ* 159.7, 142.7, 134.1, 127.8, 124.9, 90.5, 88.6, 82.0, 80.3, 77.4, 77.2, 76.9, 44.5. **HRMS (EI)** calc'd for C_12_H_8_O_3_S [M˙]^+^: 232.0194, found: 232.0187.

### 1-(4-((Trifluoromethyl)sulfonyl)phenyl)penta-1,4-diyn-3-one (6G)

Synthesized according to **GP**2 using 5G (0.18 g, 0.62 mmol) the resulting oil was triturated with hexanes to give a beige solid (0.14 g, 80%) which was used without further purification (30% EtOAc/DCM, *R*_f_ = 0.62). ^**1**^**H NMR** (300 MHz, CDCl_3_) *δ* 8.09 (d, *J* = 8.3 Hz, 2H), 7.89 (d, *J* = 8.3 Hz, 2H), 3.49 (s, 1H). ^**13**^**C NMR** (150 MHz, CDCl_3_) *δ* 159.3, 134.0, 133.3, 130.9, 127.8, 119.7 (q, *J*_C,F_ = 324.3 Hz), 91.4, 87.2, 81.7, 80.7. ^**19**^**F NMR** (282 MHz, CDCl_3_) *δ* –77.88. **HRMS (EI)** calc'd for C_12_H_5_F_3_O_3_S [M˙]^+^: 285.9911, found: 284.9909.

### *N*,*N*-Dimethyl-4-(3-oxopenta-1,4-diyn-1-yl)benzenesulfonamide (6H)

Synthesized according to **GP**2 using 5H (0.27 g, 1.03 mmol, 1.0 eq.) and the resulting yellow solid (0.26 g, >95%) was used without further purification (70% EtOAc/Hex, *R*_f_ = 0.75). ^**1**^**H NMR** (600 MHz, CDCl_3_) *δ* 7.81–7.77 (m, 4H), 3.45 (s, 1H), 2.74 (s, 6H). ^**13**^**C NMR** (150 MHz, CDCl_3_) *δ* 159.7, 138.2, 133.7, 127.9, 123.6, 90.3, 89.1, 81.9, 80.1, 37.9. **HRMS (EI)** calc'd for C_13_H_11_NO_3_S [M˙]^+^: 261.0460, found: 261.0442.

### General procedure 4: synthesis of azides

To a stirring solution of alkyl bromides or chlorides (1.0 eq., commercially available unless indicated otherwise) in DMF (∼0.5 M) was added NaN_3_ (2.0 eq.). The reaction was stirred at 60 °C for 16 h, after which it was concentrated *in vacuo*, diluted with water (20 mL), and then extracted with EtOAc (3 × 20 mL). The combined organic layer was washed with brine (25 mL), dried over MgSO_4_, and concentrated *in vacuo* to give azides (7A, E–J) which were used without further purification.

### 1-(Azidomethyl)-4-nitrobenzene (7A)

Synthesized according to **GP**4 using 4-nitrobenzyl bromide (2.16 g, 10.00 mmol) to give a yellow oil (1.43 g, 80%). ^**1**^**H NMR** (300 MHz, CDCl_3_) *δ* 8.24 (d, *J* = 6.6 Hz, 2H), 7.50 (d, *J* = 6.6 Hz, 2H), 4.50 (s, 2H). ^**13**^**C NMR** (75 MHz, CDCl_3_) *δ* 142.4, 137.8, 124.8, 120.6, 55.8. Characterization data are consistent with previously reported values.^24^

### (4-(Dimethylamino)phenyl)methanol (7B1)

To a solution of 4-(dimethylamino)benzaldehyde (0.50 g, 3.35 mmol, 1.0 eq.) in EtOH (20 mL) at R.T. was added NaBH_4_ (0.14 g, 3.69 mmol, 1.1 eq.) portion wise. The reaction was stirred at R.T. for 5 h, concentrated *in vacuo* and then quenched with H_2_O (25 mL). The aqueous phase was extracted with EtOAc (3 × 25 mL) and the combined organic layer was washed with brine (50 mL), dried over MgSO_4_ and concentrated *in vacuo* to give a clear oil (0.48 g, 95%). ^**1**^**H NMR** (300 MHz, CDCl_3_) *δ* 7.24 (d, *J* = 8.7 Hz, 2H), 6.73 (d, *J* = 8.7 Hz, 2H), 4.56 (s, 2H), 2.95 (s, 6H). Characterization data are consistent with previously reported values.^43^

### 4-(Azidomethyl)-*N*,*N*-dimethylaniline (7B2)

To a solution of 7B1 (0.43 g, 2.81 mmol, 1.0 eq.) in anhydrous THF (15 mL) at 0 °C were added diphenylphosphoryl azide (0.91 mL, 4.22 mmol, 1.5 eq.) and DBU (0.63 mL, 4.22 mmol, 1.5 eq.) dropwise. The reaction was warmed to R.T. and stirred for 16 h. The reaction mixture was quenched with 1 M HCl (30 mL) and extracted with EtOAc (25 mL). The aqueous layer was then basified with 1 M NaOH to pH ≈ 8 and extracted with EtOAc (3 × 15 mL). The combined organic layer was washed with brine (50 mL), dried over MgSO_4_ and concentrated *in vacuo* to give a yellow oil which was purified by flash column chromatography (10% EtOAc/hexanes, *R*_f_ = 0.52, dry loading using celite) to give a clear colourless oil (0.23 g, 46%). ^**1**^**H NMR** (300 MHz, CDCl_3_) *δ* 7.19 (d, *J* = 8.7 Hz, 2H), 6.72 (d, *J* = 8.7 Hz, 2H), 4.23 (s, 2H), 2.97 (s, 6H). ^**13**^**C NMR** (75 MHz, CDCl_3_) *δ* 150.5, 129.6, 122.7, 112.4, 54.7, 40.5. Characterization data are consistent with previously reported values.^44^

### (Adamantan-1-yl)methyl methanesulfonate (7C1)

To a solution of 1-adamantanemethanol (1.00 g, 6.00 mmol, 1.0 eq.) in DCM (25 mL) was added NEt_3_ (2.10 mL, 15.00 mmol, 2.5 eq.) and the mixture was cooled to 0 °C. Then methanesulfonyl chloride (0.60 mL, 7.20 mmol, 1.2 eq.) was added dropwise and the reaction mixture was allowed to warm to R.T. and stirred for 4 h. The reaction was quenched with sat. NaHCO_3_ (25 mL) and extracted with EtOAc (3 × 30 mL). The combined organic layer was washed with sat. NH_4_Cl (50 mL), brine (50 mL), and then dried over MgSO_4_ and concentrated *in vacuo* to give a pale-yellow solid which was used without further purification (1.43 g, >95%). ^**1**^**H NMR** (300 MHz, CDCl_3_) *δ* 3.78 (s, 2H), 2.99 (2, 3H), 2.02 (apparent s, 3H), 1.76–1.63 (m, 6H), 1.57 (m, 6H). ^**13**^**C NMR** (75 MHz, CDCl_3_) *δ* 79.5, 38.9, 37.2, 36.9, 33.6, 28.0. Characterization data are consistent with previously reported values.^45^

### 1-(Azidomethyl)adamantane (7C2)

To a solution of 7C1 in DMF (10 mL) was added NaN_3_ (1.95 g, 30.00 mmol, 10.0 eq.) and the reaction was stirred at 120 °C for 48 h. The reaction mixture was quenched with H_2_O (20 mL) and extracted with 1 : 4 EtOAc/hexanes (3 × 25 mL). The combined organic layer was washed with brine (25 mL), dried over MgSO_4_ and concentrated *in vacuo* to give a pale-yellow oil which was used without further purification (0.47 g, 82%). ^**1**^**H NMR** (300 MHz, CDCl_3_) *δ* 2.95 (s, 2H), 2.01–1.98 (m, 3H), 1.76–1.59 (m, 6H), 1.53–1.52 (m, 6H). ^**13**^**C NMR** (75 MHz, CDCl_3_) *δ* 64.3, 40.0, 36.8, 34.8, 28.2. Characterization data are consistent with previously reported values.^45^

### 4-(Azidomethyl)tetrahydro-2*H*-pyran (7D)

To a solution of tetrahydropyran-4-methanol (0.35 mL, 3.00 mmol, 1.0 eq.) in DCM (15 mL) was added NEt_3_ (1.00 mL, 7.50 mmol, 2.5 eq.) and the mixture was cooled to 0 °C. Then methanesulfonyl chloride (0.30 mL, 3.60 mmol, 1.2 eq.) was added dropwise and the reaction mixture was allowed to warm to R.T. and stirred for 4 h. The reaction was quenched with sat. NaHCO_3_ (15 mL) and extracted with DCM (2 × 10 mL). The combined organic layer was washed brine (25 mL) and then dried over MgSO_4_ and concentrated *in vacuo* to give a pale-yellow oil. The oil was dissolved in DMF (10 mL) and to this solution was added NaN_3_ (0.98 g, 15.00 mmol, 5.0 eq.) and the reaction was stirred at 80 °C for 16 h. The reaction mixture was quenched with H_2_O (20 mL) and extracted with Et_2_O (3 × 10 mL). The combined organic layer was washed with brine (25 mL), dried over MgSO_4_ and concentrated *in vacuo* to give a pale-yellow oil which was used without further purification (0.30 g, 72%). ^**1**^**H NMR** (300 MHz, CDCl_3_) *δ* 4.00–3.94 (m, 2H), 3.37 (td, *J* = 11.7, 2.1 Hz, 2H), 3.17 (d, *J* = 6.9 Hz, 2H), 1.83–1.72 (m, 1H), 1.68–1.60 (m, 2H), 1.41–1.24 (m, 2H). ^**13**^**C NMR** (75 MHz, CDCl_3_) *δ* 67.6, 57.3, 35.5, 30.5. Characterization data are consistent with previously reported values.^46^

### 1-(Azidomethyl)-4-(trifluoromethyl)benzene (7E)

Synthesized according to **GP**4 using 4-trifluoromethylbenzyl bromide (0.50 g, 2.10 mmol) give a clear colourless liquid (0.27 g, 65%). ^**1**^**H NMR** (300 MHz, CDCl_3_) *δ* 7.66 (d, *J* = 7.8 Hz, 2H), 7.45 (d, *J* = 7.8 Hz, 2H), 4.43 (s, 2H). ^**13**^**C NMR** (75 MHz, CDCl_3_) *δ* 139.4, 130.5 (q, *J*_CF_ = 32.3 Hz), 128.3, 125.8 (q, *J*_CF_ = 1.8 Hz), 124.0 (q, *J*_CF_ = 270.3 Hz), 54.1. ^**19**^**F NMR** (283 MHz, CDCl_3_) *δ* −62.6. Characterization data are consistent with previously reported values.^47^

### 1-Azidohexane (7F)

Synthesized according to **GP**4 using 1-bromohexane (0.42 mL, 3.00 mmol) to give a clear colourless liquid (0.32 g, 84%). ^**1**^**H NMR** (300 MHz, CDCl_3_) *δ* 3.25 (t, *J* = 6.9 Hz, 2H), 1.66–1.51 (m, 2H), 1.45–1.20 (m, 6H), 0.89 (t, *J* = 6.9 Hz, 3H). ^**13**^**C NMR** (75 MHz, CDCl_3_) *δ* 51.6, 31.5, 28.9, 26.5, 22.6, 14.1. Characterization data are consistent with previously reported values.^48^

### 4-(Azidomethyl)benzonitrile (7G)

Synthesized according to **GP**4 using 4-cyanobenzyl bromide (0.50 g, 2.50 mmol) to give a clear colourless liquid (0.33 g, 83%). ^**1**^**H NMR** (300 MHz, CDCl_3_) *δ* 7.66 (d, *J* = 8.0 Hz, 2H), 7.43 (d, *J* = 8.0 Hz, 2H), 4.47 (s, 2H). ^**13**^**C NMR** (75 MHz, CDCl_3_) *δ* 140.8, 132.6, 128.5, 118.4, 112.1, 53.7. Characterization data are consistent with previously reported values.^49^

### 4-(2-Azidoethyl)morpholine (7H)

Synthesized according to **GP**4 using 4-(2-Chloroethyl)morpholine (0.18 mL, 1.30 mmol) to give a colourless oil (0.20 g, 96%). ^**1**^**H NMR** (400 MHz, CDCl_3_) 3.72 (t, *J* = 4.4 Hz, 4H), 3.35 (t, *J* = 6.0 Hz, 2H), 2.59 (t, *J* = 6.0 Hz, 2H), 2.59 (t, *J* = 4.4 Hz, 4H). *δ*^**13**^**C NMR** (100 MHz, CDCl_3_) *δ* 67.0, 57.7, 53.7, 48.0. Characterization data are consistent with previously reported values.^50^

### *tert*-Butyl 4-(azidomethyl)piperidine-1-carboxylate (7I)

Synthesized according to **GP**4 using *tert*-butyl 4-(bromomethyl)piperidine-1-carboxylate (0.50 g, 1.80 mmol) to give a colourless oil (0.41 g, 94%). ^**1**^**H NMR** (400 MHz, CDCl_3_) 4.11 (d, *J* = 12.8 Hz, 2H), 3.17 (d, *J* = 6.0 Hz, 2H), 2.67 (td, *J* = 13.2, 2.8 Hz, 2H), 1.73–1.63 (m, 3H), 1.44 (s, 9H), 1.20–1.09 (m, 2H). *δ*^**13**^**C NMR** (100 MHz, CDCl_3_) *δ* 154.9, 79.6, 57.1, 43.6, 36.4, 29.7, 28.6. Characterization data are consistent with previously reported values.^51^

### 5-(Azidomethyl)picolinonitrile (7J)

Synthesized according to **GP**4 using 2-cyano-5-bromomethylpyridine (0.40 g, 2.00 mmol) to give a brown solid (0.26 g, 82%). ^**1**^**H NMR** (600 MHz, CDCl_3_) *δ* 8.70 (s, 1H), 7.84 (dd, *J* = 8.3, 1.8 Hz, 1H), 7.75 (d, *J* = 8.0 Hz, 1H), 4.55 (s, 2H). ^**13**^**C NMR** (150 MHz, CDCl_3_) 150.3, 136.2, 135.3, 133.6, 128.4, 116.9, 51.5. Characterization data are consistent with previously reported values.^52^

### General procedure 4 (**GP**5): CuAAC reaction with CuI and DIPEA^23^

To a solution of 6A–H (1.0 eq.), 7A–J (1.0 eq.), and CuI (1.0 eq.) in anhydrous MeCN (∼0.1 M relative to 6) under N_2_ at R.T. was added DIPEA (1.0 eq.) dropwise and the reaction was stirred for 16 h. The reaction mixture was then diluted with DCM (20 mL) and washed with 1 M HCl (2 × 15 mL), brine (20 mL), sat. NH_4_OH (4 × 15 mL, until aqueous phase was no longer blue), and brine (20 mL) again. The organic layer was then dried over MgSO_4_ and concentrated *in vacuo* to give a residue which was purified by flash column chromatography as described below.

### 1-(1-(4-Nitrobenzyl)-1*H*-1,2,3-triazol-4-yl)-3-(4-nitrophenyl)prop-2-yn-1-one (8A)

Synthesized according to **GP**5 using 6A (0.10 g, 0.50 mmol) and 7A (0.09 g, 0.50 mmol) and purified by flash column chromatography (1–5% EtOAc/DCM, in 2% EtOAc/DCM *R*_f_ = 0.23, dry loading using celite) to give a yellow solid (0.10 g, 54%). ^**1**^**H NMR** (300 MHz, DMSO-*d*_6_) *δ* 9.29 (s, 1H), 8.36 (d, *J* = 9.0 Hz, 2H), 8.26 (d, *J* = 8.7 Hz, 2H), 8.04 (d, *J* = 9.0 Hz, 2H), 7.59 (d, *J* = 8.7 Hz, 2H), 5.91 (s, 2H). ^**13**^**C NMR** (75 MHz, DMSO-*d*_6_) *δ* 168.2, 148.6, 147.4, 146.9, 142.6, 134.3, 130.9, 129.2, 125.4, 124.1, 124.0, 89.5, 88.7, 52.4. **HRMS (EI)** calc'd for C_18_H_11_N_5_O_5_ [M˙]^+^: 377.0760, found: 377.0750. Characterization data are consistent with previously reported values.^23^

### 1-(1-(4-(Dimethylamino)benzyl)-1*H*-1,2,3-triazol-4-yl)-3-(4-nitrophenyl)prop-2-yn-1-one (8B)

Synthesized according to **GP**5 using 6A (0.09 g, 0.45 mmol) and 7B (0.08 g, 0.45 mmol) and purified by flash column chromatography (1–5% EtOAc/DCM, in 2% EtOAc/DCM *R*_f_ = 0.27, dry loading using celite) to give an orange solid (0.12 g, 71%). ^**1**^**H NMR** (300 MHz, CDCl_3_) *δ* 8.27 (d, *J* = 9.0 Hz, 2H), 8.00 (s, 1H), 7.86 (d, *J* = 9.0 Hz, 2H), 7.21 (d, *J* = 8.7 Hz, 2H), 6.71 (d, *J* = 8.7 Hz, 2H), 5.91 (s, 2H), 2.98 (s, 6H). ^**13**^**C NMR** (75 MHz, CDCl_3_) *δ* 170.0, 161.2, 148.7, 148.0, 134.1, 130.0, 126.8, 126.7, 123.9, 120.1, 112.7, 90.1, 89.9, 54.7, 40.5. **HRMS (EI)** calc'd for C_20_H_17_N_5_O_3_ [M˙]^+^: 377.1331, found: 375.1324.

### 1-(1-((Adamantan-1-yl)methyl)-1*H*-1,2,3-triazol-4-yl)-3-(4-nitrophenyl)prop-2-yn-1-one (8C)

Synthesized according to **GP**5 using 6A (0.08 g, 0.40 mmol) and 7C (0.08 g, 0.40 mmol) and purified by flash column chromatography (2% EtOAc/DCM, *R*_f_ = 0.43, dry loading using celite) to give a pale-yellow solid (0.10 g, 67%). ^**1**^**H NMR** (300 MHz, CDCl_3_) *δ* 8.28 (d, *J* = 9.0 Hz, 2H), 8.10 (s, 1H), 7.89 (d, *J* = 9.0 Hz, 2H), 4.12 (s, 2H), 2.03 (apparent s, 3H, 1.75–1.58 (m, 6H), 1.53–1.52 (m, 6H). ^**13**^**C NMR** (75 MHz, CDCl_3_) *δ* 170.1, 148.8, 147.6, 134.2, 128.0, 126.8, 123.9, 90.1 (2 alkyne C), 62.8, 40.3, 36.5, 34.4, 28.1. **HRMS (ESI)** calc'd for C_22_H_22_N_4_O_3_Na [M + Na]^+^: 413.1589, found: 413.1590.

### 3-(4-Nitrophenyl)-1-(1-((tetrahydro-2*H*-pyran-4-yl)methyl)-1*H*-1,2,3-triazol-4-yl)prop-2-yn-1-one (8D)

Synthesized according to **GP**5 using 6A (0.08 g, 0.40 mmol) and 7D (0.05 g, 0.40 mmol) and purified using preparative thin layer chromatography by eluting 3 times (1% MeOH/DCM, *R*_f_ = 0.19) to give a pale-yellow solid (0.03 g, 19%).^53^^**1**^**H NMR** (300 MHz, CDCl_3_) *δ* 8.27 (d, *J* = 9.0 Hz, 2H), 8.16 (s, 1H), 7.85 (d, *J* = 9.0 Hz, 2H), 4.33 (d, *J* = 7.2 Hz, 2H), 4.00–3.96 (m, 2H), 3.35 (td, *J* = 11.4, 2.1 Hz, 2H), 2.29–2.11 (m, 1H), 1.54–1.34 (m, 4H). ^**13**^**C NMR** (75 MHz, CDCl_3_) *δ* 169.8, 148.7, 147.8, 134.1, 127.3, 126.6, 123.8, 90.1, 89.8, 67.1, 56.3, 36.2, 30.2. **HRMS (EI)** calc'd for C_17_H_16_N_4_O_4_ [M˙]^+^: 340.1172, found: 340.1149.

### 3-(4-Nitrophenyl)-1-(1-(4-(trifluoromethyl)benzyl)-1*H*-1,2,3-triazol-4-yl)prop-2-yn-1-one (8E)

Synthesized according to **GP**5 using 6A (0.08 g, 0.40 mmol) and 7E (0.08 g, 0.40 mmol) and purified using flash chromatography (1% EtOAc/DCM, *R*_f_ = 0.21, dry loading using celite) to give a beige solid (0.11 g, 71%). ^**1**^**H NMR** (300 MHz, CDCl_3_) *δ* 8.27 (d, *J* = 9.0 Hz, 2H), 8.17 (s, 1H), 7.85 (d, *J* = 9.0 Hz, 2H), 7.67 (d, *J* = 8.1 Hz, 2H), 7.43 (d, *J* = 8.1 Hz, 2H), 5.69 (s, 2H). ^**13**^**C NMR** (75 MHz, CDCl_3_) *δ* 169.7, 148.8, 148.5, 137.2, 134.2, 131.7 (q, *J*_CF_ = 32.8 Hz), 128.6, 127.0, 126.6, 126.5 (q, *J*_CF_ = 3.7 Hz), 123.9, 123.7 (q, *J*_CF_ = 293.0 Hz), 90.4, 89.8, 54.1. ^**19**^**F NMR** (282 MHz, CDCl_3_) *δ* −62.8. **HRMS (EI)** calc'd for C_19_H_11_F_3_N_4_O_3_ [M˙]^+^: 400.0783, found: 400.0784.

### 1-(1-Hexyl-1*H*-1,2,3-triazol-4-yl)-3-(4-nitrophenyl)prop-2-yn-1-one (8F)

Synthesized according to **GP**5 using 6A (0.08 g, 0.40 mmol) and 7F (0.05 g, 0.40 mmol) and purified using flash chromatography (0 → 3% EtOAc/DCM, 3% EtOAc/DCM *R*_f_ = 0.53, dry loading using celite) to give a pale-yellow solid (0.08 g, 61%). ^**1**^**H NMR** (300 MHz, CDCl_3_) *δ* 8.31 (d, *J* = 9.0 Hz, 2H), 8.22 (s, 1H), 7.91 (d, *J* = 9.0 Hz, 2H), 4.49 (t, *J* = 7.2 Hz, 2H), 2.04–1.95 (m, 2H), 1.43–1.29 (m, 6H), 0.92 (t, *J* = 7.2 Hz, 3H). ^**13**^**C NMR** (75 MHz, CDCl_3_) *δ* 169.9, 148.6, 147.9, 134.1, 126.7, 123.8, 90.0, 89.8, 51.0, 31.1, 30.1, 26.0, 22.4, 13.9. **HRMS (EI)** calc'd for C_17_H_18_N_4_O_3_ [M˙]^+^: 326.1379, found: 326.1357.

### 4-((4-(3-(4-Nitrophenyl)propioloyl)-1*H*-1,2,3-triazol-1-yl)methyl)benzonitrile (8G)

Synthesized according to **GP**5 using 6A (0.06 g, 0.32 mmol) and 7G (0.05 g, 0.32 mmol) and purified by flash column chromatography (5% EtOAc/DCM, *R*_f_ = 0.29, dry loading using celite) to give a pale-yellow solid (0.09 g, 77%). ^**1**^**H NMR** (300 MHz, DMSO-*d*_6_) *δ* 9.24 (s, 1H), 8.31 (d, *J* = 9.0 Hz, 2H), 8.01 (d, *J* = 9.0 Hz, 2H), 7.84 (d, *J* = 8.4 Hz, 2H), 7.49 (d, *J* = 8.4 Hz, 2H), 5.82 (s, 2H). ^**13**^**C NMR** (75 MHz, DMSO-*d*_6_) *δ* 168.2, 148.6, 146.9, 140.7, 134.3, 132.8, 130.8, 128.8, 125.4, 118.5, 111.2, 89.5, 88.7, 52.7. **HRMS (ESI)** calc'd for C_38_H_22_N_10_O_6_ [2 M + Na]^+^: 737.1645, found: 737.1648.

### 1-(1-(2-Morpholinoethyl)-1*H*-1,2,3-triazol-4-yl)-3-(4-nitrophenyl)prop-2-yn-1-one (8H)

Synthesized according to **GP**5 using 6A (0.08 g, 0.40 mmol) and 7H (0.06 g, 0.40 mmol), and purified by flash column chromatography (2% MeOH/EtOAc, *R*_f_ = 0.44, dry loading using celite) to give a pale-yellow solid (0.08 g, 56%). ^**1**^**H NMR** (400 MHz, CDCl_3_) *δ* 8.38 (s, 1H), 8.27 (d, *J* = 9.0 Hz, 2H), 7.87 (d, *J* = 9.0 Hz, 2H), 4.56 (t, *J* = 6.0 Hz, 2H), 3.70 (t, *J* = 4.8 Hz, 2H), 2.87 (t, *J* = 6.0 Hz, 2H), 2.52 (t, *J* = 4.8 Hz, 4H). ^**13**^**C NMR** (100 MHz, CDCl_3_) *δ* 170.0, 148.8, 148.0, 134.1, 127.7, 126.8, 123.9, 90.1, 90.0, 66.9, 57.6, 53.5, 47.7. **HRMS (ESI)** calc'd for C_17_H_18_N_5_O_4_ [M + H]^+^: 356.1359, found: 356.1320.

### *tert*-Butyl 4-((4-(3-(4-nitrophenyl)propioloyl)-1*H*-1,2,3-triazol-1-yl)methyl)piperidine-1-carboxylate (8I)

Synthesized according to **GP**5 using 6A (0.10 g, 0.50 mmol) and 7I (0.12 g, 0.50 mmol), and purified by flash column chromatography (10–30% EtOAc/DCM, *R*_f_ (30% EtOAc/DCM) = 0.72, dry loading using celite) to give a yellow solid (0.15 g, 68%). ^**1**^**H NMR** (400 MHz, CDCl_3_) *δ* 8.26 (d, *J* = 8.8 Hz, 2H), 8.15 (s, 1H), 7.85 (d, *J* = 8.8 Hz, 2H), 4.32 (d, *J* = 7.2 Hz, 2H), 4.15–4.07 (m, 2H), 2.67 (t, *J* = 12.8 Hz, 2H), 2.18–2.06 (m, 1H), 1.63–1.56 (m, 2H), 1.43 (s, 9H), 1.27–1.17 (m, 2H). ^**13**^**C NMR** (100 MHz, CDCl_3_) *δ* 169.8, 154.6, 148.7, 147.9, 134.1, 127.3, 126.6, 123.8, 90.2, 89.8, 79.8, 56.1, 43.2, 37.3, 29.4, 28.4. **(ESI)** calc'd for C_22_H_25_N_5_O_5_Na [M + Na]^+^: 462.1753, found: 462.1755.

### 3-Phenyl-1-(1-((tetrahydro-2*H*-pyran-4-yl)methyl)-1*H*-1,2,3-triazol-4-yl)prop-2-yn-1-one (8J)

Synthesized according to **GP**5 using 6B (0.06 g, 0.39 mmol) and 7D (0.06 g, 0.39 mmol), and purified by flash column chromatography (10% EtOAc/DCM, *R*_f_ = 0.26, dry loading using celite) to give a pale-yellow solid (0.07 g, 62%). ^**1**^**H NMR** (300 MHz, CDCl_3_) *δ* 8.15 (s, 1H), 7.75–7.71 (m, 2H), 7.51–7.39 (m, 2H), 4.33 (d, *J* = 7.2 Hz, 2H), 4.02–3.97 (m, 2H), 3.37 (td, *J* = 11.3, 3.7 Hz, 2H), 2.29–2.15 (m, 1H), 1.58–1.33 (m, 4H). ^**13**^**C NMR** (75 MHz, CDCl_3_) *δ* 170.2, 148.2, 133.5, 131.1, 128.6, 127.2, 119.9, 94.2, 87.1, 77.2, 77.0, 76.8, 67.1, 56.2, 36.2, 30.2. **HRMS (EI)** calc'd for C_17_H_17_N_3_O_2_ [M˙]^+^: 295.1321, found: 295.1341.

### 3-(Perfluorophenyl)-1-(1-((tetrahydro-2*H*-pyran-4-yl)methyl)-1*H*-1,2,3-triazol-4-yl)prop-2-yn-1-one (8K)

Synthesized according to **GP**5 using 6C (0.08 g, 0.33 mmol), and 7D (0.05 g, 0.33 mmol), and purified by preparative thin layer chromatography (5% MeOH/DCM, *R*_f_ = 0.20, developed 3×) to give a pale-yellow solid (0.08 g, 63%).^53^^**1**^**H NMR** (600 MHz, CDCl_3_) *δ* 8.18 (s, 1H), 4.34 (d, *J* = 7.3 Hz, 2H), 4.03–3.97 (m, 2H), 3.37 (td, *J* = 11.7, 2. Hz, 2H), 2.31–2.15 (m, 1H), 1.57–1.35 (m, 4H). ^**13**^**C NMR** (150 MHz, CDCl_3_) *δ* 168.6, 149.1–149.0 (m), 147.5, 147.4–147.3 (m), 144.5–144.3 (m), 142.7–142.6 (m), 138.7–138.5 (m), 137.0–136.8 (m), 127.7, 97.6–97.4 (m), 96.4, 76.0, 67.1, 56.3, 36.2, 30.2. ^**19**^**F NMR** (282 MHz, CDCl_3_) *δ* −132.09–−132.23 (m, 2F), −146.59–−146.77 (m, 1F), −159.88–−160.04 (m, 2F). **HRMS (EI)** calc'd for C_17_H_12_F_5_N_3_O_2_ [M˙]^+^: 385.0850, found: 385.0839.

### 3-(4-Acetylphenyl)-1-(1-((tetrahydro-2*H*-pyran-4-yl)methyl)-1*H*-1,2,3-triazol-4-yl)prop-2-yn-1-one (8L)

Synthesized according to **GP**5 using 6D (0.08 g, 0.41 mmol) and 7D (0.06 g, 0.41 mmol), and purified by flash column chromatography (20–40% EtOAc/DCM, in 20% EtOAc/DCM *R*_f_ = 0.26, dry loading using celite) to give a pale-yellow solid (0.05 g, 36%). ^**1**^**H NMR** (400 MHz, CDCl_3_) *δ* 8.16 (s, 1H), 8.00 (d, *J* = 8.4 Hz, 2H), 7.81 (d, *J* = 8.4 Hz, 2H), 4.34 (d, *J* = 6.0 Hz, 2H), 3.41–3.33 (m, 2H), 3.37 (td, *J* = 11.7, 2.1 Hz, 2H), 2.64 (s, 3H), 2.29–2.17 (m, 1H), 1.56–1.36 (m, 4H). ^**13**^**C NMR** (100 MHz, CDCl_3_) *δ* 197.1, 170.0, 148.0, 138.2, 133.4, 128.3, 127.1, 124.4, 92.1, 88.8, 67.1, 56.2, 36.2, 30.1, 26.7. **HRMS (EI)** calc'd for C_19_H_19_N_3_O_3_ [M˙]^+^: 337.1426, found: 337.1429.

### 4-(3-Oxo-3-(1-((tetrahydro-2*H*-pyran-4-yl)methyl)-1*H*-1,2,3-triazol-4-yl)prop-1-yn-1-yl)benzonitrile (8M)

Synthesized according to **GP**5 using 6E (0.07 g, 0.39 mmol) and 7D (0.06 g, 0.39 mmol), and purified by flash column chromatography (20% EtOAc/DCM *R*_f_ = 0.29, dry loading using celite) to give a pale-yellow solid (0.05 g, 38%). ^**1**^**H NMR** (600 MHz, CDCl_3_) *δ* 8.16 (s, 1H), 7.81 (d, *J* = 8.7 Hz, 2H), 7.71 (d, *J* = 8.7 Hz, 2H), 4.34 (d, *J* = 7.2 Hz, 2H), 4.00 (m, 2H), 3.37 (td, *J* = 11.8, 2.3 Hz, 2H), 2.23 (m,1H), 1.57–1.49 (m, 2H), 1.50–1.36 (m, 2H). ^**13**^**C NMR** (150 MHz, CDCl_3_) *δ* 169.9, 148.0, 133.7, 132.3, 127.3, 124.8, 118.0, 114.3, 90.7, 89.4, 77.3, 77.1, 76.9, 67.2, 56.4, 36.3, 30.3. **HRMS (EI)** calc'd for C_18_H_16_N_4_O_2_ [M˙]^+^: 320.1273, found: 320.1240.

### 3-(4-(Methylsulfonyl)phenyl)-1-(1-((tetrahydro-2*H*-pyran-4-yl)methyl)-1*H*-1,2,3-triazol-4-yl)prop-2-yn-1-one (8N)

Synthesized according to **GP**5 using 6F (0.08 g, 0.34 mmol) and 7D (0.05 g, 0.34 mmol), and purified by flash column chromatography (40% EtOAc/DCM *R*_f_ = 0.25, dry loading using celite) to give a pale-yellow solid (0.07 g, 55%). ^**1**^**H NMR** (300 MHz, CDCl_3_) *δ* 8.21 (s, 1H), 8.03 (d, *J* = 8.7 Hz, 2H), 7.93 (d, *J* = 8.7 Hz, 2H), 4.38 (d, *J* = 7.2 Hz, 2H), 4.03 (m, 2H), 3.40 (td, *J* = 11.7, 2.6 Hz, 2H), 3.12 (s, 3H), 2.26 (m, 1H), 1.59–1.39 (m, 4H). ^**13**^**C NMR** (75 MHz, CDCl_3_) *δ* 169.9, 147.9, 142.2, 133.9, 127.6, 127.3, 125.7, 90.6, 89.0, 67.1, 56.3, 44.4, 36.2, 30.1. **HRMS (EI)** calc'd for C_18_H_19_N_3_O_4_S [M˙]^+^: 373.1096, found: 373.1095.

### 1-(1-((Tetrahydro-2*H*-pyran-4-yl)methyl)-1*H*-1,2,3-triazol-4-yl)-3-(4-((trifluoromethyl)sulfonyl)phenyl)prop-2-yn-1-one (8O)

Synthesized according to **GP**5 using 6G (0.08 g, 0.28 mmol) and 7D (0.04 g, 0.28 mmol), and purified by flash column chromatography (15% EtOAc/DCM *R*_f_ = 0.51, dry loading using celite) to give a beige solid (0.04 g, 33%). ^**1**^**H NMR** (600 MHz, CDCl_3_) *δ* 8.18 (s, 1H), 8.09 (d, *J* = 8.8 Hz, 2H), 7.97 (d, *J* = 8.8 Hz, 2H), 4.35 (d, *J* = 7.2 Hz, 2H), 4.00 (m, 2H), 3.37 (td, *J* = 11.6, 2.5 Hz, 2H), 2.32–2.11 (m, 1H), 1.59–1.27 (m, 4H). ^**13**^**C NMR** (150 MHz, CDCl_3_) *δ* 169.6, 147.7, 134.1, 132.8, 130.8, 128.7, 127.3, 119.7 (q, *J*_C,F_ = 324.3 Hz), 90.3, 89.3, 67.1, 56.3, 36.2, 30.1. ^**19**^**F NMR** (282 MHz, CDCl_3_) *δ* –77.95. **HRMS (EI)** calc'd for C_18_H_16_F_3_N_3_O_4_S [M˙]^+^: 427.0814, found: 427.0787.

### 4-((4-(3-(4-(Methylsulfonyl)phenyl)propioloyl)-1*H*-1,2,3-triazol-1-yl)methyl)benzonitrile (8P)

Synthesized according to **GP**5 using 6F (0.06 g, 0.25 mmol) and 7G (0.04 g, 0.25 mmol), and purified by flash column chromatography (20% EtOAc/DCM *R*_f_ = 0.4, dry loading using celite) to give a white powder (0.07 g, 95%). ^**1**^**H NMR** (300 MHz, CDCl_3_) *δ* 8.19 (s, 1H), 8.02 (d, *J* = 8.2 Hz, 2H), 7.92 (d, *J* = 8.2 Hz, 2H), 7.74 (d, *J* = 8.0 Hz, 2H), 7.43 (d, *J* = 8.0 Hz, 2H), 5.71 (s, 2H), 3.11 (s, 3H). ^**13**^**C NMR** (75 MHz, CDCl_3_) *δ* 169.5, 148.5, 142.2, 138.4, 133.9, 133.1, 128.6, 127.6, 126.8, 125.5, 117.8, 113.4, 90.8, 88.8, 77.2, 77.0, 76.8, 53.9, 44.3. **HRMS (EI)** calc'd for C_20_H_14_N_4_O_3_S [M˙]^+^: 390.0787, found: 390.0778.

### 4-(3-(1-(4-Cyanobenzyl)-1*H*-1,2,3-triazol-4-yl)-3-oxoprop-1-yn-1-yl)-*N*,*N*-dimethylbenzenesulfonamide (8Q)

Synthesized according to **GP**5 using 6H (0.12 g, 0.44 mmol) and 7G (0.07 g, 0.44 mmol), and purified by flash column chromatography (15% EtOAc/DCM *R*_f_ = 0.53, dry loading using celite) to give a white powder (0.13 g, 72%). ^**1**^**H NMR** (300 MHz, CDCl_3_) *δ* 8.20 (s, 1H), 7.88 (d, *J* = 8.7 Hz, 2H), 7.83 (d, *J* = 8.7 Hz, 2H), 7.73 (d, *J* = 8.6 Hz, 2H), 7.43 (d, *J* = 8.6 Hz, 2H), 5.71 (s, 2H), 2.76 (s, 6H). ^**13**^**C NMR** (75 MHz, CDCl_3_) *δ* 169.6, 148.5, 138.5, 137.7, 133.7, 133.1, 128.6, 127.7, 126.9, 124.3, 117.8, 113.4, 91.3, 88.6, 53.8, 37.8. **HRMS (EI)** calc'd for C_21_H_17_N_5_O_3_S [M˙]^+^: 419.1052, found: 419.1056.

### 5-((4-(3-(4-(Methylsulfonyl)phenyl)propioloyl)-1*H*-1,2,3-triazol-1-yl)methyl)picolinonitrile (8R)

Synthesized according to **GP**5 using 6G (0.06 g, 0.25 mmol) and 7J (0.04 g, 0.25 mmol), and purified by flash column chromatography (50% EtOAc/DCM *R*_f_ = 0.40, dry loading using celite) to give an off-white powder (0.05 g, 47%). ^**1**^**H NMR** (600 MHz, DMSO-*d*_6_) *δ* 9.25 (s, 1H), 8.83 (s, 1H), 8.09–87.99 (m, 6H), 5.91 (s, 2H), 3.30 (s, 3H). ^**13**^**C NMR** (150 MHz, DMSO-*d*_6_) 168.3, 150.9, 146.9, 142.8, 137.6, 135.3, 133.8, 132.4, 130.8, 129.1, 127.6, 123.9, 117.3, 89.3, 88.5, 50.4, 43.1. **HRMS (ESI)** calc'd for C_19_H_13_N_5_O_3_SNa [M + Na]^+^: 414.0637, found: 414.0649.

### (1-((Tetrahydro-2*H*-pyran-4-yl)methyl)-1*H*-1,2,3-triazol-4-yl)methanol (9)

To a solution of propargyl alcohol (0.11 mL, 1.82 mmol, 1.0 eq.) and 7D (0.26 g, 1.82 mmol, 1.0 eq.) in 6 mL of *t*BuOH/H_2_O (2 : 1) were added CuSO_4_·5H_2_O (0.02 g, 0.09 mmol, 5 mol%) and sodium ascorbate (0.04 g, 0.18 mmol, 10 mol%). The mixture was stirred vigorously for 16 h at R.T. and then diluted with H_2_O (50 mL) and extracted with EtOAc (3 × 25 mL). The organic layer was washed with brine (50 mL), dried over MgSO_4_, and concentrated *in vacuo* to give a pale yellow oil (0.17 g, 50%) which was used without further purification (100% EtOAc, *R*_f_ = 0.13). ^**1**^**H NMR** (600 MHz, CDCl_3_) *δ* 7.54 (s, 1H), 4.24 (d, *J* = 7.2 Hz, 2H), 4.08–3.90 (m, 2H), 3.36 (td, *J* = 11.8, 2.1 Hz, 2H), 2.17 (m, 1H), 1.65–1.46 (m, 2H), 1.40 (m, 2H). ^**13**^**C NMR** (150 MHz, CDCl_3_) *δ* 147.8, 122.3, 67.2, 56.3, 55.8, 36.2, 30.2. **HRMS (EI)** calc'd for C_9_H_15_N_3_O_2_ [M˙]^+^: 197.1164, found: 197.1171.

### 1-((Tetrahydro-2*H*-pyran-4-yl)methyl)-1*H*-1,2,3-triazole-4-carbaldehyde (10)

To a solution of 9 (0.17 g, 0.91 mmol, 1.0 eq.) in acetone (10 mL) at 0 °C were added trichloroisocyanuric acid (0.19 g, 0.83 mmol, 1.05 eq.) and TEMPO (0.001 g, 0.008 mmol, 0.01 eq.). The reaction was allowed to gradually warm to R.T. over 3 h at which point TLC analysis confirmed completion (100% EtOAc, *R*_f_ = 0.68). The reaction was concentrated *in vacuo*, redissolved in DCM (50 mL) and washed with sat. NaHCO_3_ (25 mL), 1 M HCl (25 mL), and then brine (50 mL). The organic layer was dried over MgSO_4_ and concentrated *in vacuo* to give a yellow solid (0.16 g, >95%) which was used without further purification. ^**1**^**H NMR** (600 MHz, CDCl_3_) *δ* 10.16 (s, 1H), 8.10 (s, 2H), 4.34 (d, *J* = 7.2 Hz, 2H), 4.09–3.93 (m, 2H), 3.56–3.20 (td, *J* = 11.8, 2.1 Hz, 2H), 2.23 (m, 1H), 1.65–1.45 (m, 2H), 1.43 (m, 2H). ^**13**^**C NMR** (150 MHz, CDCl_3_) *δ* 185.1, 147.7, 125.5, 67.1, 56.2, 36.1, 30.1. **HRMS (EI)** calc'd for C_9_H_13_N_3_O_2_ [M˙]^+^: 195.1008, found: 195.1011.

### 1-(1-((Tetrahydro-2*H*-pyran-4-yl)methyl)-1*H*-1,2,3-triazol-4-yl)prop-2-yn-1-ol (11)

In a flamed dried flask under N_2_, 10 (0.10 g, 0.51 mmol, 1.0 eq.) was dissolved in anhydrous THF (8 mL) and the solution was cooled to −78 °C. Ethynylmagnesium bromide (0.5 M in THF) (2.00 mL, 1.02 mmol, 2.0 eq.) was then added dropwise. The reaction was stirred for 4 h, then quenched with H_2_O (10 mL) and sat. NH_4_Cl (5 mL), and extracted with EtOAc (3 × 50 mL). The organic layer was dried over MgSO_4_ and concentrated *in vacuo*. The resulting solid was purified by flash column chromatography (50% EtOAc/DCM, *R*_f_ = 0.29, dry loading using celite) to give a yellow oil (0.08 g, 75%). ^**1**^**H NMR** (600 MHz, CDCl_3_) *δ* 7.56 (s, 1H), 5.64 (d, *J* = 3.0 Hz, 1H), 4.18 (d, *J* = 7.2 Hz, 2H), 3.99–3.83 (m, 2H), 3.29 (td, *J* = 11.8, 2.2 Hz, 2H), 2.56 (d, *J* = 2.3 Hz, 1H), 2.11 (m, 1H), 1.53–1.40 (m, 2H), 1.34 (m, 2H). ^**13**^**C NMR** (150 MHz, CDCl_3_) *δ* 146.8, 121.1, 81.0, 72.9, 66.1, 55.9, 54.9, 35.2, 29.2, 29.2. **HRMS (EI)** calc'd for C_11_H_15_N_3_O_2_ [M˙]^+^: 221.1164, found: 221.1159.

### 1-(1-((Tetrahydro-2*H*-pyran-4-yl)methyl)-1*H*-1,2,3-triazol-4-yl)prop-2-yn-1-one (12)

To a solution of 11 (0.08 g, 0.36 mmol, 1.0 eq.) in acetone (5 mL) at 0 °C were added trichloroisocyanuric acid (0.09 g, 0.38 mmol, 1.05 eq.) and TEMPO (0.002 g, 0.004 mmol, 0.01 eq.). The reaction was allowed to gradually warm to R.T. over 3 h. The reaction was concentrated *in vacuo*, redissolved in DCM (10 mL) and washed with sat. NaHCO_3_ (10 mL), 1 M HCl (10 mL), and then brine (20 mL). The organic layer was dried over MgSO_4_ and concentrated *in vacuo* and purified by flash column chromatography (30% EtOAc/DCM, *R*_f_ = 0.62) to give a white solid (0.06 g, 75%). ^**1**^**H NMR** (600 MHz, CDCl_3_) *δ* 8.15 (s, 1H), 4.34 (d, *J* = 7.2 Hz, 2H), 4.03–3.93 (m, 2H), 3.53 (s, 1H), 3.38 (td, *J* = 11.9, 2.1 Hz, 2H), 2.23 (m, 1H), 1.55 (m, 2H), 1.44 (m, 2H). ^**13**^**C NMR** (150 MHz, CDCl_3_) *δ* 169.3, 147.5, 127.4, 81.4, 80.2, 67.1, 56.2, 36.1, 30.1. **HRMS (EI)** calc'd for C_11_H_13_N_3_O_2_ [M˙]^+^: 219.1008, found: 219.1008.

### 4-((4-(1-Hydroxy-3-(4-(methylsulfonyl)phenyl)prop-2-yn-1-yl)-1*H*-1,2,3-triazol-1-yl)methyl)benzonitrile (13)

To a solution of 8P (0.03 g, 0.06 mmol, 1.0 eq.) in MeOH (2 mL) at R.T. was added CeCl_3_·7H_2_O (0.05 g, 0.13, 2.0 eq.) and the mixture was stirred for 10 min before the addition of NaBH_4_ (0.005 g, 0.128 mmol, 2.0 eq.) and stirring for another 1 h. The reaction mixture was then concentrated *in vacuo*, diluted with EtOAc (20 mL) and washed with 1 M HCl (2 × 10 mL) and brine (20 mL). The organic layer was then dried over MgSO_4_ and concentrated *in vacuo* to give a yellow solid which was purified by preparative thin layer chromatography (2.5% MeOH/DCM *R*_f_ = 0.13, developed 3×) to give a white powder (0.02 g, 60%, mixture of enantiomers).^53^^**1**^**H NMR** (500 MHz, CDCl_3_) *δ* 7.93 (d, *J* = 8.7 Hz, 2H), 7.77–7.68 (m, 3H), 7.66 (d, *J* = 8.7 Hz, 2H), 7.42 (d, *J* = 8.4 Hz, 2H), 5.99 (s, 1H), 5.67 (s, 2H), 3.20 (s, 1H), 3.10 (s, 3H). ^**13**^**C NMR** (125 MHz, CDCl_3_) *δ* 148.4, 140.4, 139.3, 133.0, 132.6, 128.5, 127.8, 127.4, 121.7, 118.0, 113.0, 90.8, 84.0, 57.7, 53.6, 44.4. **HRMS (ESI)** calc'd for C_20_H_16_N_4_O_3_SNa [M + Na]^+^: 415.0837, found: 415.0841.

### Ethyl 3-(4-(methylsulfonyl)phenyl)propiolate (14)

In a flame dried round bottom flask filled with N_2_ were added 1G (0.50 g, 1.77 mmol, 1.0 eq.), PdCl_2_(PPh_3_)_2_ (0.03 g, 0.04 mmol, 2 mol%), CuI (0.01, 0.07 mmol, 4 mol%), and K_2_CO_3_ (0.50 g, 3.54 mmol, 2.0 eq.). The solids were dissolved in 5 mL of anhydrous THF followed by the addition of ethyl propiolate (0.72 mL, 7.08 mmol, 4.0 eq.). The reaction was heated to reflux and stirred for 24 h. The reaction was concentrated *in vacuo* and then diluted with H_2_O (100 mL) and EtOAc (50 mL) and filtered through celite. The layers of the filtrate were separated, and the aqueous layer was extract with EtOAc (2 × 50 mL). The combined organic layer was washed brine (100 mL) followed by drying over MgSO_4_ and concentrating *in vacuo* to give a brown solid. The residue was purified by flash column chromatography (40% EtOAc/Hex, *R*_f_ = 0.41, dry loading using celite) to give a brown solid (0.35 g, 77%). ^**1**^**H NMR** (600 MHz, CDCl_3_) *δ* 7.96 (d, *J* = 8.7 Hz, 2H), 7.76 (d, *J* = 8.7 Hz, 2H), 4.32 (q, *J* = 7.2 Hz, 2H), 3.07 (s, 3H), 1.37 (t, *J* = 7.2 Hz, 3H). ^**13**^**C NMR** (150 MHz, CDCl_3_) *δ* 153.3, 141.9, 133.5, 127.6, 125.4, 83.3, 83.0, 62.5, 44.3, 14.0. **HRMS (EI)** calc'd for C_12_H_12_O_4_S [M˙]^+^: 252.0456, found: 252.0435.

### 3-(4-(Methylsulfonyl)phenyl)propiolic acid (15)

To a solution of 14 (0.33 g, 1.31 mmol, 1.0 eq.) in acetone/H_2_O (5 : 1 mL) was added LiOH·H_2_O (0.22 g, 5.24 mmol, 4.0 eq.). The reaction was stirred for 4 h, concentrated *in vacuo*, and then diluted with sat. Na_2_CO_3_ (20 mL). The aqueous layer was washed with DCM (3 × 10 mL), acidified to pH ≈ 1, and extracted with EtOAc ((3 × 20 mL), The combined organic layer was washed brine (50 mL) followed by drying over MgSO_4_ and concentrating *in vacuo* to give a brown solid that was carried forward without further purification (40% EtOAc/Hex, *R*_f_ = 0) (0.22 g, >95%). ^**1**^**H NMR** (500 MHz, DMSO-*d*_6_) *δ* 8.01 (d, *J* = 8.7 Hz, 2H), 7.90 (d, *J* = 8.7 Hz, 2H), 3.29 (s, 3H). ^**13**^**C NMR** (125 MHz, DMSO-*d*_6_) *δ* 153.9, 142.2, 133.3, 127.4, 124.1, 83.9, 82.1, 43.1. **HRMS (EI)** calc'd for C_10_H_8_O_4_S [M˙]^+^: 224.0143, found: 224.0118.

### 1-(1*H*-Benzo[*d*][1,2,3]triazol-1-yl)-3-(4-(methylsulfonyl)phenyl)prop-2-yn-1-one (16)

To a solution of benzotriazole (0.21 g, 1.80 mmol, 4.0 eq.) in anhydrous DCM (3 mL) was added SOCl_2_ (0.03 mL, 0.45 mmol, 1.0 eq.) and the mixture was stirred for 30 min at R.T. followed by the addition of 15 (0.10 g, 0.45 mmol, 1.0 eq.). The reaction mixture was stirred for 3 h and then diluted with EtOAc (20 mL), filtered over celite, and washed with 2 M NaOH (3 × 10 mL). The organic layer was concentrated *in vacuo* to give a yellow solid which was washed with Et_2_O to give a white powder (0.04 g, 51%) (50% EtOAc/Hex *R*_f_ = 0.51). ^**1**^**H NMR** (400 MHz, CDCl_3_) *δ* 8.31 (d, *J* = 8.2 Hz, 1H), 8.18 (d, *J* = 8.2 Hz, 1H), 8.06 (d, *J* = 8.7 Hz, 2H), 7.98 (d, *J* = 8.7 Hz, 2H), 7.73 (t, *J* = 8.2 Hz, 1H), 7.58 (t, *J* = 8.2 Hz, 1H), 3.11 (s, 3H). ^**13**^**C NMR** (100 MHz, CDCl_3_) *δ* 149.9, 146.5, 142.9, 134.3, 131.0, 130.9, 127.9, 127.1, 124.8, 120.7, 114.3, 92.5, 83.4, 44.5. **HRMS (ESI)** calc'd for C_16_H_11_N_3_O_3_S [M + Na]^+^: 348.0419, found: 348.0389.

### 3-(4-(Methylsulfonyl)phenyl)-*N*-(prop-2-yn-1-yl)propiolamide (17)

To a solution of 15 (0.10 g, 0.45 mmol, 1.1 eq.) and propargyl amine (0.03 mL, 0.41 mmol, 1.0 eq.) in anhydrous DCM (3 mL) under N_2_ was added a solution of DCC (0.09 g, 0.25 mol, 1.1 eq.) and DMAP (0.001 g, 0.008 mmol, 2 mol%) in anhydrous DCM (2 mL). The reaction was stirred at R.T. for 3 h and then diluted EtOAc (20 mL) and filtered over celite. The organic layer was washed with 5% AcOH (3 × 10 mL), brine (20 mL), sat. NaHCO_3_ (3 × 10 mL), and then brine (20 mL) again. The combined organic layer was dried over MgSO_4_ and concentrated *in vacuo* to give a brown solid that was purified by flash column chromatography (50% EtOAc/Hex, *R*_f_ = 0.18) to give a pale-yellow solid (0.05 g, 42%). ^**1**^**H NMR** (500 MHz, CDCl_3_ + CD_3_OD) *δ* 7.93 (d, *J* = 6.5 Hz, 2H), 7.72 (d, *J* = 6.5 Hz, 2H), 4.14–4.00 (m, 1H), 3.47–3.37 (m, 2H), 3.13 (s, 4H), 1.69–1.66 (m, 1H). ^**13**^**C NMR** (125 MHz, CDCl_3_ + CD_3_OD) *δ* 152.5, 141.3, 133.2, 127.5, 125.9, 85.2, 82.7, 78.3, 71.9, 44.2, 33.7. **HRMS (EI)** calc'd for C_13_H_11_O_3_S [M˙]^+^: 261.0460, found: 261.0466.

### 3-(4-(Methylsulfonyl)phenyl)-*N*-((1-((tetrahydro-2*H*-pyran-4-yl)methyl)-1*H*-1,2,3-triazol-4-yl)methyl)propiolamide (18)

To a solution of 17 (0.05 g, 0.19 mmol, 1.0 eq.) and 7D (0.03 g, 0.19 mmol, 1.0 eq.) in *t*BuOH/H_2_O (2 : 2 mL) were added CuSO_4_·5H_2_O (0.003 g, 0.009 mmol, 5 mol%) and sodium ascorbate (0.004 g, 0.019 mmol, 10 mol%). The reaction mixture was stirred vigorously for 16 h and was then concentrated *in vacuo* to give a yellow solid, which was purified by flash column chromatography (2% MeOH/EtOAc *R*_f_ = 0.23, dry loading using celite) to give a white powder (0.04 g, 51%). ^**1**^**H NMR** (600 MHz, CDCl_3_) *δ* 7.98 (d, *J* = 8.6 Hz, 2H), 7.74 (d, *J* = 8.6 Hz, 2H), 7.64 (s, 1H), 6.97 (s, 1H), 4.67 (d, *J* = 5.7 Hz, 2H), 4.28 (d, *J* = 7.1 Hz, 2H), 4.02 (dd, *J* = 11.4, 2.9 Hz, 2H), 3.40 (td, *J* = 11.8, 2.3 Hz, 2H), 3.11 (s, 3H), 2.26–2.16 (m, 1H), 1.58–1.54 (m, 2H), 1.49–1.39 (m, 2H). ^**13**^**C NMR** (150 MHz, CDCl_3_) *δ* 152.6, 143.4, 141.6, 133.3, 127.6, 125.8, 122.9, 85.4, 82.7, 67.1, 55.9, 44.3, 36.2, 35.3, 30.3. **HRMS (ESI)** calc'd for C_19_H_22_N_4_O_4_SNa [M + Na]^+^: 425.1259, found: 425.1241.

### Ethyl 1-(4-cyanobenzyl)-1*H*-1,2,3-triazole-4-carboxylate (19A)

To a solution of 7G (0.20 g, 1.26 mmol, 1.0 eq.), ethyl propiolate (0.13 mL, 1.26 mmol, 1.0 eq.), and CuI (0.24 g, 1.26 mmol, 1.0 eq.) in anhydrous MeCN (10 mL) under N_2_ at R.T. was added DIPEA (0.22 mL, 1.26 mmol, 1.0 eq) dropwise and the reaction was stirred for 16 h. The reaction mixture was then diluted with DCM (20 mL) and washed with 1 M HCl (2 × 15 mL), brine (20 mL), sat. NH_4_OH (4 × 15 mL, until the aqueous phase was no longer blue), and brine (20 mL) again. The organic layer was then dried over MgSO_4_ and concentrated *in vacuo* to give a brown solid that was purified by flash column chromatography (10% EtOAc/DCM *R*_f_ = 0.47, dry loading using celite) to give the title compound as white fluffy needles (0.35 g, 78%). ^**1**^**H NMR** (500 MHz, CDCl_3_) *δ* 8.05 (s, 1H), 7.69 (d, *J* = 8.4 Hz, 2H), 7.37 (d, *J* = 8.4 Hz, 2H), 5.65 (s, 2H), 4.41 (q, *J* = 7.1 Hz, 2H), 1.39 (t, *J* = 7.1 Hz, 3H). ^**13**^**C NMR** (125 MHz, CDCl_3_) *δ* 160.6, 141.2, 139.0, 133.2, 128.7, 127.6, 118.1, 113.4, 77.4, 77.2, 76.9, 61.6, 53.8, 14.4. **HRMS (ESI)** calc'd for C_13_H_12_N_4_O_2_Na [M + Na]^+^: 279.0858, found: 279.0828.

### 1-(4-Cyanobenzyl)-1*H*-1,2,3-triazole-4-carboxylic acid (20A)

To a solution of 19A (0.10 g, 0.40 mmol, 1.0 eq.) in acetone/H_2_O (5 : 1 mL) was added LiOH·H_2_O (0.07 g, 1.60 mmol, 4.0 eq.). The reaction was stirred for 4 h, concentrated *in vacuo*, and then diluted with sat. Na_2_CO_3_ (10 mL). The aqueous layer was washed with DCM (3 × 10 mL), acidified to pH ≈ 1, and extracted with EtOAc (3 × 20 mL). The combined organic layer was washed brine (20 mL) followed by drying over MgSO_4_ and concentrating *in vacuo* to give a white powder that was carried forward without further purification (50% EtOAc/Hex, *R*_f_ = 0) (0.08 g, 88%). ^**1**^**H NMR** (500 MHz, DMSO-*d*_6_) *δ* 8.82 (s, 1H), 7.86 (d, *J* = 8.7 Hz, 2H), 7.49 (d, *J* = 8.7 Hz, 2H), 5.77 (s, 2H). ^**13**^**C NMR** (125 MHz, DMSO-*d*_6_) *δ* 162.0, 141.4, 140.5, 129.9, 129.3, 119.0, 111.5, 52.9. **HRMS (ESI)** calc'd for C_11_H_8_N_4_O_2_Na [M + Na]^+^: 251.0545, found: 251.0557.

### *tert*-Butyl (1-(4-cyanobenzyl)-1*H*-1,2,3-triazol-4-yl)carbamate (21A)

To a suspension of 20A (0.83 g, 3.65 mmol, 1.0 eq.) in *t*BuOH (20 mL) were added NEt_3_ (0.63 mL, 4.40 mmol, 1.2 eq.) and DPPA (0.95 mL, 4.40 mmol, 1.2 eq.). The reaction was stirred for 24 h at 90 °C and then concentrated *in vacuo*. The crude residue was purified by flash column chromatography (70% EtOAc/Hex *R*_f_ = 0.71, dry loading using celite) to give a white powder (0.41 g, 38%). ^**1**^**H NMR** (500 MHz, CDCl_3_) *δ* 7.60 (s, 1H), 7.56 (d, *J* = 8.3 Hz, 2H), 7.25 (d, *J* = 8.3 Hz, 2H), 7.19 (s, 1H), 5.41 (s, 2H), 1.39 (s, 9H). ^**13**^**C NMR** (125 MHz, CDCl_3_) *δ* 152.1, 145.0, 139.7, 132.9, 128.5, 118.2, 112.9, 111.6, 81.5, 53.9, 28.2. **HRMS (ESI)** calc'd for C_15_H_17_N_5_O_2_Na [M + Na]^+^: 322.1280, found: 322.1304.

### 4-((4-Amino-1*H*-1,2,3-triazol-1-yl)methyl)benzonitrile hydrochloride (22A)

A solution of 21A (0.20 g, 0.67 mmol) in DCM : 4 M HCl in Dioxanes (2 mL : 2 mL) was stirred at R.T. for 16 h. The reaction was concentrated, and the residue was washed with Et_2_O to give a yellow solid (0.13 g, >95%) which was used without further purification. ^**1**^**H NMR** (500 MHz, DMSO-*d*_6_) *δ* 8.27 (s, 1H), 7.88 (d, *J* = 8.3 Hz, 2H), 7.50 (d, *J* = 8.3 Hz, 2H), 5.75 (s, 2H). ^**13**^**C NMR** (125 MHz, DMSO-*d*_6_) *δ* 141.0, 139.7, 132.8, 128.8, 118.5, 117.3, 111.1, 52.8. **HRMS (ESI)** calc'd for C_10_H_10_N_5_ [M]^+^: 200.0936, found: 200.0925.

### Ethyl 1-((tetrahydro-2*H*-pyran-4-yl)methyl)-1*H*-1,2,3-triazole-4-carboxylate (19B)

To a solution of 7D (0.40 g, 2.83 mmol, 1.0 eq.), ethyl propiolate (0.30 mL, 2.83 mmol, 1.0 eq.), and CuI (0.54 g, 2.83 mmol, 1.0 eq.) in anhydrous MeCN (10 mL) under N_2_ at R.T. was added DIPEA (0.50 mL, 2.83 mmol, 1.0 eq.) dropwise and the reaction was stirred for 16 h. The reaction mixture was then diluted with DCM (50 mL) and washed with 1 M HCl (2 × 25 mL), brine (25 mL), sat. NH_4_OH (4 × 25 mL, until aqueous phase was no longer blue), and brine (25 mL) again. The organic layer was then dried over MgSO_4_ and concentrated *in vacuo* to give an orange solid which was washed with Et_2_O to give the title compound as pale-orange powder (0.50 g, 73%) that was carried forward without further purification. ^**1**^**H NMR** (500 MHz, CDCl_3_) *δ* 8.05 (s, 1H), 4.42 (q, *J* = 7.1 Hz, 2H), 4.29 (d, *J* = 7.1 Hz, 2H), 4.04–3.90 (m, 2H), 3.35 (td, *J* = 11.7, 2.4 Hz, 2H), 2.19 (m, 1H), 1.61–1.14 (m, 7H). ^**13**^**C NMR** (125 MHz, CDCl_3_) *δ* 160.9, 140.4, 127.9, 67.3, 61.5, 56.2, 36.3, 30.3, 14.5. **HRMS (ESI)** calc'd for C_11_H_17_N_3_O_3_Na [M + Na]^+^: 262.1184, found: 262.1168.

### 1-((Tetrahydro-2*H*-pyran-4-yl)methyl)-1*H*-1,2,3-triazole-4-carboxylic acid (20B)

To a solution of 19B (0.50 g, 2.09 mmol, 1.0 eq.) in acetone/H_2_O (20 : 5 mL) was added LiOH·H_2_O (0.35 g, 8.36 mmol, 4.0 eq.). The reaction was stirred for 4 h, concentrated *in vacuo*, and then diluted with sat. Na_2_CO_3_ (25 mL). The aqueous layer was washed with DCM (3 × 25 mL), acidified to pH ≈ 1, and extracted with EtOAc (3 × 25 mL). The combined organic layer was washed with brine (25 mL), dried over MgSO_4_, and concentrated *in vacuo* to give a white powder that was carried forward without further purification (50% EtOAc/Hex, *R*_f_ = 0) (0.21 g, 50%). ^**1**^**H NMR** (500 MHz, DMSO-*d*_6_) *δ* 8.68 (s, 1H), 4.32 (d, *J* = 7.2 Hz, 2H), 3.82 (dd, *J* = 11.5, 2.6 Hz, 1H), 3.24 (td, *J* = 11.5, 2.6 Hz, 2H), 2.10 (m,1H), 1.62–0.96 (m, 4H). ^**13**^**C NMR** (125 MHz, DMSO-*d*_6_) *δ* 162.2, 140.0, 129.8, 66.8, 55.2, 35.8, 30.1. **HRMS (ESI)** calc'd for C_9_H_13_N_3_O_2_Na [M + Na]^+^: 234.0837, found: 234.0855.

### *tert*-Butyl (1-((tetrahydro-2*H*-pyran-4-yl)methyl)-1*H*-1,2,3-triazol-4-yl)carbamate (21B)

To a suspension of 20B (0.90 g, 4.26 mmol, 1.0 eq.) in tBuOH (20 mL) were added NEt_3_ (0.90 mL, 6.39 mmol, 1.2 eq.) and DPPA (1.40 mL, 6.39 mmol, 1.2 eq.). The reaction was stirred for 24 h at 90 °C and then concentrated *in vacuo*. The crude residue was purified by flash column chromatography (80% EtOAc/Hex *R*_f_ = 0.48, dry loading using celite) to give a white powder (0.59 g, 49%). ^**1**^**H NMR** (600 MHz, CDCl_3_) *δ* 7.71 (s, 1H), 7.15 (s, 1H), 4.17 (d, *J* = 7.2 Hz, 2H), 3.97 (apparent dd, *J* = 9.4, 4.6 Hz, 2H), 3.36 (td, *J* = 11.8, 2.2 Hz, 2H), 2.16 (m, 2H), 1.57–1.44 (m, 11H), 1.40 (m, 2H). ^**13**^**C NMR** (150 MHz, CDCl_3_) *δ* 152.2, 144.2, 112.0, 81.3, 67.3, 56.3, 36.2, 30.3, 28.3. **HRMS (ESI)** calc'd for C_13_H_22_N_4_O_3_Na [M + Na]^+^: 305.1590, found: 305.1595.

### 1-((Tetrahydro-2*H*-pyran-4-yl)methyl)-1*H*-1,2,3-triazol-4-amine hydrochloride (22B)

A solution of 21B (0.25 g, 0.89 mmol) in DCM : 4 M HCl in dioxanes (3 mL : 3 mL) was stirred at R.T. for 16 h. The reaction was concentrated, and the residue was washed with Et_2_O to give a white powder (0.20 g, >95%) which was used without further purification. ^**1**^**H NMR** (500 MHz, DMSO-*d*_6_) *δ* 8.11 (s, 1H), 4.30 (d, *J* = 7.1 Hz, 2H), 3.91–3.74 (m, 2H), 3.26 (td, *J* = 11.5, 2.5, 2H), 2.09 (m, 1H), 1.44–1.37 (m, 2H), 1.34–1.11 (m, 2H). ^**13**^**C NMR** (125 MHz, DMSO-*d*_6_) *δ* 139.8, 117.5, 66.8, 55.7, 35.9, 30.1. **HRMS (ESI)** calc'd for C_8_H_15_N_4_ [M]^+^: 183.1246, found: 183.1248.

### General procedure 6 (**GP**6): amide coupling with TCFH and NMI^30^

To a solution of propiolic acids (1.0 eq., commercially available unless indicated otherwise), 22A or 22B (1.3 eq.), and NMI (3.5 eq.) in anhydrous MeCN (∼0.1 M relative to acid) at R.T. was added TCFH (1.2 eq.). The reaction was stirred at R.T. for 3 h and then diluted with EtOAc (20 mL). The organic layer was washed with 5% AcOH (3 × 10 mL), brine (20 mL), sat. NaHCO_3_ (3 × 10 mL), and brine again (20 mL). The organic layer was then dried over MgSO_4_ and concentrated *in vacuo*. The crude residue was purified by flash column chromatography as described below.

### *N*-(1-(4-Cyanobenzyl)-1*H*-1,2,3-triazol-4-yl)-3-(4-(methylsulfonyl)phenyl)propiolamide (23A)

Synthesized according to **GP**6 using 15 (0.08 g, 0.39 mmol) and 22A (0.12 g, 0.50 mmol), and purified by flash column chromatography (40% EtOAc/Hex *R*_f_ = 0.40, dry loading using celite) to give a white powder (0.06 g, 36%). ^**1**^**H NMR** (500 MHz, DMSO-*d*_6_) *δ* 12.08 (s, 1H), 8.39 (s, 1H), 8.03 (d, *J* = 8.5 Hz, 2H), 7.91 (d, *J* = 8.5 Hz, 2H), 7.86 (d, *J* = 8.4 Hz, 2H), 7.49 (d, *J* = 8.4 Hz, 2H), 5.72 (s, 2H), 3.28 (s, 3H). ^**13**^**C NMR** (125 MHz, DMSO-*d*_6_) *δ* 148.8, 142.6, 142.1, 141.9, 141.4, 133.3, 142.8, 128.8, 127.5, 124.6, 118.6, 115.3, 110.9, 85.4, 83.4, 52.5, 43.2. **HRMS (ESI)** calc'd for C_20_H_15_N_5_O_3_Na [M + Na]^+^: 428.0793, found: 428.0811.

### 3-(4-(Methylsulfonyl)phenyl)-*N*-(1-((tetrahydro-2*H*-pyran-4-yl)methyl)-1*H*-1,2,3-triazol-4-yl)propiolamide (23B)

Synthesized according to **GP**6 using 15 (0.16 g, 0.70 mmol) and 22B (0.20 g, 0.91 mmol), and the resulting residue was suspended in MeOH (10 mL) and DCM (1 mL) and then filtered to give the title compound as a pale-yellow solid which was used without further purification (0.02 g, 7%) (50% EtOAc/Hex *R*_f_ = 0.59). ^**1**^**H NMR** (600 MHz, CDCl_3_) *δ* 11.98 (s, 1H), 8.21 (s, 1H), 8.00 (d, *J* = 8.7 Hz, 2H), 7.87 (d, *J* = 8.7 Hz, 1H), 4.24 (d, *J* = 7.1 Hz, 2H), 3.79 (apparent d, *J* = 11.5 Hz, 3H), 3.25–3.17 (m, 5H), 2.10–2.02 (m, 1H), 1.53–1.12 (m, 4H). ^**13**^**C NMR** (150 MHz, CDCl_3_) *δ* 149.2, 142.7, 142.6, 133.7, 128.0, 125.1, 115.8, 86.0, 83.8, 66.9, 55.4, 55.4, 43.7, 36.0, 30.2. **HRMS (ESI)** calc'd for C_18_H_20_N_4_O_4_Na [M + Na]^+^: 411.1103, found: 411.1108.

### 3-Phenyl-*N*-(1-((tetrahydro-2*H*-pyran-4-yl)methyl)-1*H*-1,2,3-triazol-4-yl)propiolamide (23C)

Synthesized according to **GP**6 using phenylpropiolic acid (0.07 g, 0.46 mmol) and 22B (0.12 g, 0.55 mmol), and the resulting residue was suspended in MeOH (5 mL) and DCM (0.5 mL) and then filtered to give the title compound as a pale-yellow solid which was used without further purification (0.03 g, 21%) (50% EtOAc/Hex *R*_f_ = 0.65). ^**1**^**H NMR** (600 MHz, DMSO-*d*_6_) *δ* 8.21 (s, 1H), 7.80–7.52 (m, 2H), 7.60–7.23 (m, 3H), 4.27 (d, *J* = 7.1 Hz, 2H), 3.83 (apparent dd, *J* = 12.0, 4.1 Hz, 2H), 3.25 (td, *J* = 11.7, 2.3 Hz, 2H), 2.09 (ddd, *J* = 11.3, 7.4, 4.0 Hz, 1H), 1.49–1.05 (m, 4H). ^**13**^**C NMR** (150 MHz, CDCl_3_) *δ* 149.7, 142.9, 132.9, 131.2, 129.5, 120.0, 115.6, 85.8, 83.8, 66.9, 55.4, 36.0, 30.2. **HRMS (ESI)** calc'd for C_17_H_18_N_4_O_2_Na [M + Na]^+^: 333.1327, found: 333.1337.

### *N*-(1-((Tetrahydro-2*H*-pyran-4-yl)methyl)-1*H*-1,2,3-triazol-4-yl)but-2-ynamide (23D)

Synthesized according to **GP**6 using 2-butynoic acid (0.02 g, 0.27 mmol) and 22B (0.07 g, 0.35 mmol), and purified by flash column chromatography (50% EtOAc/Hex *R*_f_ = 0.54, dry loading using celite) to give a white powder (0.02 g, 31%). ^**1**^**H NMR** (300 MHz, CDCl_3_) *δ* 8.70 (s, 1H), 7.97 (s, 1H), 4.20 (d, *J* = 7.2 Hz, 2H), 3.98 (apparent dd, *J* = 12.2, 4.3 Hz, 2H), 3.36 (td, *J* = 11.7, 2.4 Hz, 2H), 2.16 (ddd, *J* = 11.4, 7.9, 4.0 Hz, 1H), 1.53–1.30 (m, 4H). ^**13**^**C NMR** (150 MHz, CDCl_3_) *δ* 149.8, 142.8, 114.3, 86.4, 74.4, 67.4, 56.5, 36.3, 30.4, 4.0. **HRMS (ESI)** calc'd for C_12_H_16_N_4_O_2_Na [M + Na]^+^: 271.1171, found: 271.1192.

### *N*-(1-((Tetrahydro-2*H*-pyran-4-yl)methyl)-1*H*-1,2,3-triazol-4-yl)propiolamide (23E)

Synthesized according to **GP**6 using propiolic acid (0.02 mL, 0.30 mmol) and 22B (0.08 g, 0.37 mmol), and purified by flash column chromatography (50% EtOAc/Hex *R*_f_ = 0.51, dry loading using celite) to give a pale-yellow solid (0.03 g, 43%). ^**1**^**H NMR** (600 MHz, CDCl_3_ + CD_3_OD) *δ* 7.95 (s, 1H), 4.15 (d, *J* = 7.2 Hz, 3H), 3.97–3.82 (m, 2H), 3.33–3.29 (m, 2H, overlapping with residual methanol), 3.07 (s, 1H), 2.09 (ddd, *J* = 11.1, 7.4, 3.8 Hz, 1H), 1.49–1.41 (m, 2H), 1.41–1.27 (m, 2H). ^**13**^**C NMR** (150 MHz, CDCl_3_ + CD_3_OD) *δ* 149.1, 142.5, 114.8, 76.4, 75.5, 75.5, 67.1, 56.1, 35.9, 30.0. **HRMS (ESI)** calc'd for C_11_H_14_N_4_O_2_Na [M + Na]^+^: 257.1014, found: 257.1034.

### *tert*-Butyl 4-(4-nitrobenzoyl)piperazine-1-carboxylate (24)

4-Nitrobenzoic acid (0.49 g, 2.95 mmol, 1.1 eq.), HBTU (1.32 g, 3.48 mmol, 1.3 eq.), and DIPEA (1.40 mL, 8.04 mmol, 3.0 eq.) were stirred in DCM (25 mL) at R.T. for 15 min, after which *N*-Boc-piperazine (0.50 g, 2.68 mmol, 1.0 eq.) was added. The reaction was stirred for 3 h and then diluted with EtOAc (100 mL). The organic layer was washed with 5% AcOH (3 × 25 mL), brine (50 mL), sat. NaHCO_3_ (50 mL), and brine (50 mL) again, and then dried over MgSO_4_ and concentrated *in vacuo* to give a yellow solid (0.90 g, >95%), which was used without further purification. ^**1**^**H NMR** (300 MHz, CDCl_3_) *δ* 8.29 (d, *J* = 8.9 Hz, 2H), 7.57 (d, *J* = 8.9 Hz, 2H), 3.76–3.35 (m, 8H), 1.46 (s, 9H). ^**13**^**C NMR** (75 MHz, CDCl_3_) *δ* 167.3, 153.8, 147.8, 142.0, 128.3, 123.7, 79.2, 46.7 (2C), 41.4 (2C), 28.0. Characterization data are consistent with previously reported values.^54^

### (4-Nitrophenyl)(piperazin-1-yl)methanone hydrochloride (25)

To a solution of 24 (0.50 g, 1.50 mmol, 1.0 eq.) in DCM (5 mL) was added 4 M HCl/dioxane (5 mL) and the reaction was stirred for 3 h at R.T. Upon completion, the reaction was concentrated *in vacuo* and the resulting solid was washed with DCM (3 × 10 mL) to give a white solid (0.37 g, 93%). ^**1**^**H NMR** (300 MHz, DMSO-*d*_6_) *δ* 9.41 (br s, 2H), 8.31 (d, *J* = 8.9 Hz, 2H), 7.75 (d, *J* = 8.9 Hz, 2H), 3.85 (apparent br s, 2H), 3.50 (apparent br s, 2H), 3.18–3.12 (m, 4H). ^**13**^**C NMR** (75 MHz, DMSO-*d*_6_) *δ* 168.2, 148.5, 142.2, 128.2, 124.1, 49.0, 46.6, 46.0, 43.5. Characterization data are consistent with previously reported values.^54^

### *tert*-Butyl (2-(4-(4-nitrobenzoyl)piperazin-1-yl)-2-oxoethyl)carbamate (26)

*N*-Boc-glycine (0.25 g, 1.43 mmol, 1.1 eq.), HBTU (0.64 g, 1.69 mmol, 1.3 eq.), and DIPEA (0.90 mL, 5.20 mmol, 3.0 eq.) were stirred in DCM (15 mL) at R.T. for 15 min after which 25 (0.37 g, 1.30 mmol, 1.0 eq.) was added. The reaction was stirred for 3 h and then diluted with EtOAc (100 mL). The organic layer was washed with 5% AcOH (3 × 25 mL), brine (50 mL), sat. NaHCO_3_ (50 mL), and brine (50 mL) again, and then dried over MgSO_4_ and concentrated *in vacuo*. The residue was washed with hexanes (3 × 25 mL) to give a yellow solid (0.44 g, 85%), which was used without further purification. ^**1**^**H NMR** (300 MHz, CDCl_3_) *δ* 8.31 (d, *J* = 8.9 Hz, 2H), 7.59 (d, *J* = 8.9 Hz, 2H), 5.43 (br s, 1H), 3.99 (br s, 2H), 3.81–3.41 (m, 8H), 1.44 (s, 9H). ^**13**^**C NMR** (75 MHz, CDCl_3_) *δ* 168.4, 167.5, 155.9, 148.8, 141.1, 128.3, 124.2, 80.1, 47.3, 44.3, 42.3 (3C), 28.5. **HRMS (ESI)** calc'd for C_18_H_24_N_4_O_6_Na [M + Na]^+^: 415.1576, found: 415.1594.

### 2-Amino-1-(4-(4-nitrobenzoyl)piperazin-1-yl)ethan-1-one (27)

26 (0.38 g, 0.97 mmol, 1.0 eq.) was dissolved in DCM (5.0 mL) and treated with TFA (1.00 mL, 5.90 mmol, 6.0 eq.) until the starting material was completely consumed by TLC (10% MeOH/DCM) in 3 h. The reaction mixture was diluted with DCM (15 mL) and washed with 0.5 M HCl (3 × 25 mL). The pH of the combined aqueous layer was adjusted to 9–10 using sat. K_2_CO_3_ and then extracted using DCM (3 × 35 mL). The combined organic layer was dried over MgSO_4_ and concentrated *in vacuo* to give a white solid (0.12 g, 44%) (10% MeOH/DCM, *R*_f_ = 0.27). ^**1**^**H NMR** (300 MHz, DMSO-*d*_6_) *δ* 8.29 (d, *J* = 8.9 Hz, 2H), 7.70 (d, *J* = 8.9 Hz, 2H), 3.61–3.27 (m, 19H). ^**13**^**C NMR** (75 MHz, DMSO-*d*_6_) *δ* 171.5, 167.3, 147.9, 142.0, 128.4, 123.8, 46.7, 43.3, 42.6 41.4. **HRMS (EI)** calc'd for C_13_H_16_N_4_O_4_ [M˙]^+^: 292.1172, found: 292.1175.

### *N*-(2-(4-(4-Nitrobenzoyl)piperazin-1-yl)-2-oxoethyl)acrylamide (28)

27 (0.12 g, 0.35 mmol, 1.0 eq.) and DIPEA (0.20 mL, 1.10 mmol, 3.0 eq.) were dissolved in anhydrous DCM (5.0 mL) and cooled to 0 °C. To this solution was added acryloyl chloride (0.0630 mL, 0.39 mmol, 1.1 eq.) and the reaction was stirred under nitrogen for 3 h. The reaction was concentrated and purified by flash column chromatography (5% MeOH/EtOAc, *R*_f_ = 0.33) to give a white solid (0.12 g, 90%). ^**1**^**H NMR** (300 MHz, CDCl_3_) *δ* 8.31 (d, *J* = 8.9 Hz, 2H), 7.59 (d, *J* = 8.9 Hz, 2H), 6.67 (br s, 1H), 6.35–6.29 (m, 2H), 5.69 (dd, *J* = 9.9, 1.8 Hz, 1H), 4.18 (s, 2H), 3.81–3.45 (m, 8H). ^**13**^**C NMR** (75 MHz, CDCl_3_) *δ* 168.4, 167.0, 165.5, 148.8, 141.0, 130.3, 128.3, 127.3, 124.2, 47.2, 44.4, 42.2 (2C), 41.4. **HRMS (ESI)** calc'd for C_16_H_18_N_4_O_5_Na [M + Na]^+^: 369.1175, found: 369.1171.

### *tert*-Butyl (2-(methoxy(methyl)amino)-2-oxoethyl)carbamate (29)

To a solution of *N*-Boc-Gly (2.00 g, 11.42 mmol, 1.0 eq.), *N*,*O*-dimethylhydroxylamine hydrochloride (1.23 g, 12.56, 1.1 eq.), and DIPEA (8.00 mL, 45.68 mmol, 4.0 eq.) in DCM (50 mL) at 0 °C were added EDCI-HCl (2.41 g, 12.56 mmol, 1.1 eq.) and DMAP (0.14 g, 1.14 mmol, 0.1 eq.). The reaction was allowed to warm to R.T. and stirred for 24 h. The reaction was diluted with EtOAc (150 mL) and washed with 1 M HCl (50 mL), brine (50 mL), sat. NaHCO_3_ (2 × 50 mL), and brine again (50 mL). The organic layer was then dried over MgSO_4_ and concentrated *in vacuo* to give the product as a white solid (1.50 g, 60%). ^**1**^**H NMR** (300 MHz, CDCl_3_) *δ* 5.26 (br s, 1H), 4.08–4.09 (d, *J* = 5.1 Hz, 2H), 3.71 (s, 3H), 3.20 (s, 3H), 1.45 (s, 9H). ^**13**^**C NMR** (75 MHz, CDCl_3_) *δ* 170.3, 156.0, 79.6, 61.5, 41.7, 32.4, 28.3. Characterization data are consistent with previously reported values.^55^

### *tert*-Butyl (2-oxobut-3-yn-1-yl)carbamate (30)

In a flame-dried round-bottom flask flushed with nitrogen, 29 (0.97 g, 4.60 mmol, 1.0 eq.) was dissolved in in anhydrous THF (80 mL) and the solution was cooled to −78 °C. Ethynyl magnesium bromide (0.5 M in THF) (37.00 mL, 18.40 mmol, 4.0 eq.) was then added dropwise and the reaction was allowed to warm to R.T. over 16 h. The reaction was quenched with cold 1 M NaHSO_4_ (50 mL) and concentrated to removed majority of the THF. The aqueous layer was then extracted with EtOAc (3 × 100 mL). The combined organic layer was washed with sat. NaHCO_3_ (100 mL) and brine (100 mL), dried over MgSO_4_, and concentrated *in vacuo* to give an orange oil (0.83 g, >95%) which was used without further purification. ^**1**^**H NMR** (300 MHz, CDCl_3_) *δ* 5.16 (br s, 1H), 4.21–4.19 (d, *J* = 5.1 Hz, 2H), 3.38 (s, 1H), 1.48 (s, 9H). ^**13**^**C NMR** (75 MHz, CDCl_3_) *δ* 183.1, 155.6, 81.7, 80.3, 79.7, 52.2, 28.3. Characterization data are consistent with previously reported values.^55^

### *tert*-Butyl (2-(1-((adamantan-1-yl)methyl)-1*H*-1,2,3-triazol-4-yl)-2-oxoethyl)carbamate (31)

To a solution of 30 (0.50 g, 2.73 mmol, 1.0 eq.) and 7C (0.52 g, 2.73 mmol, 1.0 eq.) in 1 : 1 *t*BuOH/H_2_O (14 mL) were added freshly prepared aqueous solutions of CuSO_4_·5H_2_O (50 mg mL^−1^) (0.70 mL, 0.14 mmol, 0.05 eq.) and 1 M sodium ascorbate (0.55 mL, 0.55 mmol, 0.2 eq.). The reaction mixture was stirred vigorously overnight, at which point the reaction was still in complete by TLC. Another 0.2 mL of CuSO_4_·5H_2_O solution and 0.1 mL of sodium ascorbate solution were added, and the reaction was stirred for another 3 h, after which the reaction mixture was diluted with water (25 mL) and extracted with EtOAc (3 × 20 mL). The combined organic layer was dried over MgSO_4_ and concentrated *in vacuo* to give a yellow oil which was purified by flash column chromatography (dry loading with celite, 40% EtOAc/hexanes, *R*_f_ = 0.38) to give a white foam (0.46 g, 40%). ^**1**^**H NMR** (300 MHz, CDCl_3_) *δ* 8.01 (s, 1H), 5.32 (br s, 1H), 4.76–4.74 (d, *J* = 5.1 Hz, 2H), 4.07 (s, 2H), 2.00 (apparent s, 3H), 1.72–1.55 (m, 6H), 1.48 (apparent s, 6H), 1.46 (s, 9H). ^**13**^**C NMR** (75 MHz, CDCl_3_) *δ* 190.0, 155.9, 145.4, 127.0, 79.9, 62.6, 48.3, 40.2, 36.5, 34.3, 28.5, 28.1. **HRMS (ESI)** calc'd for C_16_H_18_N_4_O_5_Na [M + Na]^+^: 397.2216, found: 397.2203.

### 1-(1-((Adamantan-1-yl)methyl)-1*H*-1,2,3-triazol-4-yl)-2-aminoethan-1-one hydrochloride (32)

To a solution of 31 (0.24 g, 0.64 mmol, 1.0 eq.) in DCM (5 mL) was added 5 mL of HCl (4 M in dioxane). The reaction was stirred for 4 h at room temperature and then concentrated *in vacuo* to give an off-white solid which was washed with DCM (3 × 20 mL) and used without further purification (0.18 g, 89%). ^**1**^**H NMR** (300 MHz, CD_3_OD) *δ* 8.65 (s, 1H), 4.58 (s, 2H), 4.19 (s, 2H), 1.97 (apparent s, 3H), 1.73–1.61 (m, 6H), 1.55–1.54 (m, 6H). ^**13**^**C NMR** (75 MHz, CD_3_OD) *δ* 185.6, 128.5, 126.9, 61.7, 42.0, 39.8, 36.2, 33.8, 28.2. **HRMS (ESI)** calc'd for C_15_H_23_N_4_O [M]^+^: 275.1872, found: 275.1890.

### *N*-(2-(1-((Adamantan-1-yl)methyl)-1*H*-1,2,3-triazol-4-yl)-2-oxoethyl)acrylamide (33)

A solution of 32 (0.15 g, 0.48 mmol, 1.0 eq.) and DIPEA (0.25 mL, 1.44 mmol, 3.0 eq.) in anhydrous DCM (7 mL) was stirred at 0 °C until complete dissolution of the amine. Acryloyl chloride (0.04 mL, 0.53 mmol, 1.1 eq.) was then added dropwise and the reaction was allowed to stir at R.T. for 3 h and then concentrated *in vacuo*. The crude product was purified by flash column chromatography (dry loading using celite, 100% EtOAc, *R*_f_ = 0.58) to give a white solid (0.11 g, 69%). ^**1**^**H NMR** (300 MHz, CDCl_3_) *δ* 8.05 (s, 1H), 6.61 (apparent s, 1H), 6.36–6.18 (m, 2H), 5.68 (dd, *J* = 9.6, 1.5 Hz, 1H), 4.94 (d, *J* = 4.8 Hz, 1H), 4.07 (s, 2H), 1.99 (apparent s, 3H), 1.71–1.54 (m, 6H), 1.49 (apparent s, 6H). ^**13**^**C NMR** (75 MHz, CDCl_3_) *δ* 189.3, 165.6, 145.2, 130.5, 127.2, 127.1, 62.6, 47.3, 40.2, 36.5, 34.3, 28.1. **HRMS (EI)** calc'd for C_18_H_24_N_4_O_2_ [M˙]^+^: 328.1899, found: 328.1874.

### 1-(4-(Adamantane-1-carbonyl)piperazin-1-yl)-3-(4-nitrophenyl)prop-2-yn-1-one (36)

To a stirring solution of 35, which was synthesized as previously described,^20^ (0.19 g, 0.58 mmol, 1.1 eq.) and 34, which was synthesized as previously described,^24^ (0.10 g, 0.52 mmol, 1.0 eq.) in anhydrous DCM (10 mL) was added HBTU (0.32 g, 0.84 mmol, 1.6 eq.) and Hünig's base (0.36 mL, 2.09 mmol, 4.0 eq.). The reaction mixture was stirred overnight at R.T, under N_2_. The reaction was confirmed complete by TLC (10% MeOH/DCM). The reaction mixture was concentrated *in vacuo* and the solid was resuspended in EtOAc (30 mL). The organic phase was washed with 5% AcOH (3 × 25 mL), brine (25 mL), NaHCO_3_ (25 mL) and then brine again (25 mL). The organic phase was dried over MgSO_4_ and concentrated *in vacuo* to give a yellow solid, which was purified by silica gel column chromatography (10% MeOH/DCM, *R*_f_ = 0.74, dry loading using celite), giving a pale-yellow solid (0.18 g, 80%). ^**1**^**H NMR** (300 MHz, CDCl_3_) *δ* 8.30–8.20 (d, *J* = 9.0 Hz, 2H), 7.77–7.67 (d, *J* = 9.0 Hz, 2H), 3.84–3.64 (m, 8H), 2.14–2.04 (m, 3H), 2.04–1.90 (m, 6H), 1.86–1.65 (m, 6H). ^**13**^**C NMR** (75 MHz, CDCl_3_) *δ* 176.2, 152.3, 148.3, 133.2, 126.9, 123.8, 88.5, 84.5, 47.3, 45.3, 42.1, 39.2, 36.5, 28.4. **HRMS (ESI)** calc'd for C_24_H_27_N_3_O_4_Na [M + Na]^+^: 444.1894, found: 444.1899.

### *N*-(2-(4-(Adamantane-1-carbonyl)piperazin-1-yl)-2-oxoethyl)-3-(4-nitrophenyl)propiolamide (38)

Compound 37, which was synthesized as previously described,^20^ (0.10 g, 0.33 mmol, 1.0 eq.) and 34 (0.07 g, 0.36 mmol, 1.1 eq.) were stirred in anhydrous DCM (2 mL) under N_2_ at 0 °C. A solution of DCC (0.07 g, 0.36 mmol, 1.1 eq.) and DMAP (0.001 g, 0.008 mmol, 2 mol%) in anhydrous DCM (2 mL) was added, the ice bath was removed and the reaction was stirred for 3 h. The mixture was filtered through celite and the filtrate was diluted with EtOAc (50 mL). The organic phase was washed with 5% AcOH (3 × 15 mL), brine (25 mL), NaHCO_3_ (25 mL), and brine again (25 mL). The organic phase was dried over MgSO_4_ and concentrated *in vacuo* to give a yellow solid, which was purified by silica gel column chromatography (8% MeOH/DCM, *R*_f_ = 0.42, dry loading using celite) (0.14 g, 88%). ^**1**^**H NMR** (300 MHz, CDCl_3_) *δ* 8.33–8.25 (d, *J* = 9.0 Hz, 2H), 7.78–7.72 (d, *J* = 9.0 Hz, 2H), 4.27–4.19 (d, *J* = 3.0 Hz, 2H), 3.87–3.66 (m, 6H), 3.57–3.39 (m, 2H), 2.15–2.08 (m, 3H), 2.06–2.00 (m, 6H), 1.86–1.67 (m, 6H) ^**13**^**C NMR** (75 MHz, CDCl_3_) *δ* 176.12, 165.7, 152.2, 148.3, 133.4, 126.7, 123.7, 86.2, 82.5, 45.2, 44.6, 41.8, 41.6, 39.1, 36.5, 28.4. **HRMS (ESI)** calc'd for C_26_H_30_N_4_O_5_Na [M + Na]^+^: 501.2126, found: 501.2114.

### 1-(4-(4-Nitrobenzyl)piperazin-1-yl)-3-(4-nitrophenyl)prop-2-yn-1-one (40)

39, which was synthesized as previously described,^56^ (0.05 g, 0.25 mmol, 1.1 eq.), 34 (0.06 g, 0.23 mmol, 1.0 eq.), EDCI-HCl (0.05 g, 0.27 mmol, 1.2 eq.) and HOBt·H_2_O (0.04 g, 0.27 mmol, 1.2 eq.) were stirred in dry DCM (2 mL) under N_2_. After 24 h, the reaction mixture was diluted with DCM (15 mL) and washed with H_2_O (2 × 15 mL), followed by brine (15 mL). The organic layer was then dried over MgSO_4_ and concentrated *in vacuo*, to give a white solid, which was purified by silica gel column chromatography, giving a white solid (5% DCM/EtOAc, *R*_f_ = 0.52, dry loading using celite) (0.02 g, 20%). ^**1**^**H NMR** (300 MHz, CDCl_3_) *δ* 8.28–8.15 (m, 4H), 7.74–7.64 (d, *J* = 9 Hz, 2H), 7.62–7.43 (d, *J* = 9 Hz, 2H), 3.95–3.79 (m, 2H), 3.79–3.69 (m, 2H), 3.69–3.54 (s, 2H), 2.70–2.37 (m, 4H). ^**13**^**C NMR** (75 MHz, CDCl_3_) *δ* 152.1, 148.2, 147.4, 145.5, 133.1, 129.5, 127.0, 123.8, 88.1, 84.8, 61.8, 52.4, 47.0, 41.6. **HRMS (ESI)** calc'd for C_20_H_18_N_4_O_5_ [M + H]^+^: 395.1357, found: 395.1387.

### TG2 inhibition assay

Recombinant TG2 was expressed from *E. coli* and purified as described previously.^57^ TG2 activity was determined according to a previously published colorimetric activity assay using the chromogenic substrate Cbz-Glu(γ-pnitrophenylester)Gly (AL5).^58^ In order to determine irreversible inhibition parameters for each inhibitor, enzymatic assays were run under Kitz and Wilson conditions,^25,26^ in the presence of 100 μM AL5 substrate, in triplicate. A large excess of substrate was used to ensure that any curvature in the slope is not attributed to depletion of the substrate, but rather to time-dependent inhibition. Buffered solutions of 50 mM of 3-(4-morpholino)propanesulfonic acid (MOPS) (pH 6.9), 7.5 mM CaCl_2_, 100 μM AL5, and various concentrations of inhibitor (from 0.25 to 1200 μM, depending on the inhibitor) were prepared in a 96-well polystyrene microplate with a final volume of 200 μL at 25 °C. AL5 and inhibitor stocks were prepared in DMSO ensuring that the final concentration of this co-solvent did not exceed 10% v/v. To initiate the enzymatic reaction, 5 mU mL^−1^ (0.25 μM) TG2, or water for the blank, was added to the well and the formation of the hydrolysis product, *p*-nitrophenolate, was followed at 405 nm for 20 min (a period of time over which the positive control is linear and not being impacted by substrate depletion) using a BioTek Synergy 4 plate reader. Background AL5 hydrolysis was corrected for by subtracting the blank from each reaction. Observed first order rate constants of inactivation (*k*_obs_) were obtained by fitting the inhibition data sets by non-linear regression to mono-exponential association eqn (1) using GraphPad Prism software.

1 Abs_*t*_ = Abs_max_(1 − e^−*k*_obs_*t*^)

The rate constants measured at different inhibitor concentrations were then fitted by non-linear regression to a saturation kinetics model, using eqn (2).2
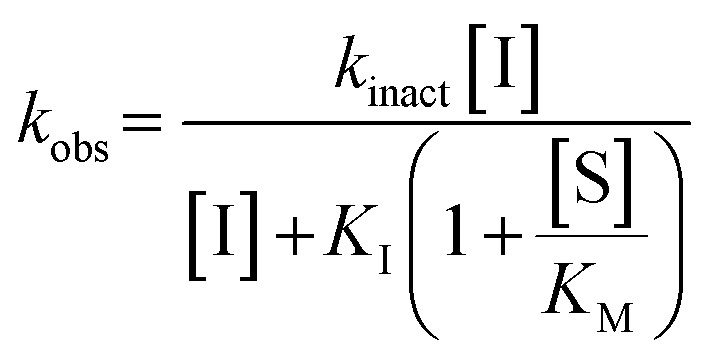


In order to correct for the competition with the assay substrate, AL5, the inhibitor concentrations were divided by *α*, which equals (1 + ([S]/*K*_M_)), where *K*_M_ = 10 μM. Inhibition parameters, *k*_inact_ and *K*_I_, were then extrapolated from the fitting.

In cases where a saturation plot could not be generated due to solubility constraints and only a few inhibitor concentrations tested, a double-reciprocal plot of (eqn (2)) was made to estimate *k*_inact_ and *K*_I_ according to eqn (3).3
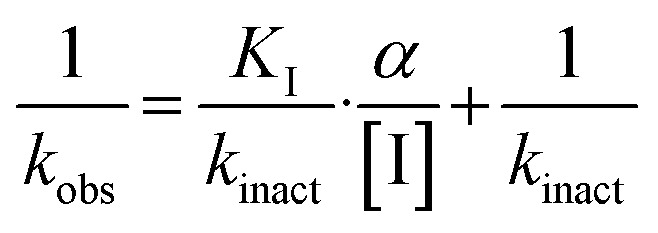


Simple linear regression was performed on the *k*_obs_ values measured at the lowest concentrations of each inhibitor for validation of *k*_inact_/*K*_I_ ratios.

For reversible inhibitor 16, initial rates of inhibition (*v*_i_) were measured at various inhibitor concentrations instead of *k*_obs_ values. These rates were then normalized with respect to the uninhibited control (% *v*_i_/*v*_0_). These percent relative rate values were plotted against the inhibitor concentration on a logarithmic scale to generate a IC_50_ curve. An IC_50_ value was determined using four-parameter fitting eqn (4) in GraphPad Prism 9.4
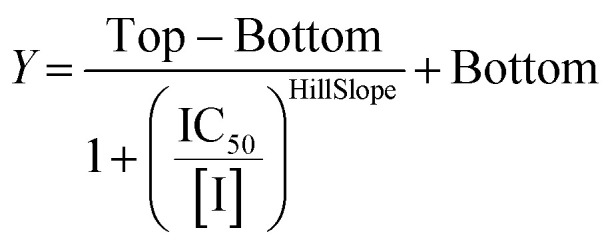


An estimated *K*_i_ value was then calculated using the Cheng–Prusoff eqn (5)^59^5
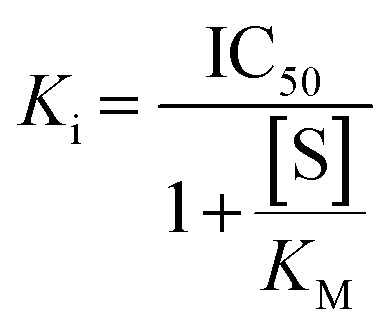


### Isozyme selectivity

First, for each inhibitor, a single corrected inhibitor concentration ([I]/*α*) was chosen, which would result in the complete inactivation of TG2 in under 5 min. Then for each isozyme, the concentration of inhibitor was adjusted according to the value of *α* determined by the concentration and *K*_M_ value of the substrate of each isozyme assay. In this way the effective inhibitor concentration ([I]/*α*) remained constant for each isozyme.^20^

The activities of TG1 and TG6 were measured by a colorimetric assay using the substrate AL5. Assays were performed as previously described for TG2 under Kitz & Wilson conditions established for each transglutaminase isoform by varying the concentration of substrate to 112 μM and 436 μM of AL5 for TG1 and TG6, respectively. The reaction was initiated with the addition of enzyme, 0.10 μM TG1 or 0.32 μM TG6. Formation of the hydrolysis product, *p*-nitrophenolate, was followed at 405 nm for 100 min for TG1 or 60 min for TG6.^16^

The isopeptidase activities of activated TG3a and hFXIIIa (purchased from Zedira) were measured by a fluorescence-based assay based on the use of the peptidic FRET-quenched probe A101 (Zedira).^16,32^ The final assay mixture comprised 50 mM TRIS (pH 7.0), 10 mM CaCl_2_, 100 mM NaCl, 2.8 mM TCEP, 50 μM A101 and 14 mM H-Gly-OMe. The reaction was monitored at 25 °C using a BioTek Synergy 4 plate reader (Ex/Em: 318/413 nm). Enzymatic inhibition assays were run under Kitz and Wilson conditions, which were established for TG3a and FXIIIa at a substrate (A101) concentration of 50 μM using enzyme concentrations of 0.17 μM and 0.11 μM for TG3a and FXIIIa, respectively. Experiments were completed at least in triplicate. A *k*_obs_ value was obtained for each isozyme as described above.

### GTP binding assay

GTP binding experiments were performed following a previously described protocol.^34^ hTG2 (20 μg) was incubated at 25 °C for 30 min with or without an irreversible inhibitor (each at a concentration of 2 × *K*_I_) with 15 mM CaCl_2_ in 100 mM MOPS (pH = 6.91). The buffer was then exchanged *via* dialysis to 100 mM MOPS (pH = 7.0), 1 mM EGTA, and 5 mM MgCl_2_ to remove calcium using a 14-kDa molecular weight cut-off membrane cuvette (purchased from Millipore-Sigma, Oakville, ON, Canada). The fluorescent, nonhydrolyzable GTP analogue BODIPY GTP-γ-S (purchased from Invitrogen, Waltham, MA, USA), whose fluorescence increases when bound to protein, was then added at a final concentration of 0.5 μM. Fluorescence was then measured on a microplate reader after 10 min of incubation (Ex/Em: 490/520 nm).

### Intrinsic reactivity

HPLC traces were collected by Gilson-Mandel GXP271 high performance liquid chromatography (HPLC) with UV detection at 214 and 254 nm (Phenomenex Luna, 150 mm × 4.6 mm, 30 min, 1.5 mL min^−1^ flow rate, 5–95% CH_3_CN with 0.1% TFA in H_2_O with 0.1% TFA, 30 min method).

Intrinsic reactions were monitored with a 10-fold excess of GSH for compounds 8N, 18, 23B, 23C, 23E and a 100-fold excess for compounds 13 and 23D, depending on the stability of the warhead. In 2 mL HPLC vials fitted with a pre-slit screw cap, 1250 μL of aqueous buffer was added (pH = 7.4, phosphate buffer for 8N, 16, 18, 23B, 23C, 23E and pH = 10.4 CAPS buffer for 13 and 23D, buffer stability over 24 h was confirmed in all cases). Then, 150 μL of a 25- or 250-mM stock solution of GSH in the same buffer was added. The reactions were initiated by the addition of 150 μL of 2.5-mM warhead stock solution in DMSO to a final concentration of 0.25 mM with 10% v/v DMSO as co-solvent. The final concentration of GSH was either 2.5 or 25 mM. Once initiated, the HPLC vials were inverted, placed on the autosampler tray and an initial aliquot was taken immediately. The vials were incubated at 22 °C with aliquots taken at pre-determined time points over the course of at least 5–6 half lives. The disappearance or decrease in the area under the curve (AUC) of the chromatogram of each compound was measured in triplicate.

The decreasing AUC of the inhibitor peaks of the HPLC chromatograms were fitted to a mono-exponential decay model, with the constraint that the lower plateau = 0 (eqn (6)).

6 AUC = AUC_0_(e^−*k*_obs_*t*^)

Second order rates constants were then calculated by dividing these pseudo-first order rate constants by the concentration of excess thiol (eqn (7)).7
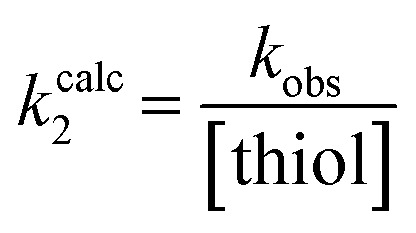


The final, corrected second order rate constants were then calculated by dividing by the fraction of thiolate at the reaction pH, using the corresponding p*K*_α_ value (8.7) of the thiol (eqn (8)).^35^8
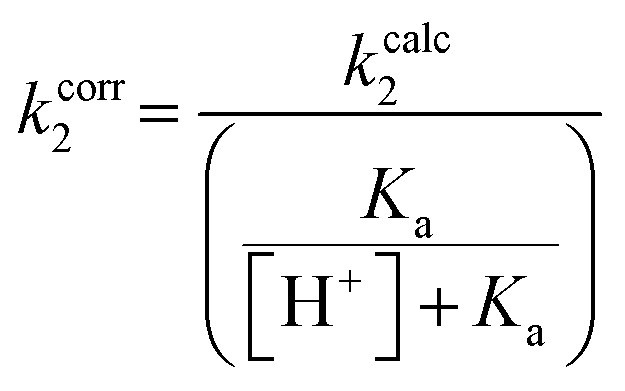


For compound 16 (12 mM), due to limited aqueous solubility, a stability half-life (*t*_½_) with 80 mM GSH was determined by ^1^H NMR in DMSO-*d*_6_.^23^

### Molecular docking in molecular operating software (MOE)

Two crystals were selected as a target receptor enzyme, and were imported from their PDB files, namely 2Q3Z and 3S3S. The structure was prepared using the preparation tool from MOE. First, water molecules, salts and ions were removed from the structure. All hydrogen atoms were then added (electrostatics: 1/*r*^2^, dielectric: 2, solvent: 80, van der Waals: 12–6) and the protein structures were finally verified, and corrected manually, for any problems or warnings such as chain breaks, termini missing or unreasonable charges. Ligands were drawn in ChemDraw and imported to MOE; partial charges were calculated using a MMFF94x forcefield and the system was eventually minimized following a 0.0001 kcal mol^−1^ Å^−2^ gradient. After solvation, minimization was repeated.

The “compute” tool from MOE was used to perform docking analysis of each ligand by a *non-covalent* approach. Ligand placement was achieved using the Triangle Matcher protocol (London dG) to produce 30 poses. In addition, a Rigid Receptor refinement protocol was performed (GBVI/WSA dG) and a total of 5 to 15 final poses were obtained. Finally, the top pose (lowest S-score) was chosen and using the builder tool from MOE, the covalent bond between residue CYS277 and the warhead of the bound inhibitors were manually created, prior to minimization of the system (0.001 kcal mol^−1^ Å^−2^).

## Author contributions

Conceptualization: J. W. K. and L. K. M.; funding acquisition: J. W. K.; supervision: J. W. K.; investigation and methodology: L. K. M., N. M., J. E. B.; writing – original draft: L. K. M., writing – reviewing and editing: all authors.

## Conflicts of interest

There are no conflicts of interest to declare.

## List of abbreviations

AcOH: Acetic acid
Ad: Adamantane
Boc: *tert*-Butyloxycarbonyl
Cys: Cysteine
DCC: *N*,*N*′-Dicyclohexylcarbodiimide
DCM: Dichloromethane
DIPEA: Diisopropylethylamine
DMAP: 4-Dimethylaminopyridine
DMF: Dimethylformamide
DMP: Dess–Martin Periodinane
DPPA: Diphenylphosphoryl azide
EDCI: 1-Ethyl-3-(3-dimethylaminopropyl)carbodiimide
Et_2_O: Diethyl ether
EtOAc: Ethyl acetate
EtOH: Ethanol
HBTU: *N*,*N*,*N*′,*N*′-Tetramethyl-*O*-(1*H*-benzotriazol-1-yl)uronium hexafluorophosphate
HOBt: Hydroxybenzotriazole
MeCN: Acetonitrile
MeOH: Methanol
Na Asc: Sodium Ascorbate
NEt_3_: Triethylamine
NMI: *N*-Methyl imidazole
R.T.: Room temperature
*t*BuOH: *tert*-butanol
TCFH: *N*′-tetramethylformamidinium hexafluorophosphate
TCICA: Trichloroisocyanuric acid
TEMPO: 2,2,6,6-Tetramethylpiperidinyloxy
TFA: Trifluoroacetic acid
THF: Tetrahydrofuran
THP: Tetrahydropyranyl

## Supplementary Material

MD-OLF-D5MD00777A-s001

## Data Availability

The data supporting this article (kinetic graphs, HPLC traces, and NMR spectra) have been included as part of the supplementary information (SI). Supplementary information is available. See DOI: https://doi.org/10.1039/d5md00777a.
