# Supplementary material for: Discovery of an internal alkyne warhead scaffold for irreversible hTG2 inhibition
Source: RSC Med Chem. 2025 Oct 9;16(12):6228–61. doi: 10.1039/d5md00777a (PMC12560834; doi:10.1039/d5md00777a)

Supplementary Information (SI) for RSC Medicinal Chemistry.
This journal is © The Royal Society of Chemistry 2025

Electronic Supplementary Information

for

Discovery of an Internal Alkyne Warhead Scaffold for Irreversible hTG2 Inhibition

Lavleen K. Mader, Namita Maunick, Jessica E. Borean, Jeffrey W. Keillor*

Department of Chemistry and Biomolecular Sciences, University of Ottawa, Ottawa, Ontario K1N 6N5, Canada.

*Corresponding author: jkeillor@uottawa.ca

Table of Contents

hTG2 Inhibition Kinetics Data **S1**
Isozyme Selectivity Data **S13**
Intrinsic Reactivity Data **S14**
HPLC Purity Analysis of Final Inhibitors **S17**
NMR Spectra of Final Inhibitors and New Synthetic Intermediates **S25**

hTG2 Inhibition Kinetics Data:

8A

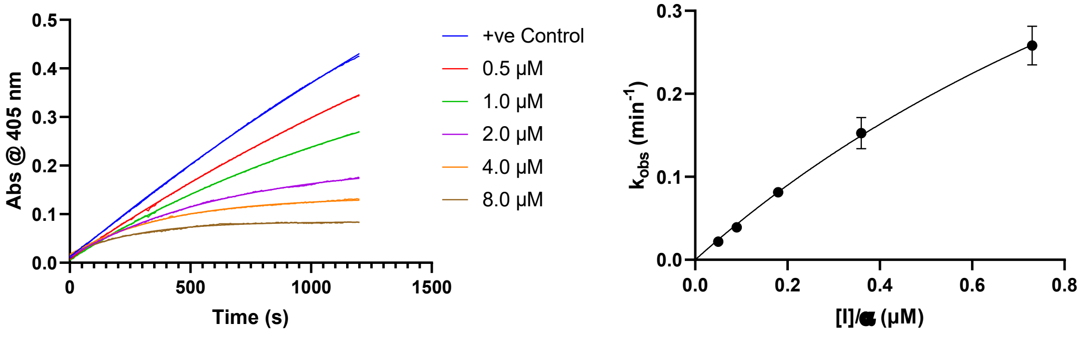

8B

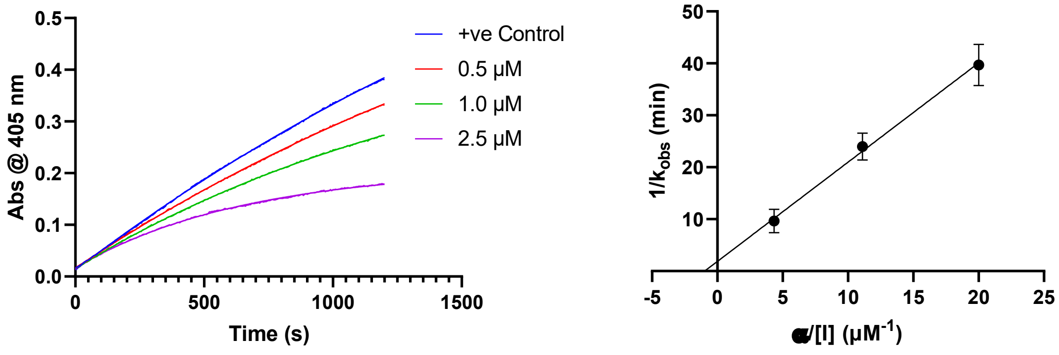

8C

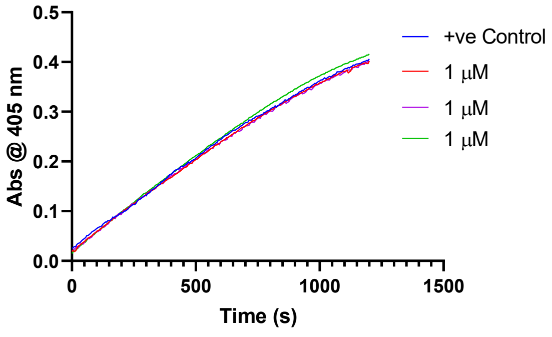

8D

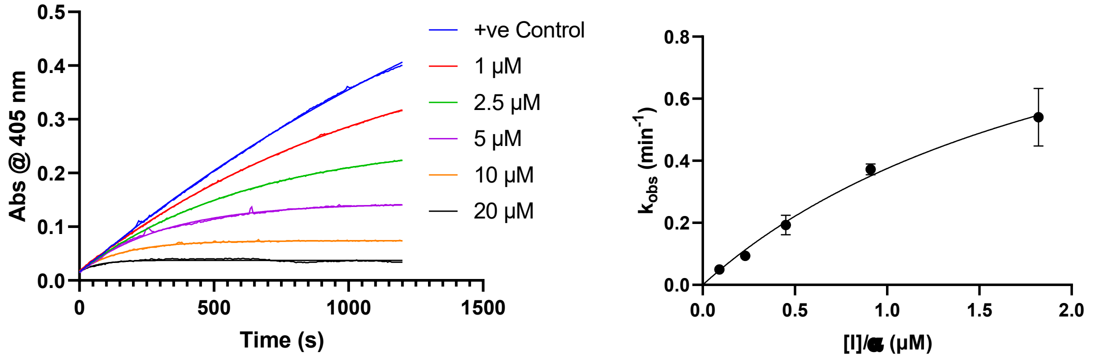
Abs @ 405 nm
Time (s)
+ve Control
1 μM
2.5 μM
5 μM
10 μM
20 μM
k_{obs} (min^{-1})

8E

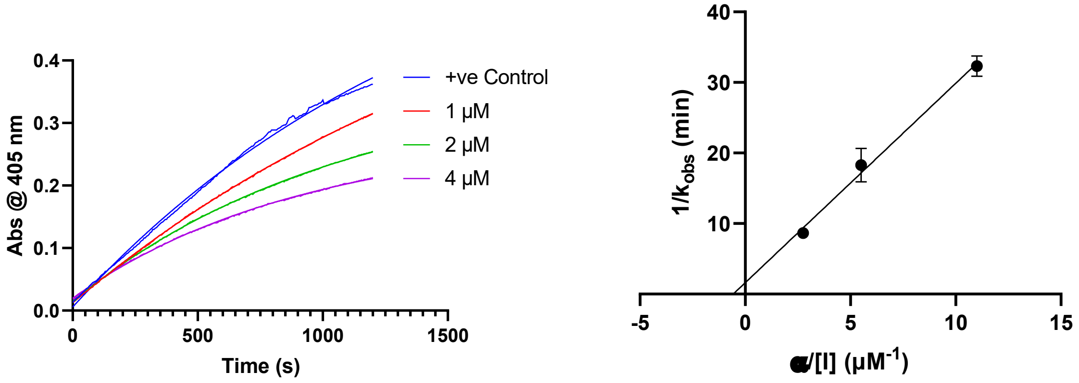
Abs @ 405 nm
Time (s)
+ve Control
1 μM
2 μM
4 μM
$1/k_{obs}$ (min)

8F

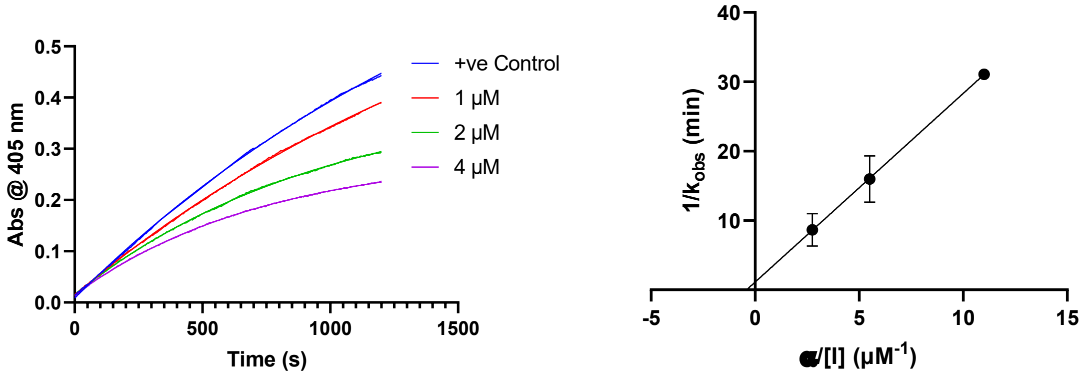
Abs @ 405 nm
Time (s)
+ve Control
1 μM
2 μM
4 μM
$1/k_{obs}$ (min)

8G

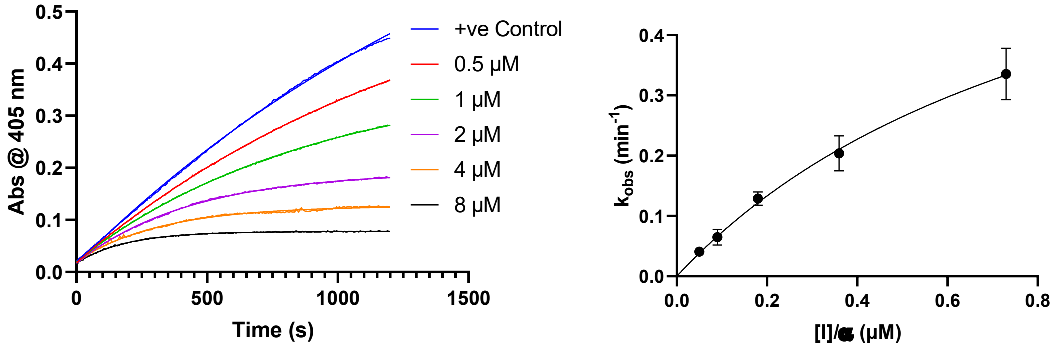

Abs @ 405 nm
Time (s)
+ve Control
0.5 µM
1 µM
2 µM
4 µM
8 µM
k_{obs} (min^{-1})

8H

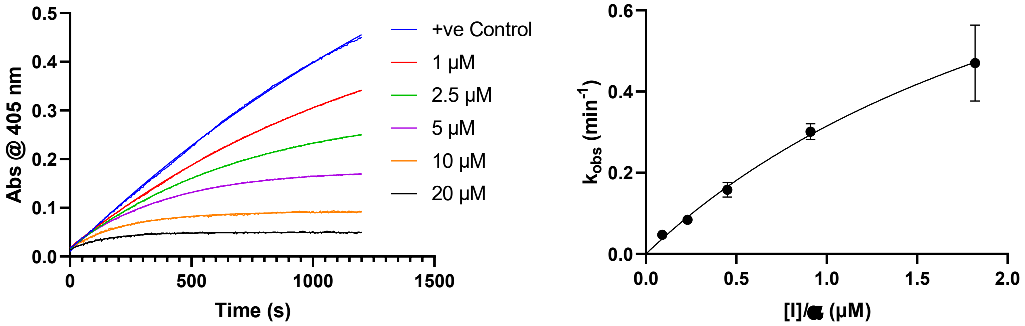

Abs @ 405 nm
Time (s)
+ve Control
1 µM
2.5 µM
5 µM
10 µM
20 µM
k_{obs} (min^{-1})

8I

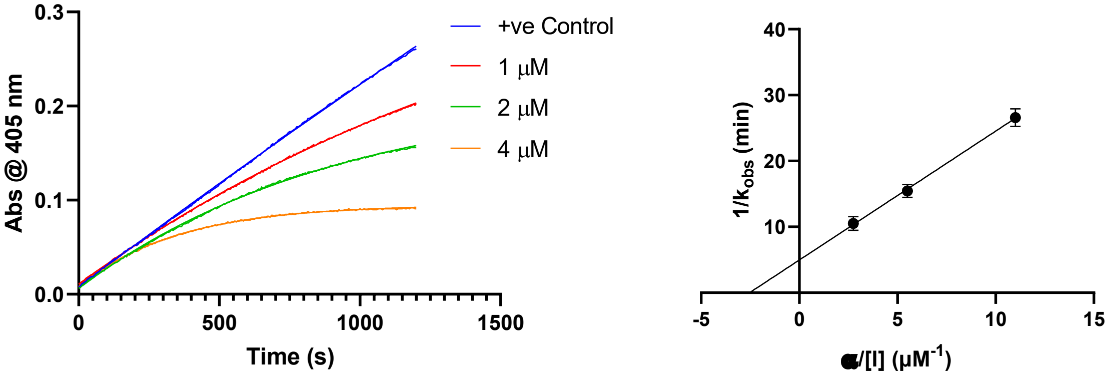

Abs @ 405 nm
Time (s)
+ve Control
1 µM
2 µM
4 µM
1/k_{obs} (min)

8J

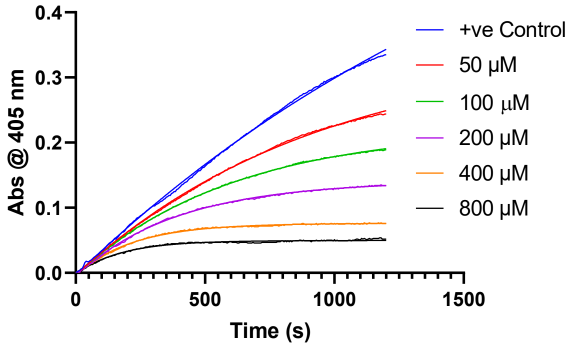
Abs @ 405 nm
Time (s)
+ve Control
50 µM
100 µM
200 µM
400 µM
800 µM

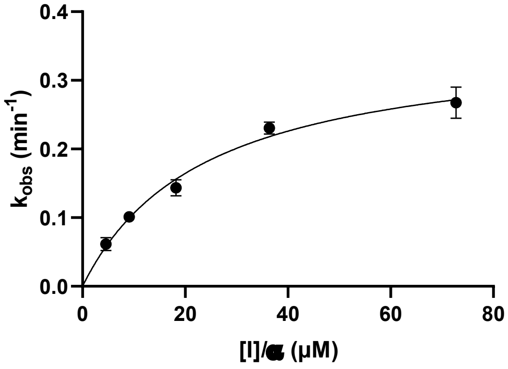
k_{obs} (min^{-1})
[I]/α (µM)

8K

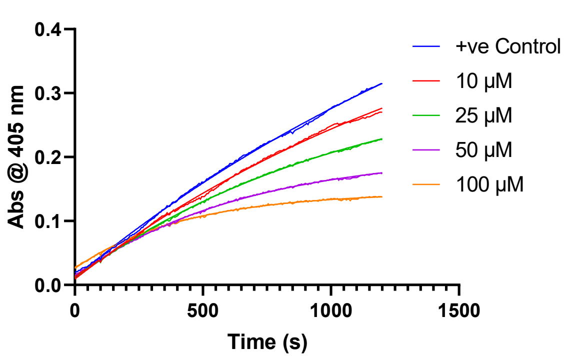
Abs @ 405 nm
Time (s)
+ve Control
10 µM
25 µM
50 µM
100 µM

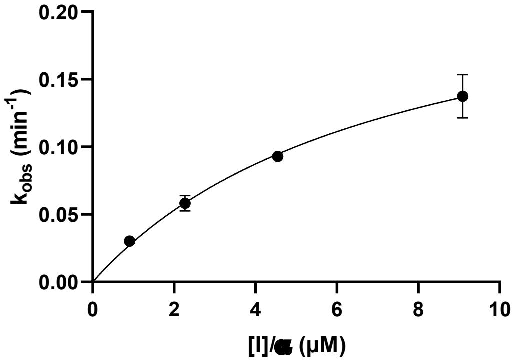
k_{obs} (min^{-1})
[I]/α (µM)

8L

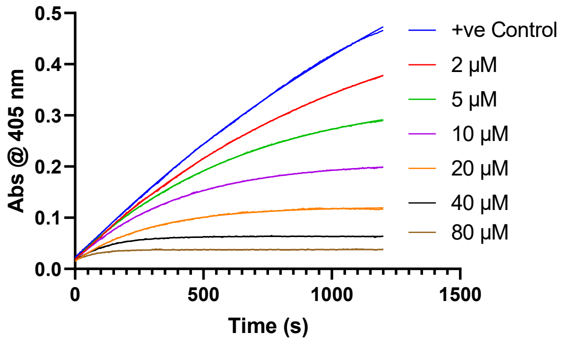
Abs @ 405 nm
Time (s)
+ve Control
2 µM
5 µM
10 µM
20 µM
40 µM
80 µM

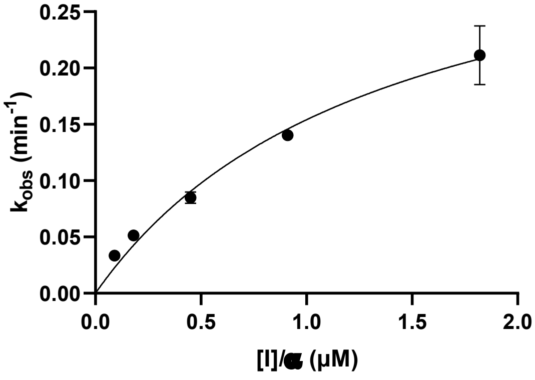
k_{obs} (min^{-1})
[I]/α (µM)

8M

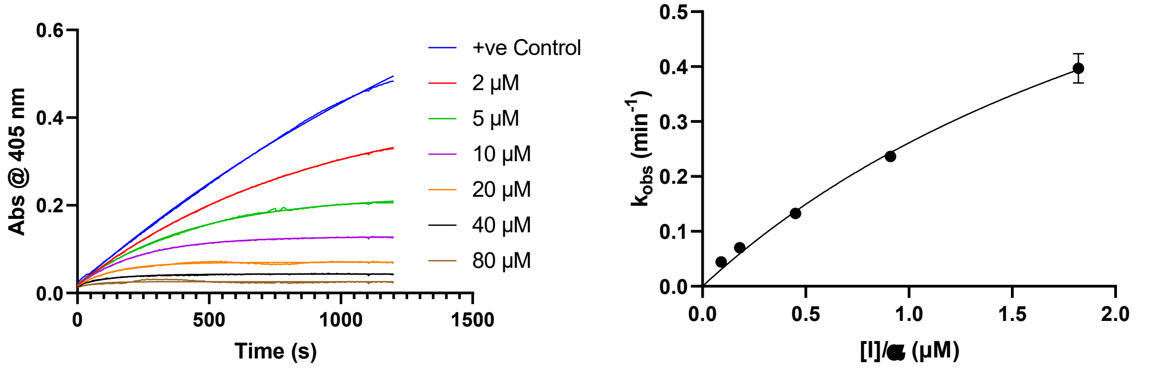
Abs @ 405 nm
Time (s)
+ve Control
2 μM
5 μM
10 μM
20 μM
40 μM
80 μM
k$_{obs}$ (min^{-1})
[I]/ (μM)

8N

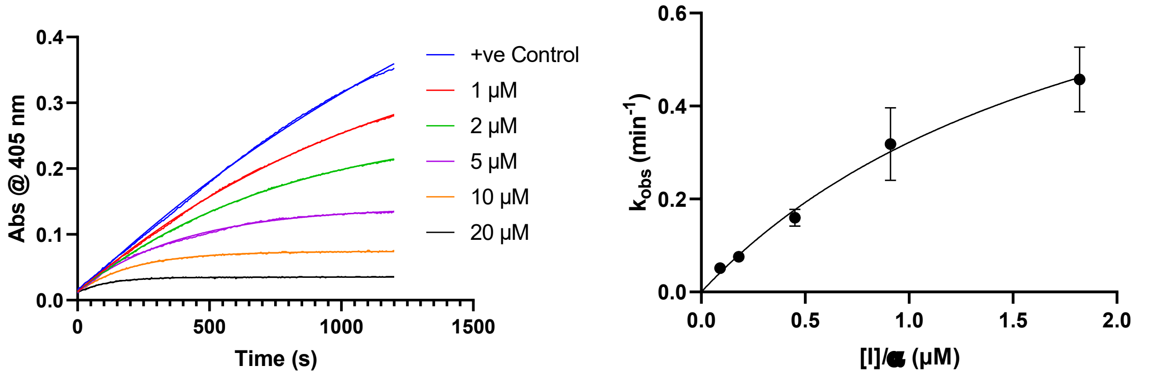
Abs @ 405 nm
Time (s)
+ve Control
1 μM
2 μM
5 μM
10 μM
20 μM
k$_{obs}$ (min^{-1})
[I]/ (μM)

8O

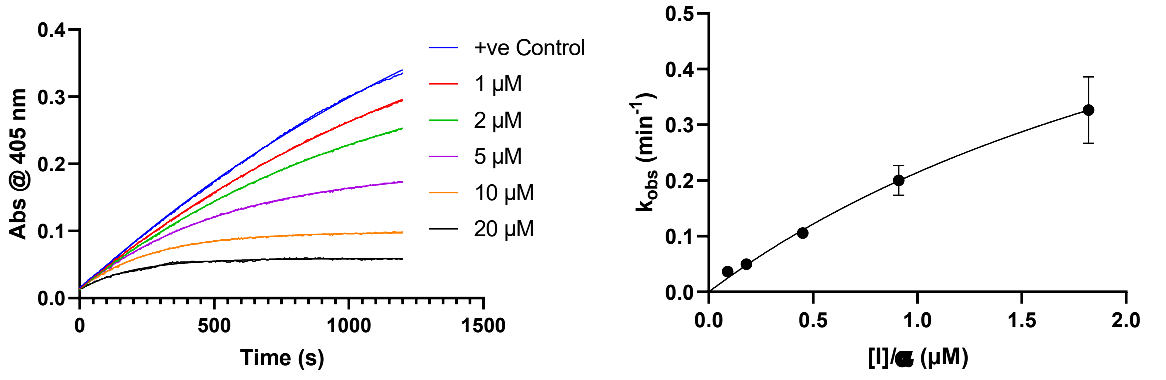
Abs @ 405 nm
Time (s)
+ve Control
1 μM
2 μM
5 μM
10 μM
20 μM
k$_{obs}$ (min^{-1})
[I]/ (μM)

8P

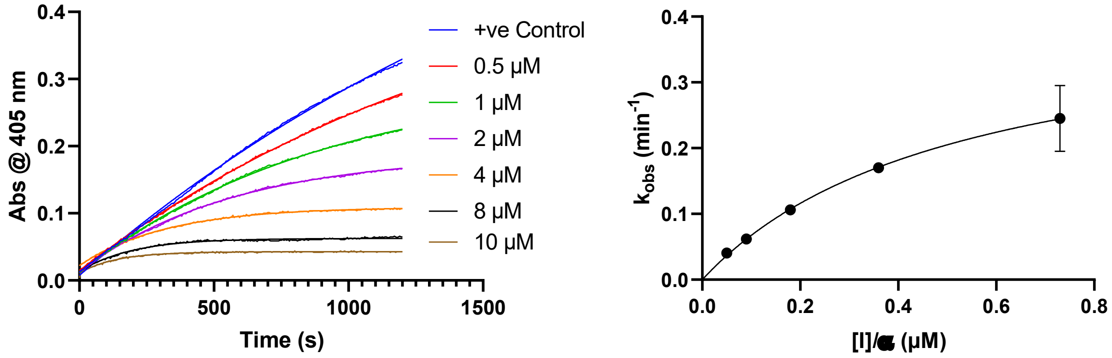
Abs @ 405 nm
Time (s)
+ve Control
0.5 μM
1 μM
2 μM
4 μM
8 μM
10 μM
k_{obs} (min^{-1})

8Q

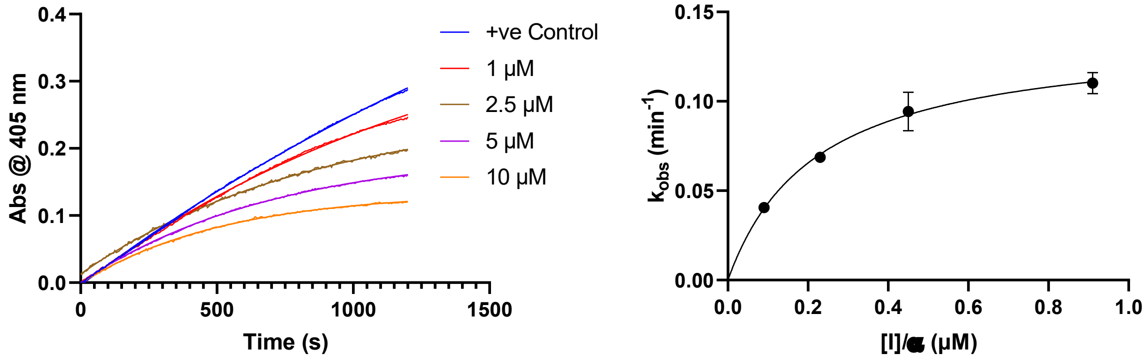
Abs @ 405 nm
Time (s)
+ve Control
1 μM
2.5 μM
5 μM
10 μM
k_{obs} (min^{-1})

8R

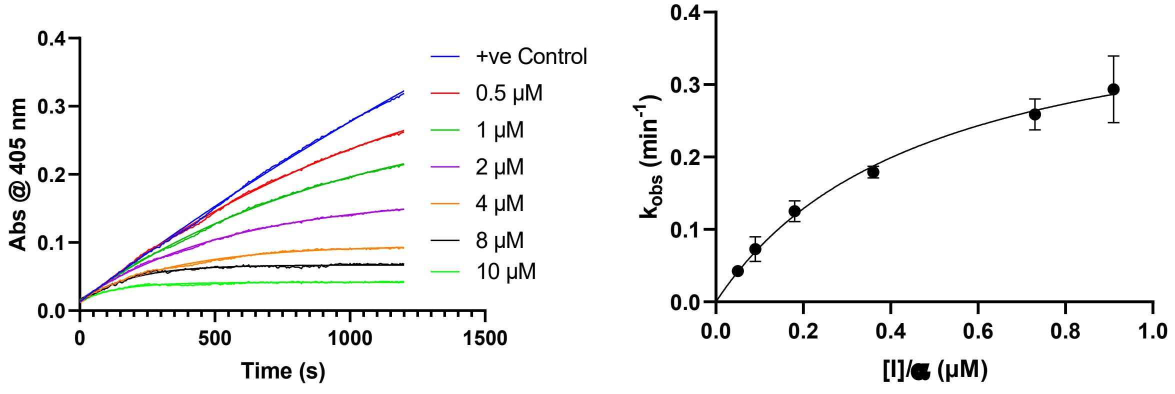
Abs @ 405 nm
Time (s)
+ve Control
0.5 μM
1 μM
2 μM
4 μM
8 μM
10 μM
k_{obs} (min^{-1})

12

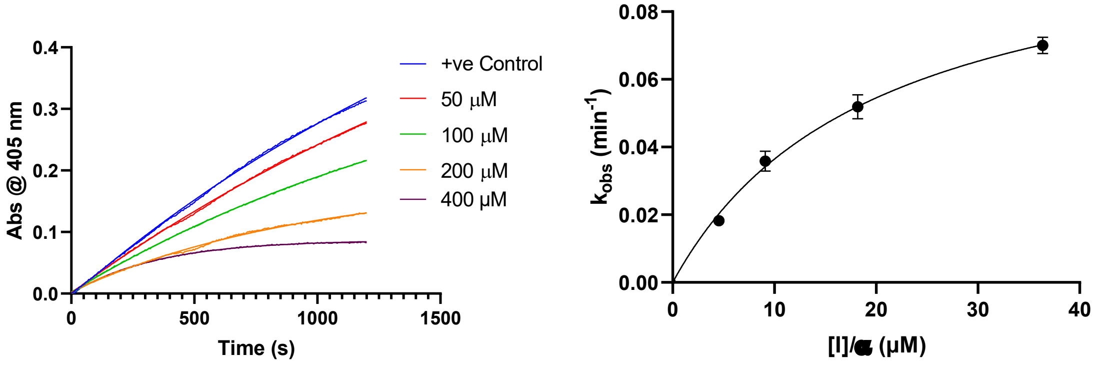

Abs @ 405 nm
Time (s)
+ve Control
50 μM
100 μM
200 μM
400 μM
k_{obs} (min^{-1})

13

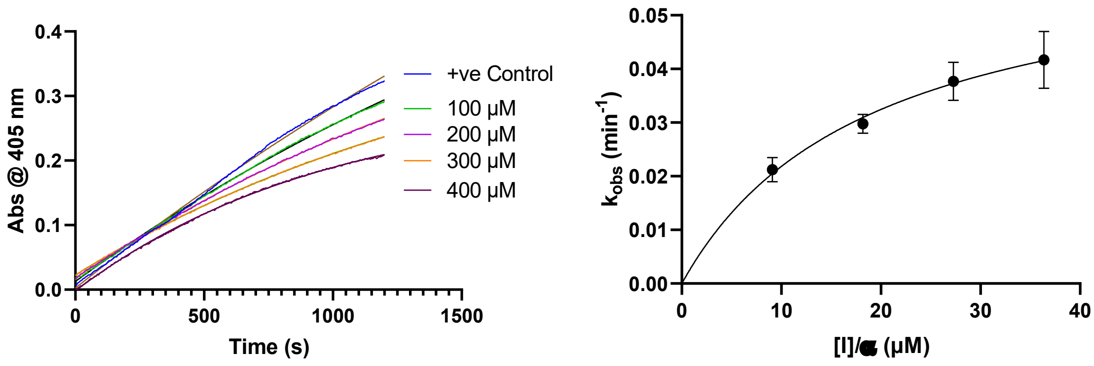

Abs @ 405 nm
Time (s)
+ve Control
100 μM
200 μM
300 μM
400 μM
k_{obs} (min^{-1})

16

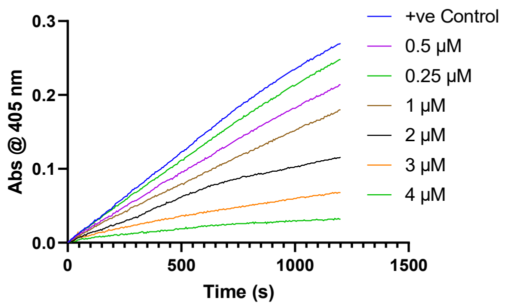

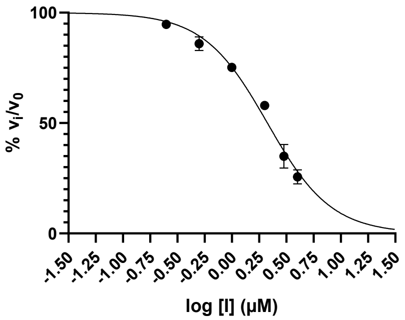

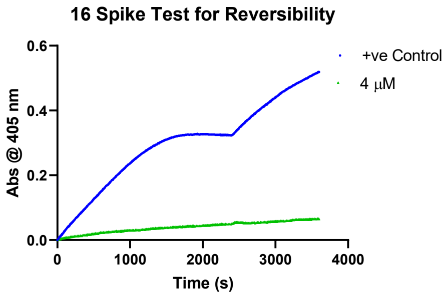

15 and Benzotriazole (To verify inhibitor 16)

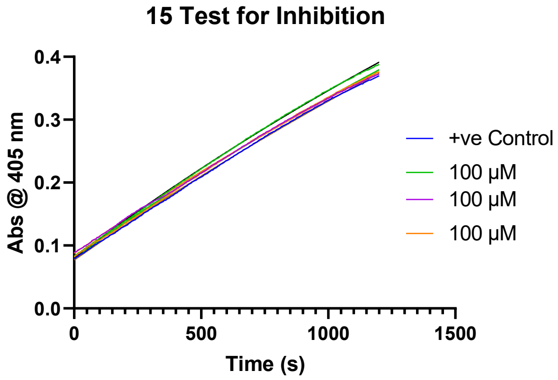

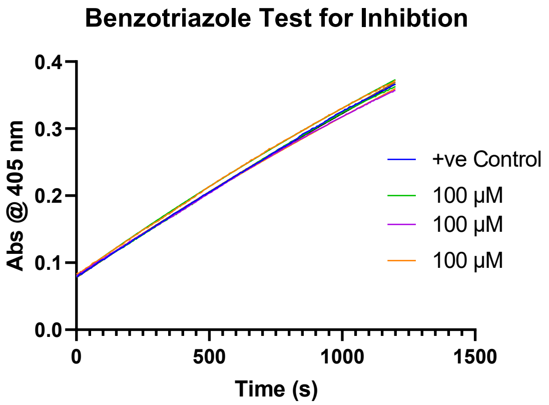

18

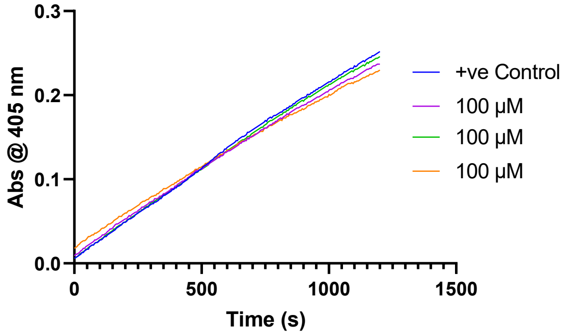
Abs @ 405 nm
Time (s)
0.0
0.1
0.2
0.3
0
500
1000
1500
+ve Control
100 μM
100 μM
100 μM

23A

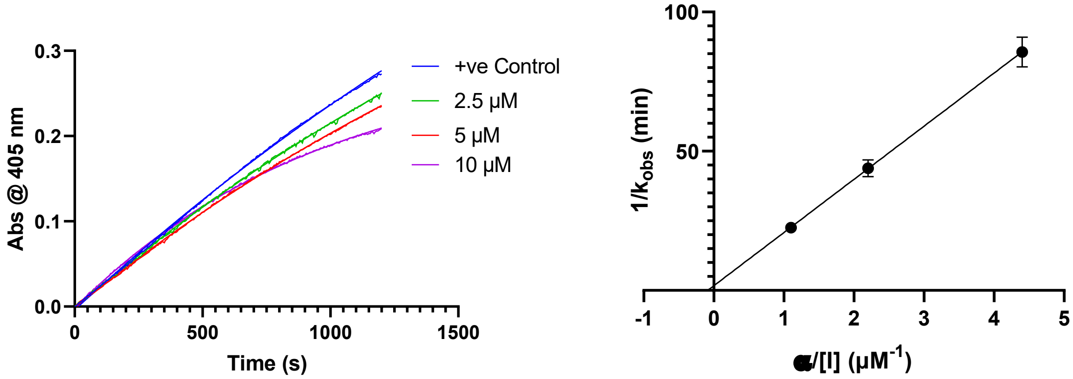
Abs @ 405 nm
Time (s)
0.0
0.1
0.2
0.3
0
500
1000
1500
+ve Control
2.5 μM
5 μM
10 μM
1/k_obs (min)
50
100
-1
0
1
2
3
4
5
/[I] (μM^-1)

23B

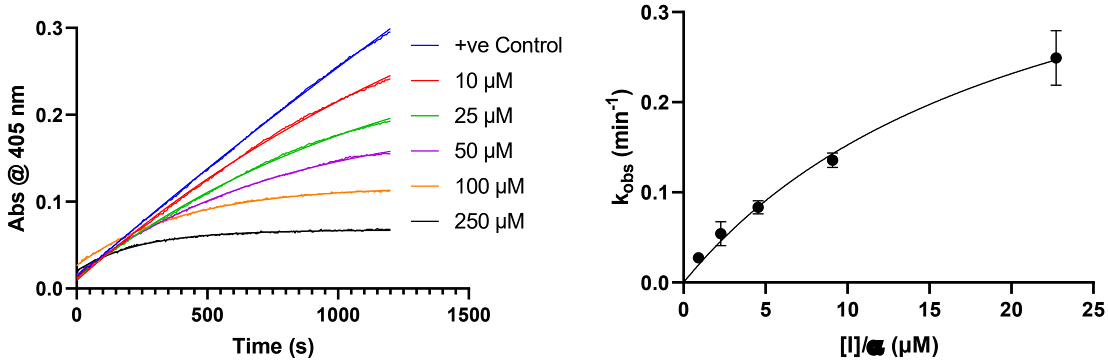
Abs @ 405 nm
Time (s)
0.0
0.1
0.2
0.3
0
500
1000
1500
+ve Control
10 μM
25 μM
50 μM
100 μM
250 μM
k_obs (min^-1)
0.0
0.1
0.2
0.3
0
5
10
15
20
25
[I]/ (μM)

28

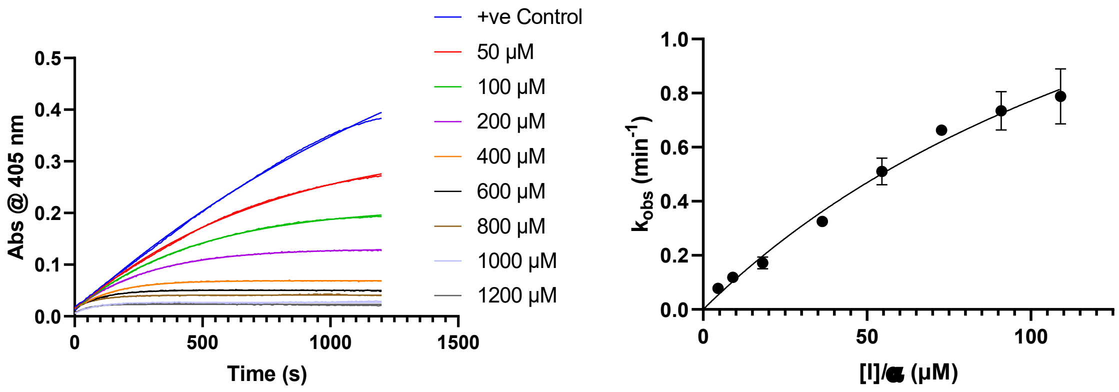

+ve Control
50 μM
100 μM
200 μM
400 μM
600 μM
800 μM
1000 μM
1200 μM
Abs @ 405 nm
Time (s)
kobs (min-1)

33

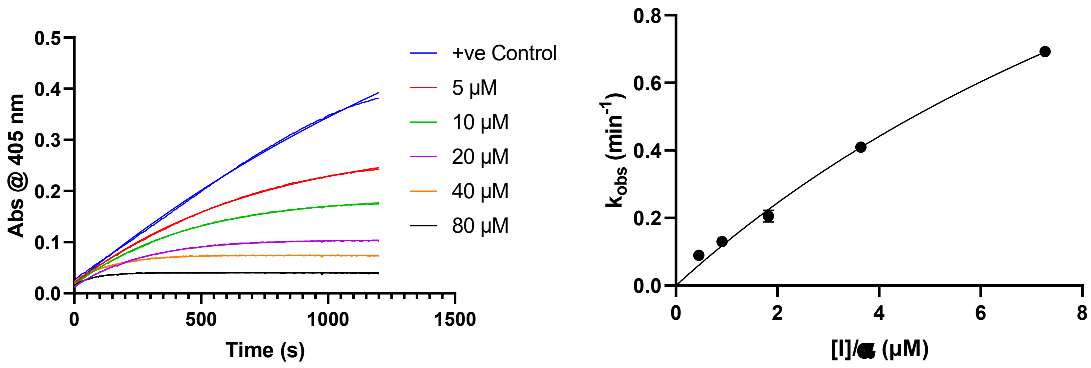

+ve Control
5 μM
10 μM
20 μM
40 μM
80 μM
Abs @ 405 nm
Time (s)
kobs (min-1)

36

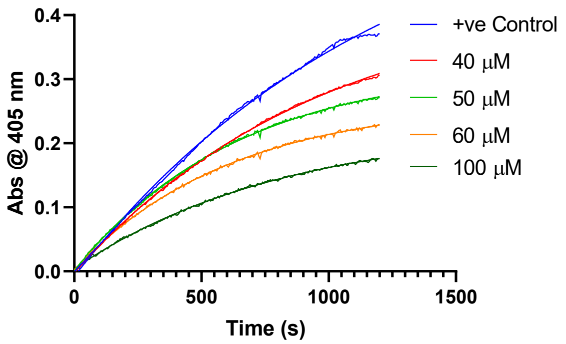
Abs @ 405 nm
0.0
0.1
0.2
0.3
0.4
0
500
1000
1500
Time (s)
+ve Control
40 μM
50 μM
60 μM
100 μM

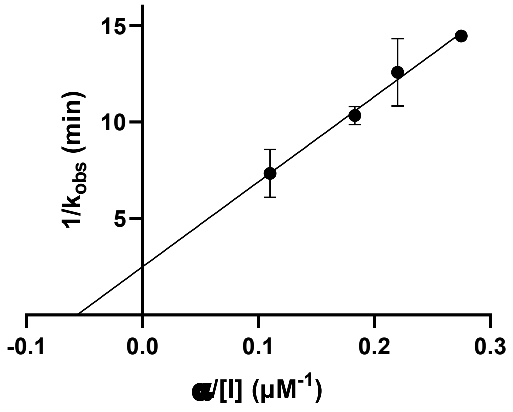
1/kobs (min)
5
10
15
-0.1
0.0
0.1
0.2
0.3
1/[I] (μM-1)

38

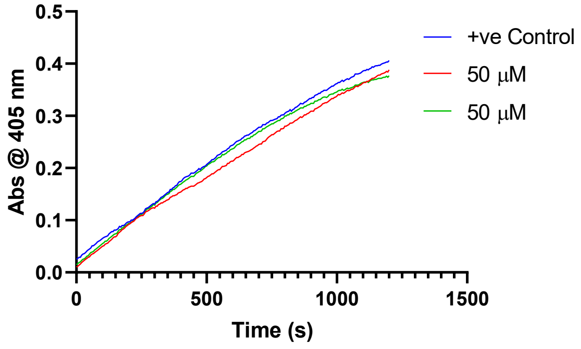
Abs @ 405 nm
0.0
0.1
0.2
0.3
0.4
0.5
0
500
1000
1500
Time (s)
+ve Control
50 μM
50 μM

40

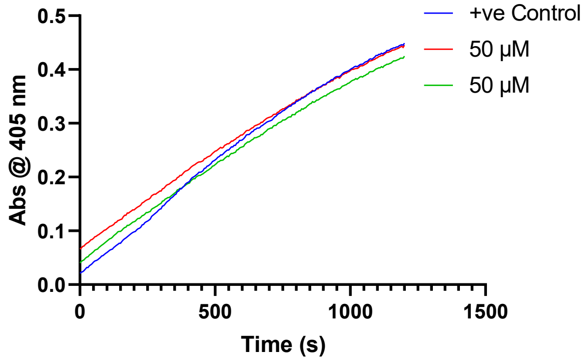
Abs @ 405 nm
0.0
0.1
0.2
0.3
0.4
0.5
0
500
1000
1500
Time (s)
+ve Control
50 μM
50 μM

Isozyme Selectivity of 8R:

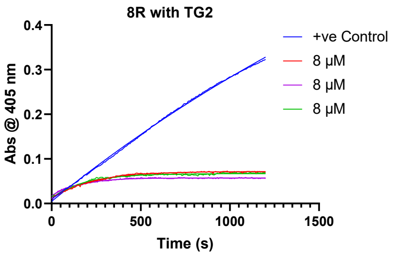

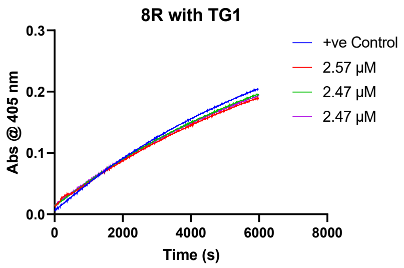

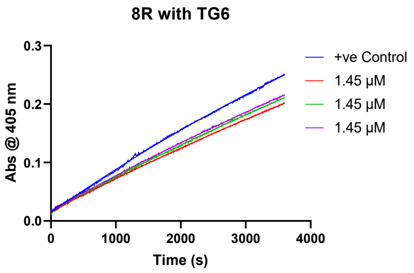

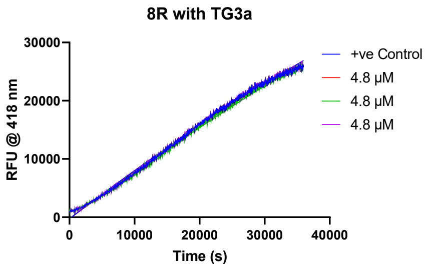

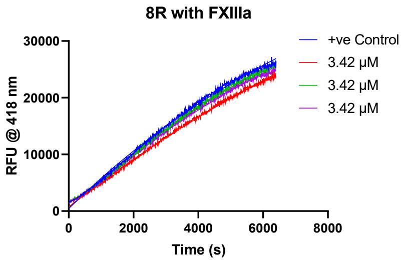

[8R]/α = 0.73 μM	***TG2***	***TG1***	***TG6***	***TG3***	***FXIIIa***
**k_{obs} (min^{-1})**	0.3332 ± 0.0497	n.d.	0.0040 ± 0.0001	n.d.	n.d.

n.d. : Not determined. Unable to measure a rate constant distinct from the no-inhibitor control.

Intrinsic Reactivity Data:

13

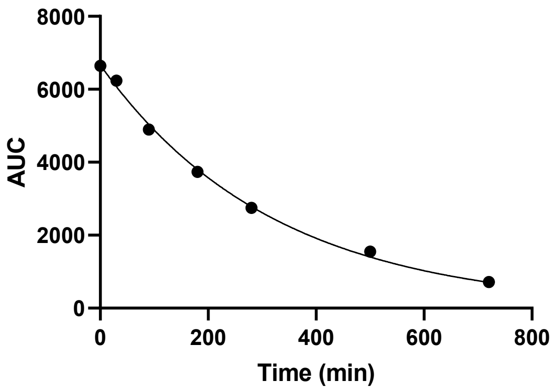

16

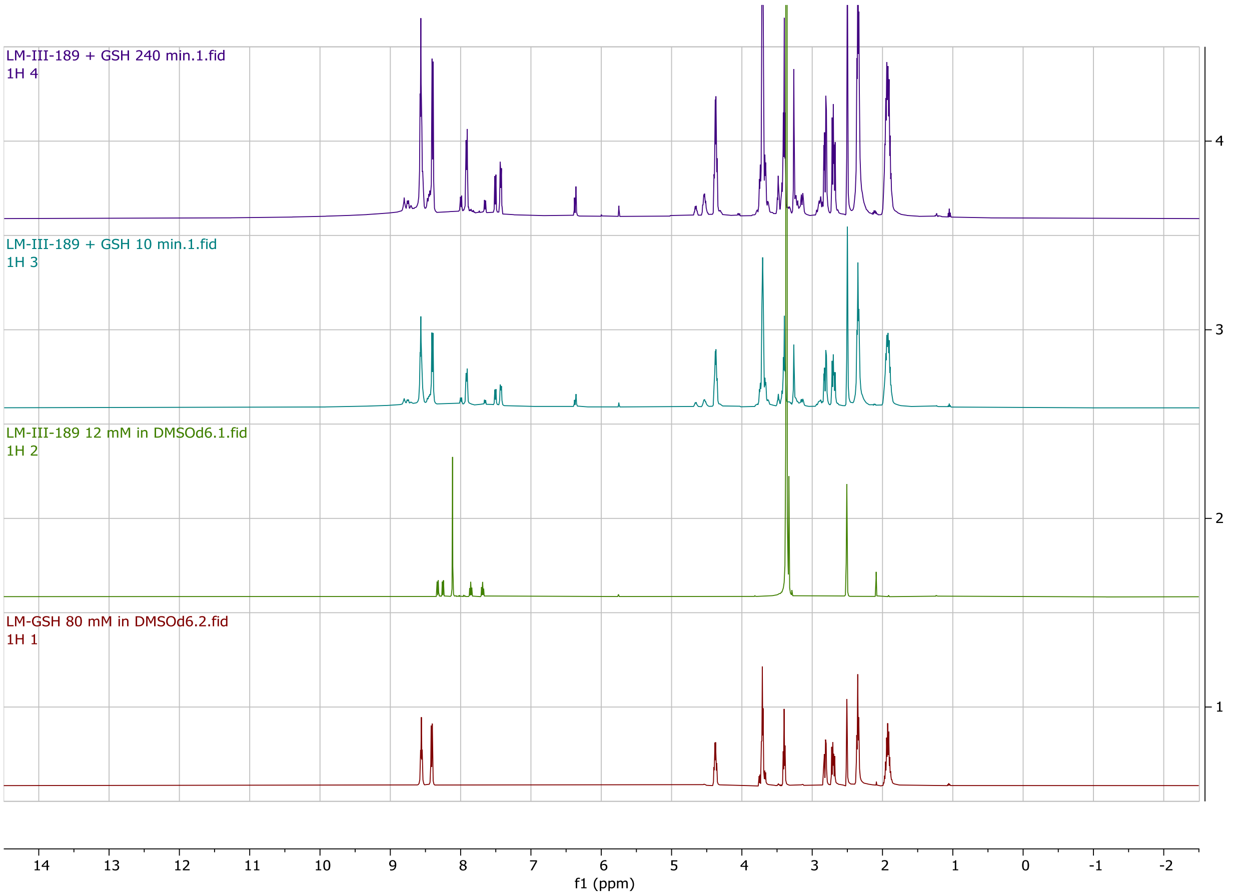

18

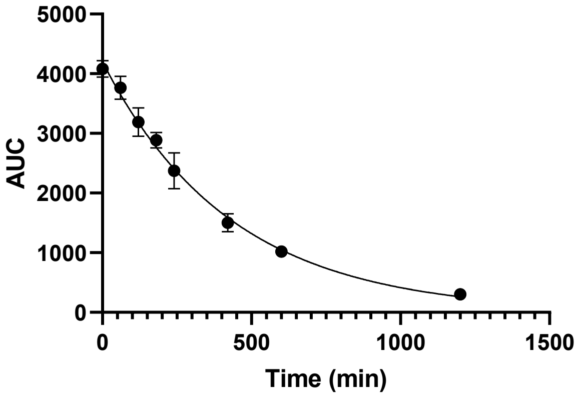
5000
4000
3000
2000
1000
0
AUC
0
500
1000
1500
Time (min)

23B

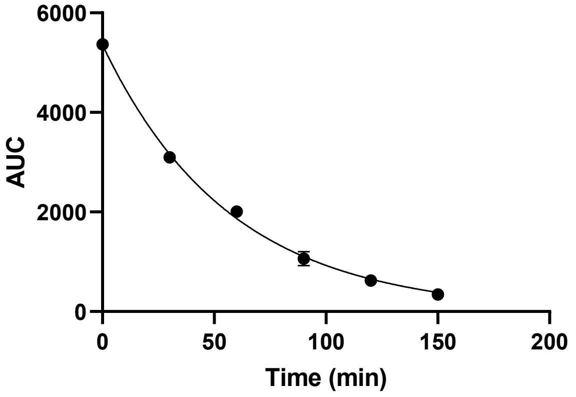
6000
4000
2000
0
AUC
0
50
100
150
200
Time (min)

23C

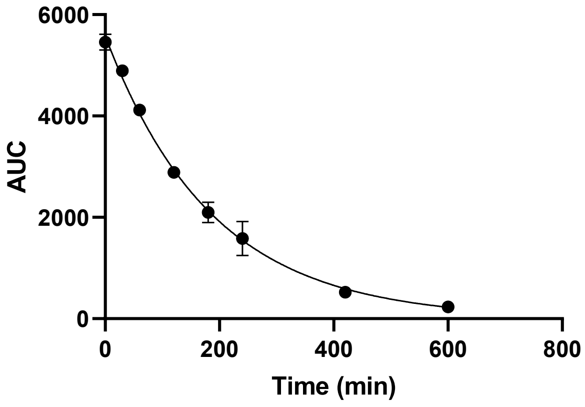
6000
4000
2000
0
AUC
0
200
400
600
800
Time (min)

23D

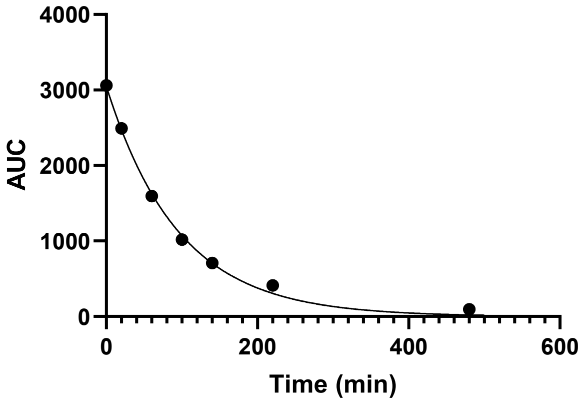

4000
3000
2000
1000
0
AUC
0
200
400
600
Time (min)

23E

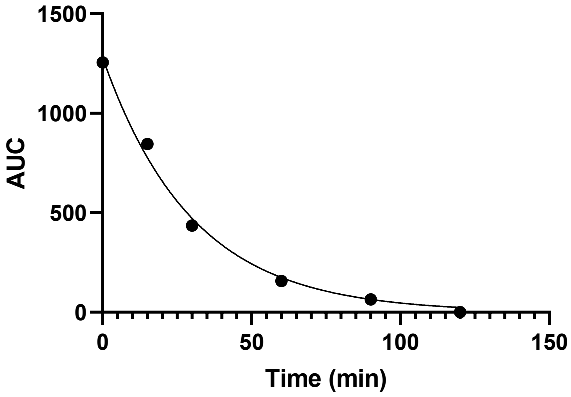

1500
1000
500
0
AUC
0
50
100
150
Time (min)

HPLC Purity Analysis of Final Compounds:

8A (99%)

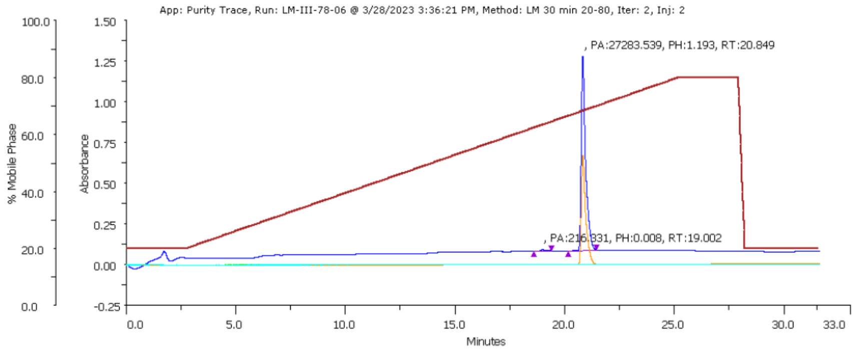

8B (99%)

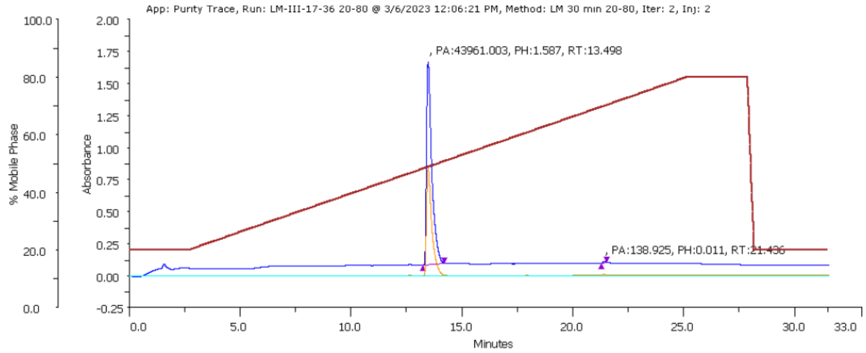

8C (98%)

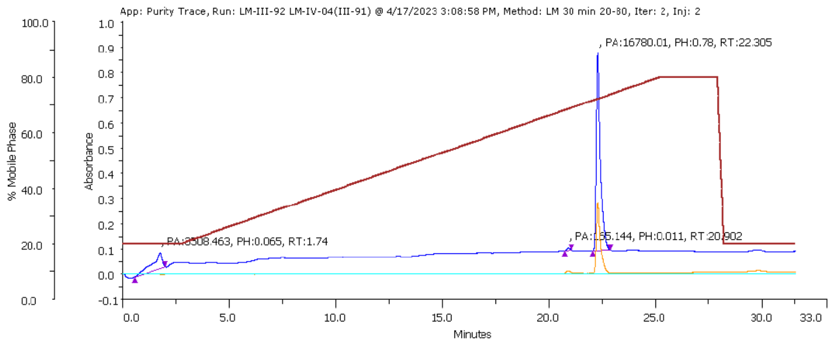

8D (100%)

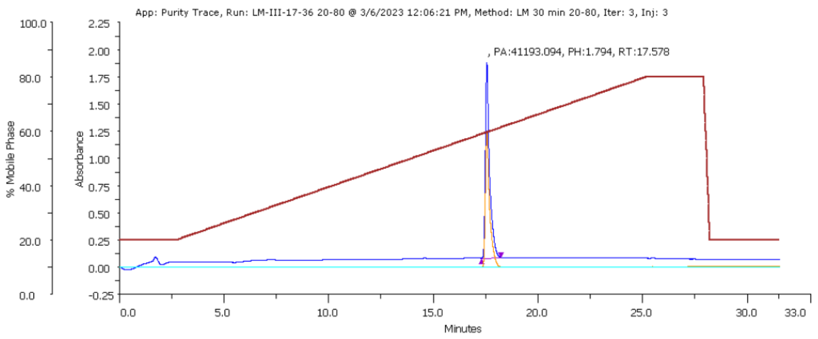

8E (99%)

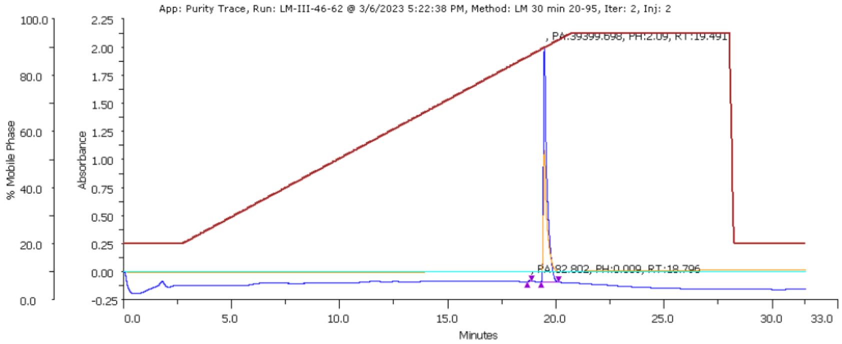

8F (100%)

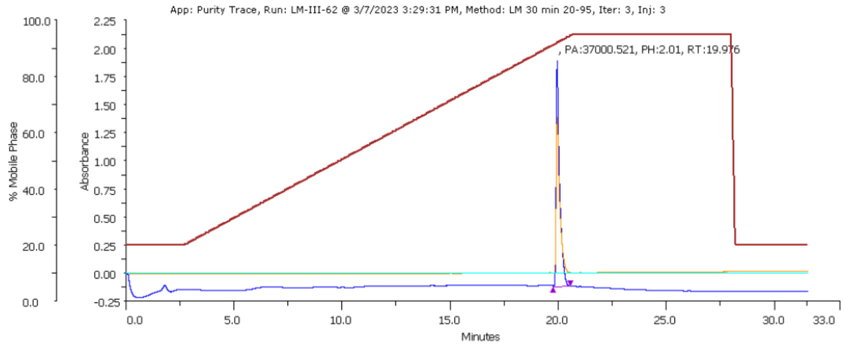

8G (100%)

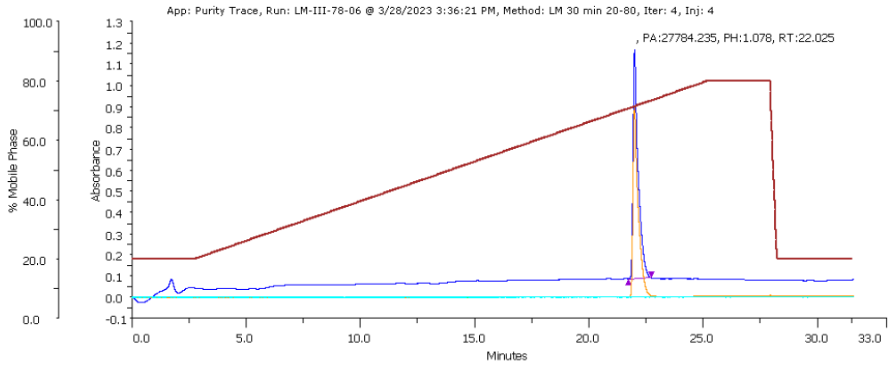

8H (99%)

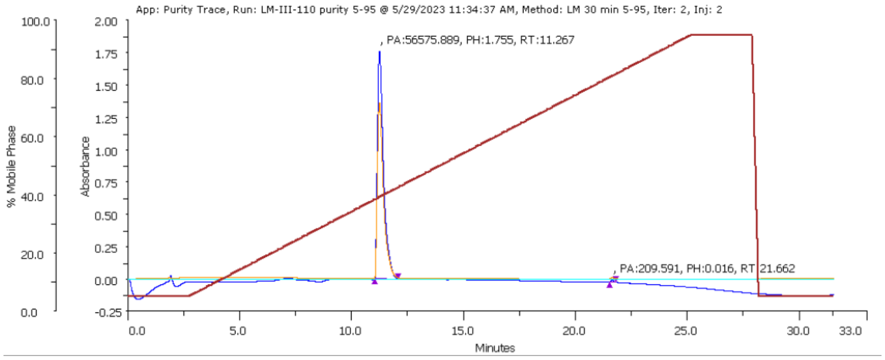

8I (100%)

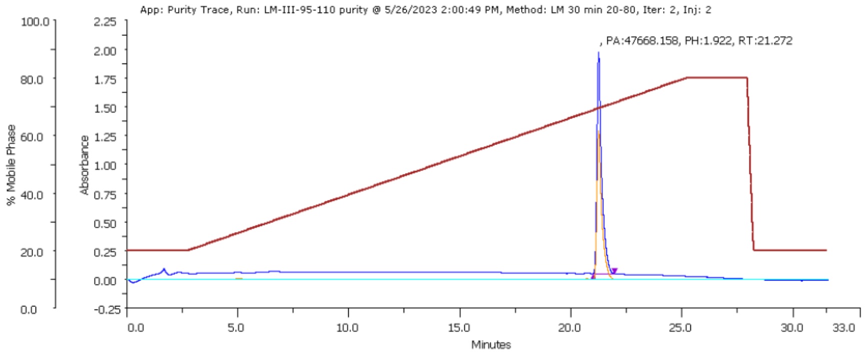

8J (99%)

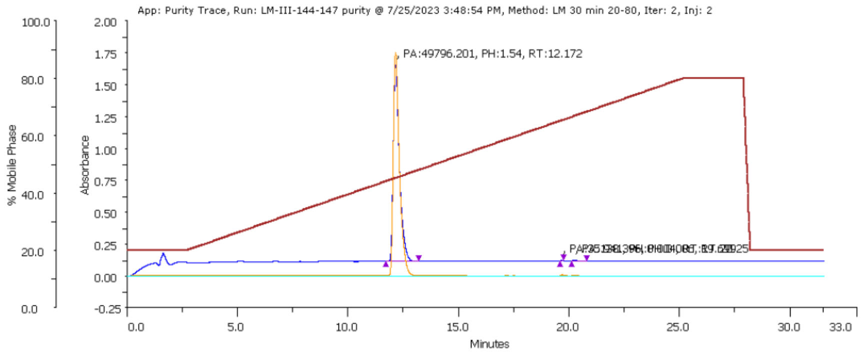

8K (96%)

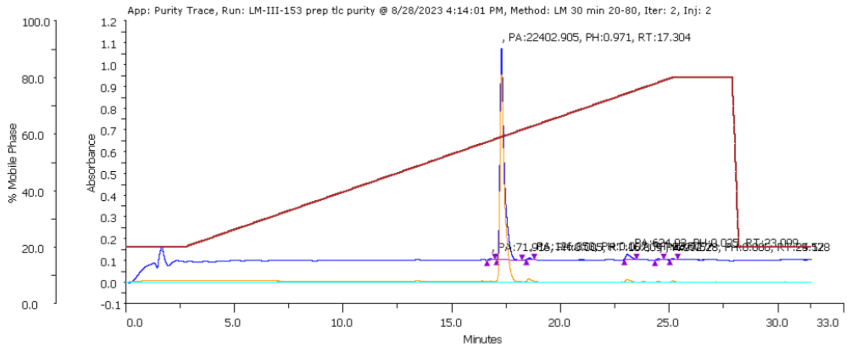

8L (99%)

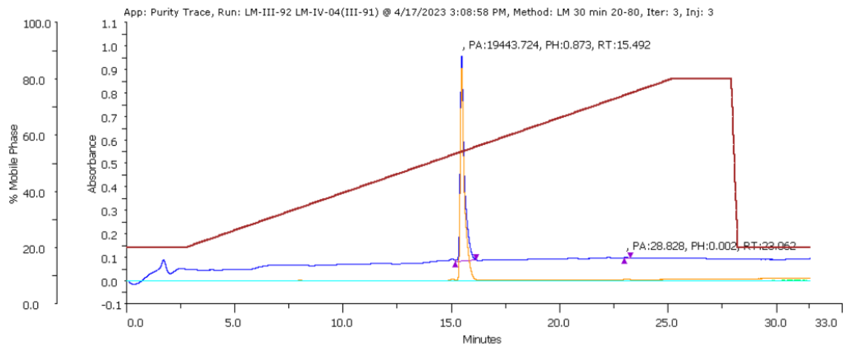

8M (99%)

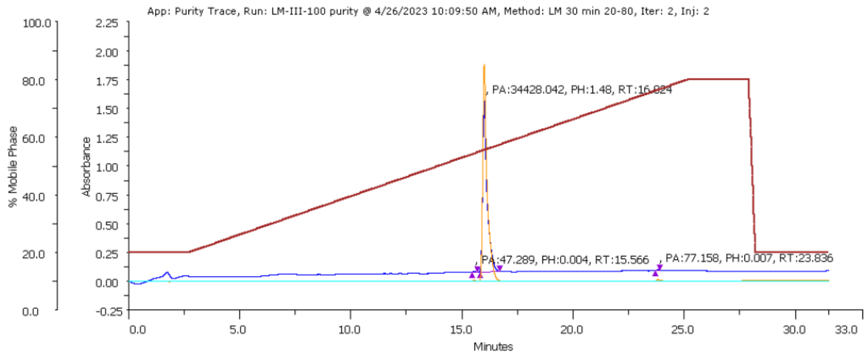

8N (100%)

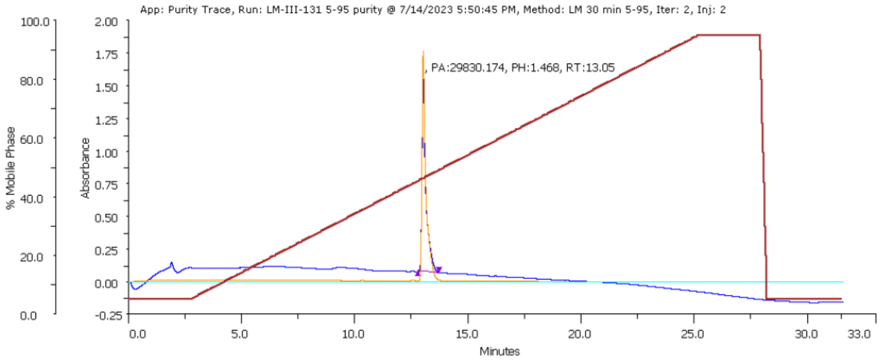

8O (99%)

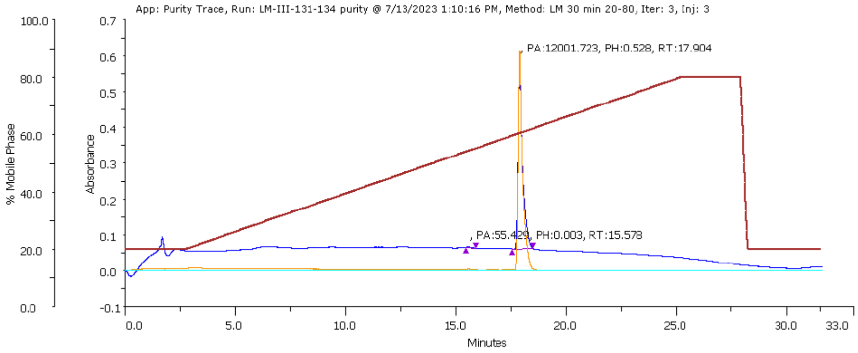

8P (98%)

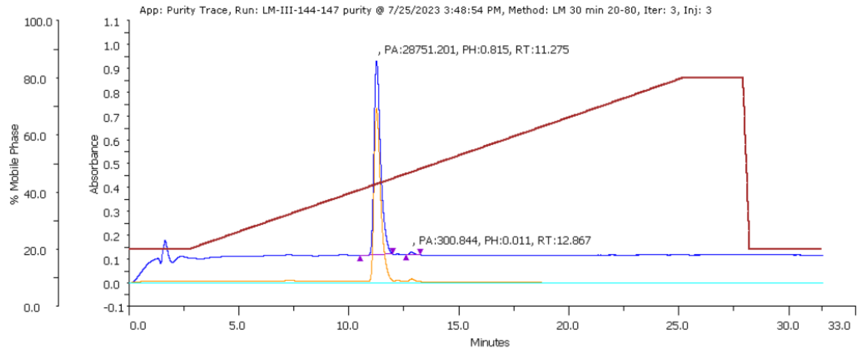

8Q (95%)

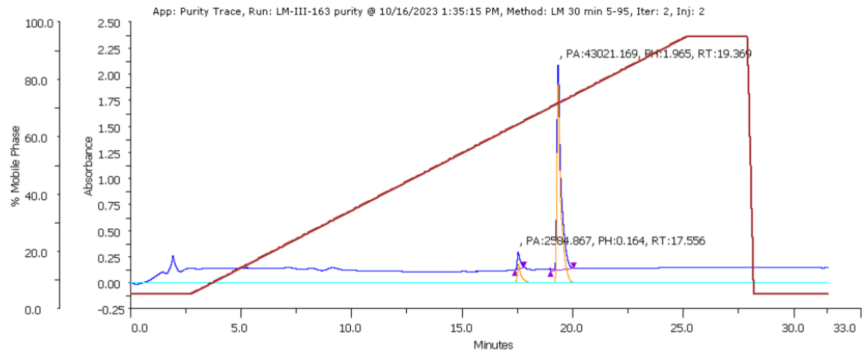

8R (100%)

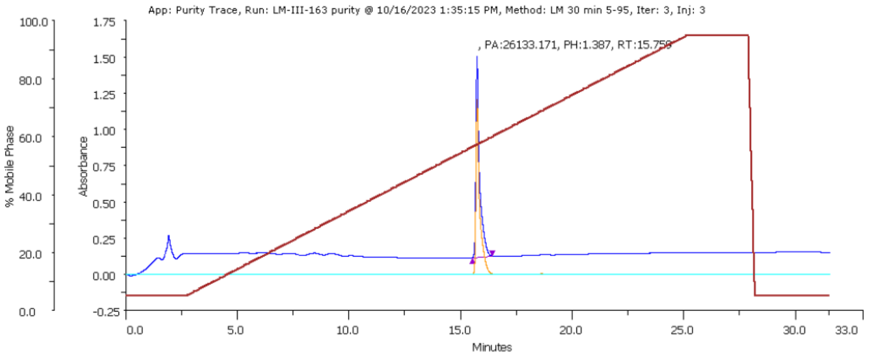

12 (100%)

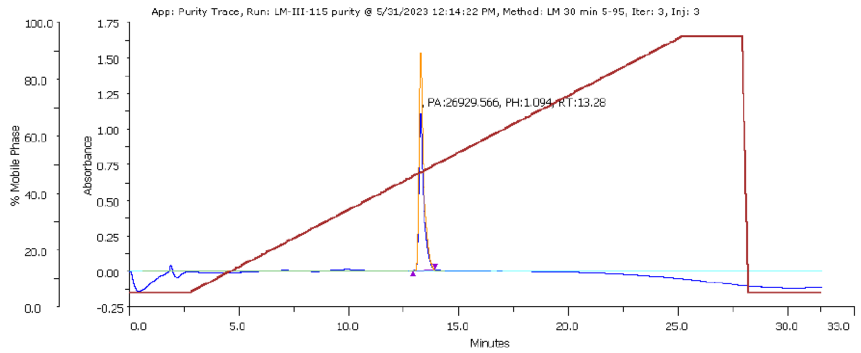

13 (98%)

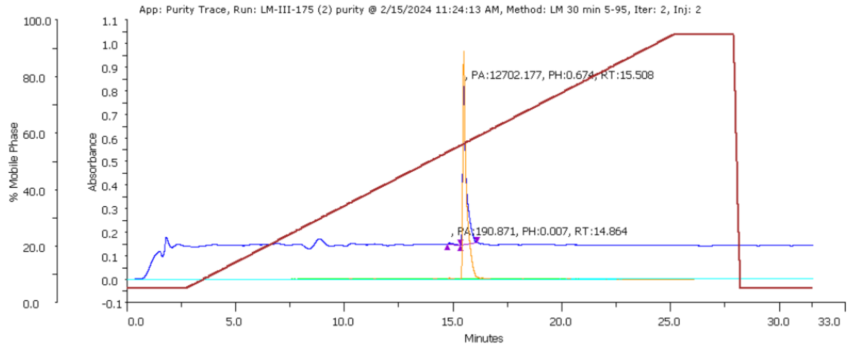

16 (99%)

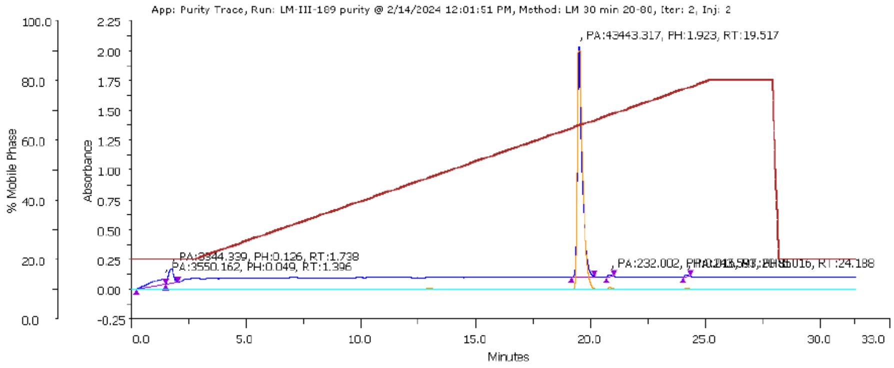

18 (99%)

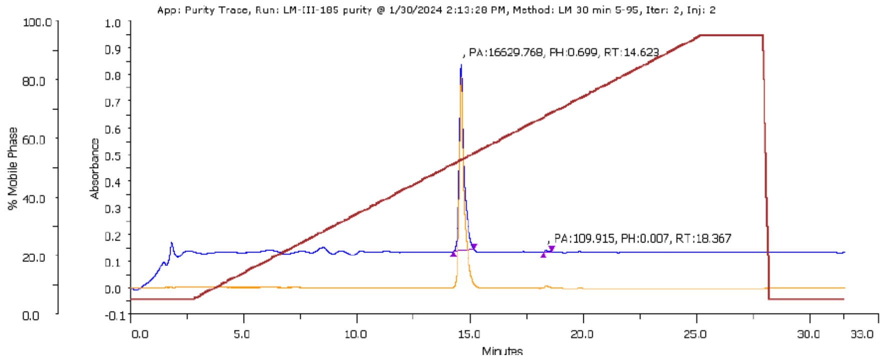

23A (100%)

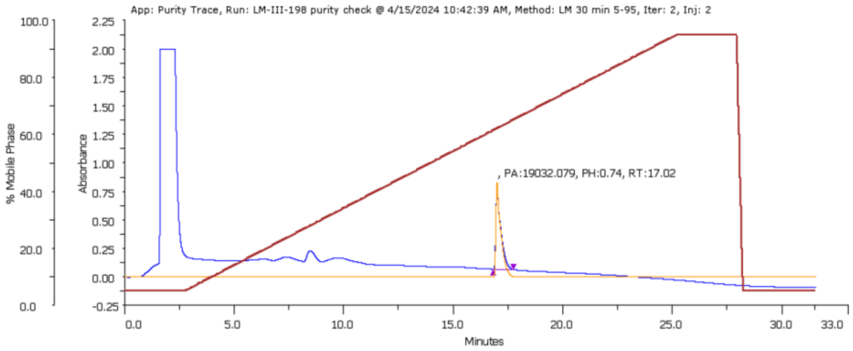

23B (100%)

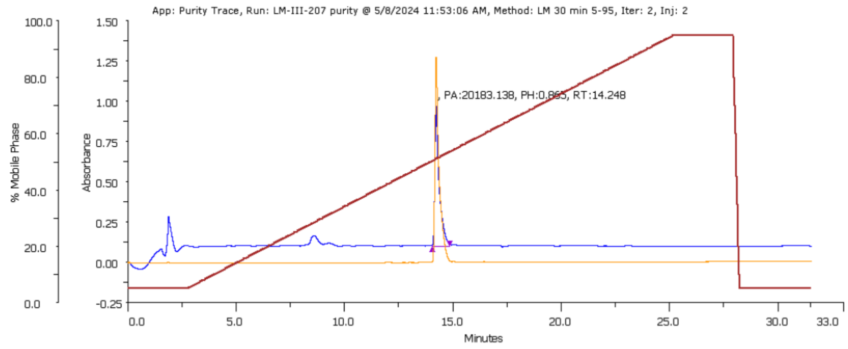

23C (100%)

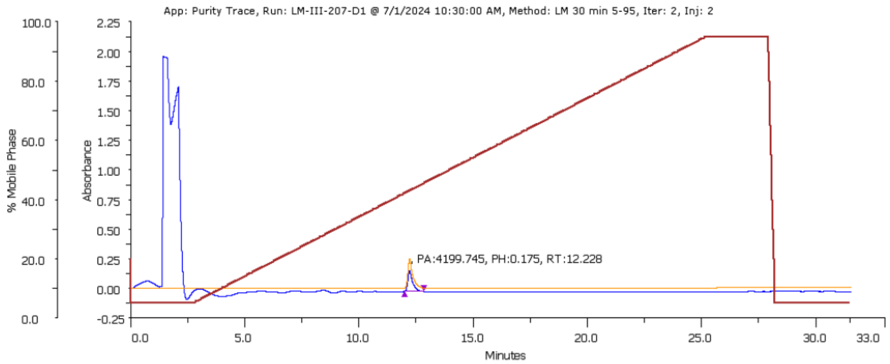

23D (100%)

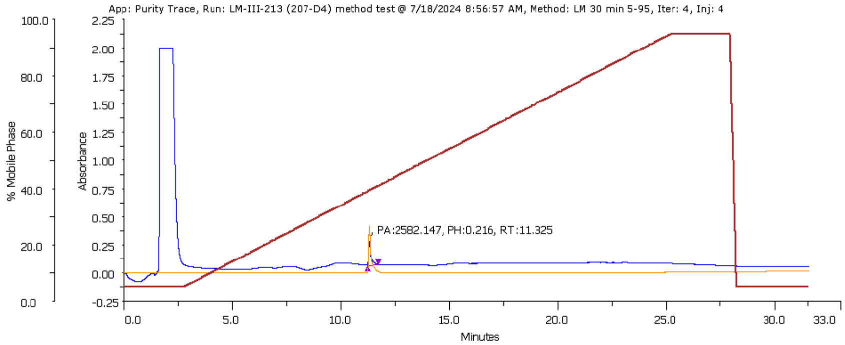

23E (100%)

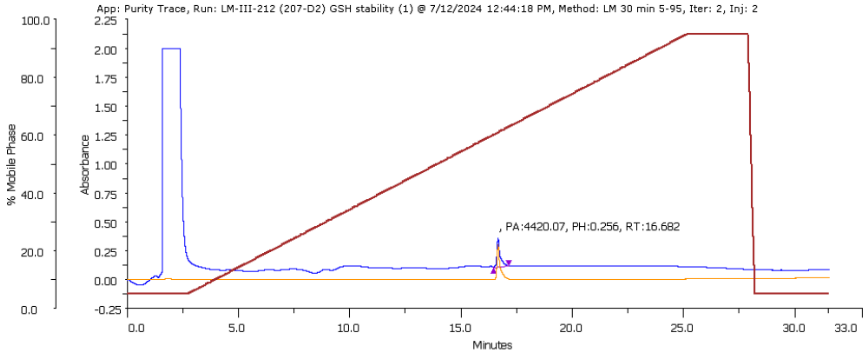

28 (97%)

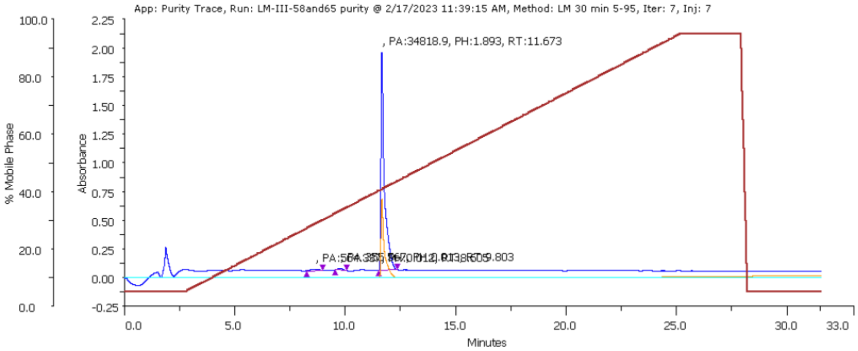

33 (97%)

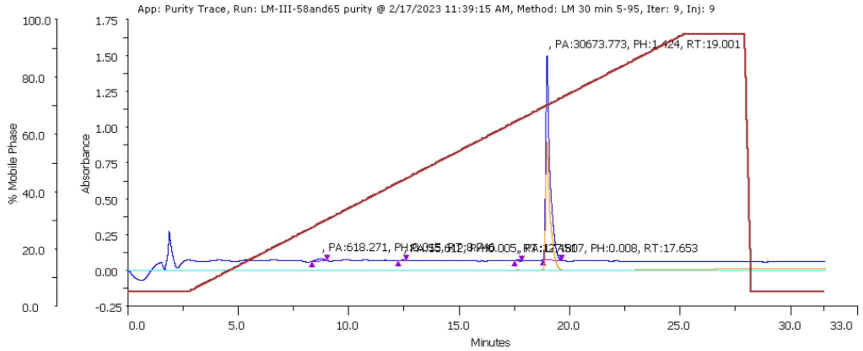

36 (99%)

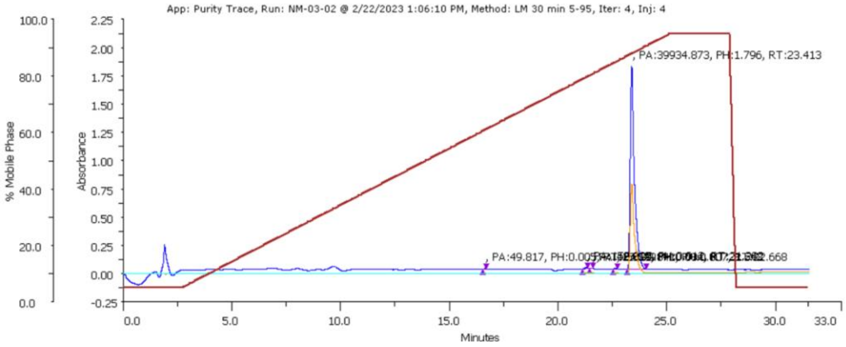

38 (91%)

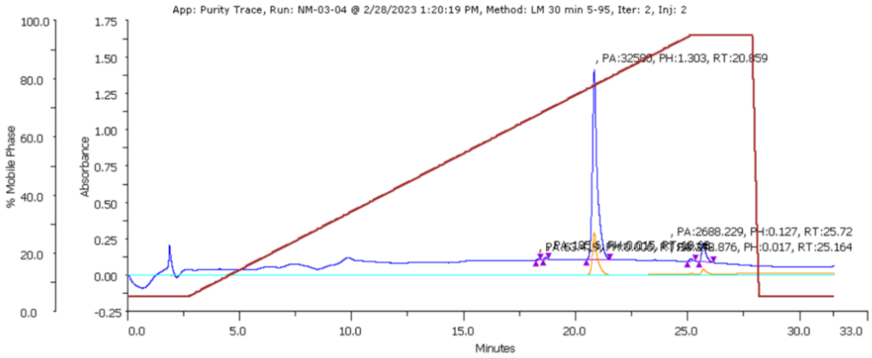

40 (99%)

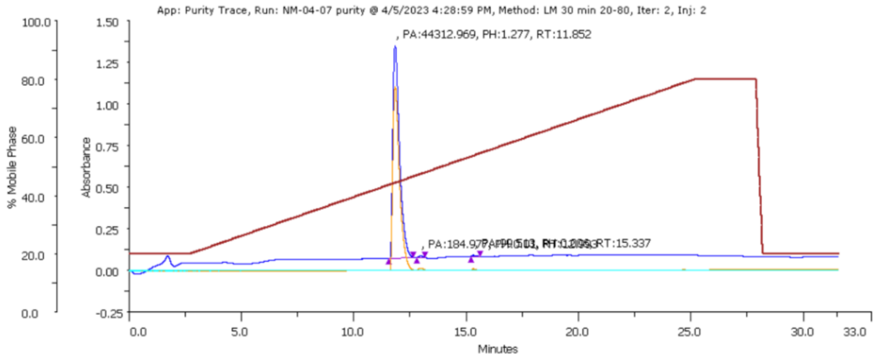

NMR Spectra of Final Compounds and New Synthetic Intermediates:

^{1}H NMR (300 MHz, $CDCl_3$) Spectrum of Compound **3C1**

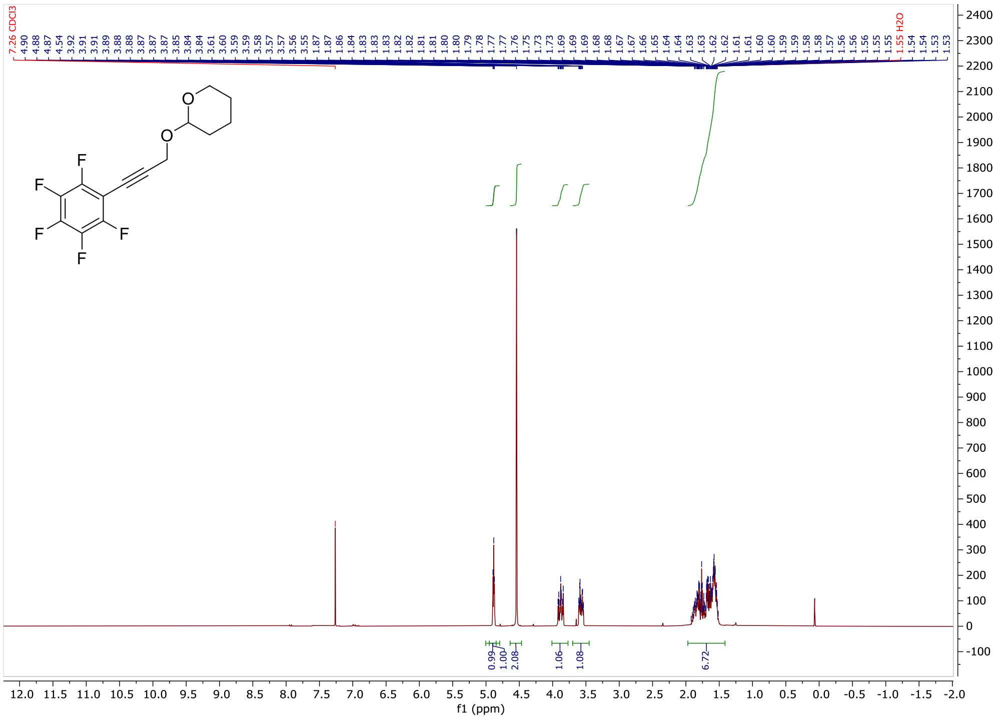

^{13}C NMR (75 MHz, $CDCl_3$) Spectrum of Compound **3C1**

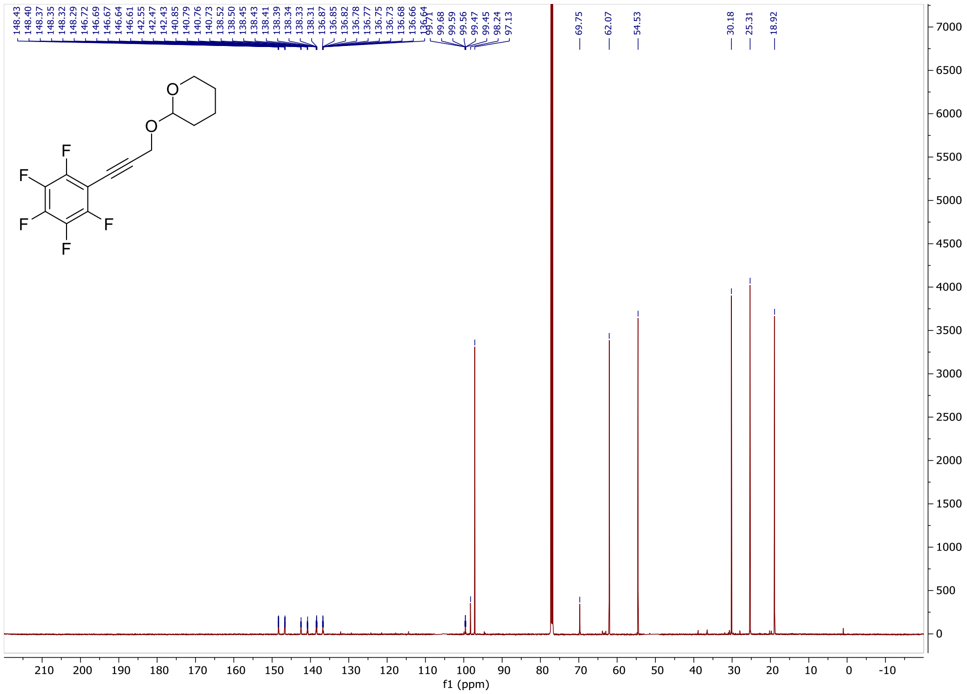

^{19}F NMR (282 MHz, $CDCl_3$) Spectrum of Compound **3C1**

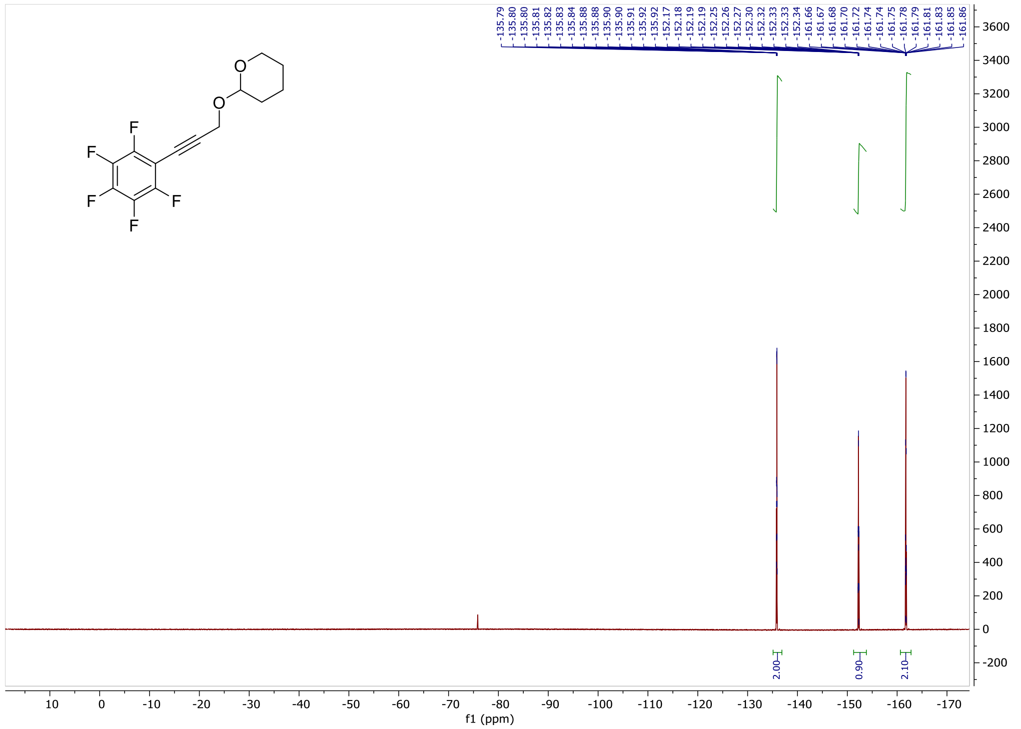

^{1}H NMR (300 MHz, $CDCl_3$) Spectrum of Compound **3C2**

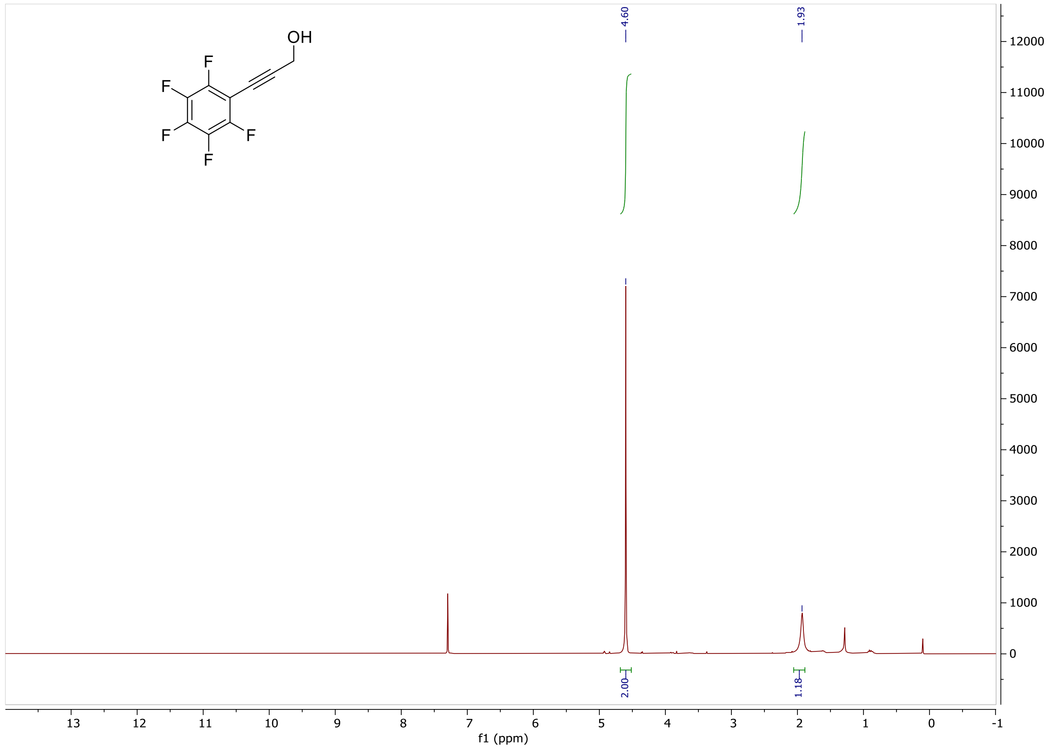

^{13}C NMR (75 MHz, $CDCl_3$) Spectrum of Compound **3C2**

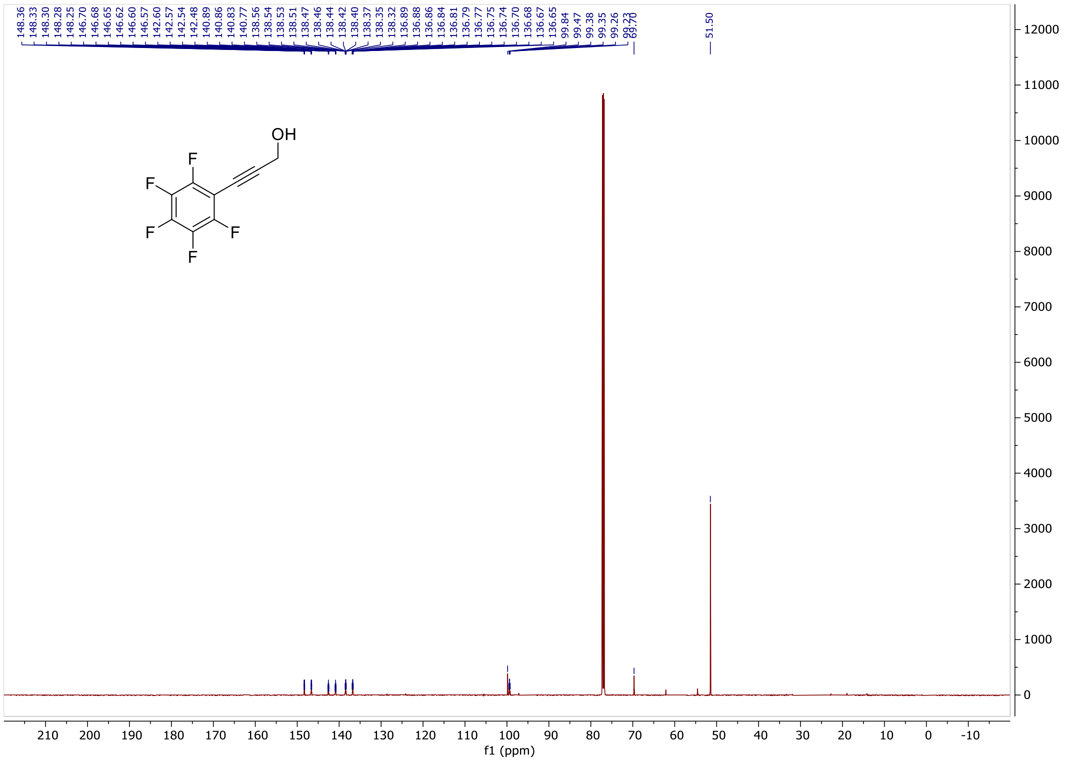

^{19}F NMR (282 MHz, $CDCl_3$) Spectrum of Compound **3C2**

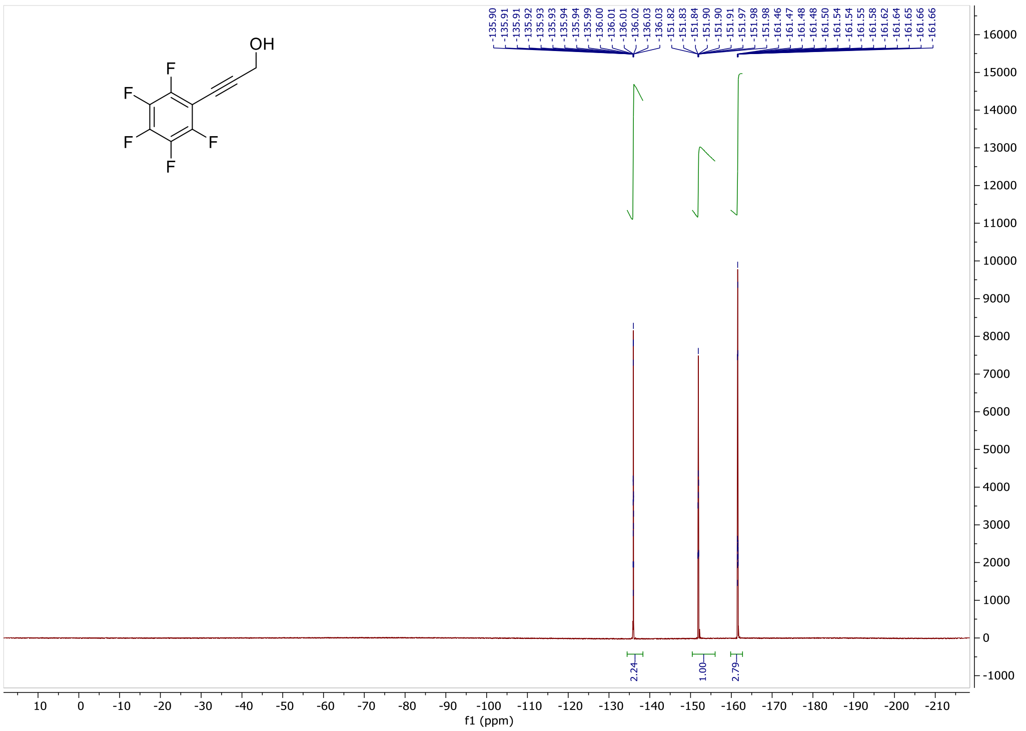

^{1}H NMR (600 MHz, $CDCl_3$) Spectrum of Compound **3G**

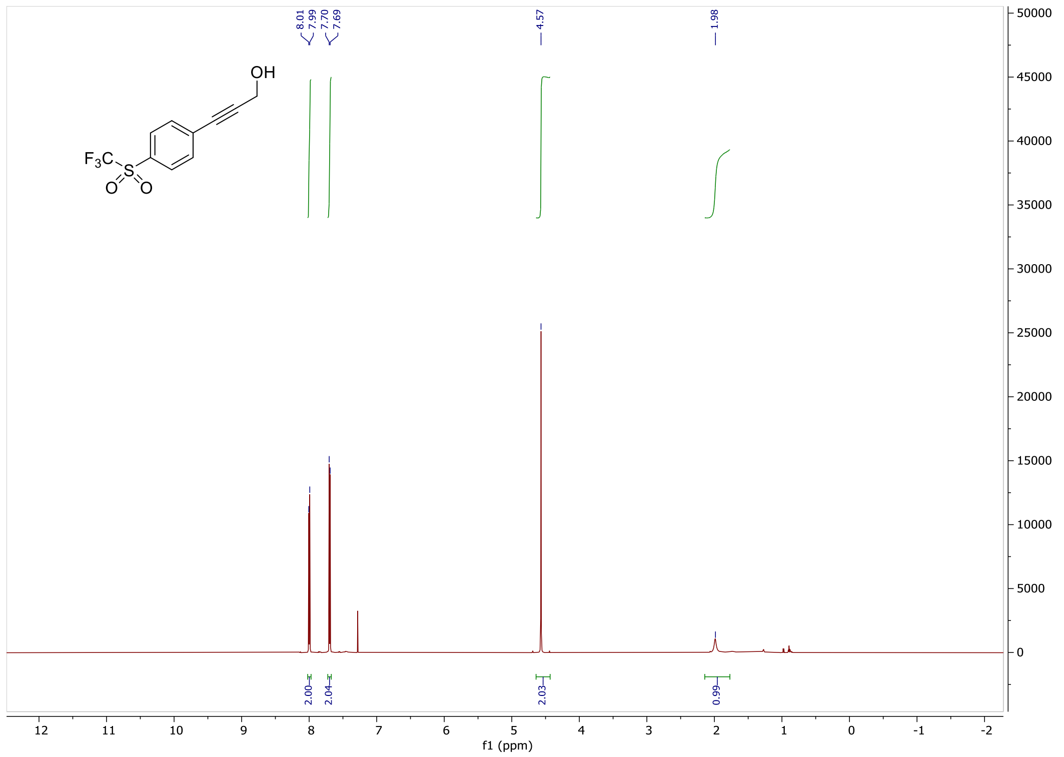

^{13}C NMR (150 MHz, $CDCl_3$) Spectrum of Compound **3G**

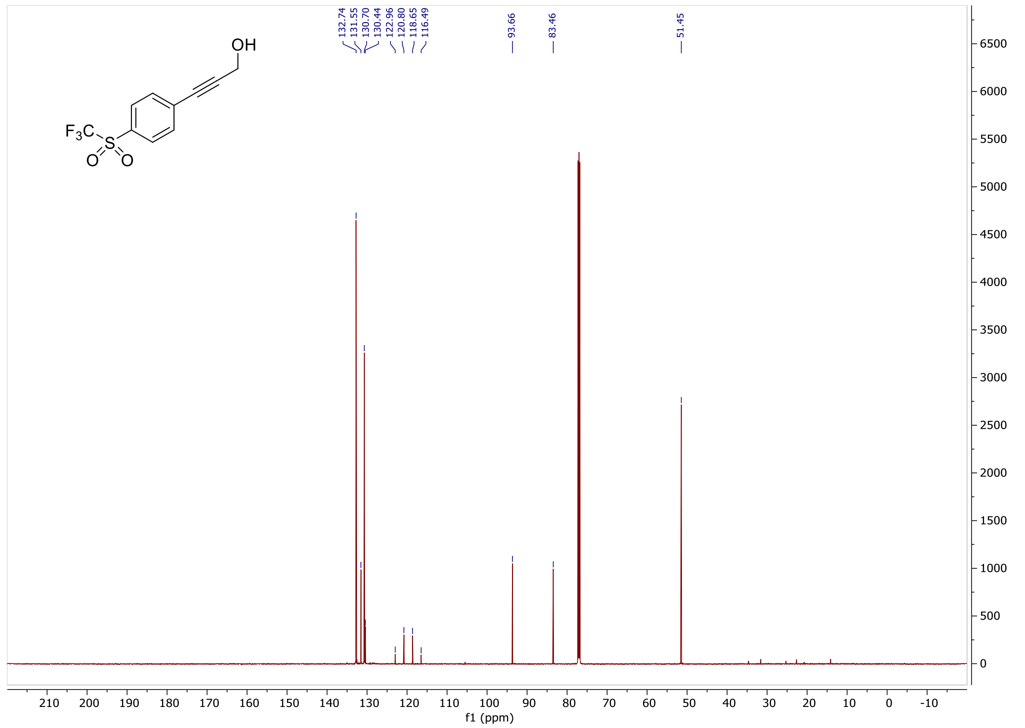

^{19}F NMR (282 MHz, $CDCl_3$) Spectrum of Compound **3G**

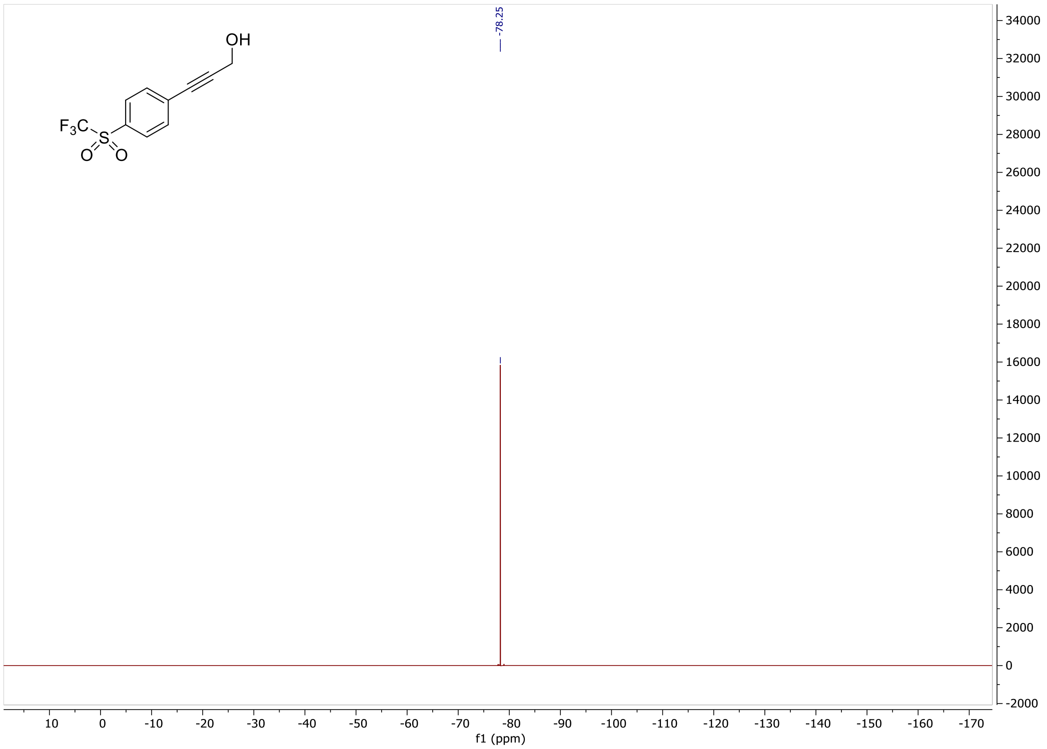

^{1}H NMR (600 MHz, $CDCl_3$) Spectrum of Compound **3H**

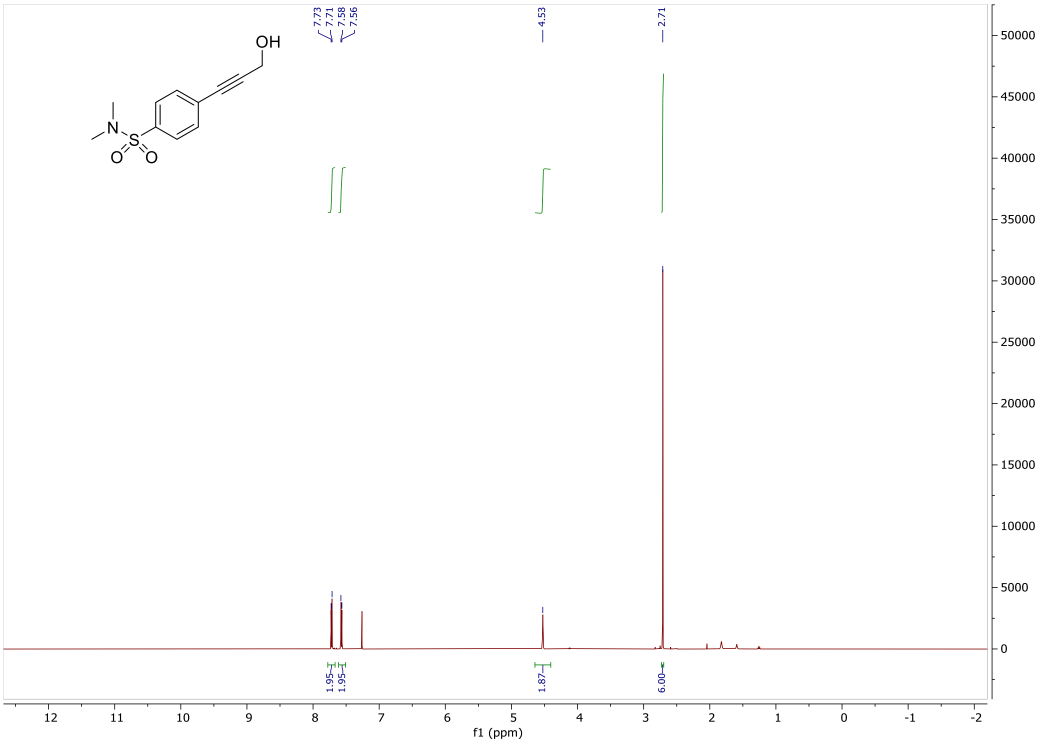

^{13}C NMR (150 MHz, $CDCl_3$) Spectrum of Compound **3H**

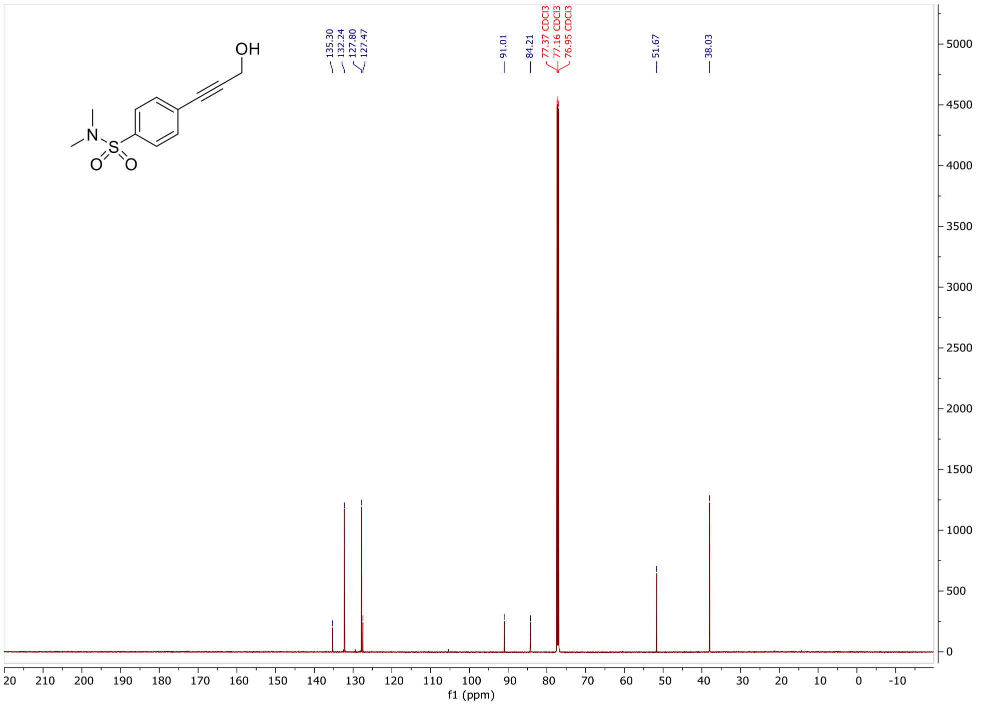

^{1}H NMR (300 MHz, $CDCl_3$) Spectrum of Compound **4C**

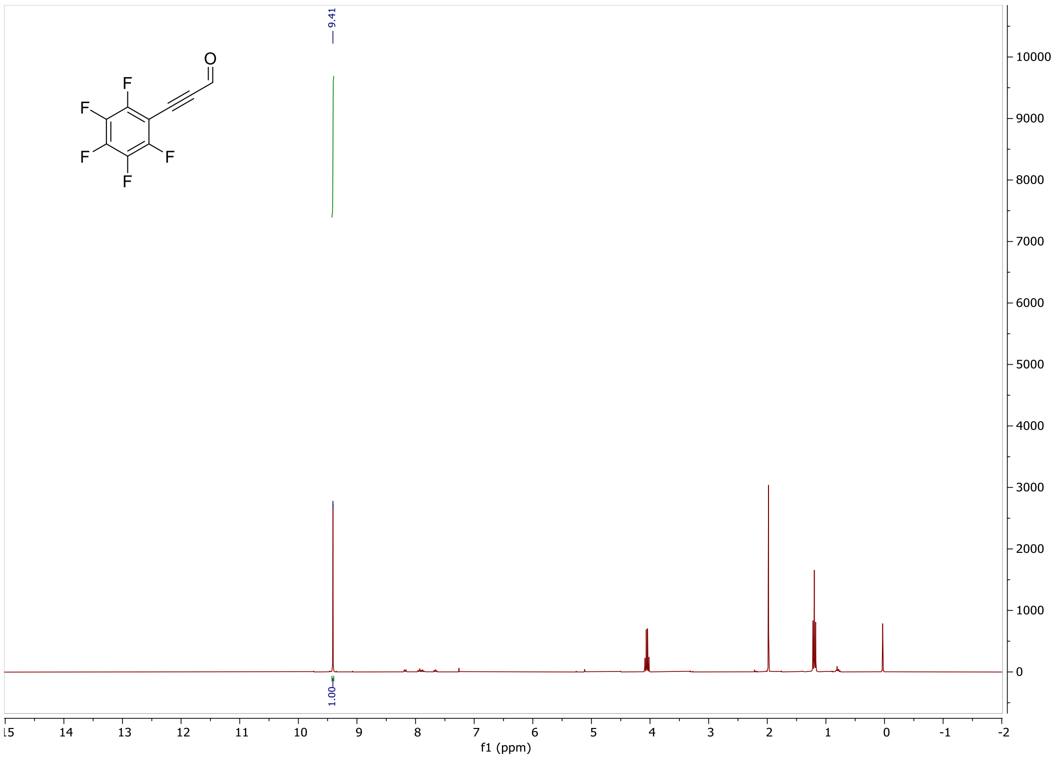

^{13}C NMR (75 MHz, $CDCl_3$) Spectrum of Compound **4C**

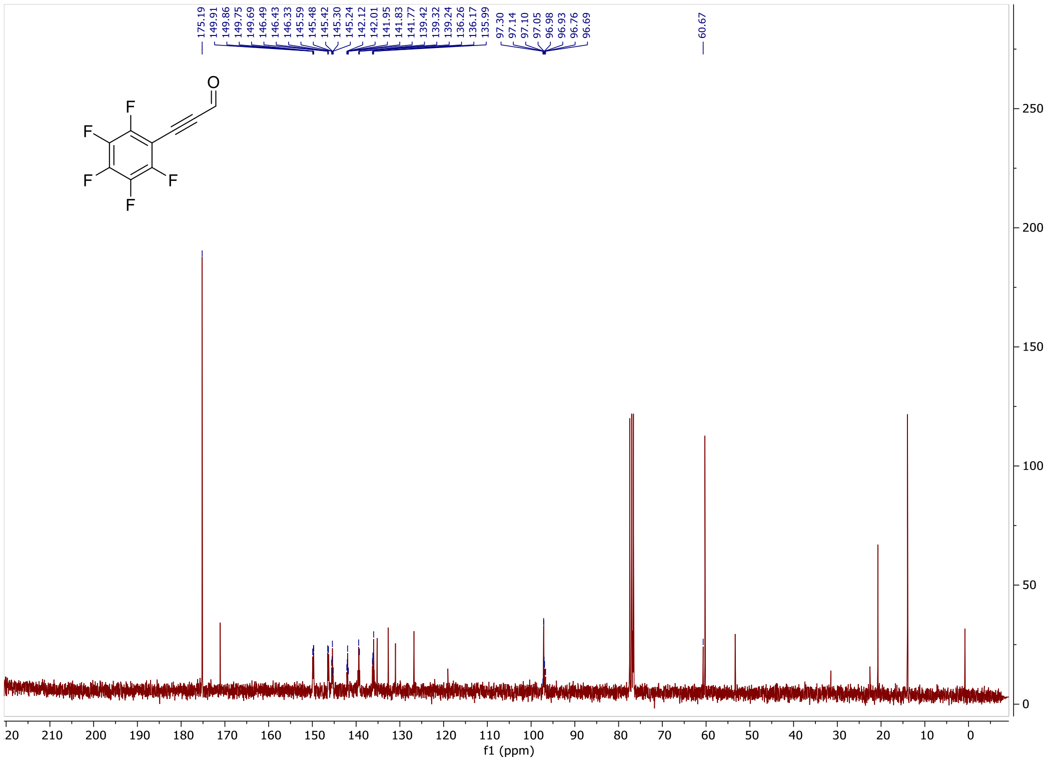

^{19}F NMR (282 MHz, $CDCl_3$) Spectrum of Compound **4C**

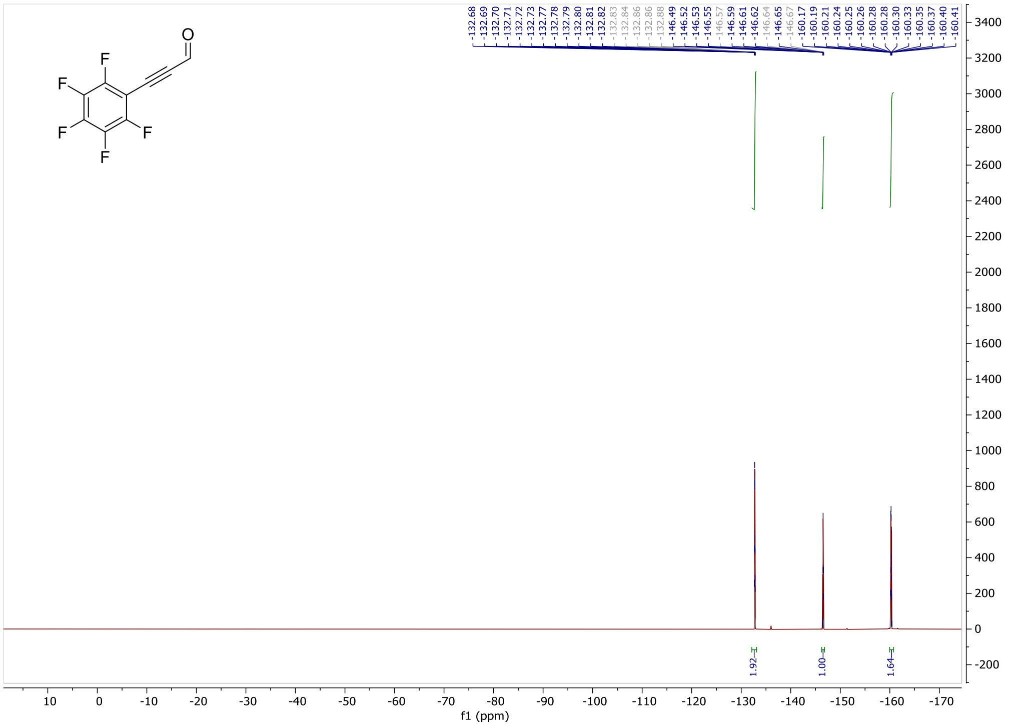

^{1}H NMR (300 MHz, $CDCl_3$) Spectrum of Compound **4F**

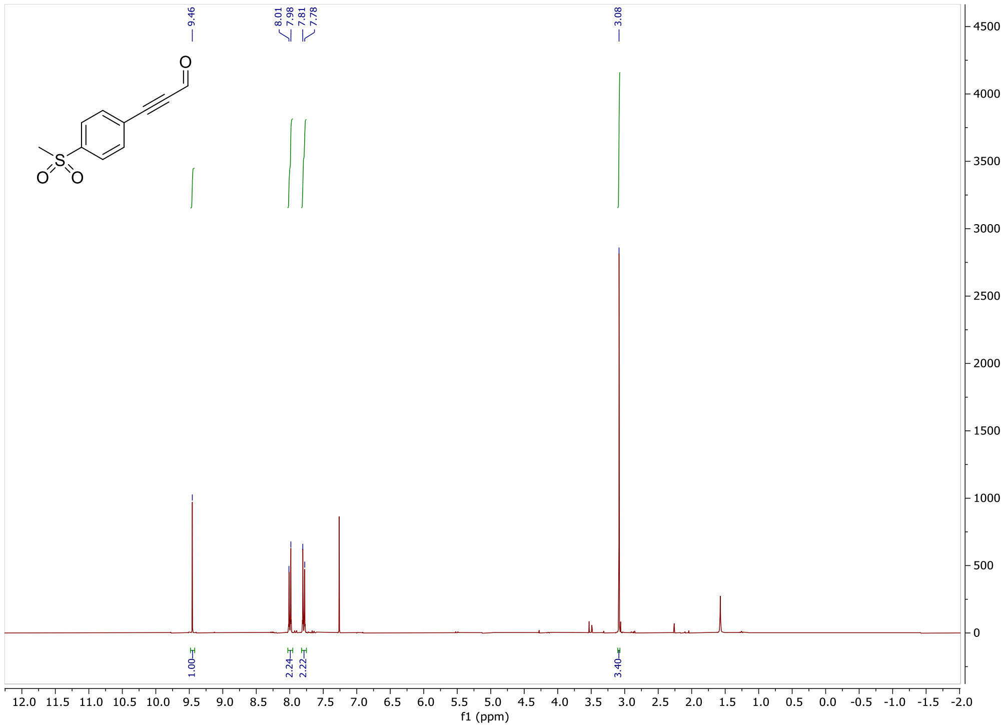

^{13}C NMR (75 MHz, $CDCl_3$) Spectrum of Compound **4F**

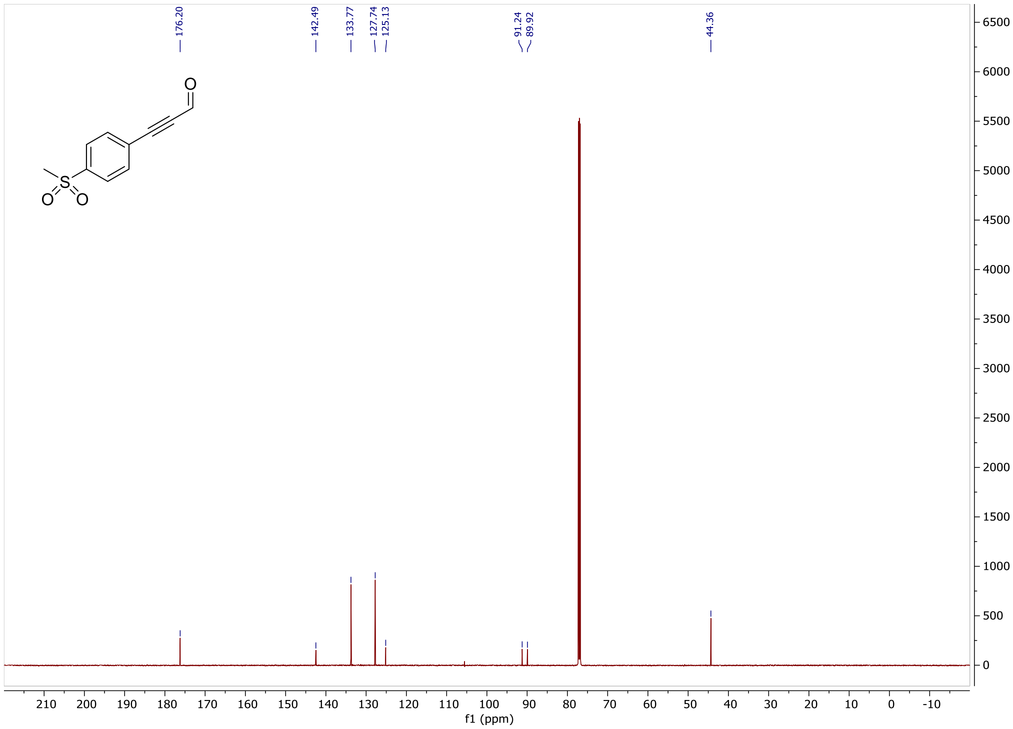

^{1}H NMR (300 MHz, $CDCl_3$) Spectrum of Compound **4G**

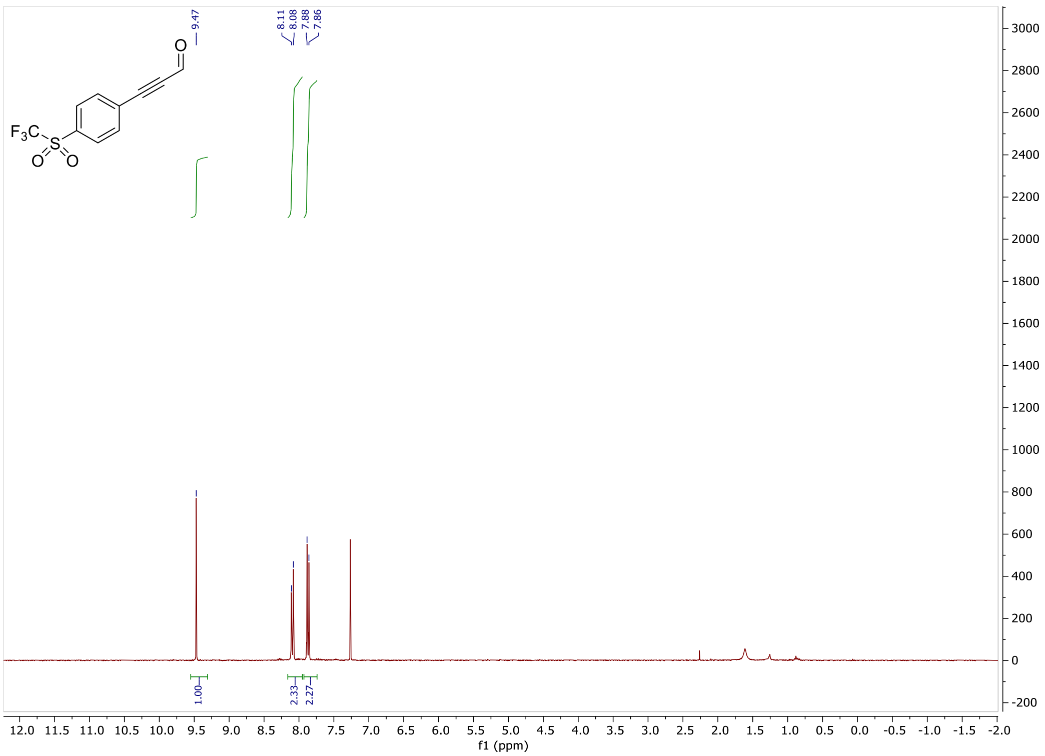

^{13}C NMR (150 MHz, $CDCl_3$) Spectrum of Compound **4G**

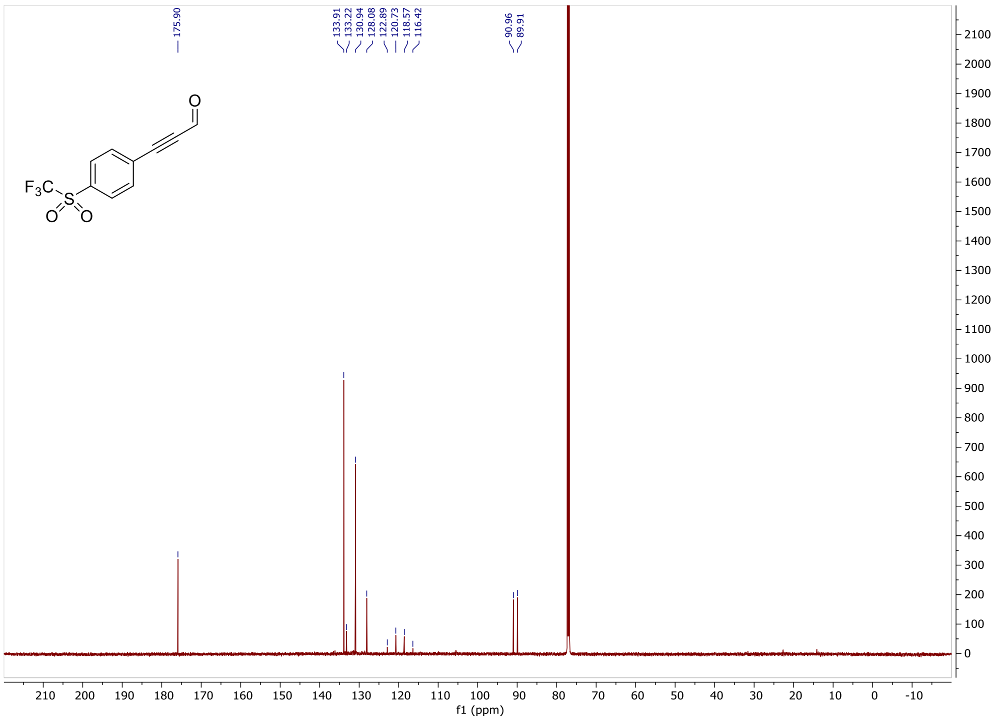

^{19}F NMR (282 MHz, $CDCl_3$) Spectrum of Compound **4G**

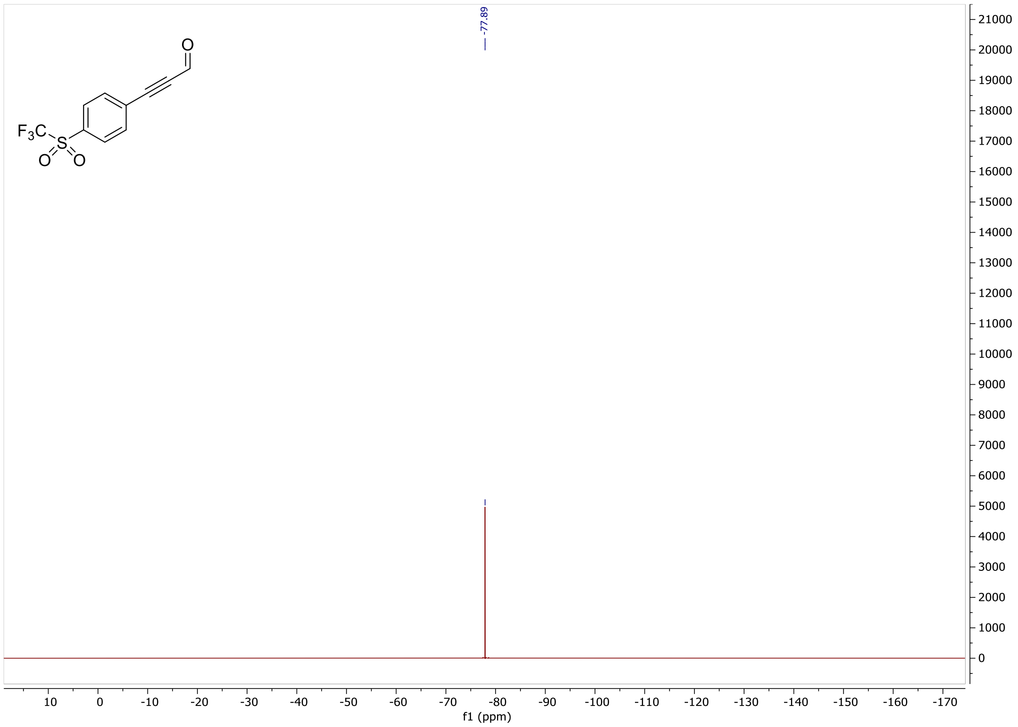

^{1}H NMR (600 MHz, $CDCl_3$) Spectrum of Compound **4H**

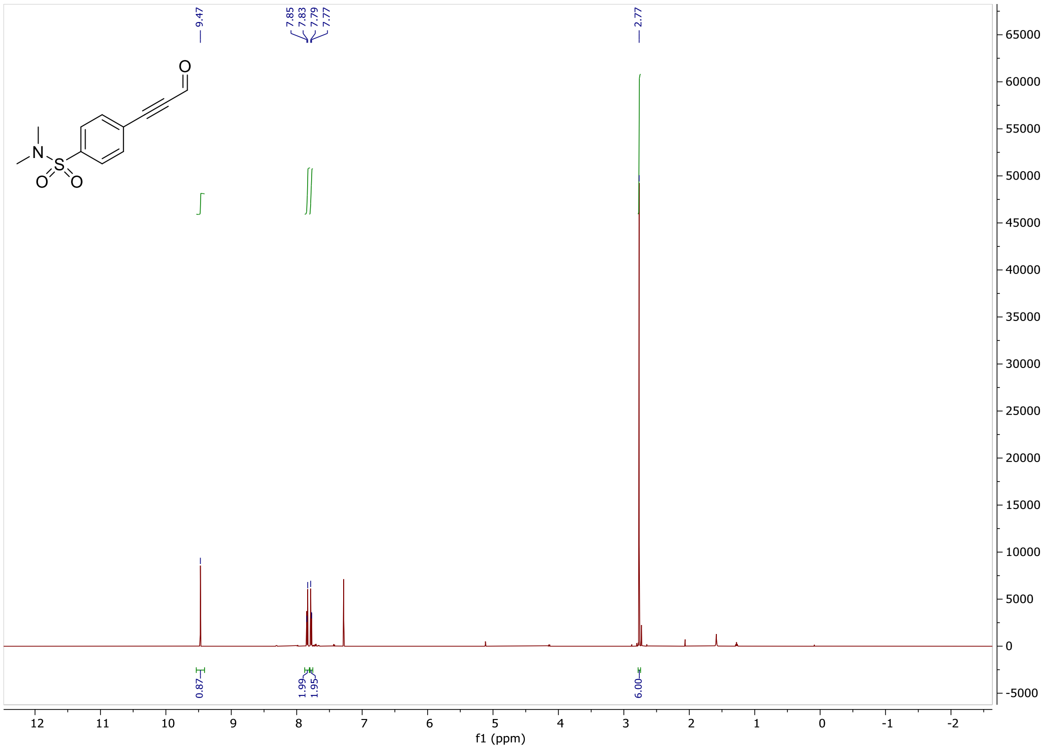

^{13}C NMR (150 MHz, $CDCl_3$) Spectrum of Compound **4H**

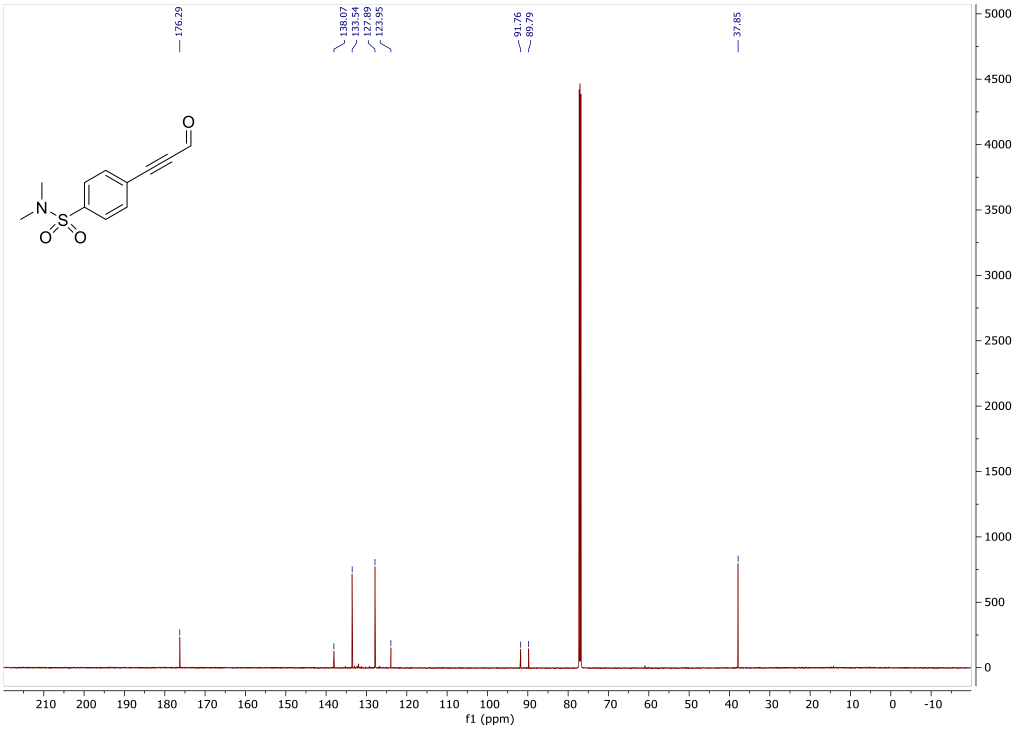

^{1}H NMR (600 MHz, $CDCl_3$) Spectrum of Compound **5C**

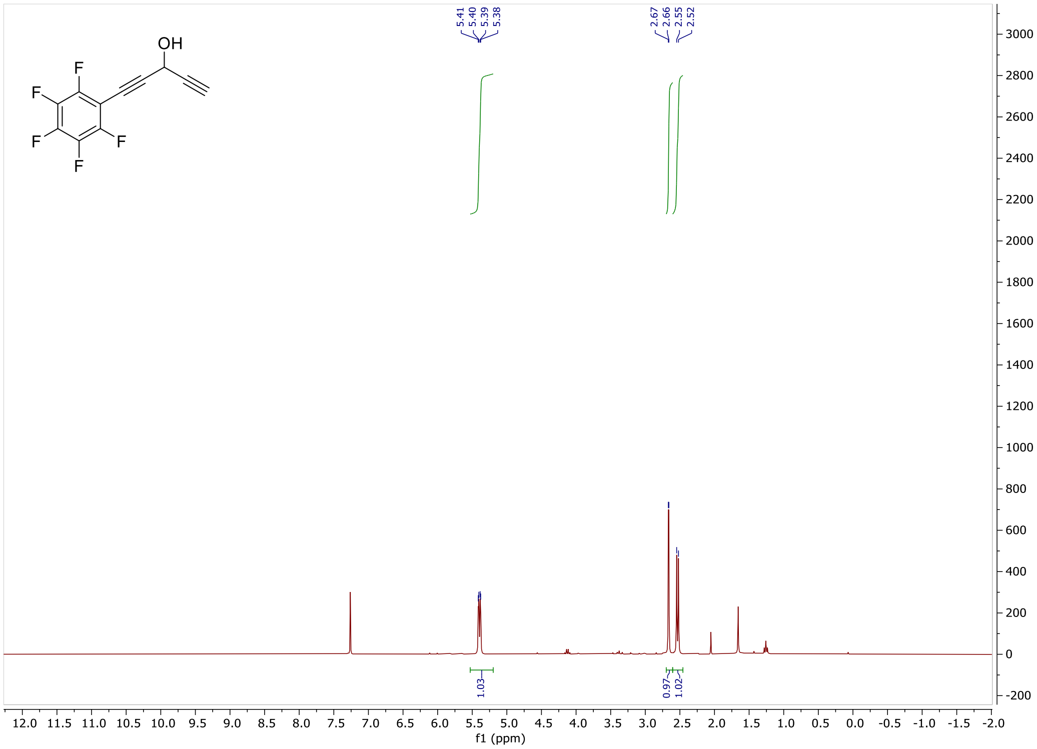

^{13}C NMR (150 MHz, $CDCl_3$) Spectrum of Compound **5C**

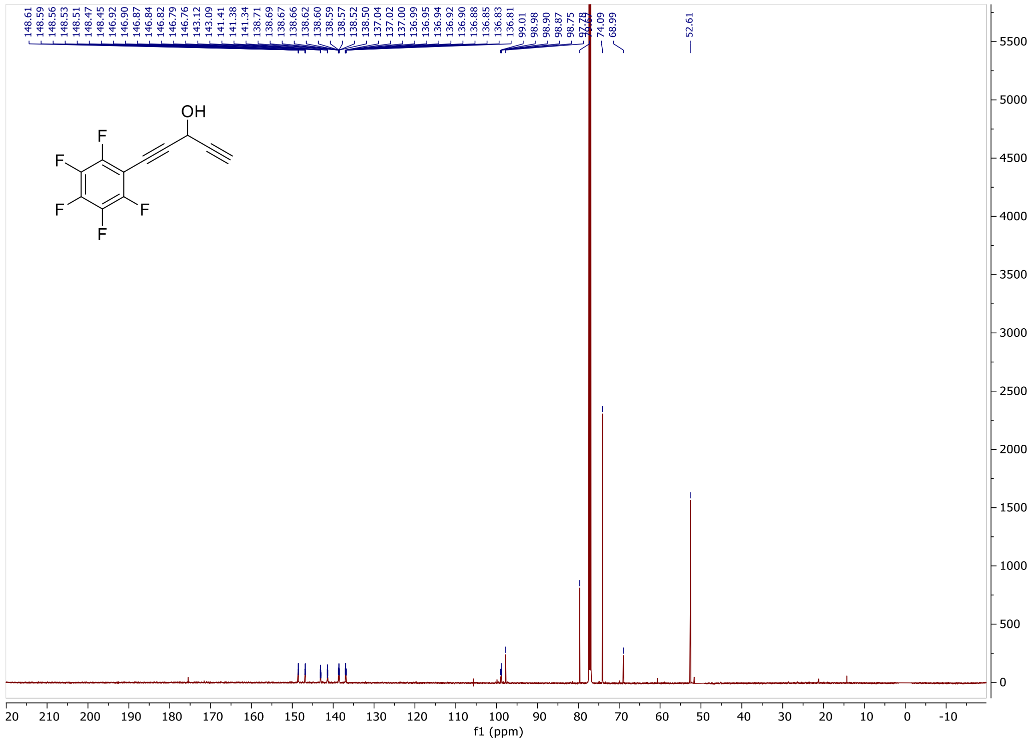

^{19}F NMR (282 MHz, $CDCl_3$) Spectrum of Compound **5C**

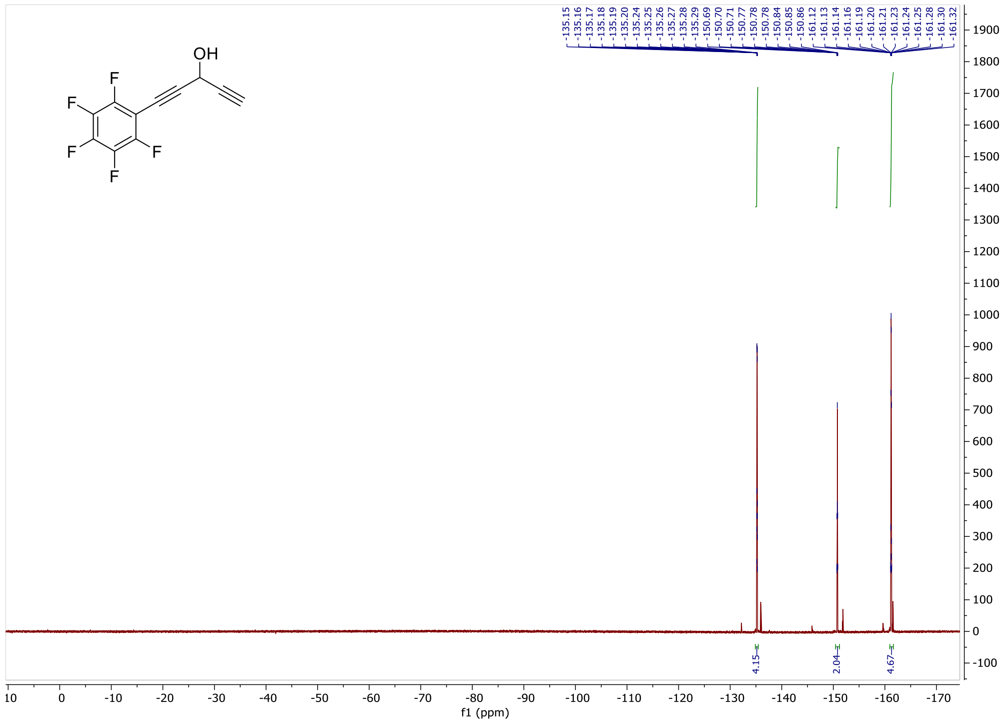

^{1}H NMR (300 MHz, $CDCl_3$) Spectrum of Compound **5E**

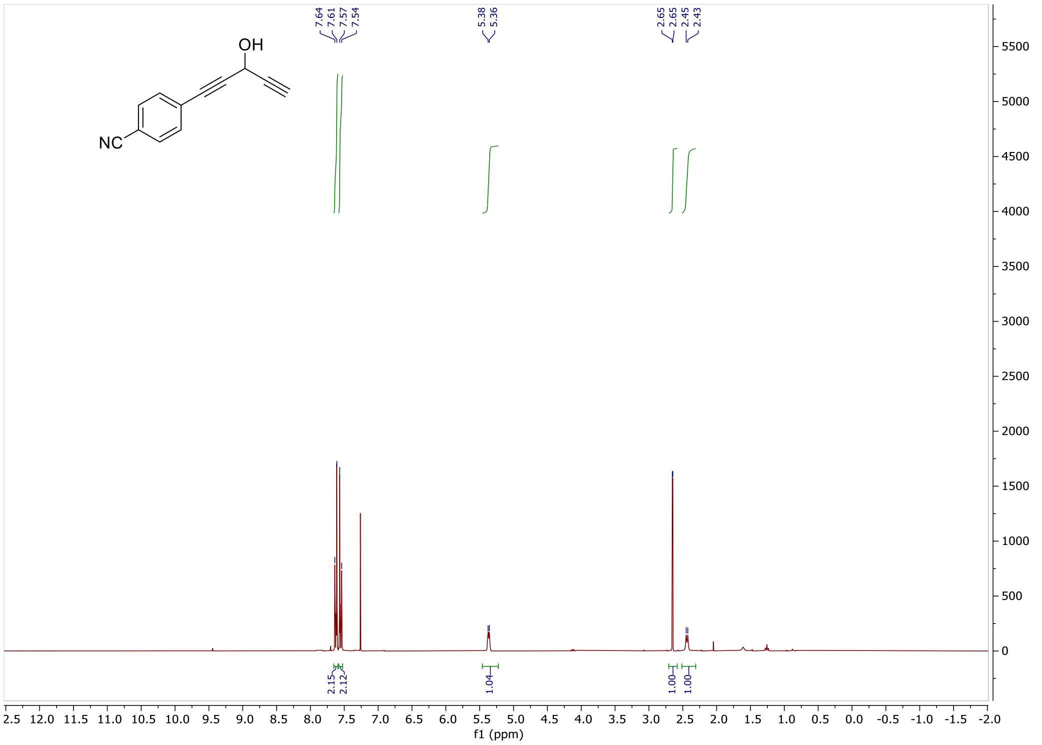

^{13}C NMR (75 MHz, $CDCl_3$) Spectrum of Compound **5E**

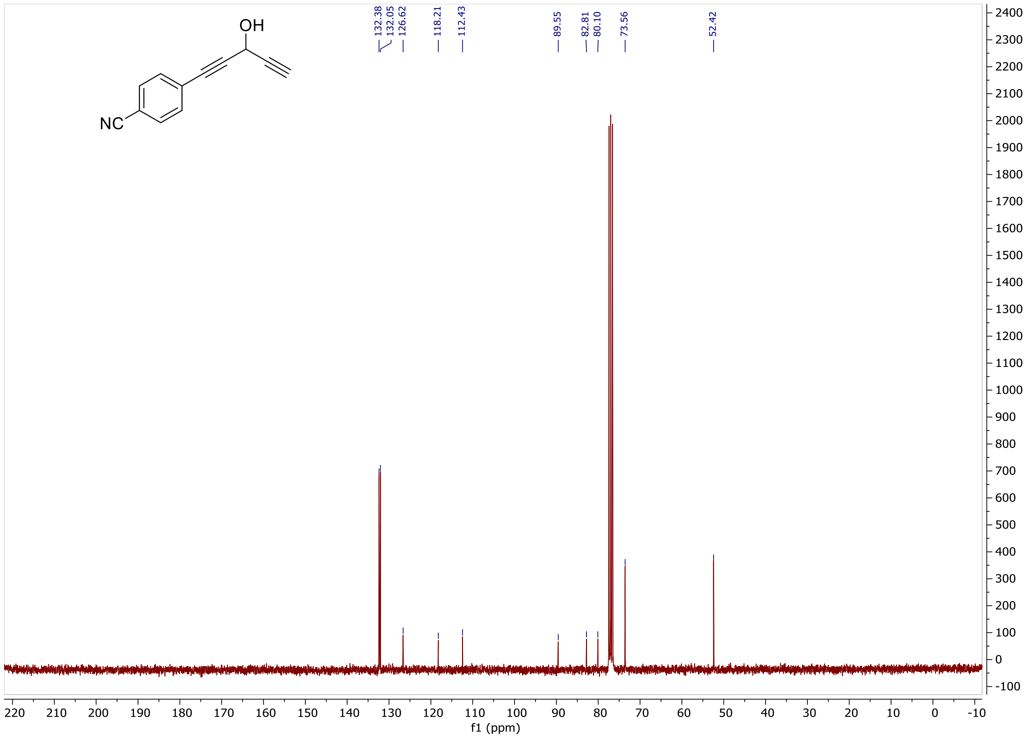

^{1}H NMR (300 MHz, $CDCl_3$) Spectrum of Compound **5F**

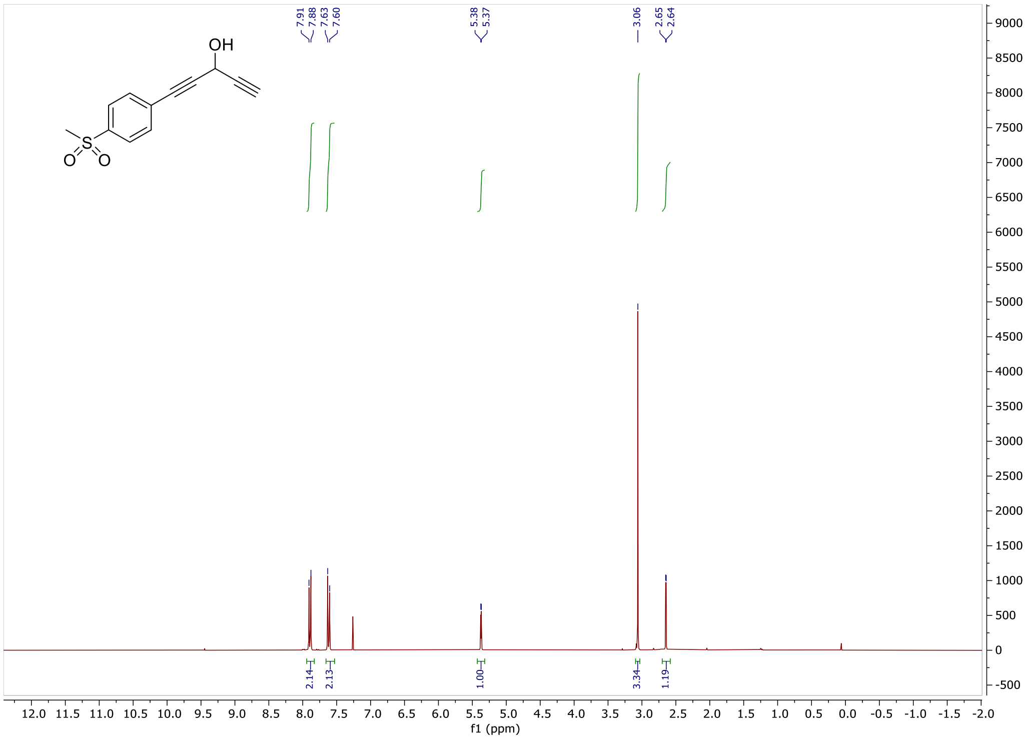

^{13}C NMR (75 MHz, $CDCl_3$) Spectrum of Compound **5F**

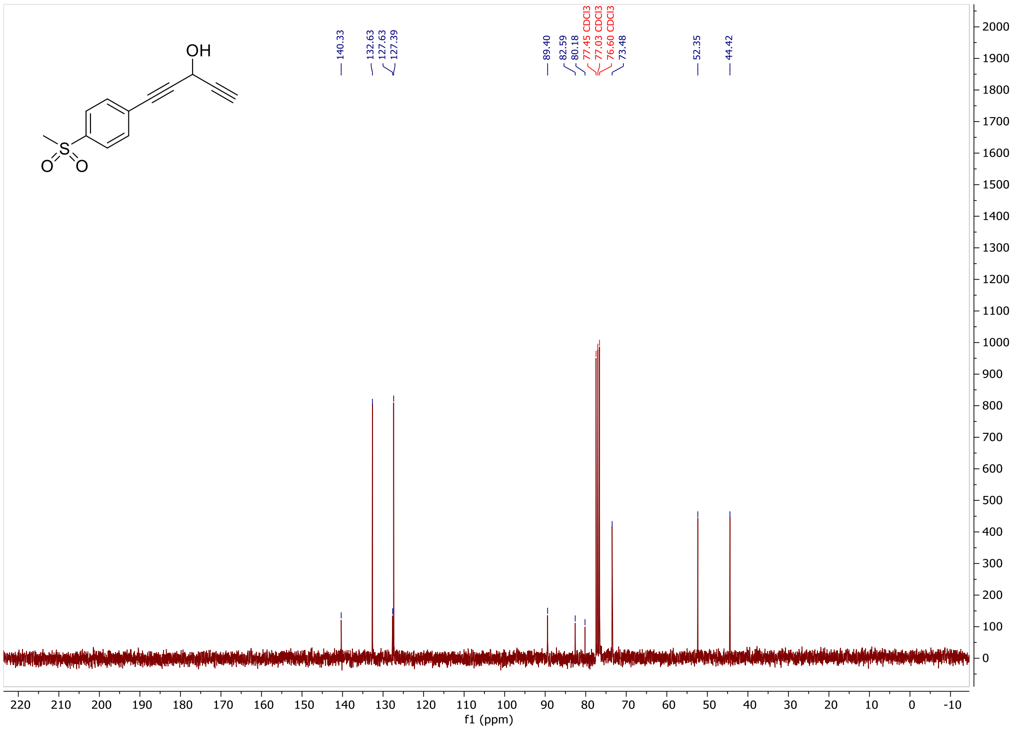

^{1}H NMR (300 MHz, $CDCl_3$) Spectrum of Compound **5G**

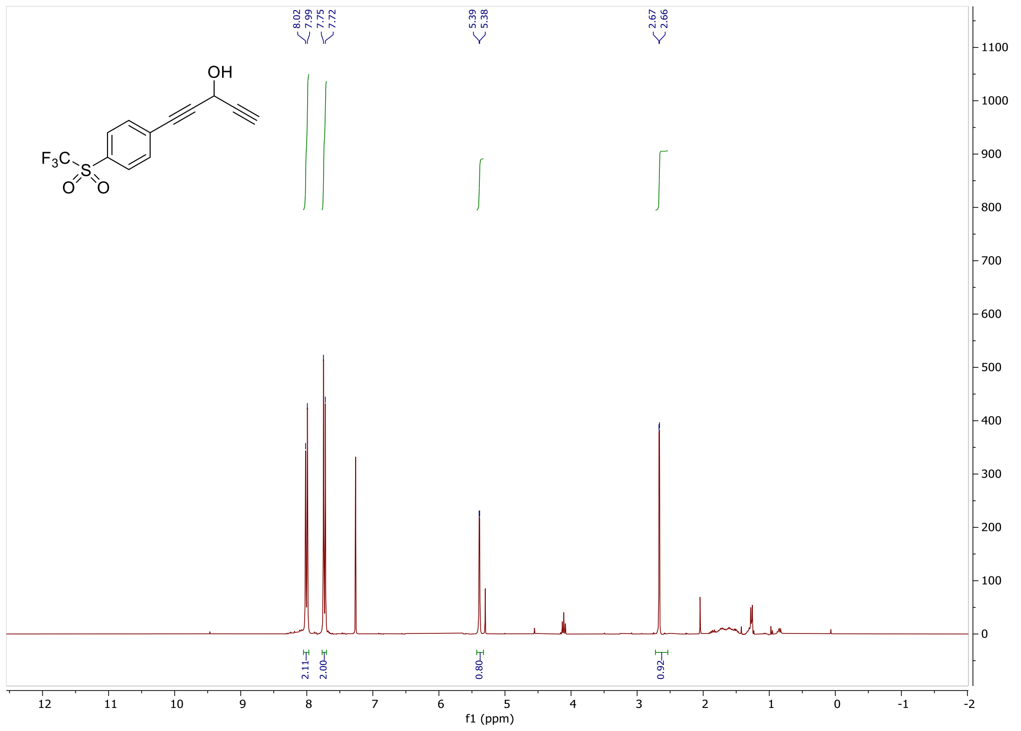

^{13}C NMR (150 MHz, $CDCl_3$) Spectrum of Compound **5G**

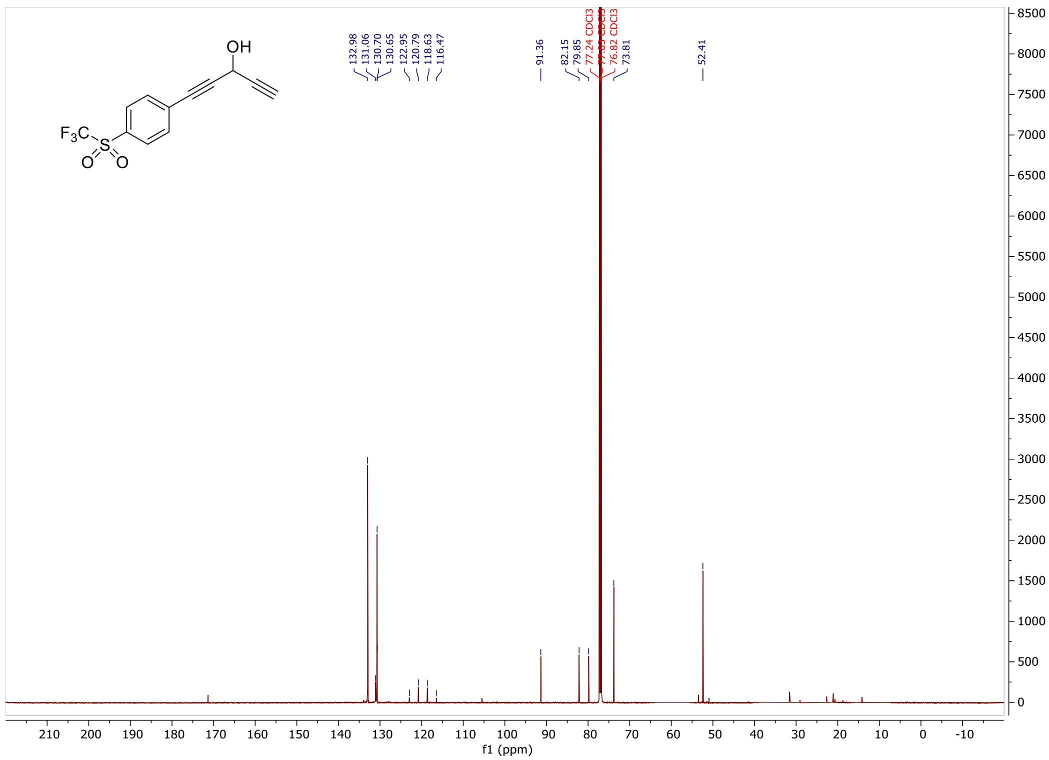

^{19}F NMR (282 MHz, $CDCl_3$) Spectrum of Compound **5G**

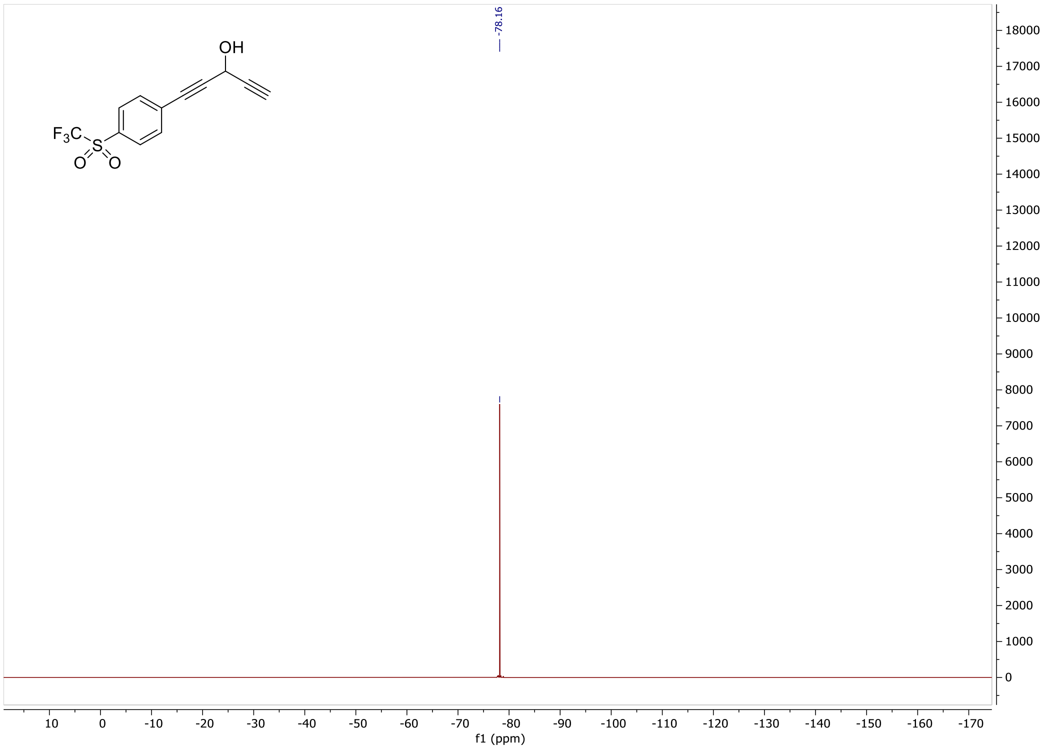

^{1}H NMR (600 MHz, $CDCl_3$) Spectrum of Compound **5H**

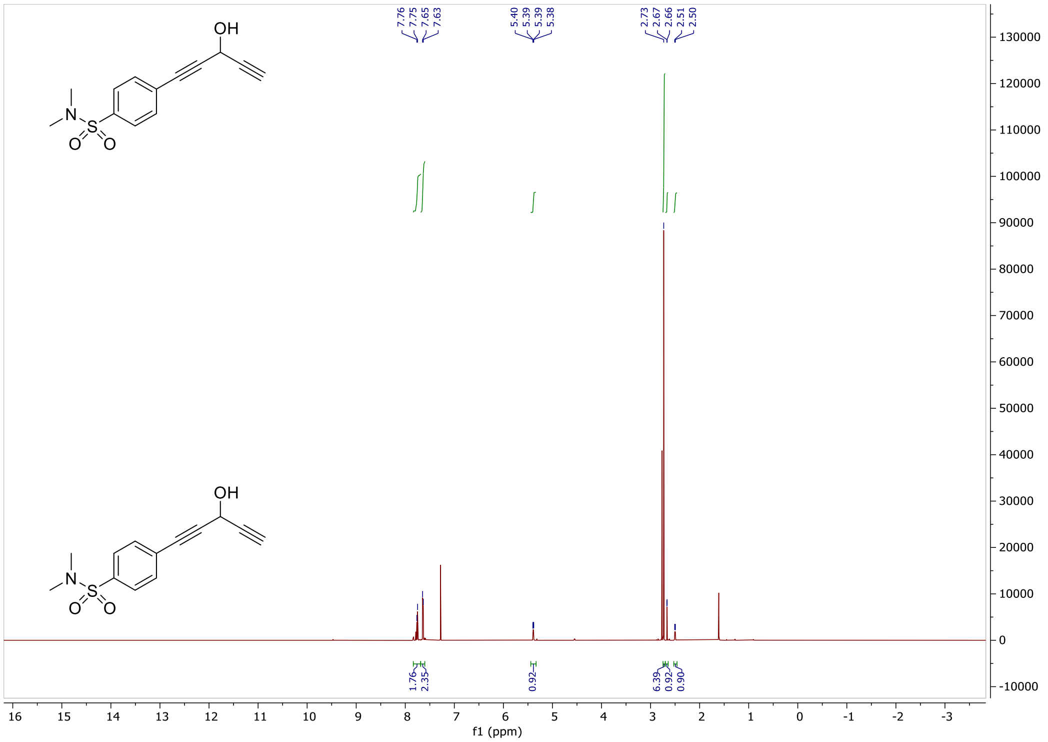

^{13}C NMR (150 MHz, $CDCl_3$) Spectrum of Compound **5H**

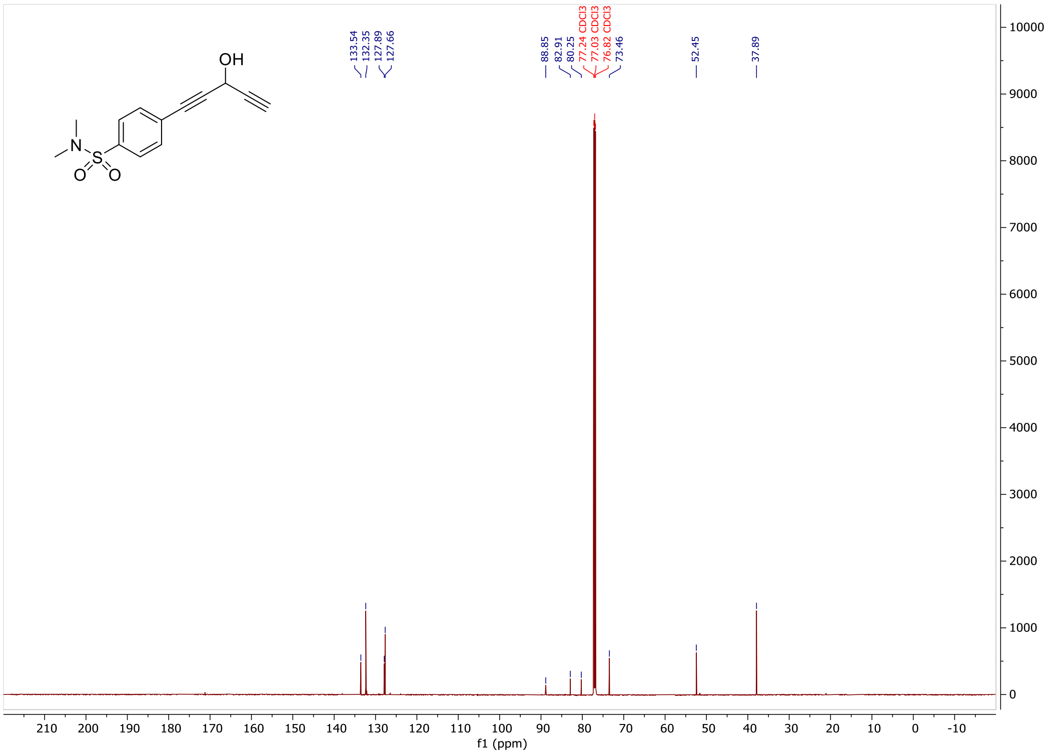

^{1}H NMR (600 MHz, $CDCl_3$) Spectrum of Compound **6C**

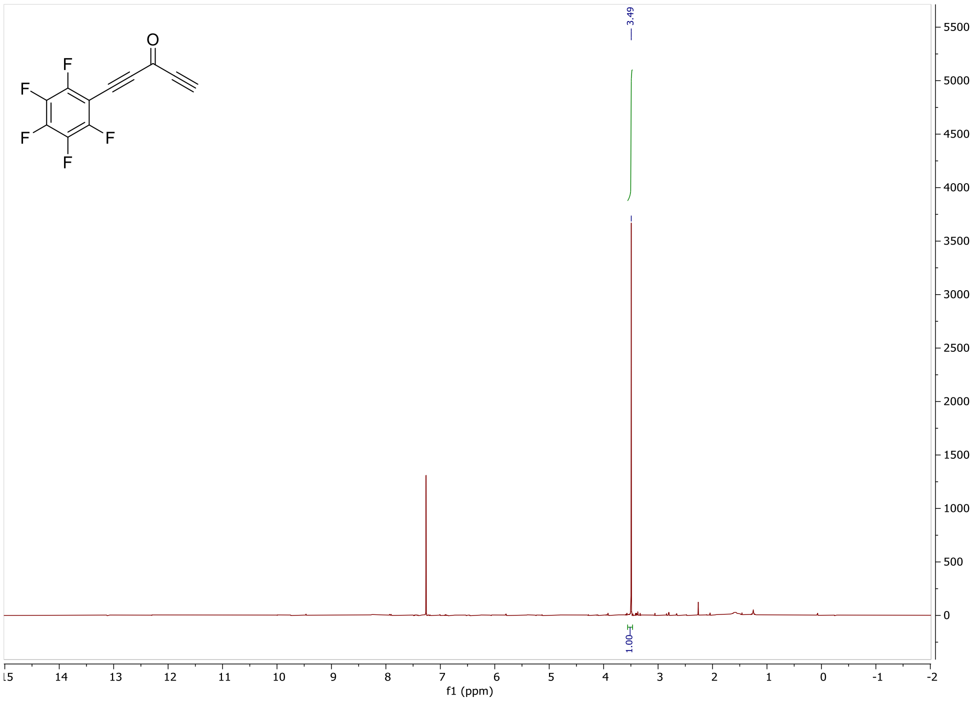

^{13}C NMR (150 MHz, $CDCl_3$) Spectrum of Compound **6C**

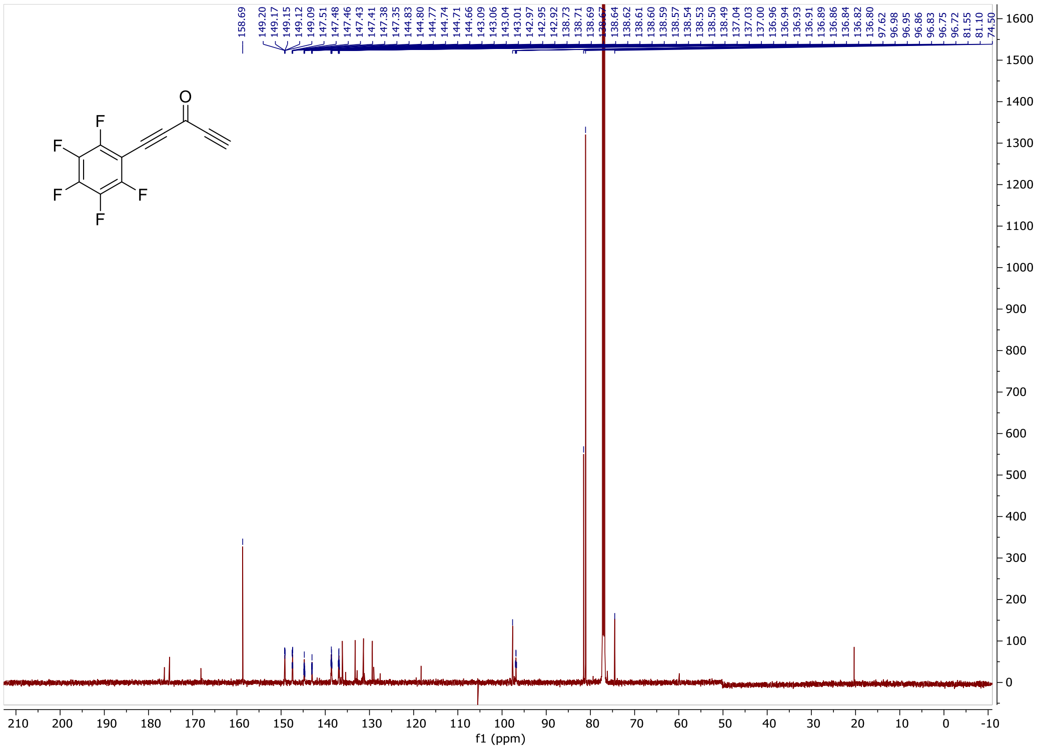

^{19}F NMR (282 MHz, $CDCl_3$) Spectrum of Compound **6C**

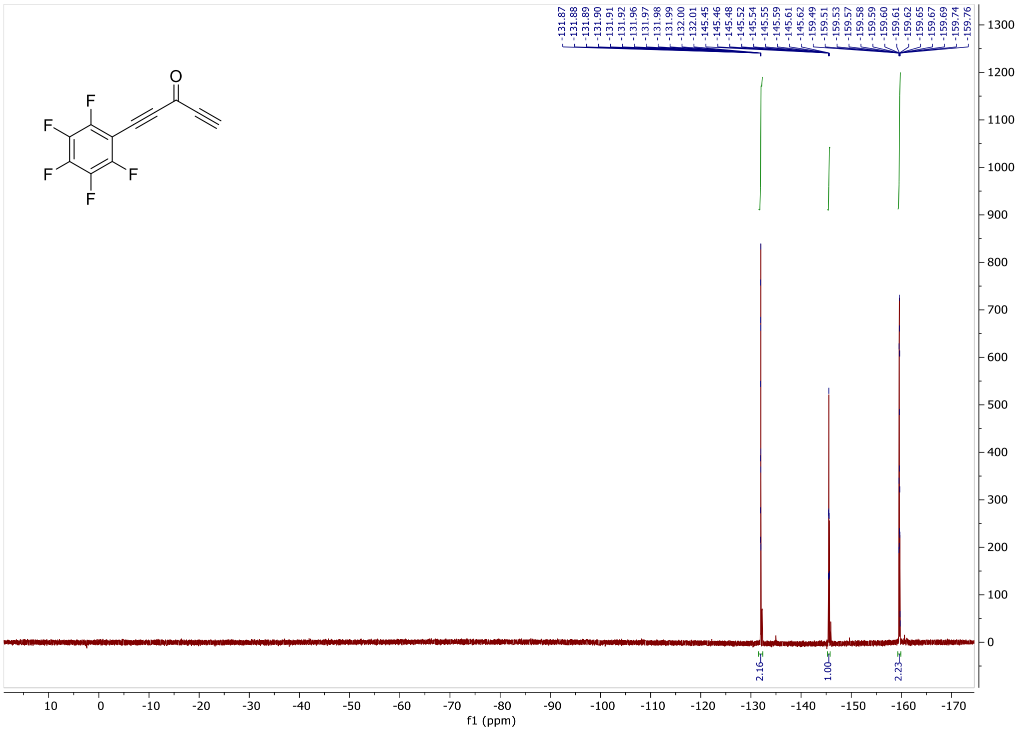

^{1}H NMR (300 MHz, $CDCl_3$) Spectrum of Compound **6E**

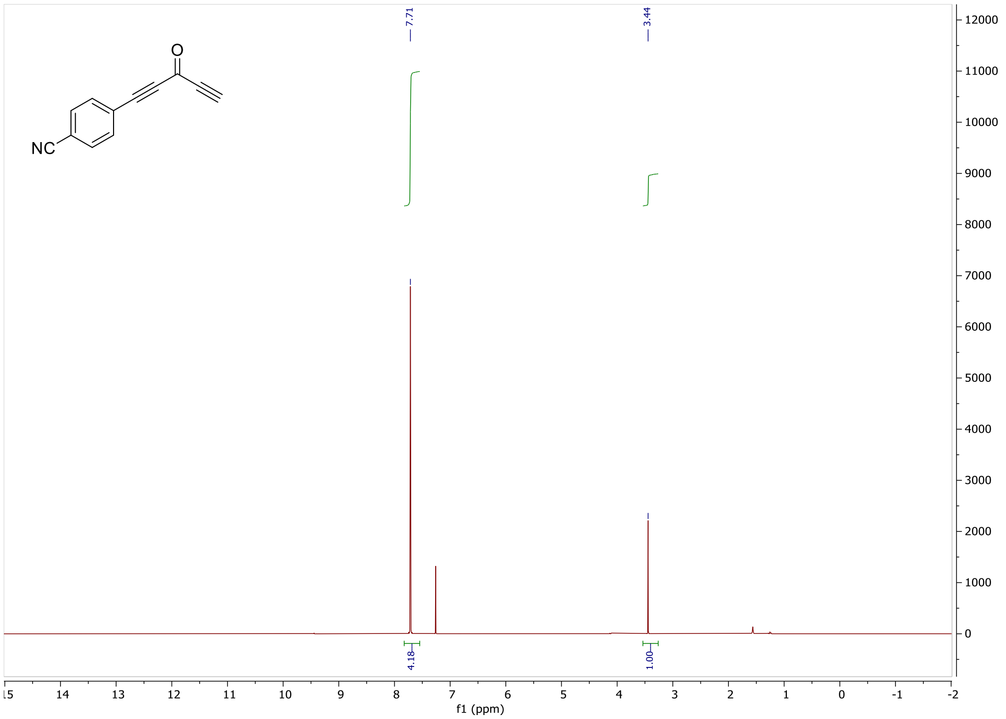

^{13}C NMR (75 MHz, $CDCl_3$) Spectrum of Compound **6E**

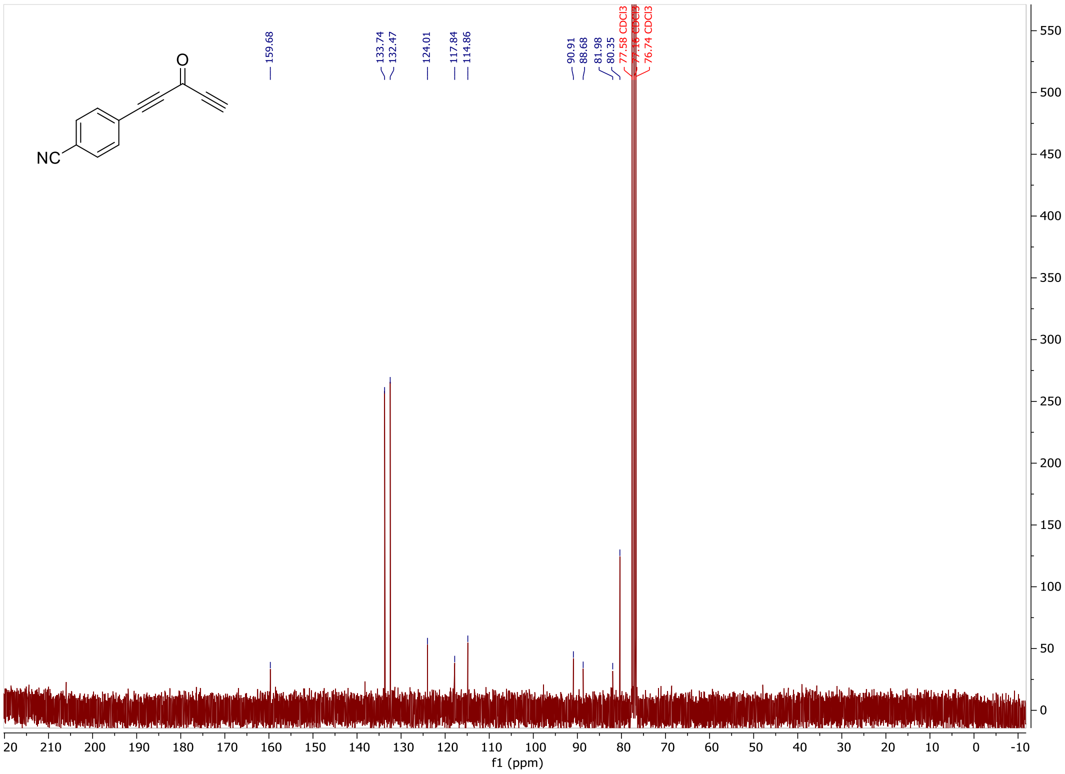

^{1}H NMR (600 MHz, $CDCl_3$) Spectrum of Compound **6F**

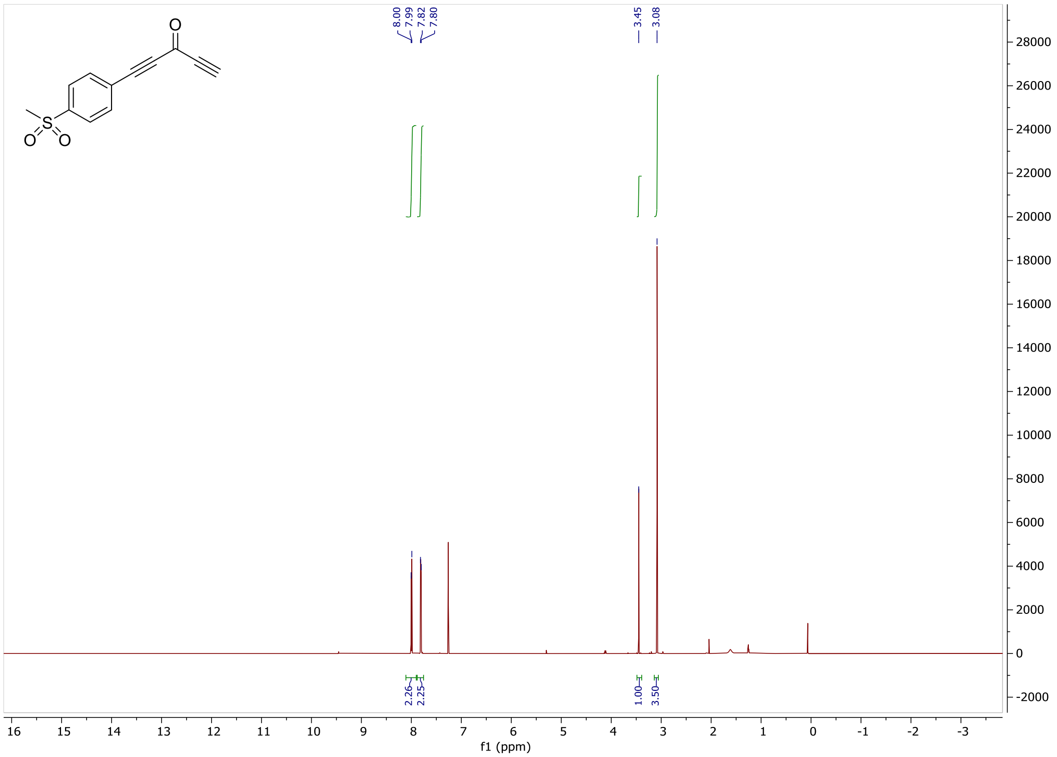

^{13}C NMR (150 MHz, $CDCl_3$) Spectrum of Compound **6F**

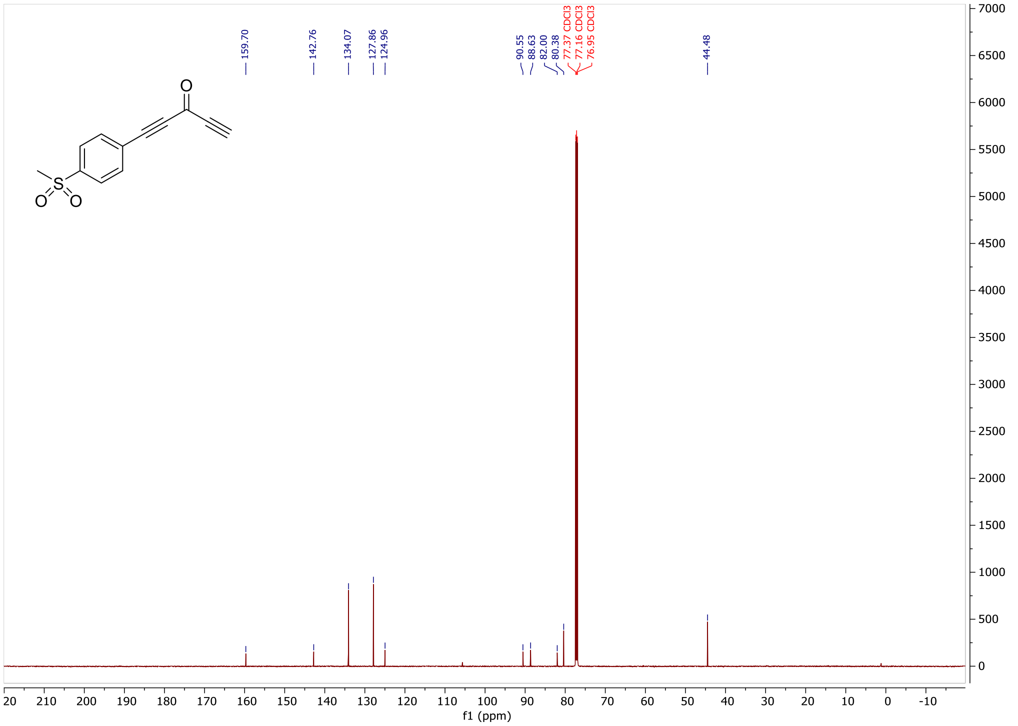

^{1}H NMR (600 MHz, $CDCl_3$) Spectrum of Compound **6G**

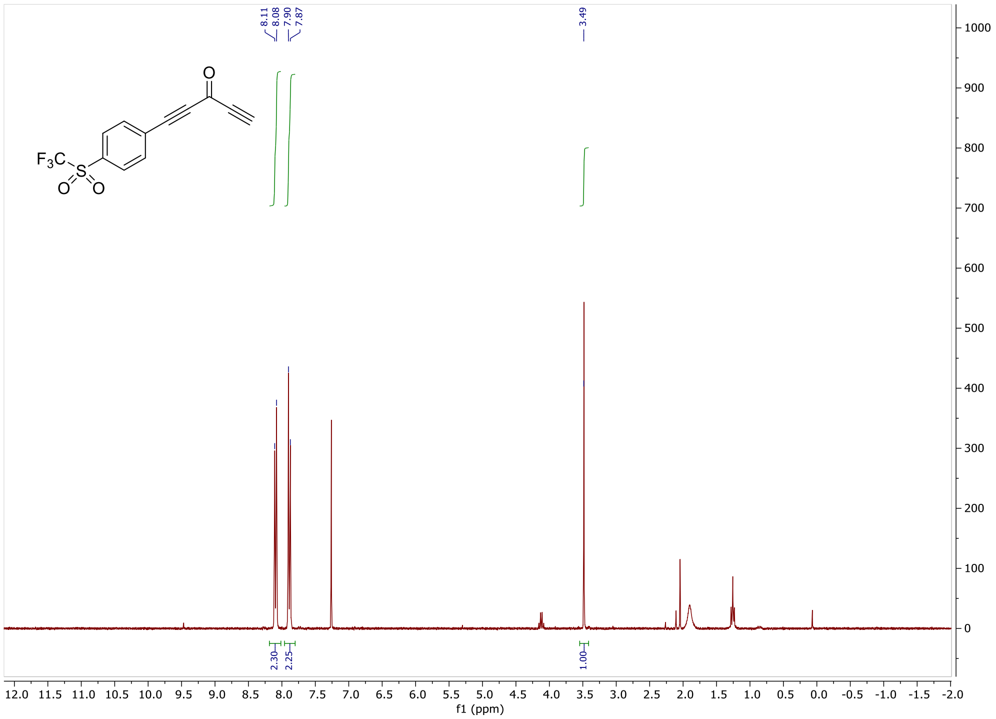

^{13}C NMR (150 MHz, $CDCl_3$) Spectrum of Compound **6G**

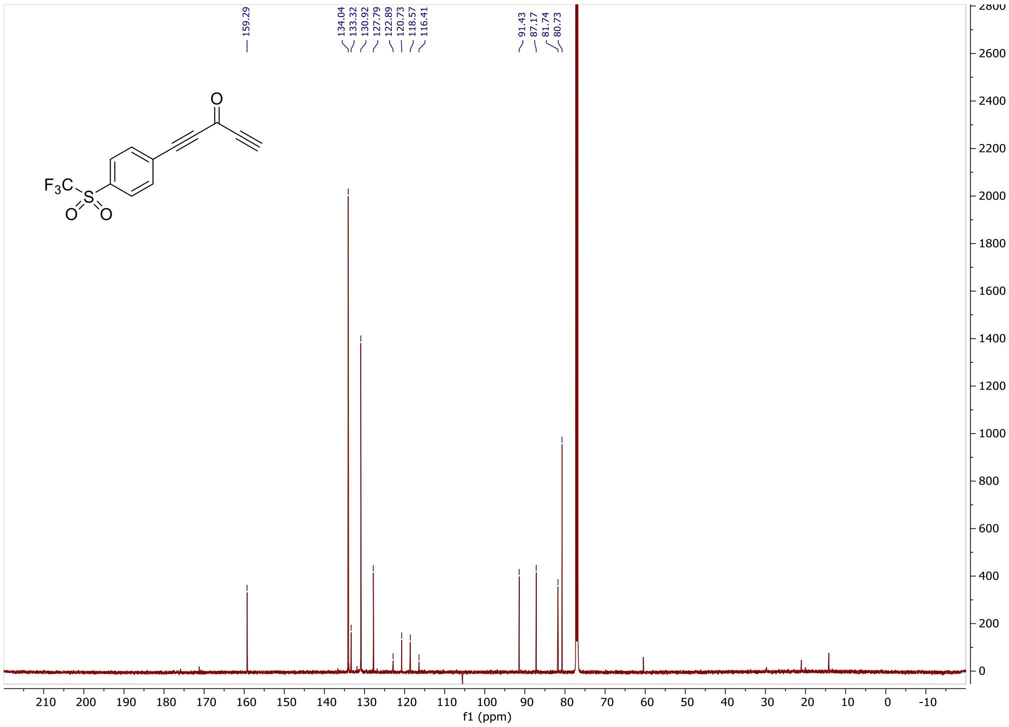

^{19}F NMR (282 MHz, $CDCl_3$) Spectrum of Compound **6G**

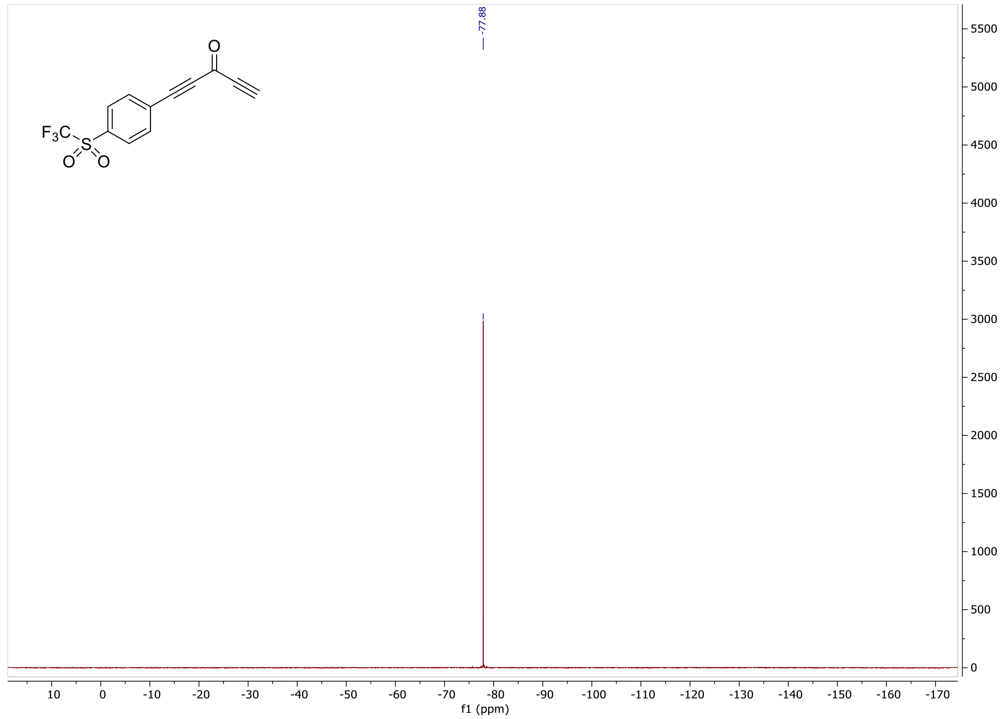

^{1}H NMR (600 MHz, $CDCl_3$) Spectrum of Compound **6H**

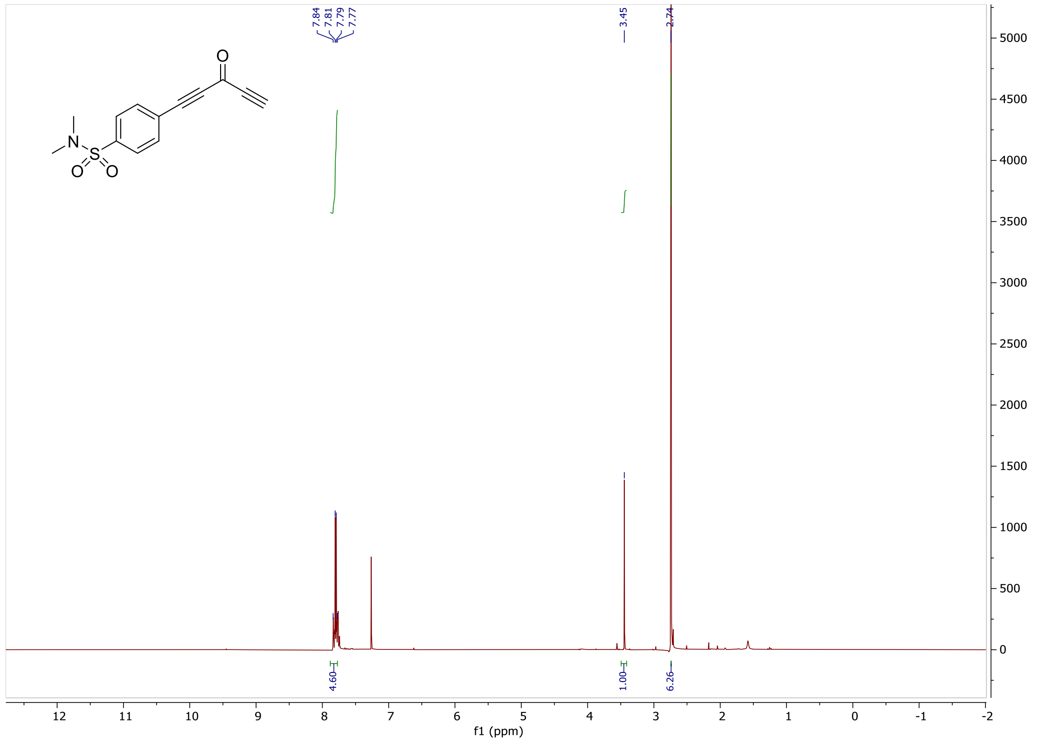

^{13}C NMR (150 MHz, $CDCl_3$) Spectrum of Compound **6H**

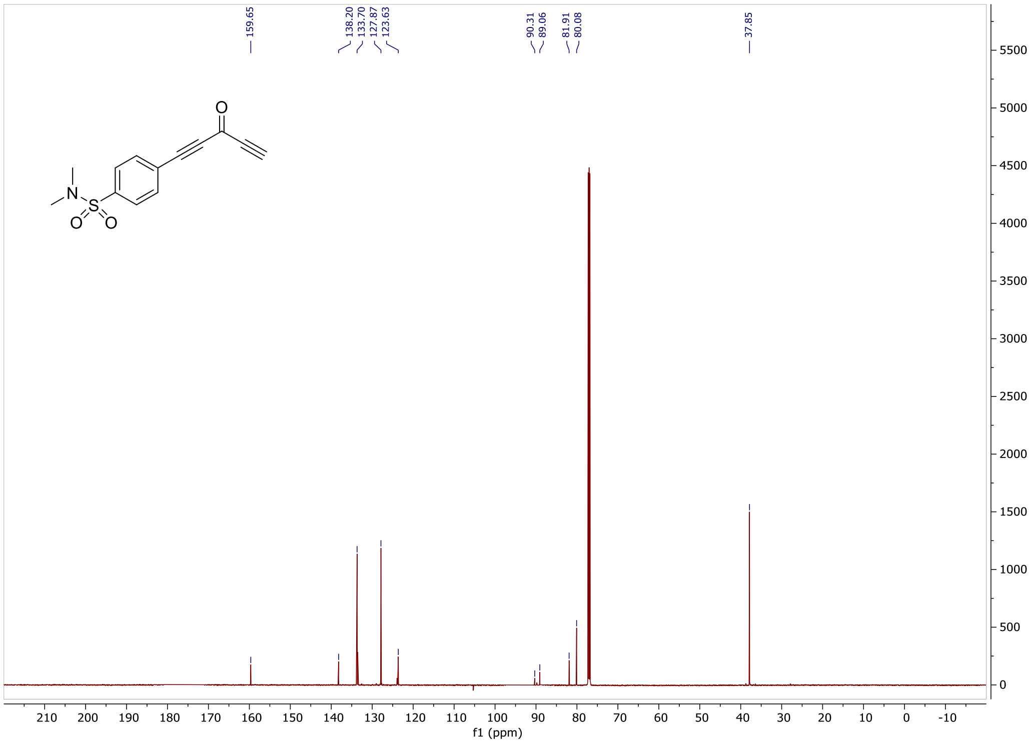

^{1}H NMR (300 MHz, DMSO-d_6) Spectrum of Compound **8A**

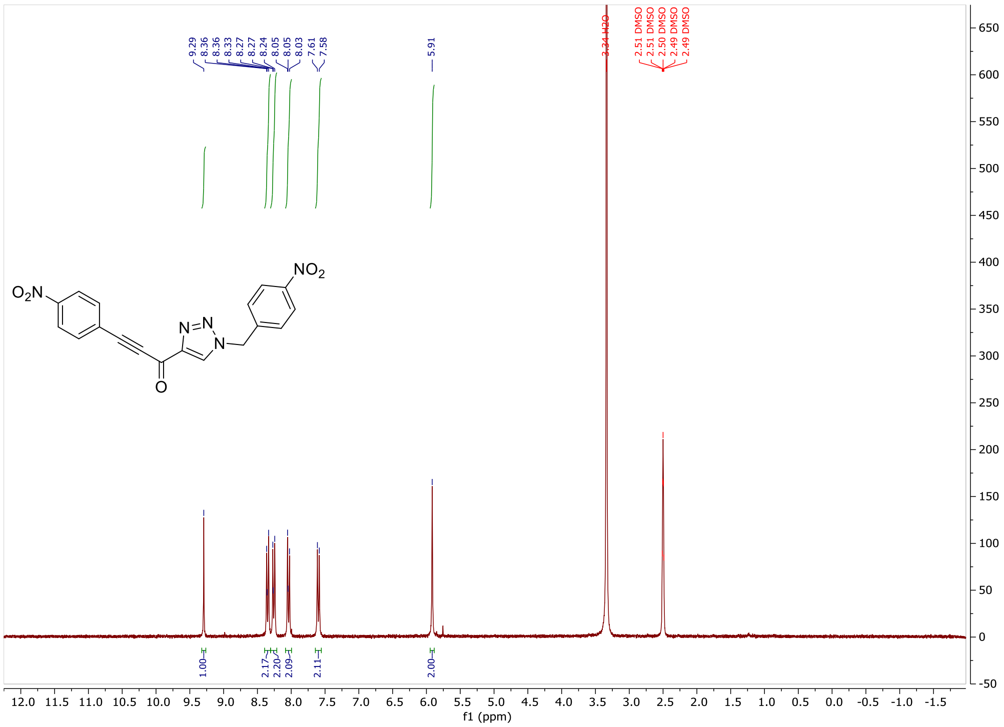

^{13}C NMR (75 MHz, DMSO-d_6) Spectrum of Compound **8A**

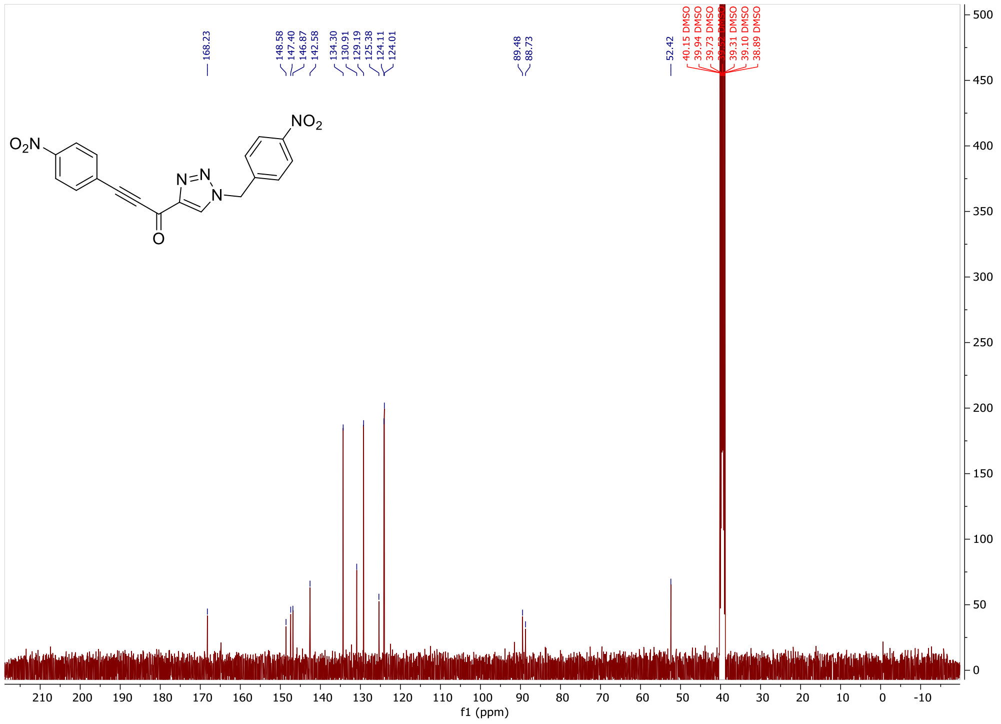

^{1}H NMR (300 MHz, $CDCl_3$) Spectrum of Compound **8B**

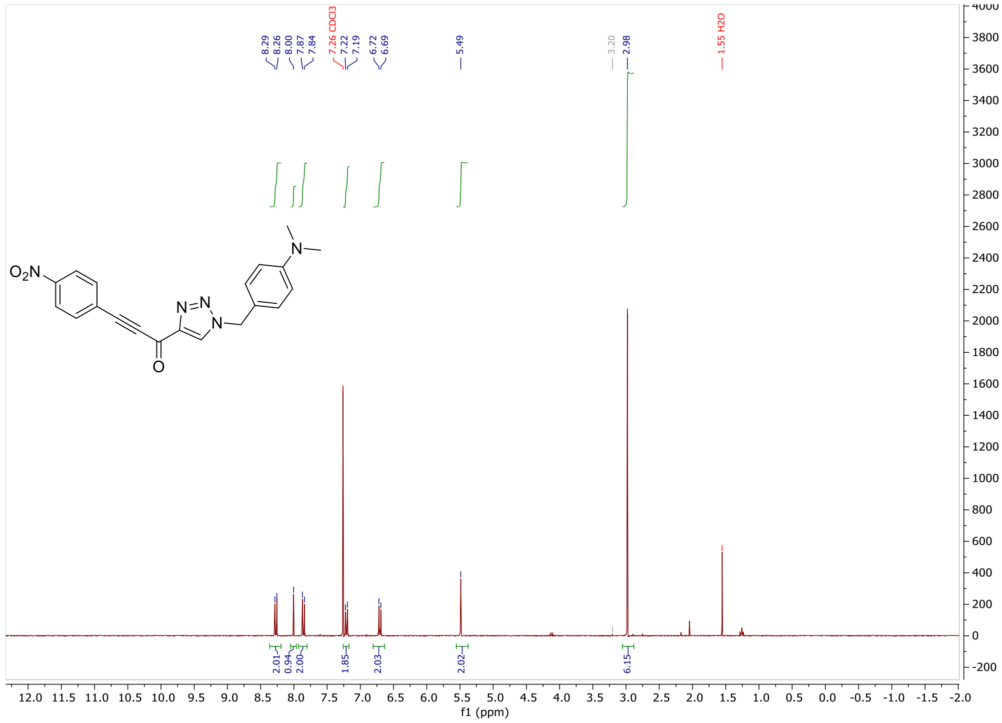

^{13}C NMR (75 MHz, $CDCl_3$) Spectrum of Compound **8B**

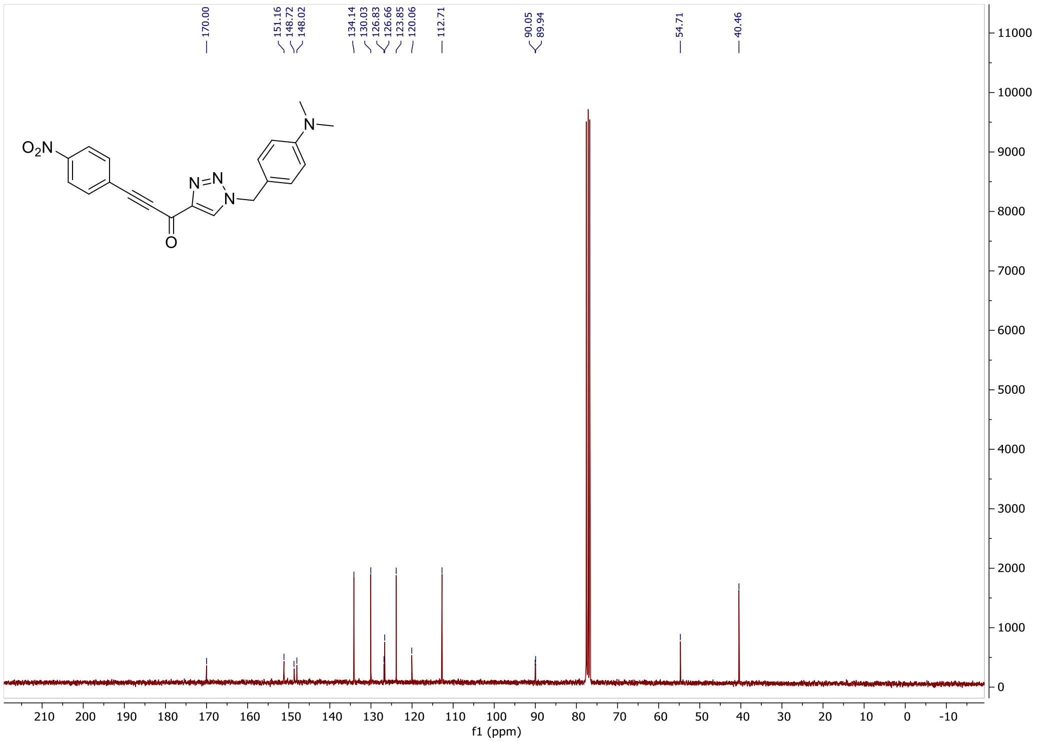

^{1}H NMR (300 MHz, $CDCl_3$) Spectrum of Compound **8C**

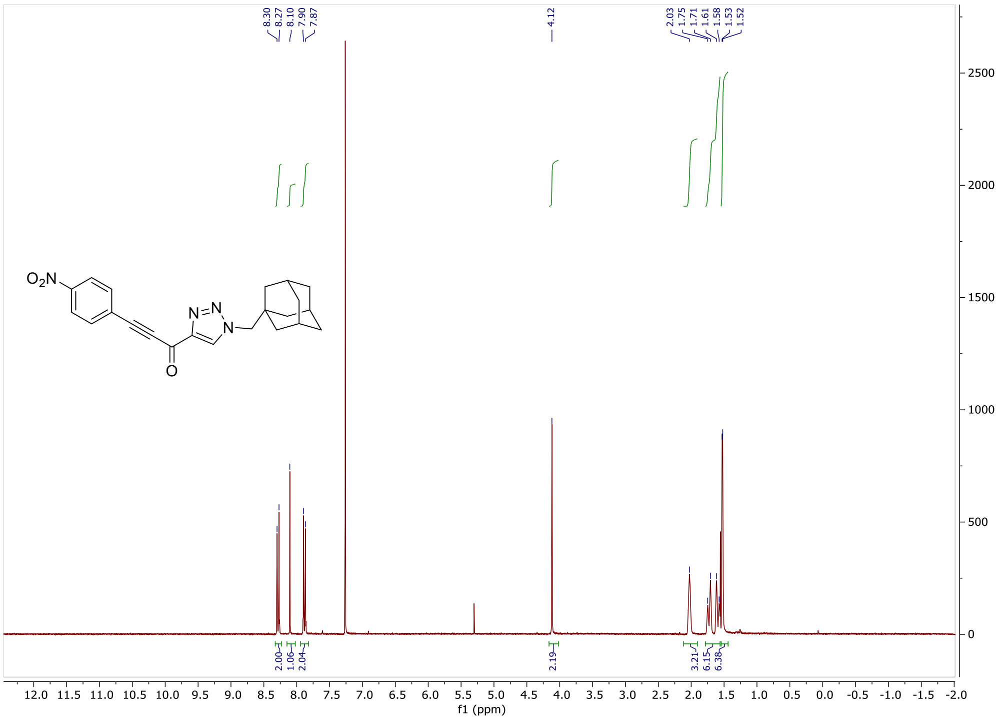

^{13}C NMR (75 MHz, $CDCl_3$) Spectrum of Compound **8C**

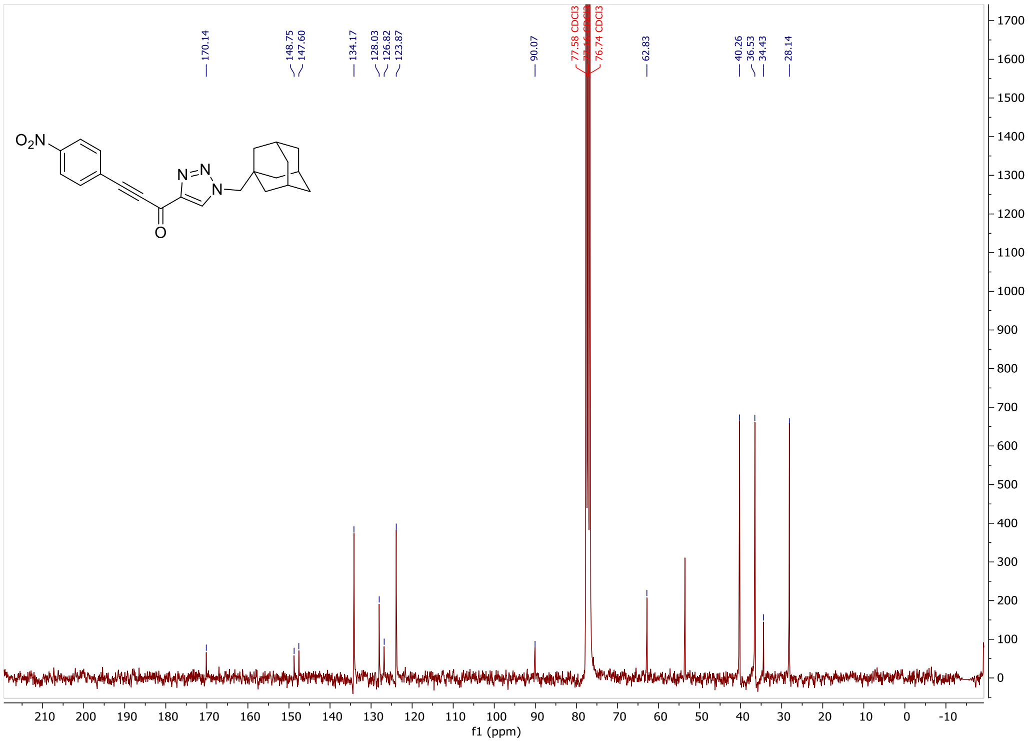

^{1}H NMR (300 MHz, $CDCl_3$) Spectrum of Compound **8D**

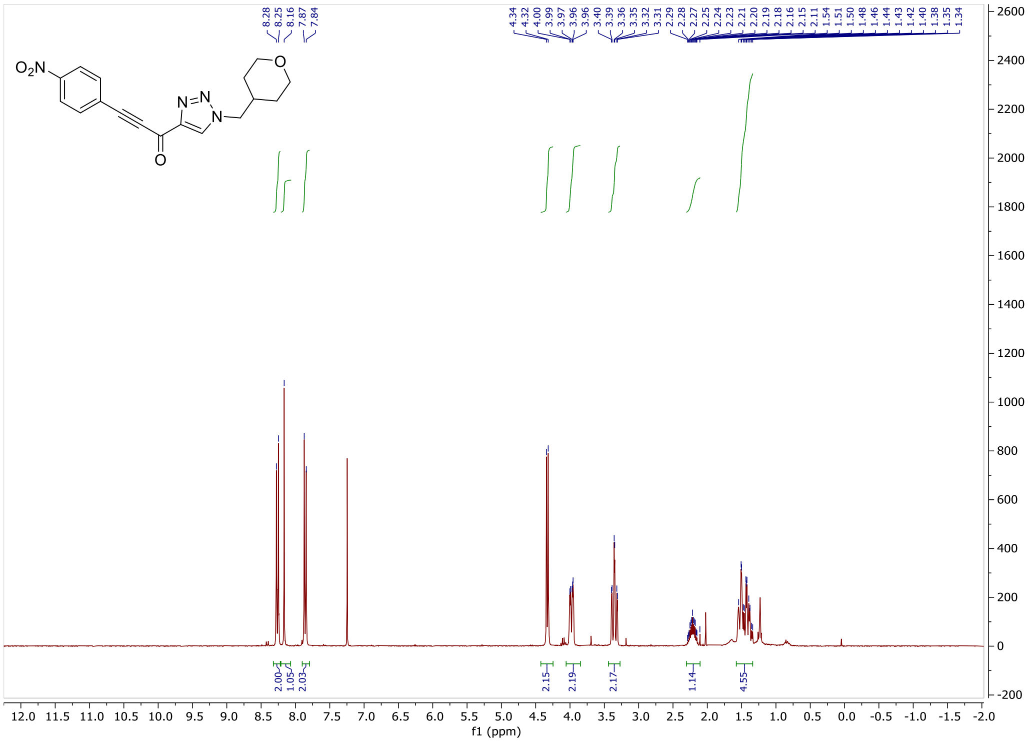

^{13}C NMR (75 MHz, $CDCl_3$) Spectrum of Compound **8D**

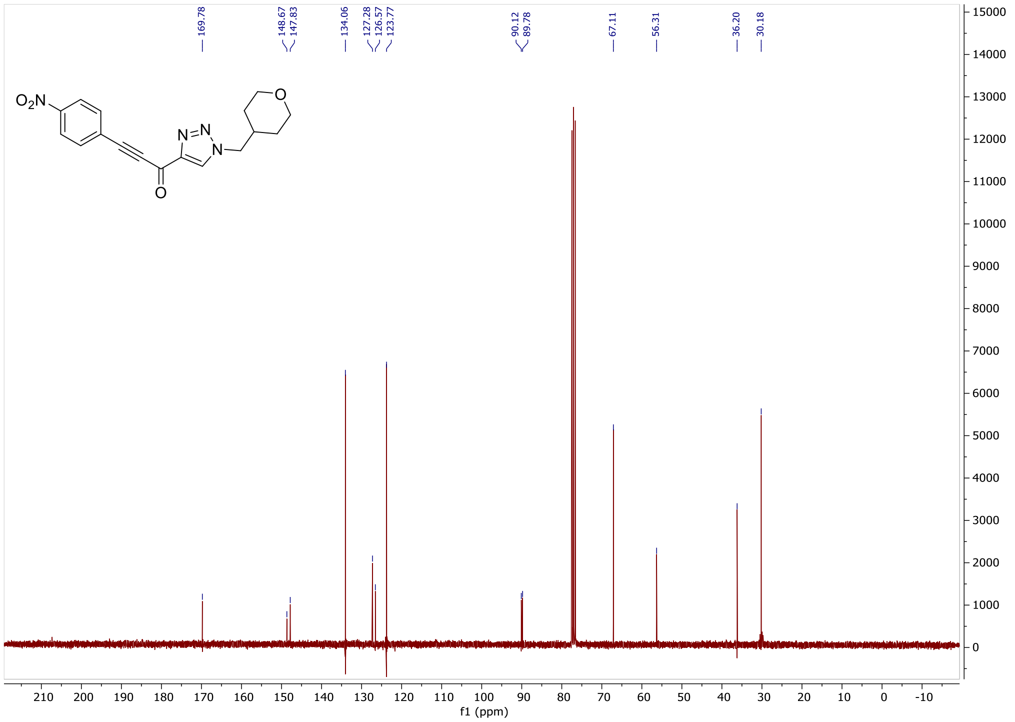

^{1}H NMR (300 MHz, $CDCl_3$) Spectrum of Compound **8E**

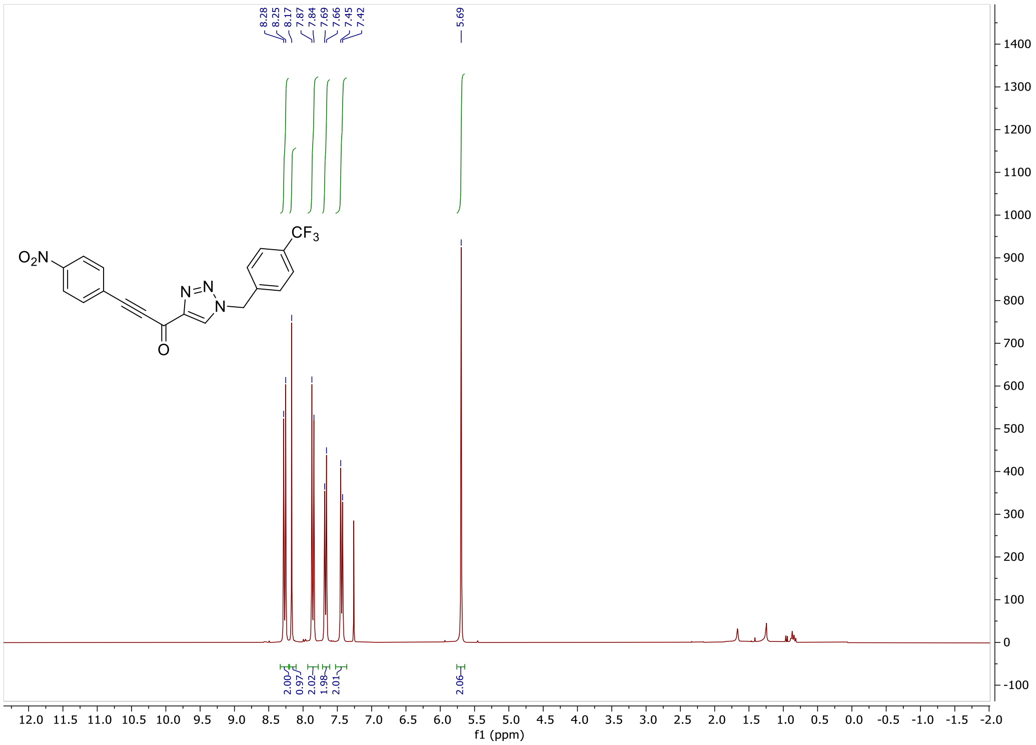

^{13}C NMR (75 MHz, $CDCl_3$) Spectrum of Compound **8E**

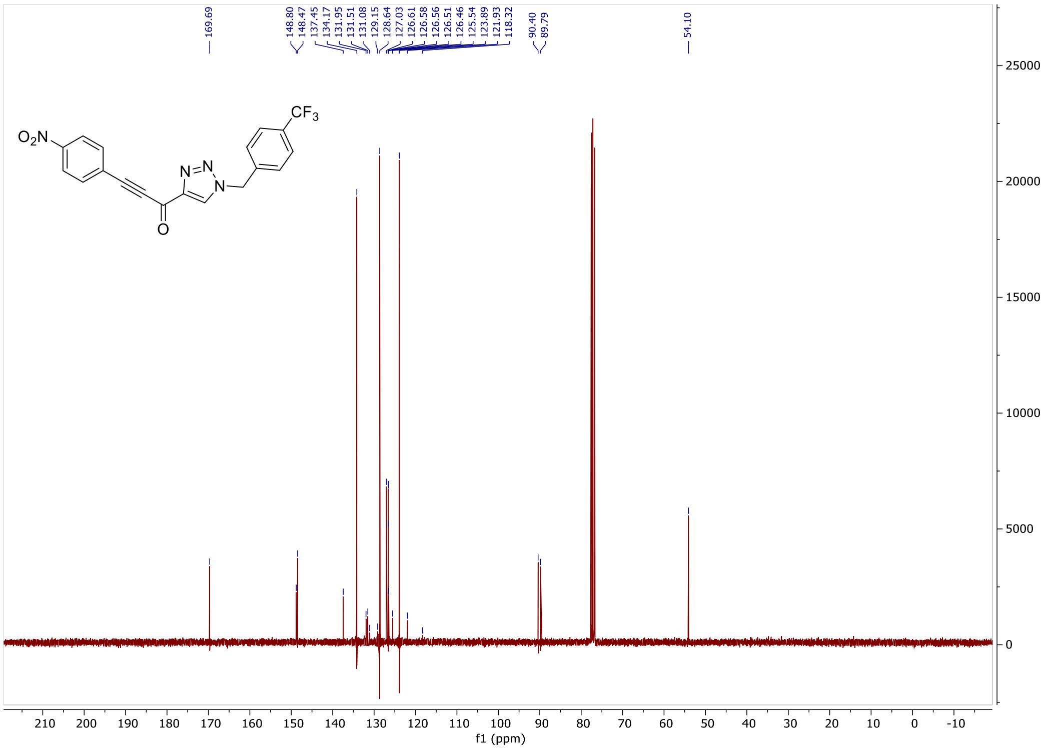

^{19}F NMR (282 MHz, $CDCl_3$) Spectrum of Compound **8E**

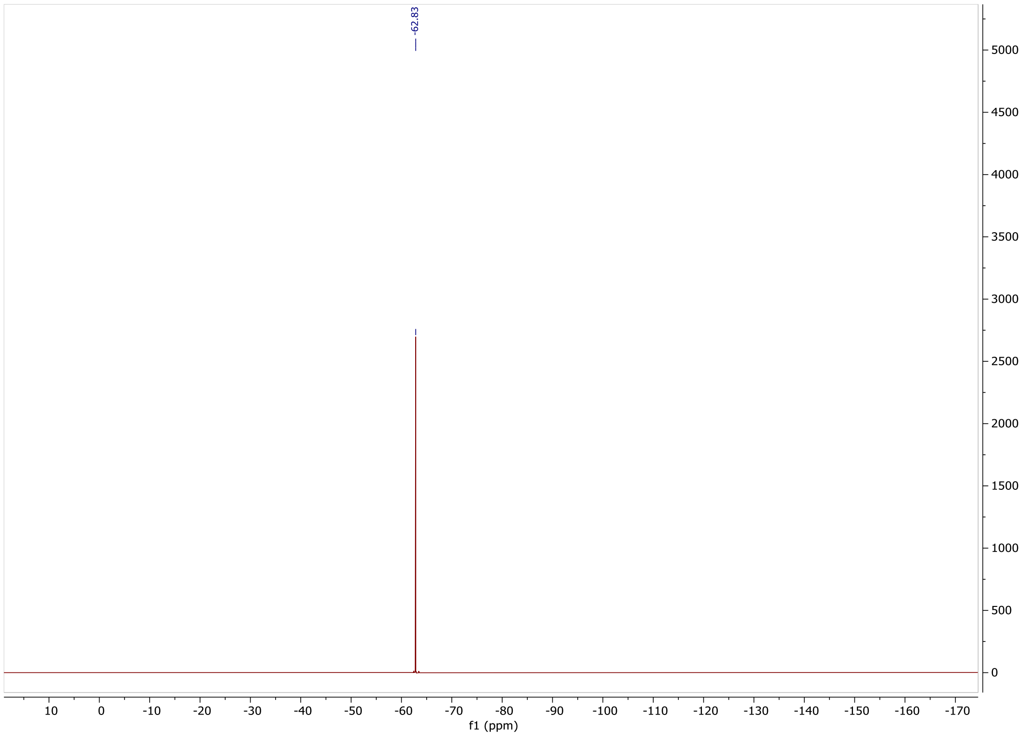

^{1}H NMR (300 MHz, $CDCl_3$) Spectrum of Compound **8F**

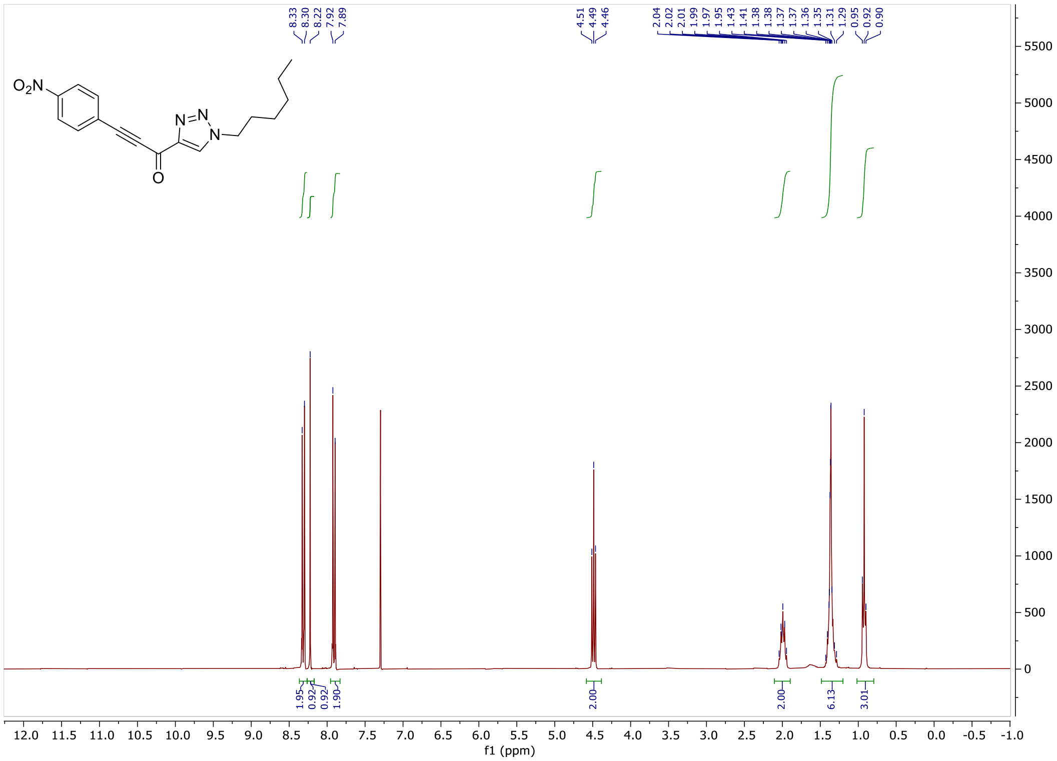

^{13}C NMR (75 MHz, $CDCl_3$) Spectrum of Compound **8F**

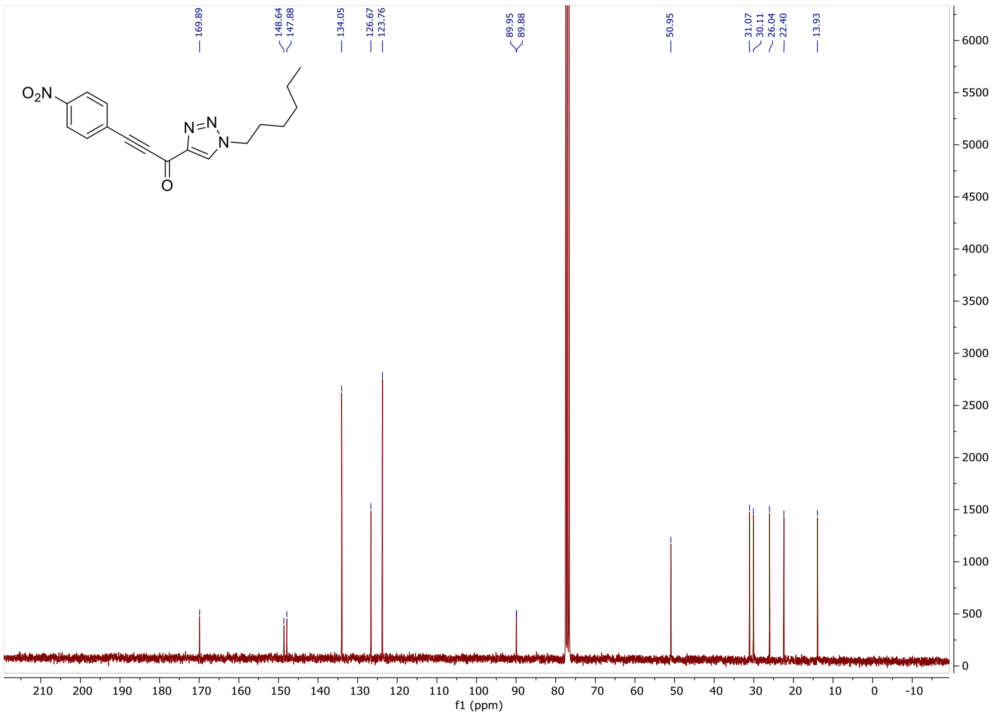

^{1}H NMR (300 MHz, DMSO-d_6) Spectrum of Compound **8G**

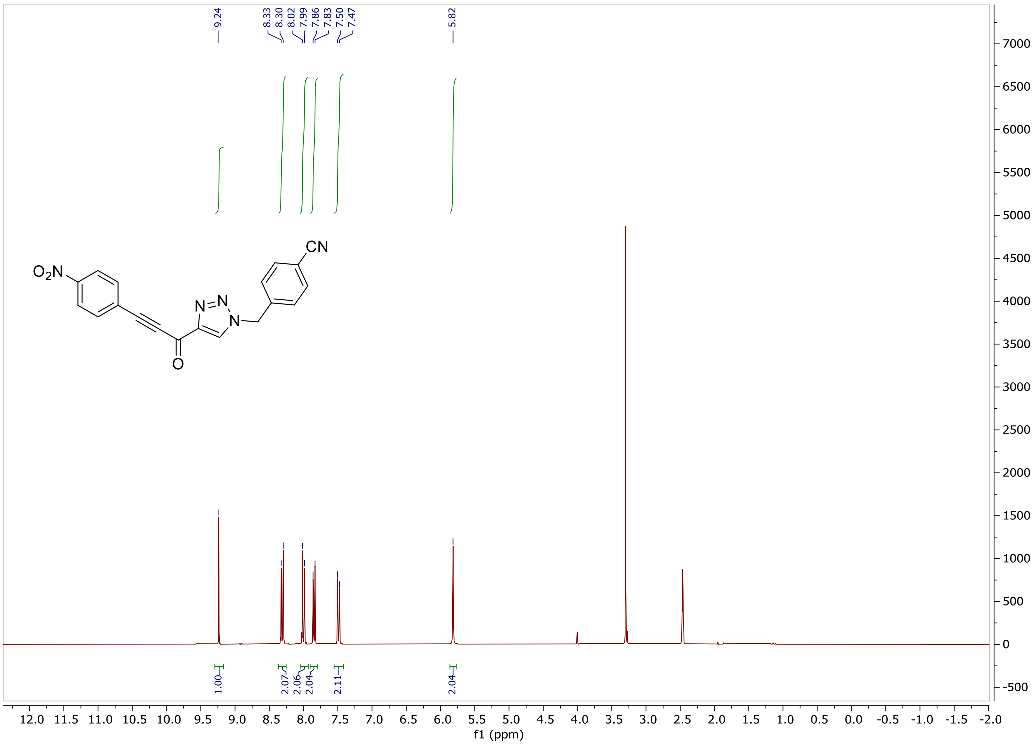

^{13}C NMR (75 MHz, DMSO-d_6) Spectrum of Compound **8G**

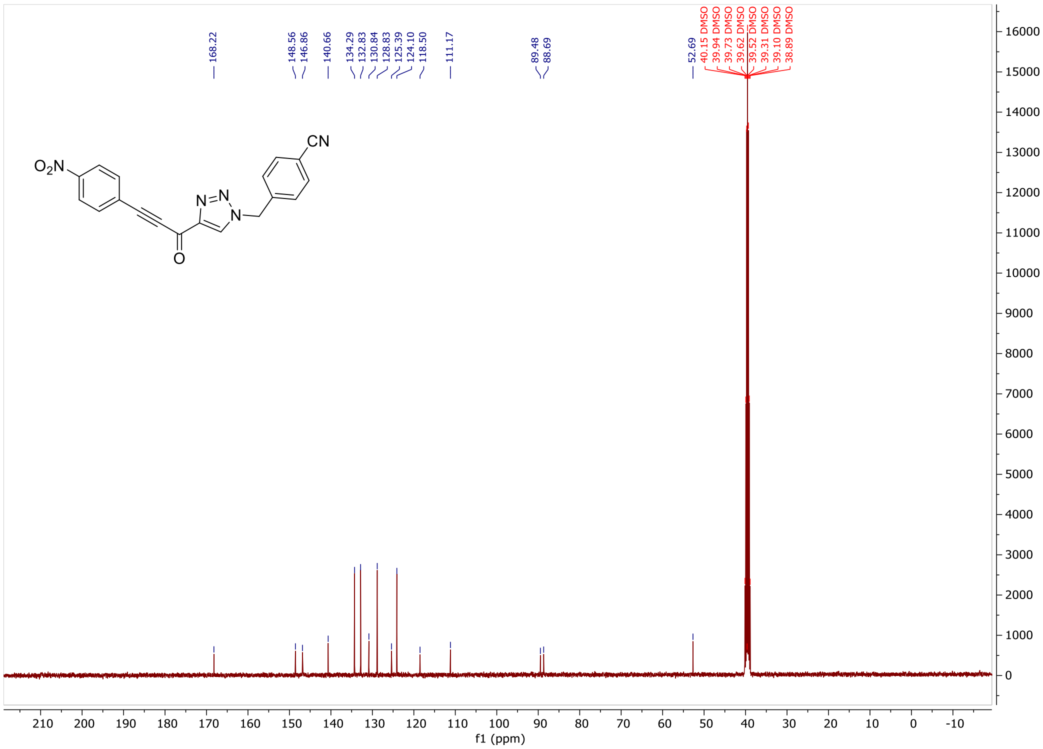

^1H NMR (400 MHz, $CDCl_3$) Spectrum of Compound **8H**

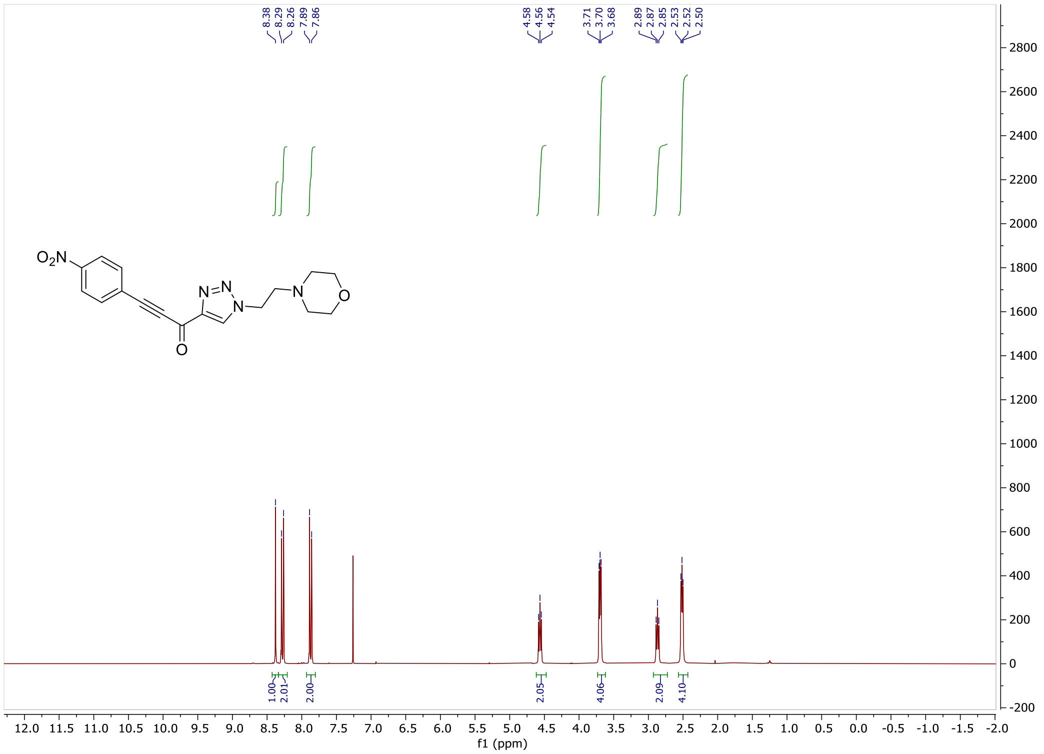

^{13}C NMR (100 MHz, $CDCl_3$) Spectrum of Compound **8H**

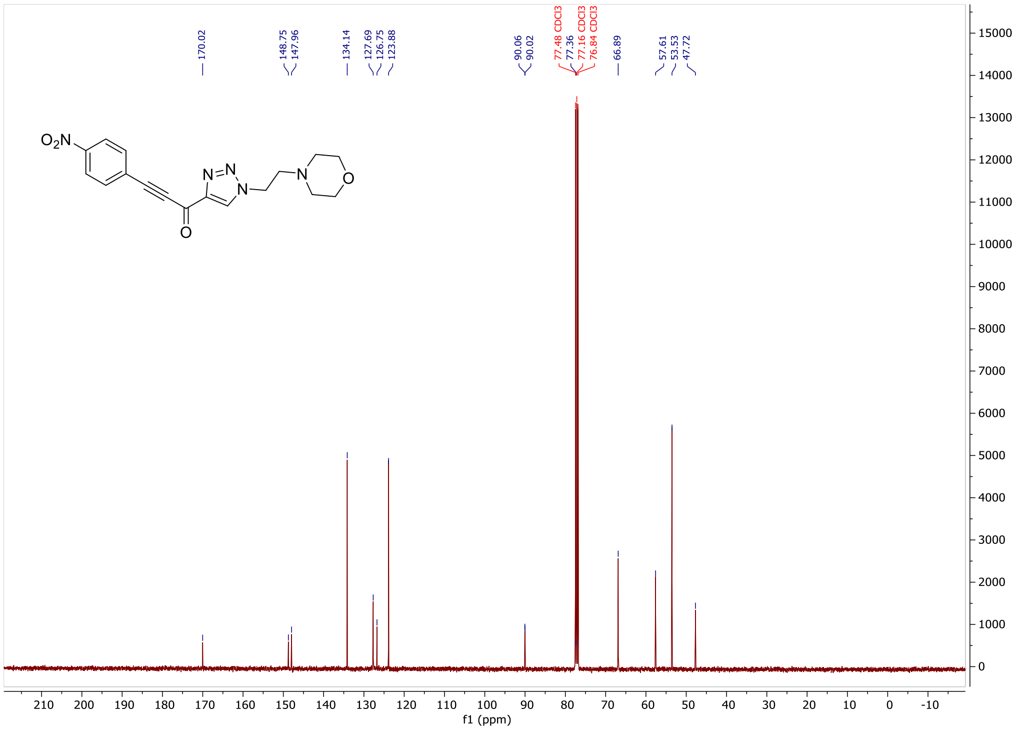

^{1}H NMR (400 MHz, $CDCl_3$) Spectrum of Compound **8l**

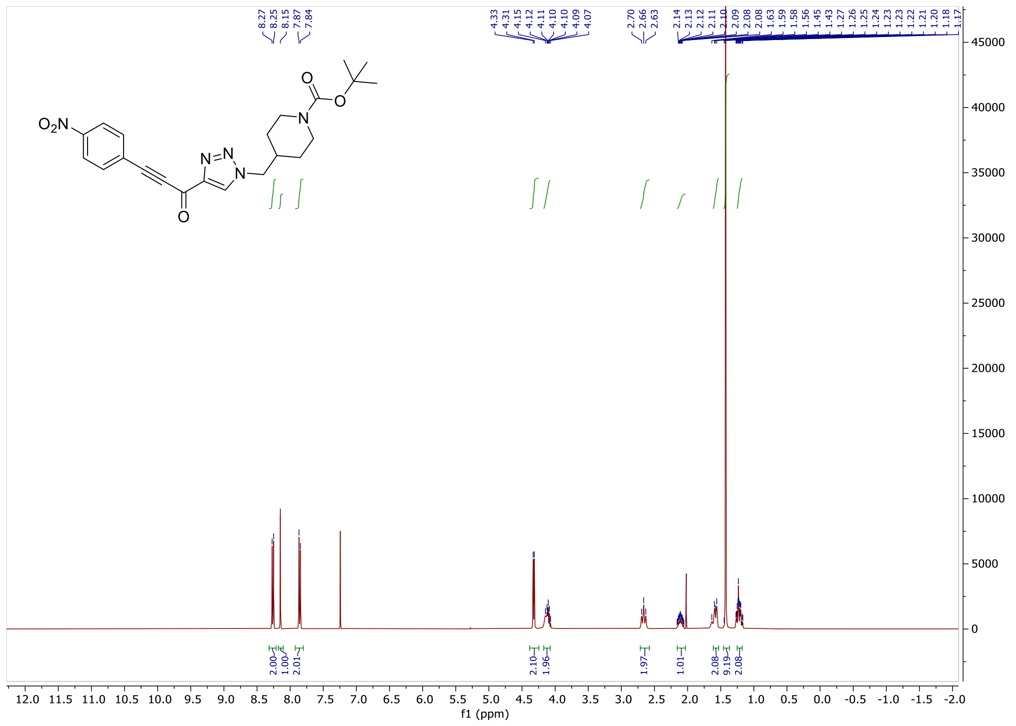

^{13}C NMR (100 MHz, $CDCl_3$) Spectrum of Compound **8l**

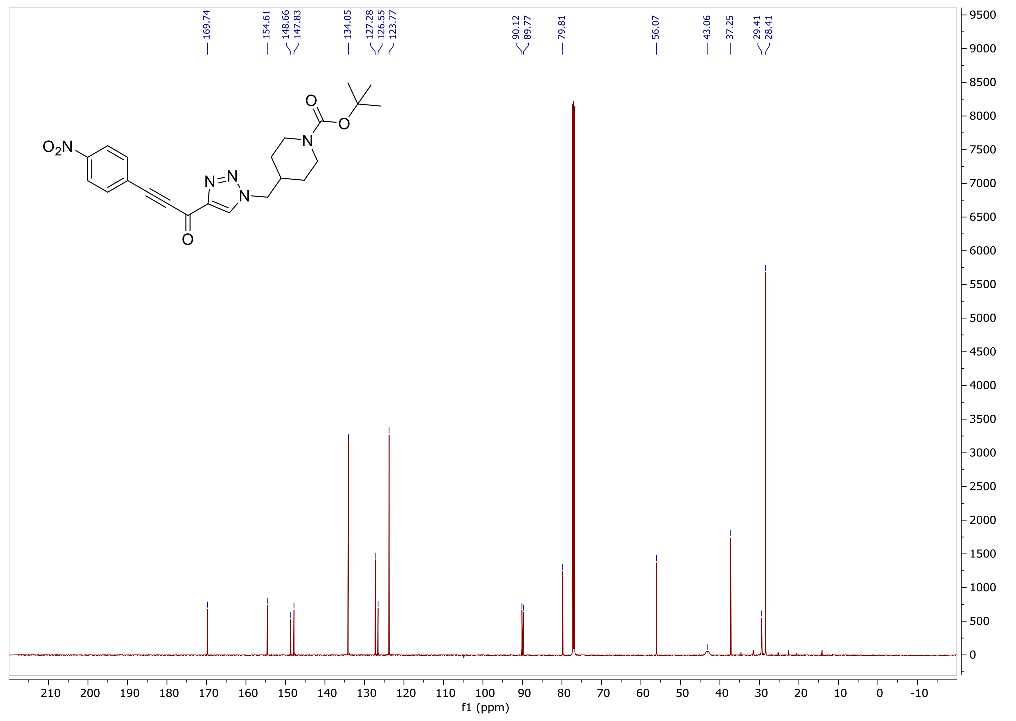

^{1}H NMR (400 MHz, $CDCl_3$) Spectrum of Compound **8J**

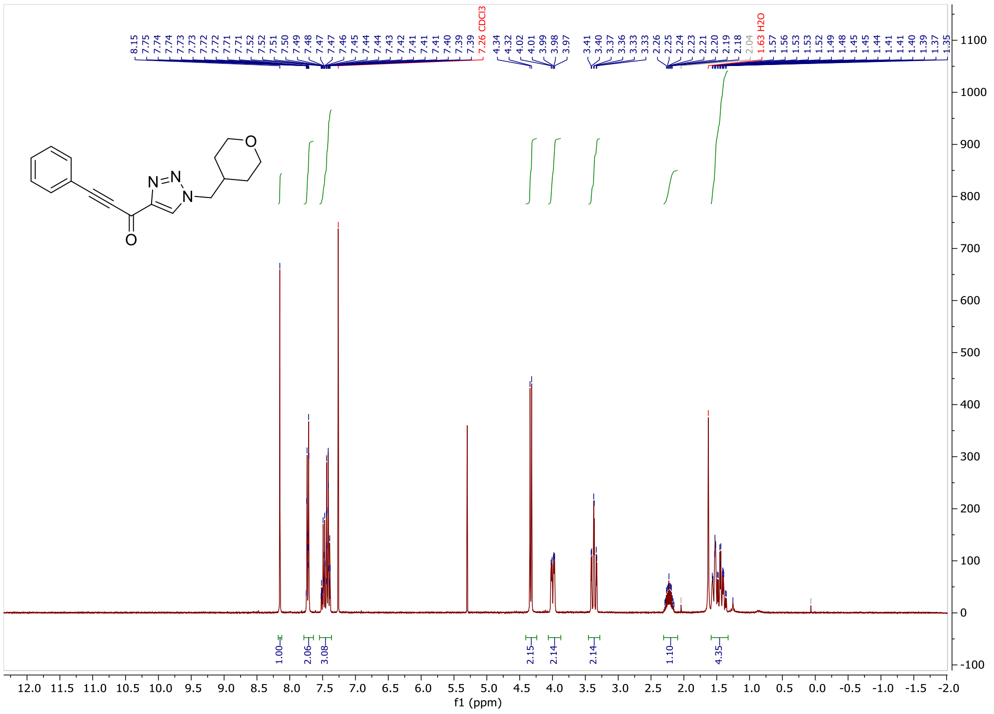

^{13}C NMR (100 MHz, $CDCl_3$) Spectrum of Compound **8J**

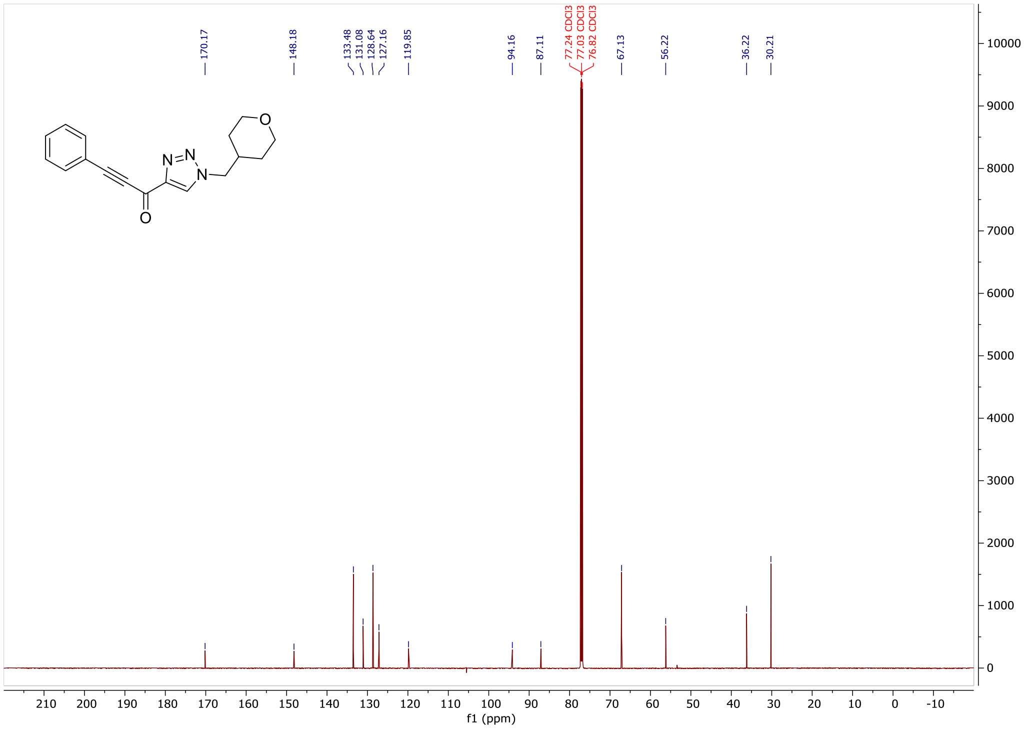

^{1}H NMR (600 MHz, $CDCl_3$) Spectrum of Compound **8K**

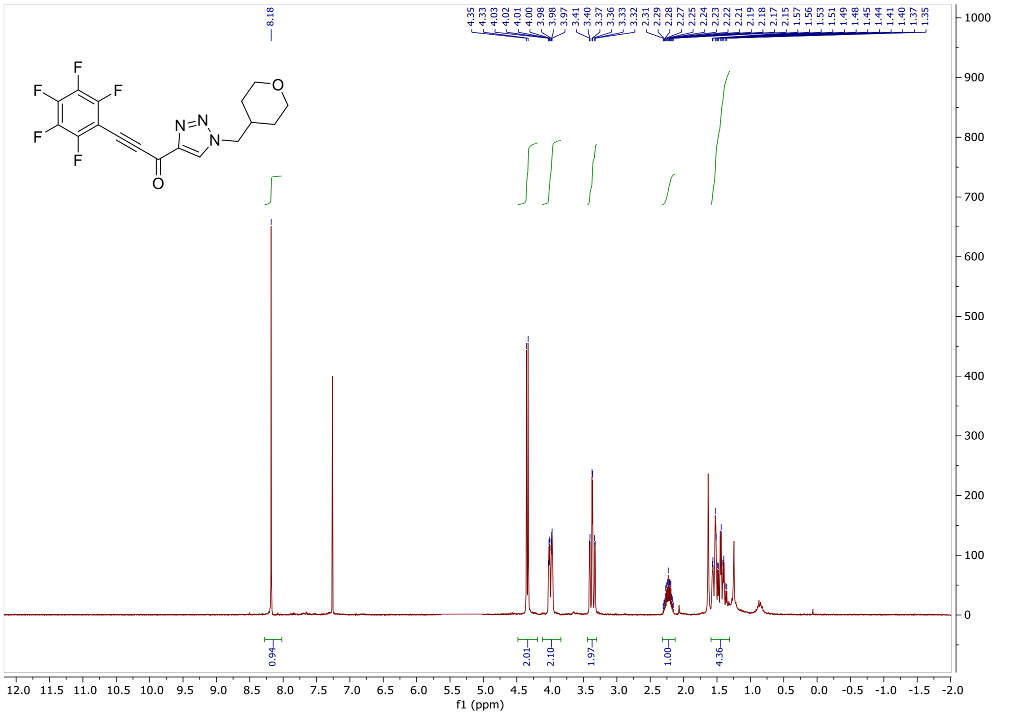

^{13}C NMR (150 MHz, $CDCl_3$) Spectrum of Compound **8K**

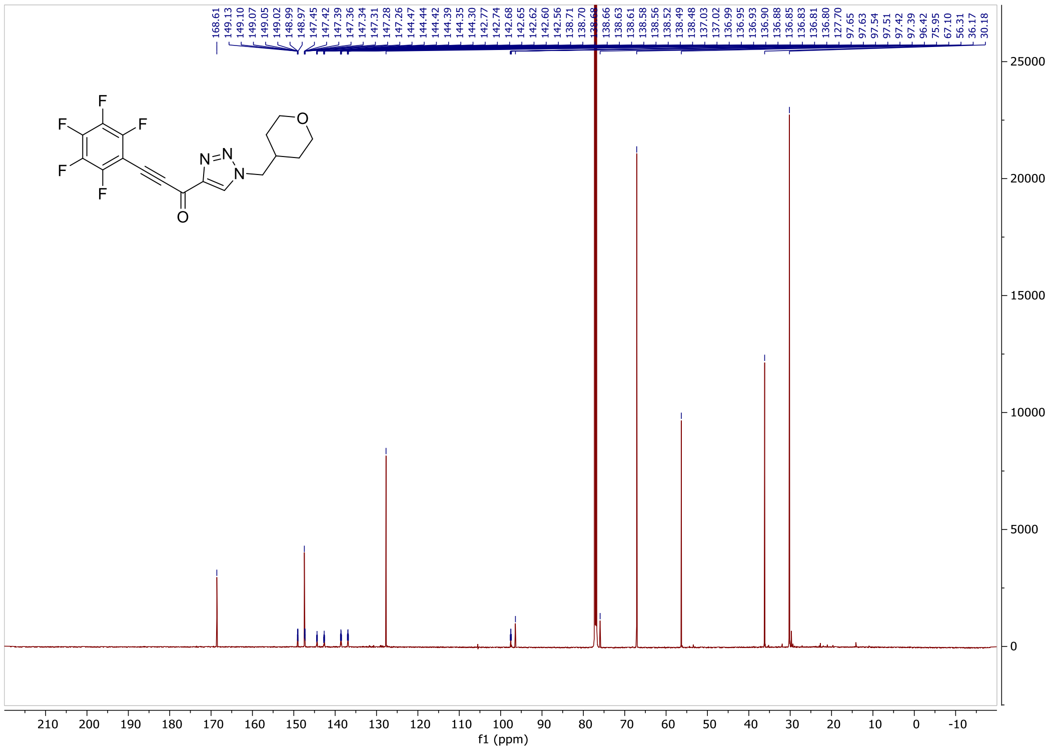

^{19}F NMR (282 MHz, $CDCl_3$) Spectrum of Compound **8K**

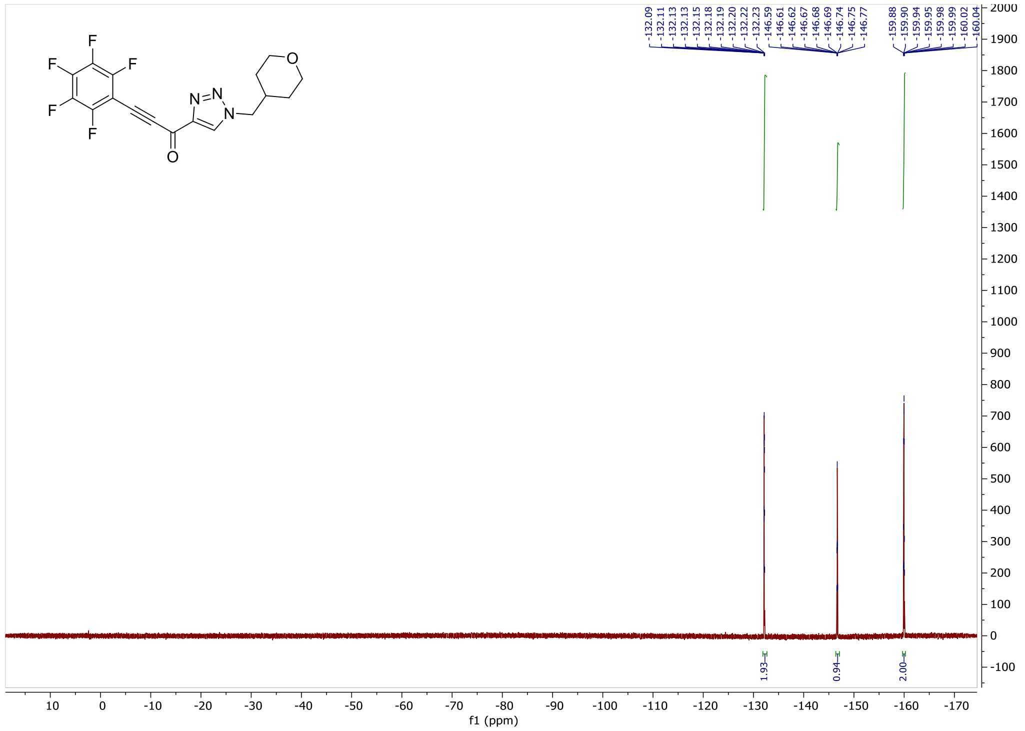

^{1}H NMR (400 MHz, $CDCl_3$) Spectrum of Compound **8L**

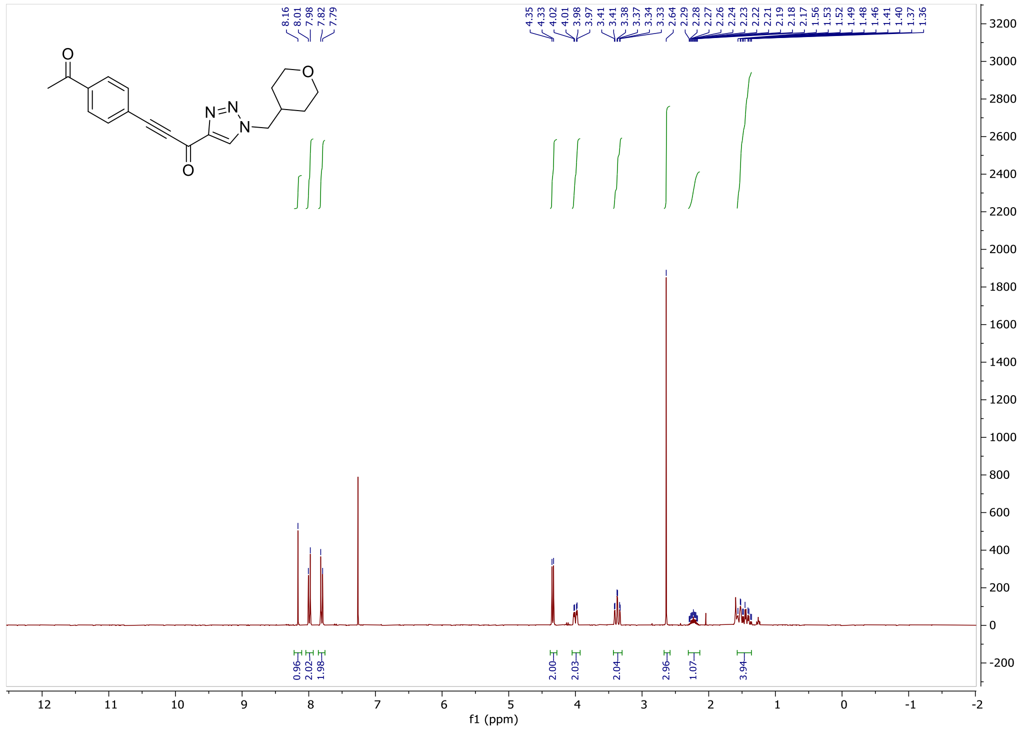

^{13}C NMR (100 MHz, $CDCl_3$) Spectrum of Compound **8L**

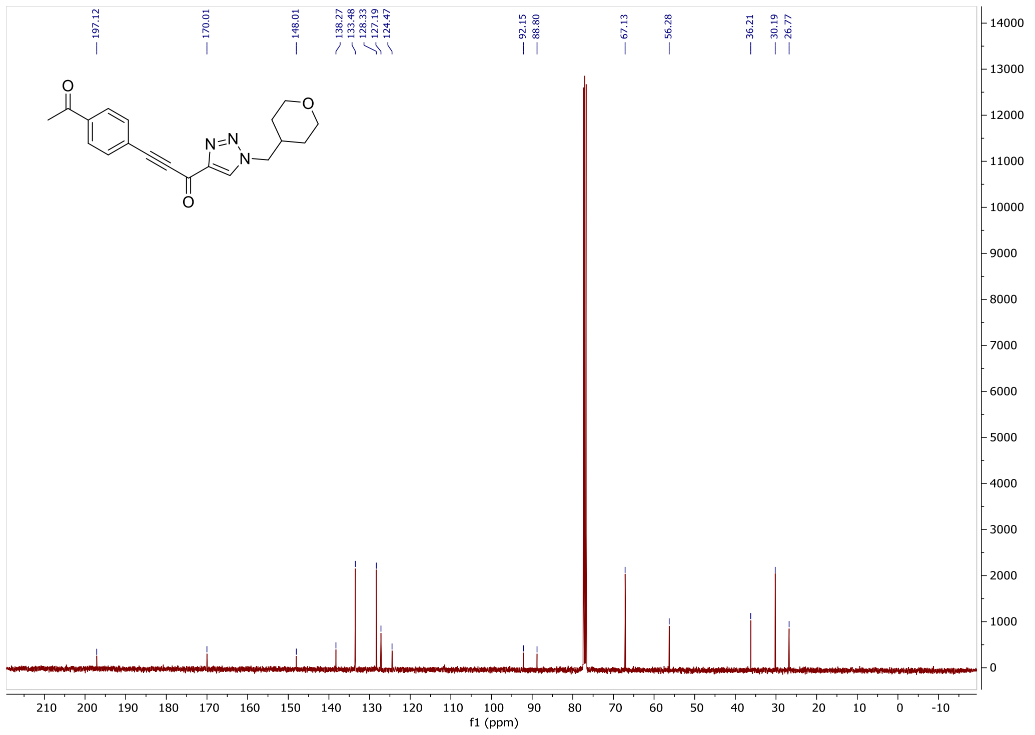

^{1}H NMR (600 MHz, $CDCl_3$) Spectrum of Compound **8M**

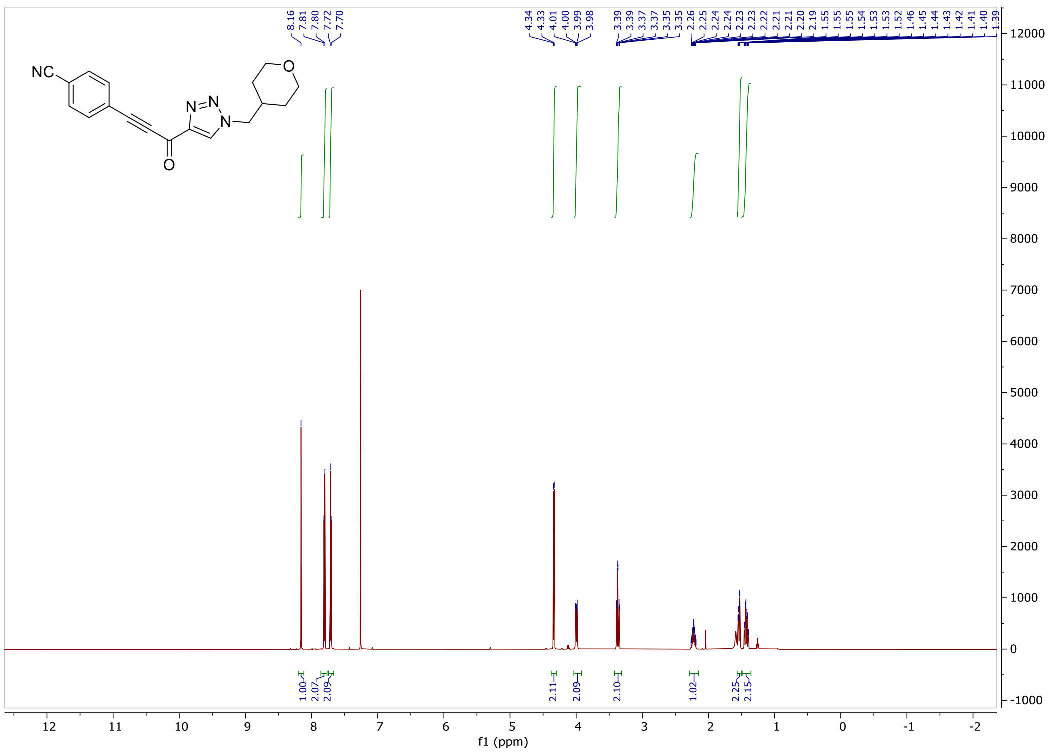

[13] C NMR (150 MHz, $CDCl_3$) Spectrum of Compound **8M**

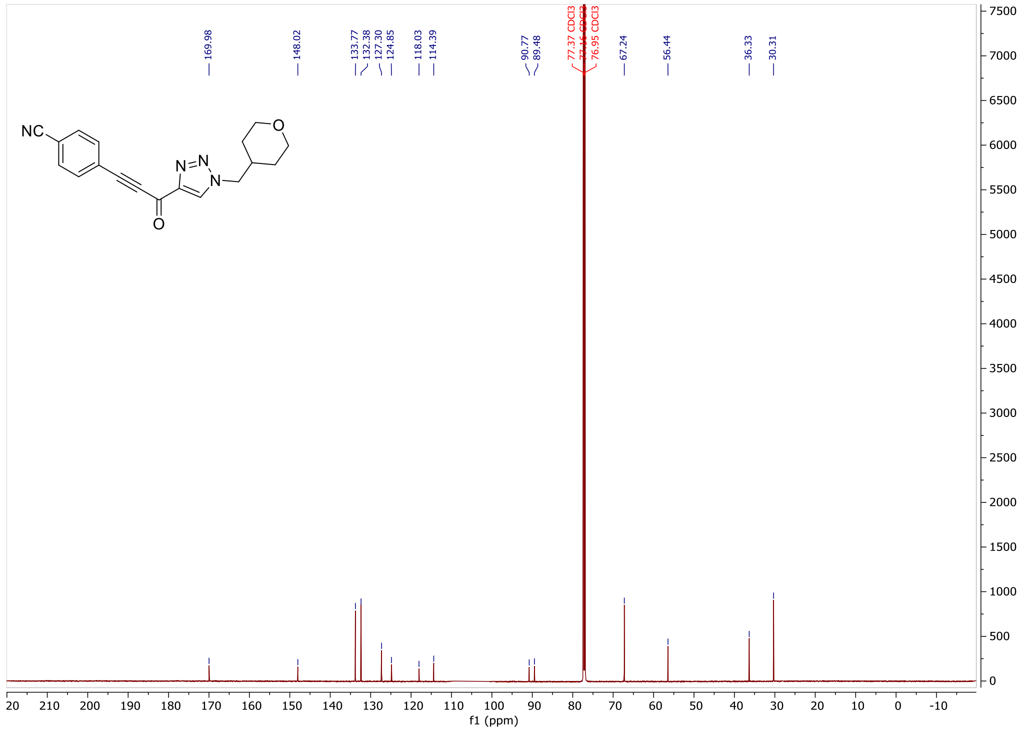

[1] H NMR (300 MHz, $CDCl_3$) Spectrum of Compound **8N**

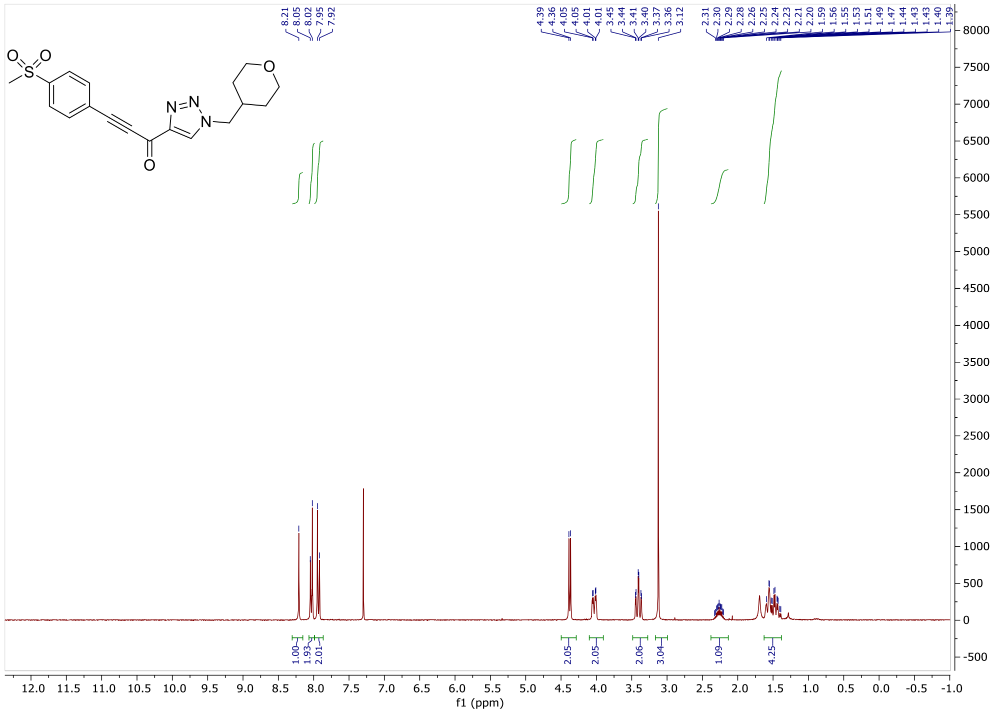

^{13}C NMR (75 MHz, $CDCl_3$) Spectrum of Compound **8N**

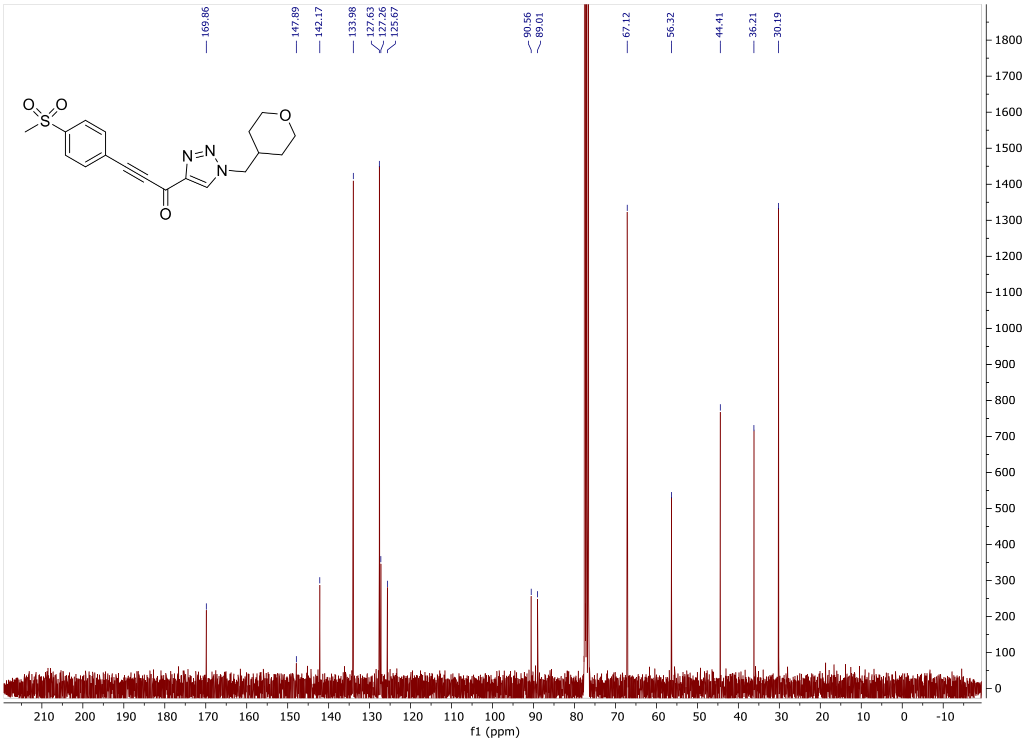

^{1}H NMR (600 MHz, $CDCl_3$) Spectrum of Compound **8O**

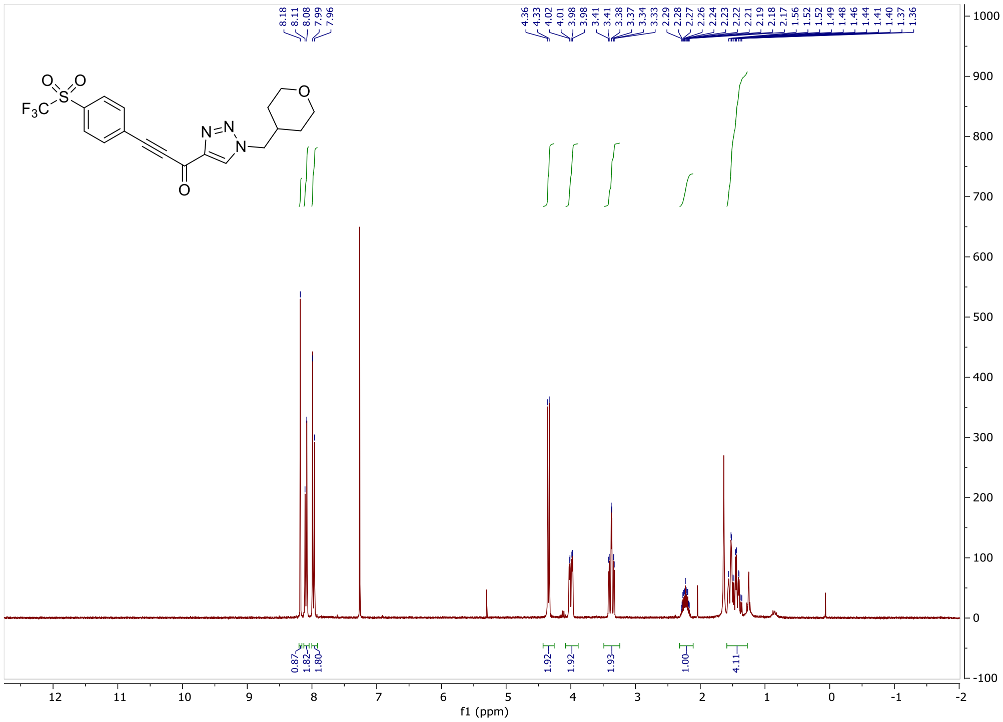

^{13}C NMR (150 MHz, $CDCl_3$) Spectrum of Compound **8O**

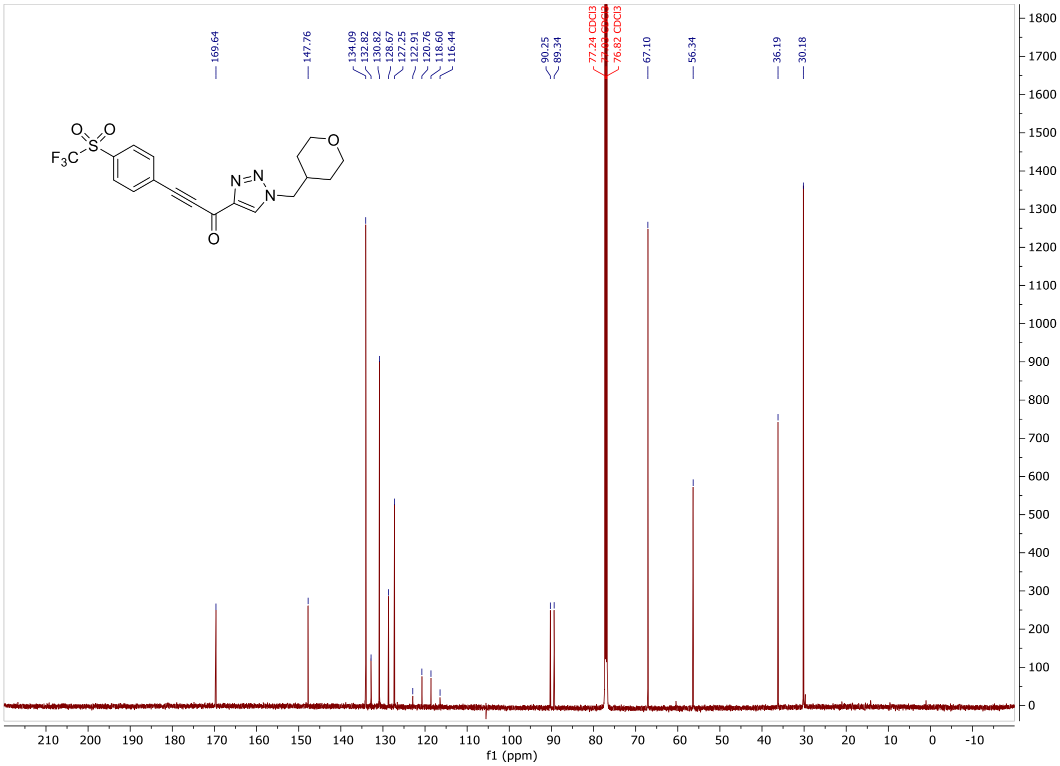

^{19}F NMR (282 MHz, $CDCl_3$) Spectrum of Compound **8O**

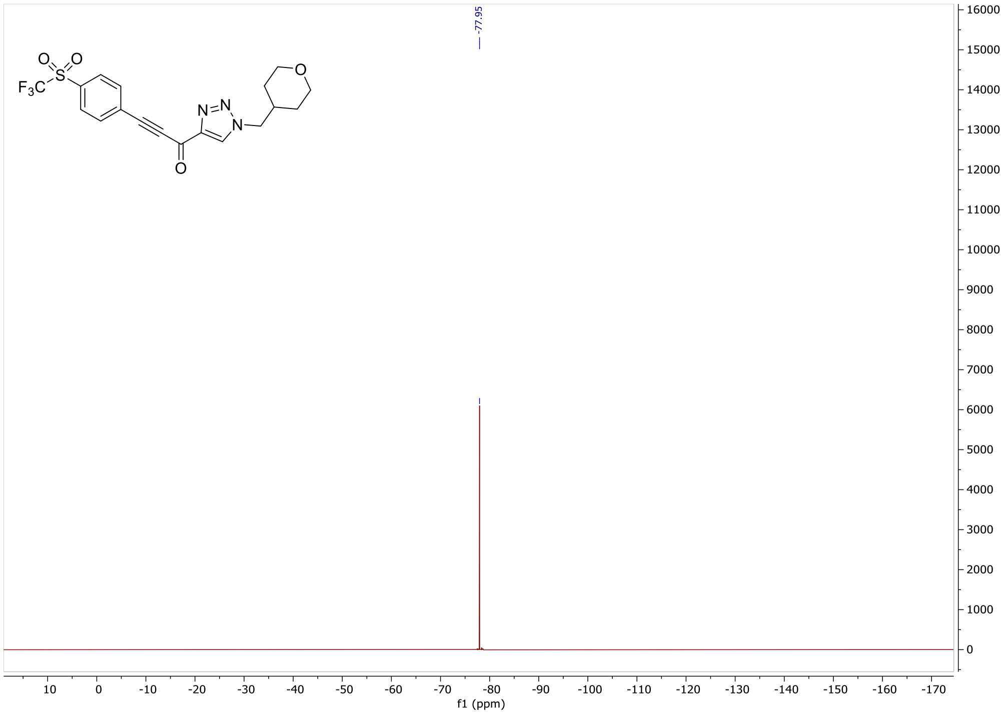

^{1}H NMR (300 MHz, $CDCl_3$) Spectrum of Compound **8P**

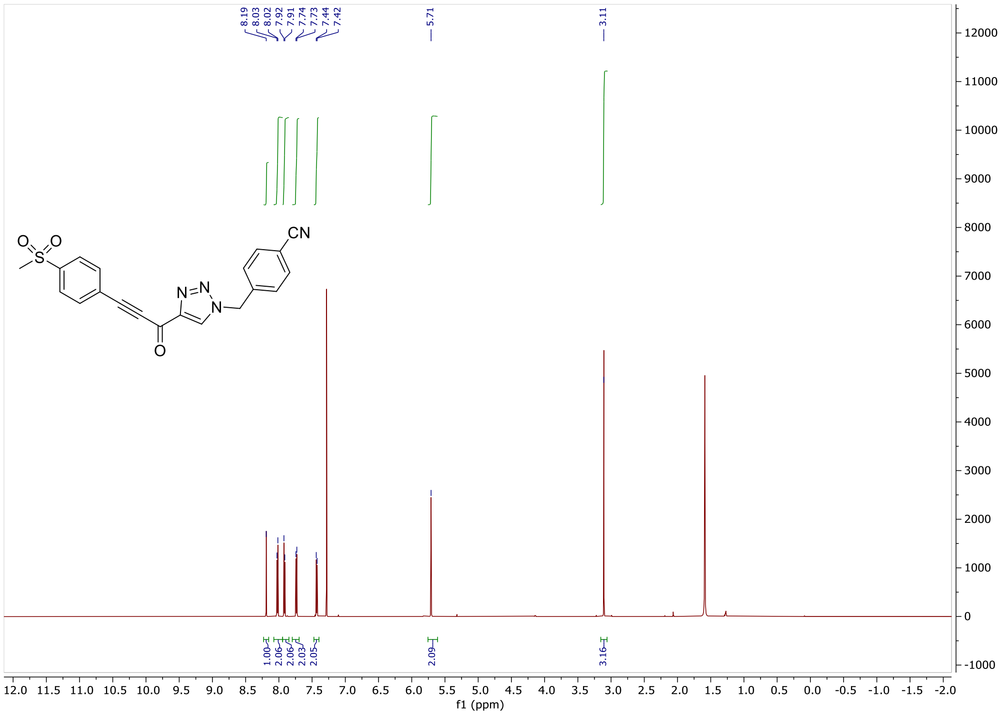

^{13}C NMR (75 MHz, $CDCl_3$) Spectrum of Compound **8P**

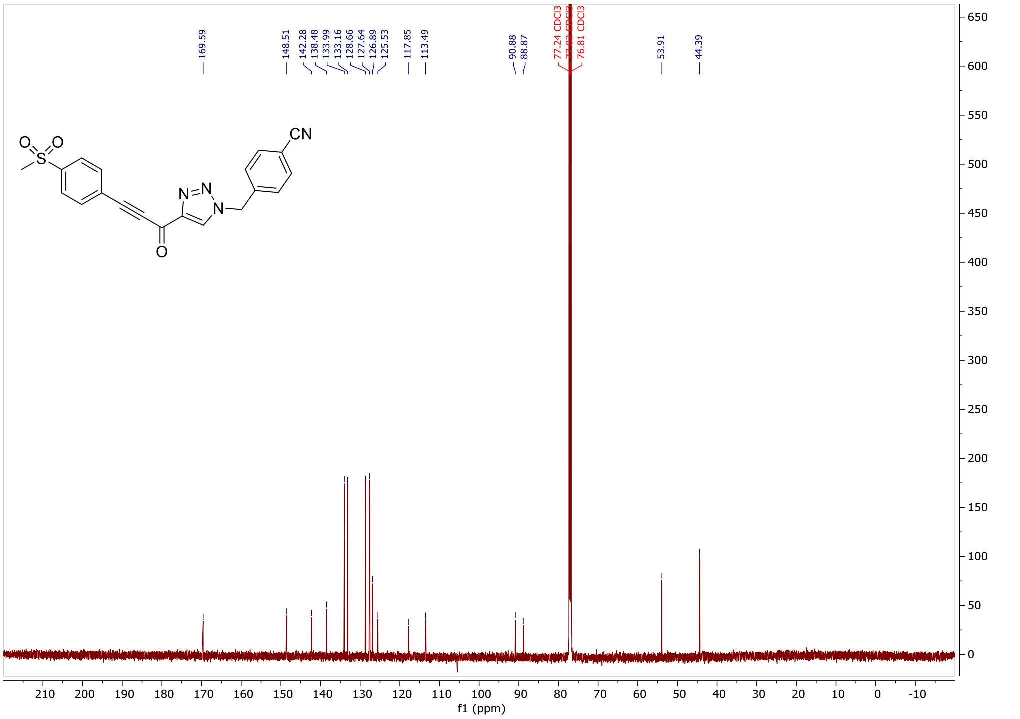

^{1}H NMR (300 MHz, $CDCl_3$) Spectrum of Compound **8Q**

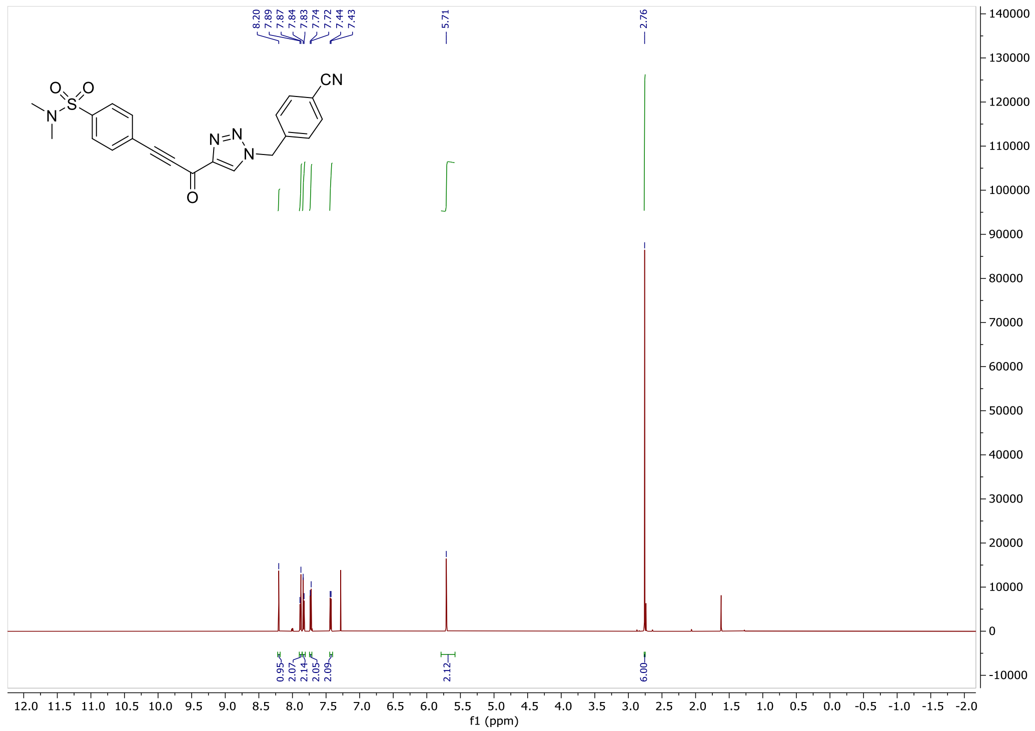

^{13}C NMR (75 MHz, $CDCl_3$) Spectrum of Compound **8Q**

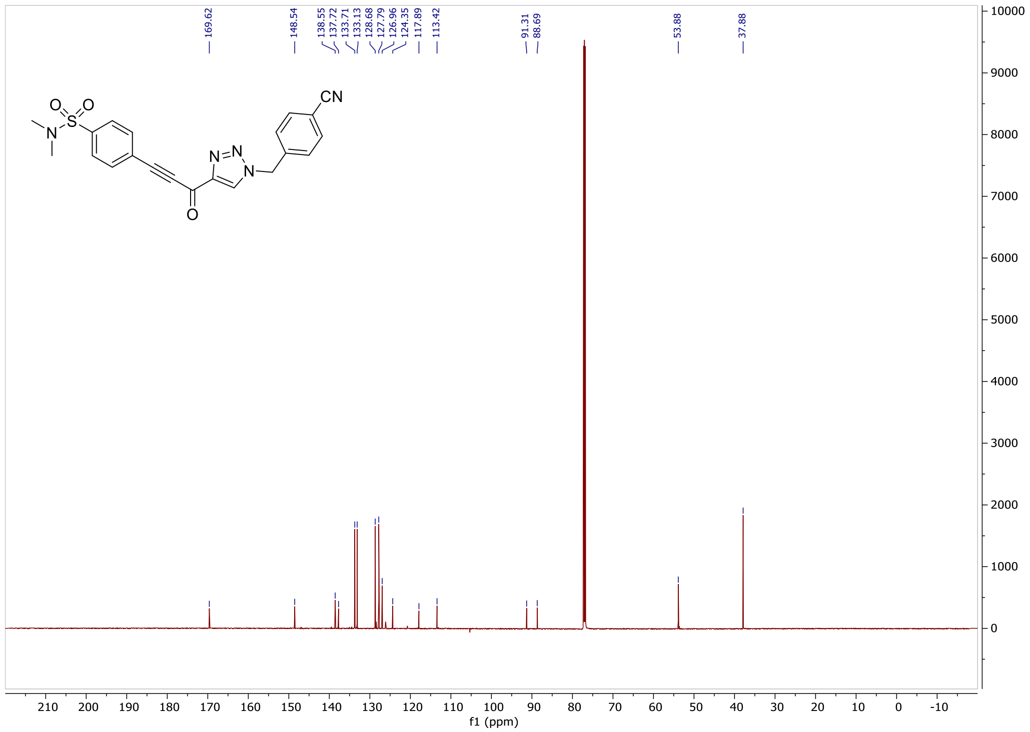

^{1}H NMR (600 MHz, DMSO-d_6) Spectrum of Compound **8R**

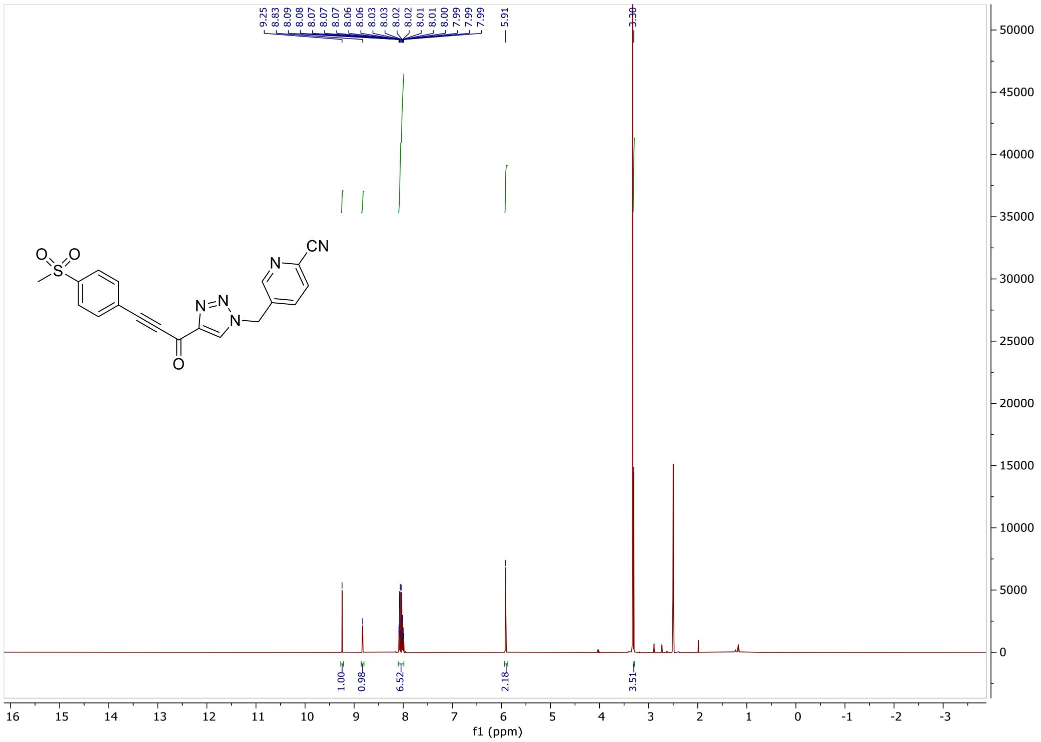

^{13}C NMR (150 MHz, DMSO-d_6) Spectrum of Compound **8R**

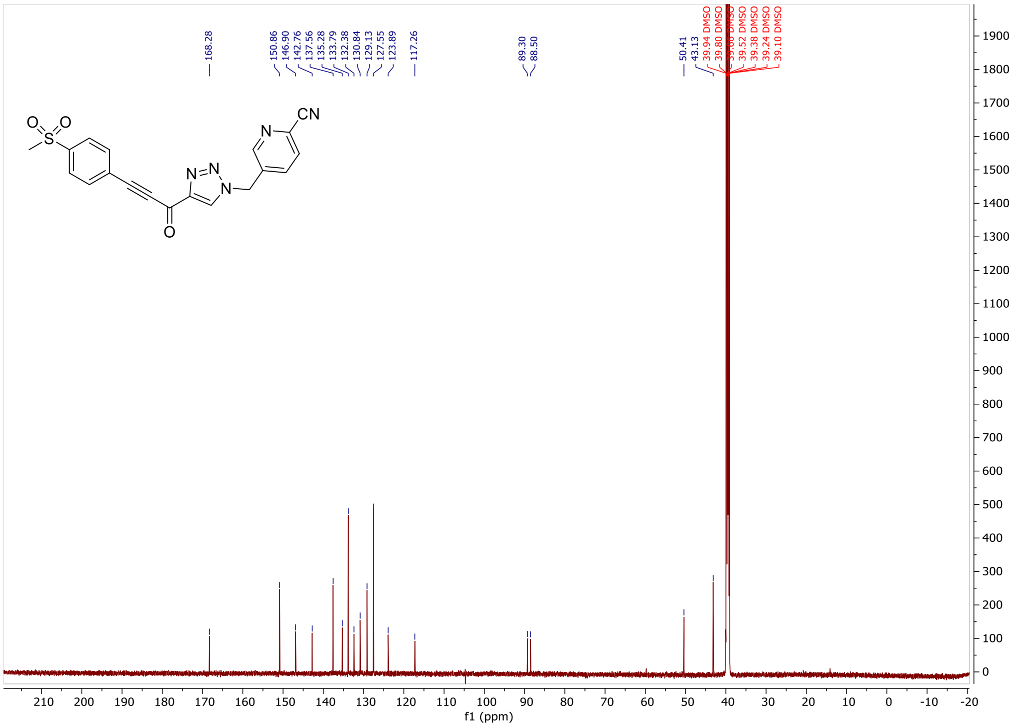

^{1}H NMR (600 MHz, $CDCl_3$) Spectrum of Compound **9**

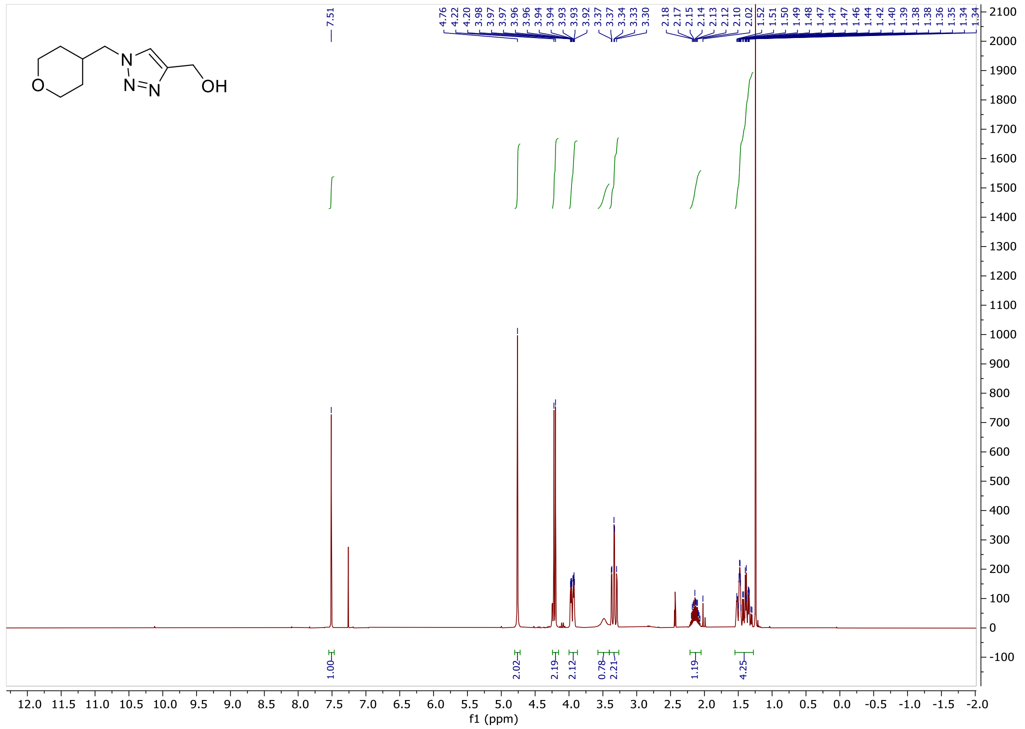

^{13}C NMR (150 MHz, $CDCl_3$) Spectrum of Compound **9**

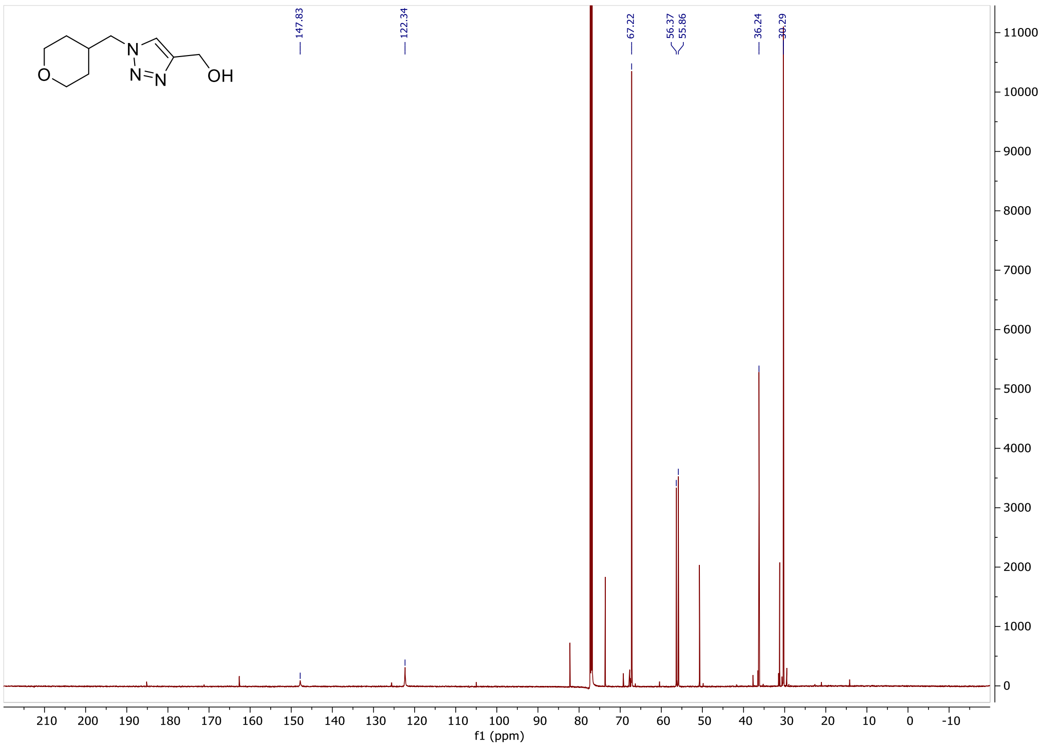

^{1}H NMR (600 MHz, $CDCl_3$) Spectrum of Compound **10**

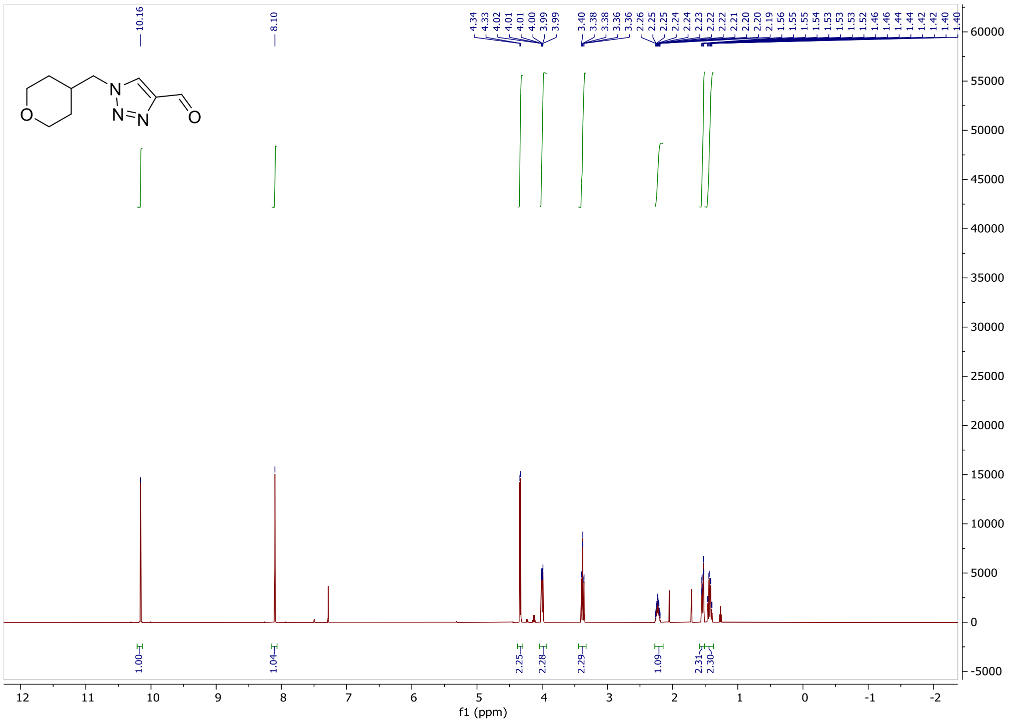

^{13}C NMR (150 MHz, $CDCl_3$) Spectrum of Compound **10**

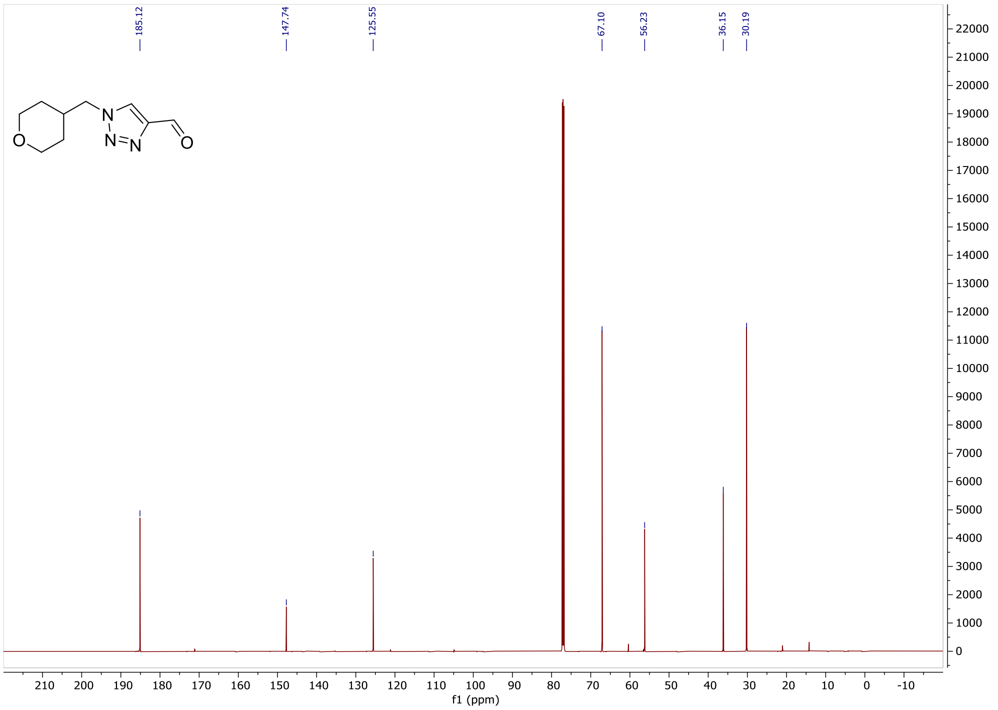

^{1}H NMR (600 MHz, $CDCl_3$) Spectrum of Compound **11**

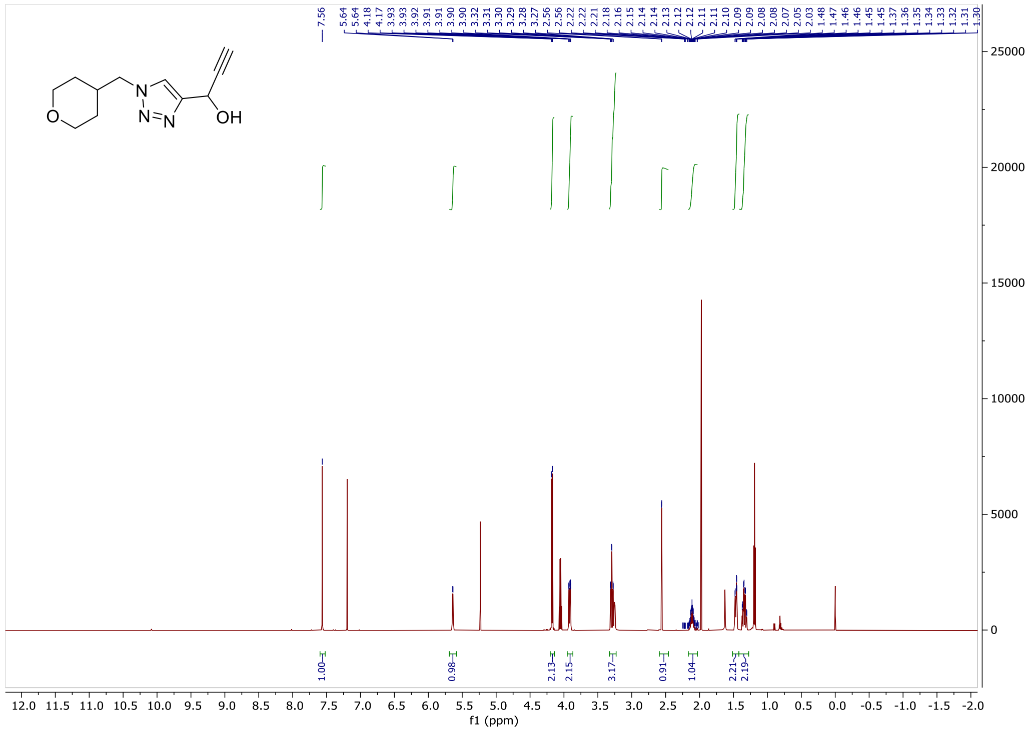

^{13}C NMR (150 MHz, $CDCl_3$) Spectrum of Compound **11**

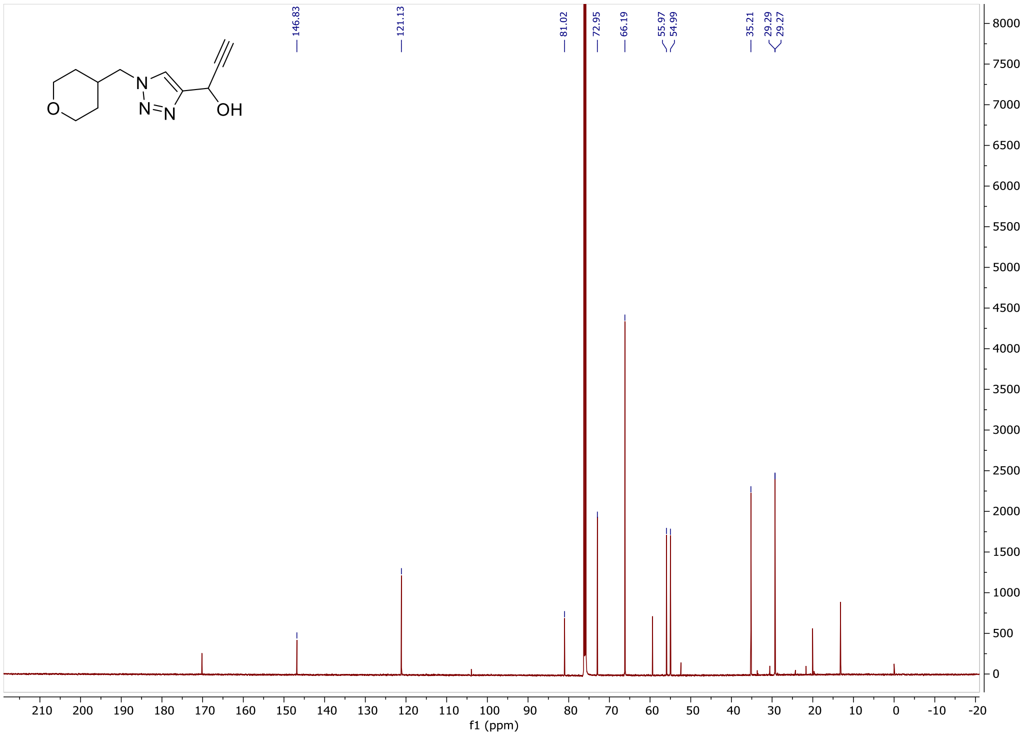

^{1}H NMR (600 MHz, $CDCl_3$) Spectrum of Compound **12**

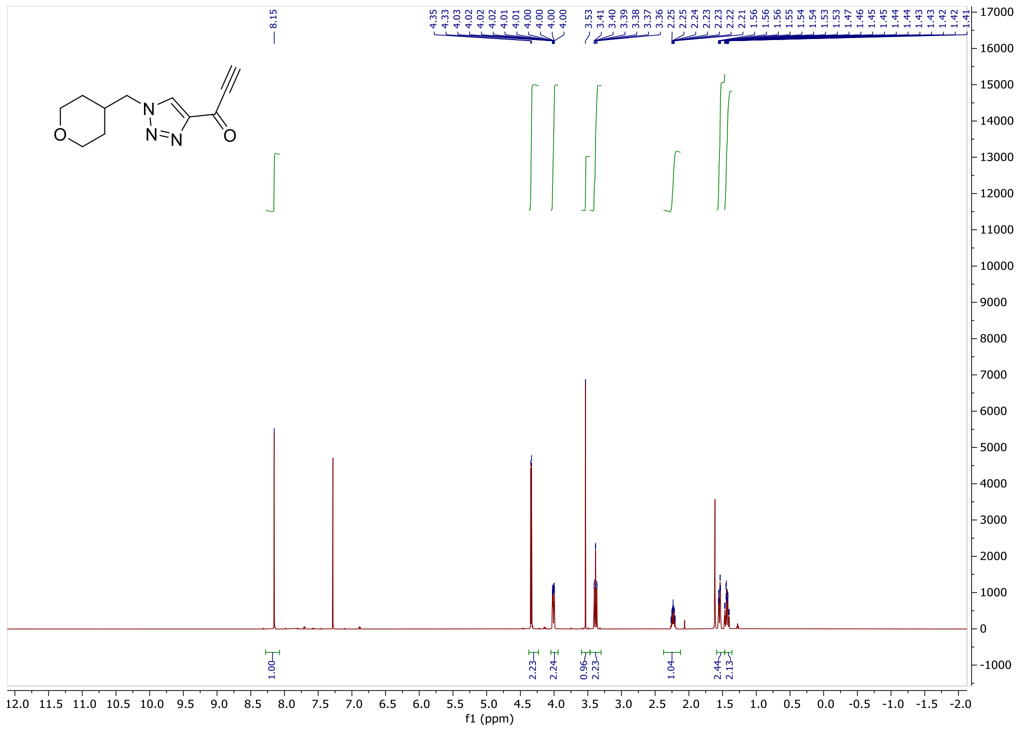

^{13}C NMR (150 MHz, $CDCl_3$) Spectrum of Compound **12**

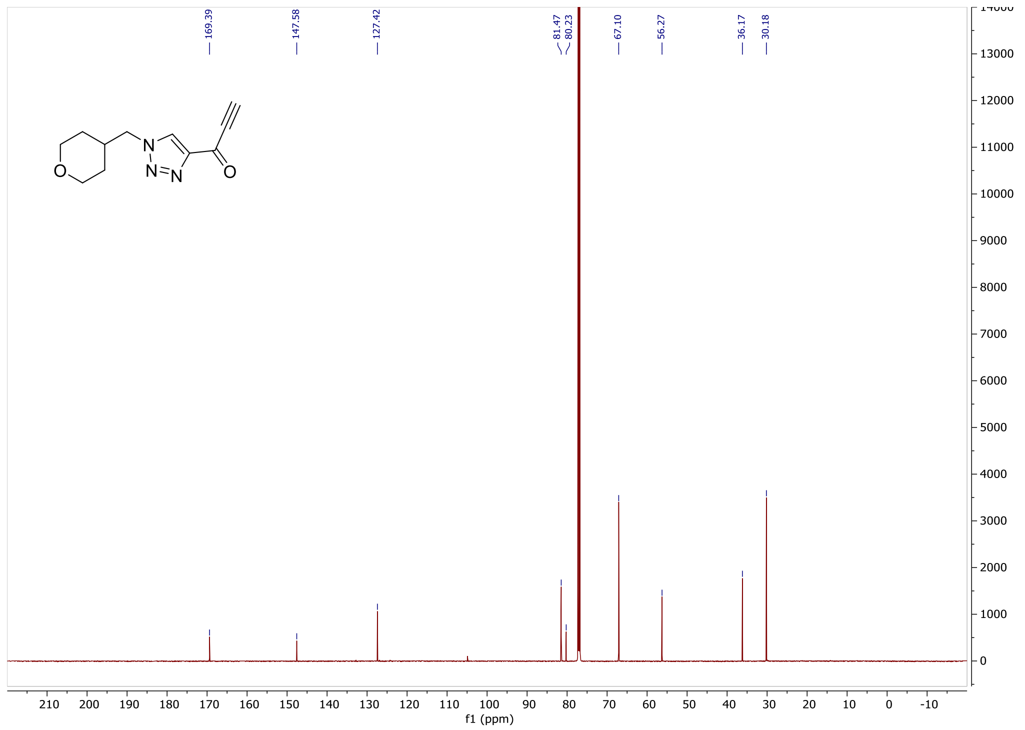

^{1}H NMR (500 MHz, $CDCl_3$) Spectrum of Compound **13**

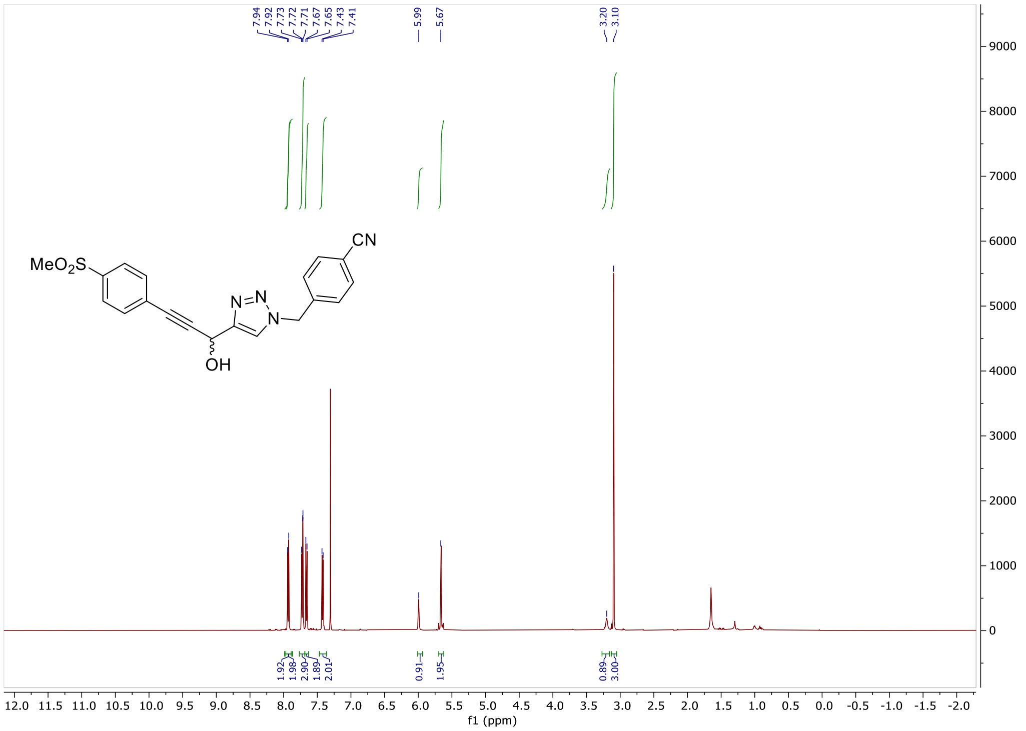

^{13}C NMR (500 MHz, $CDCl_3$) Spectrum of Compound **13**

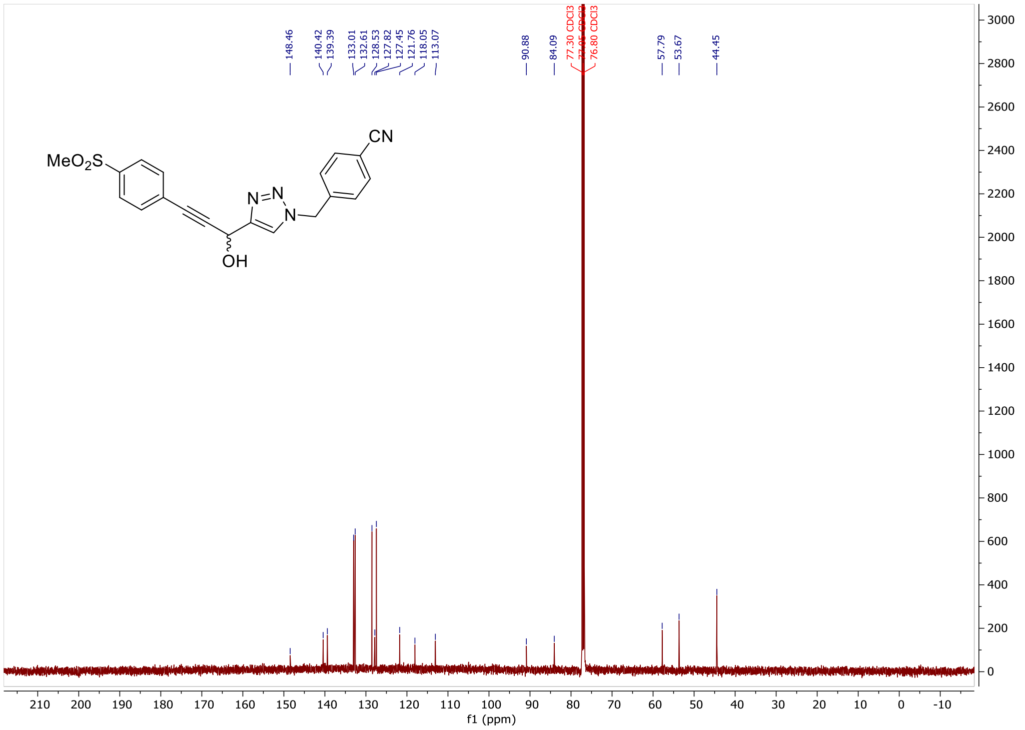

^{1}H NMR (600 MHz, $CDCl_3$) Spectrum of Compound **14**

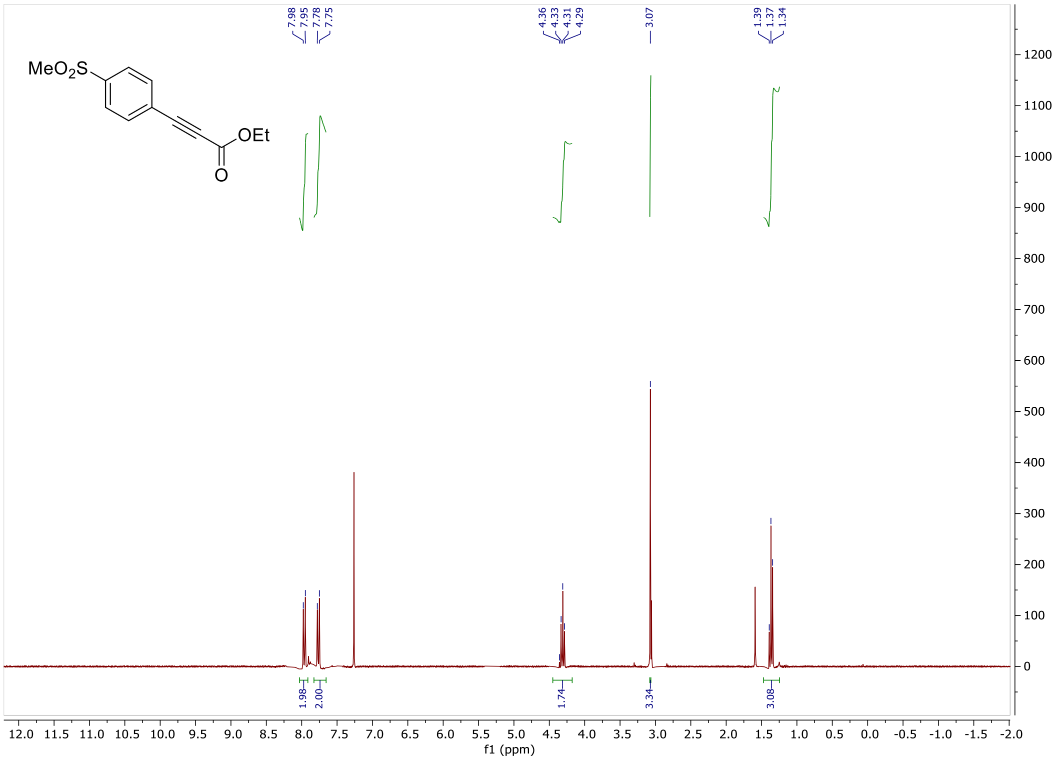

^{13}C NMR (150 MHz, $CDCl_3$) Spectrum of Compound **14**

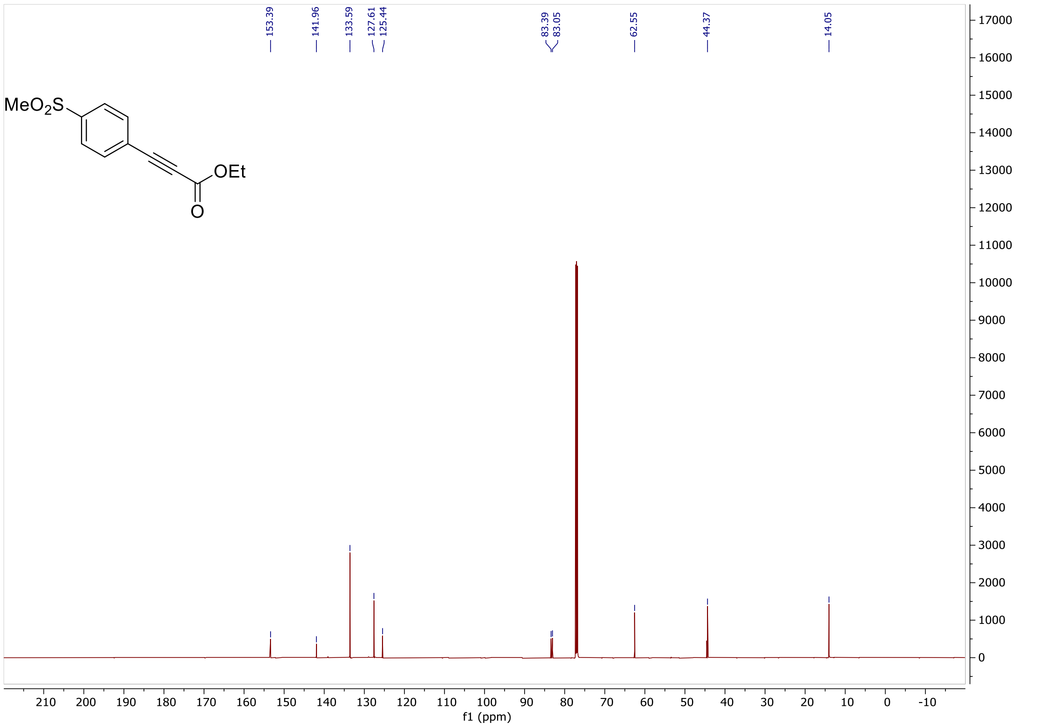

^{1}H NMR (500 MHz, DMSO-d_6) Spectrum of Compound **15**

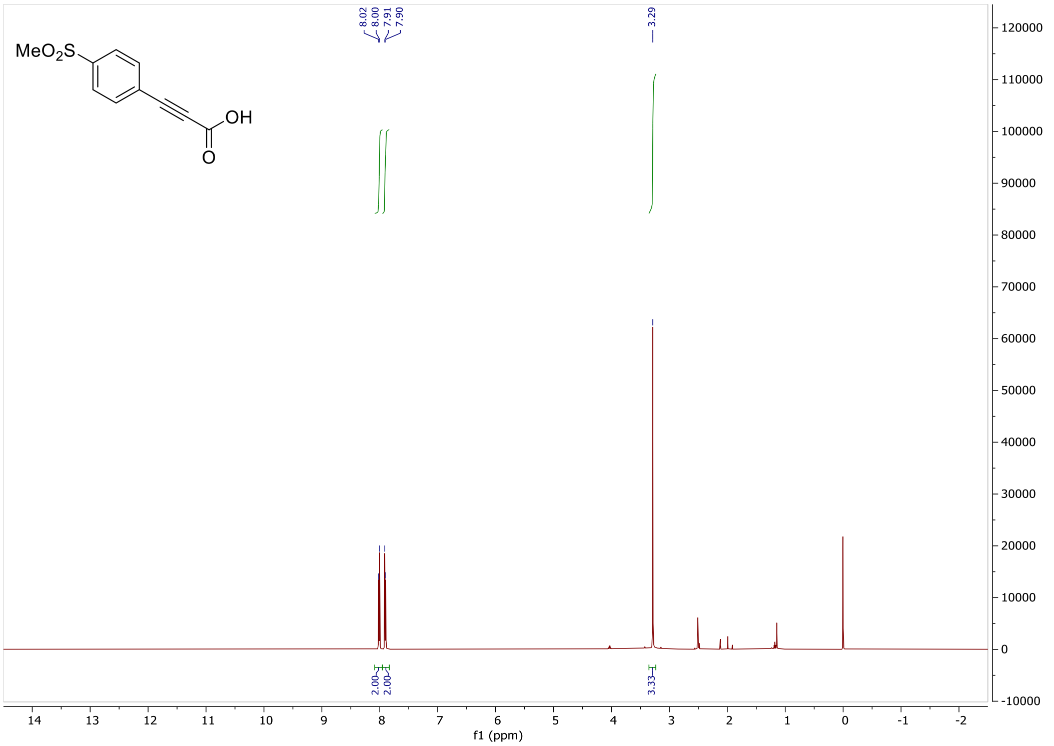

^{13}C NMR (125 MHz, DMSO-d_6) Spectrum of Compound **15**

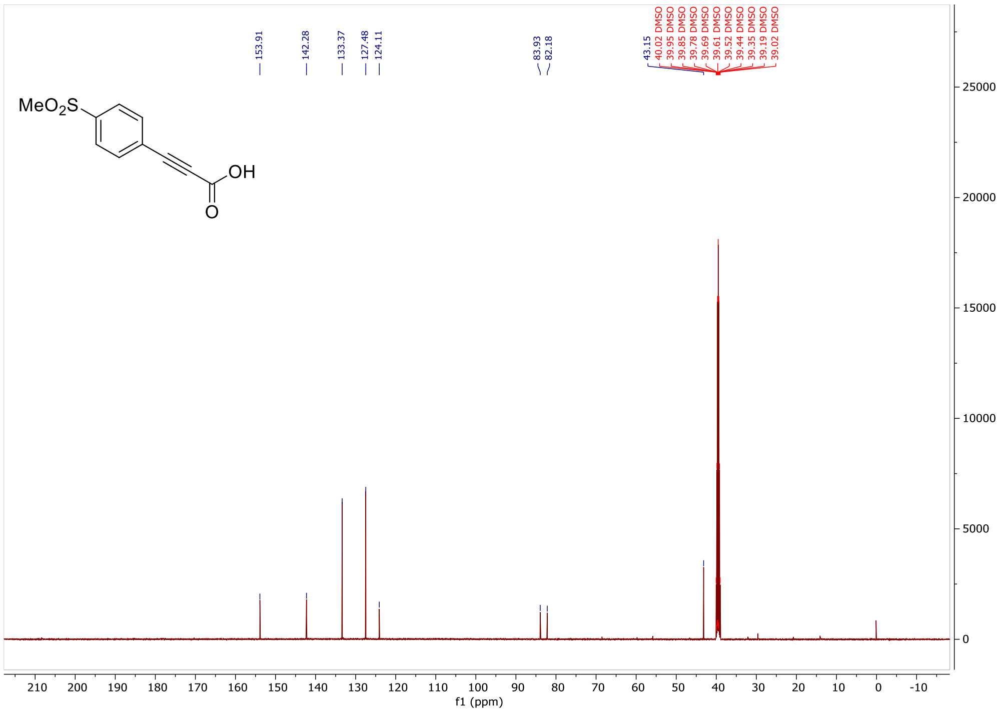

^{1}H NMR (500 MHz, $CDCl_3$) Spectrum of Compound **16**

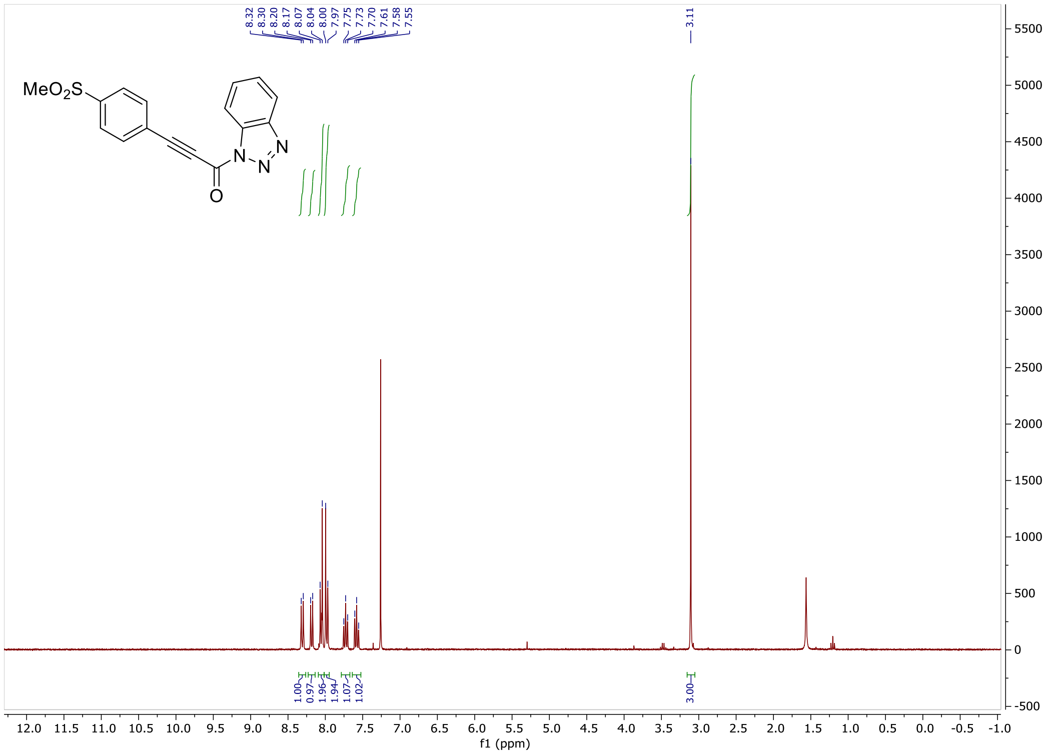

^{13}C NMR (125 MHz, $CDCl_3$) Spectrum of Compound **16**

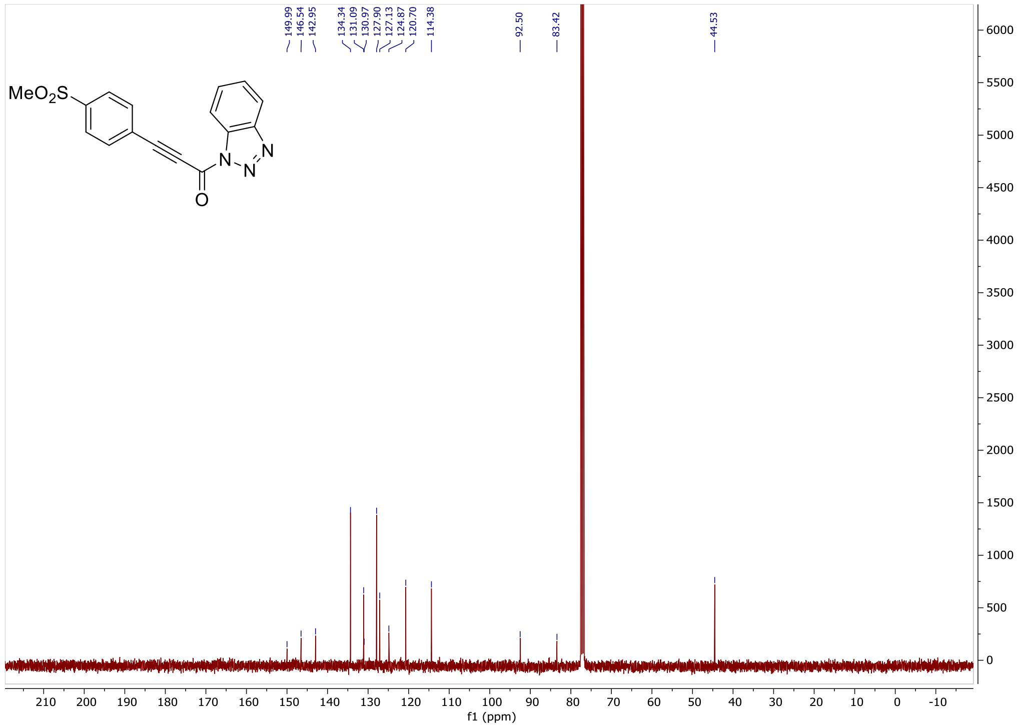

^{1}H NMR (500 MHz, $CDCl_3 + CD_3OD$) Spectrum of Compound **17**

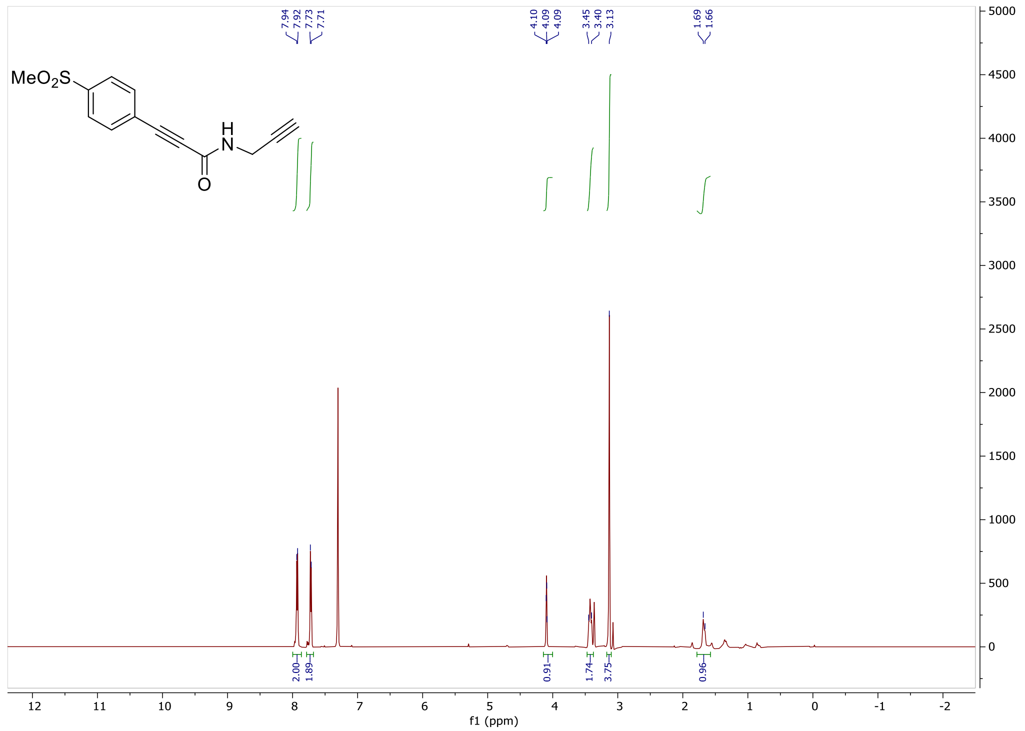

^{13}C NMR (125 MHz, $CDCl_3 + CD_3OD$) Spectrum of Compound **17**

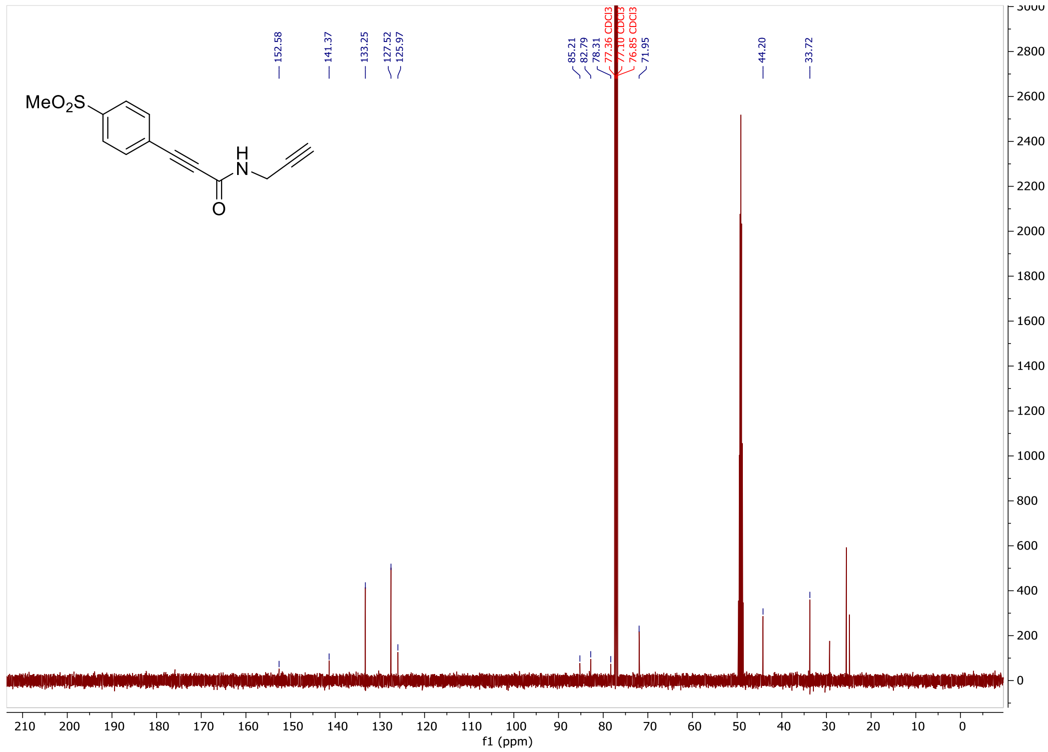

^{1}H NMR (600 MHz, $CDCl_3$) Spectrum of Compound **18**

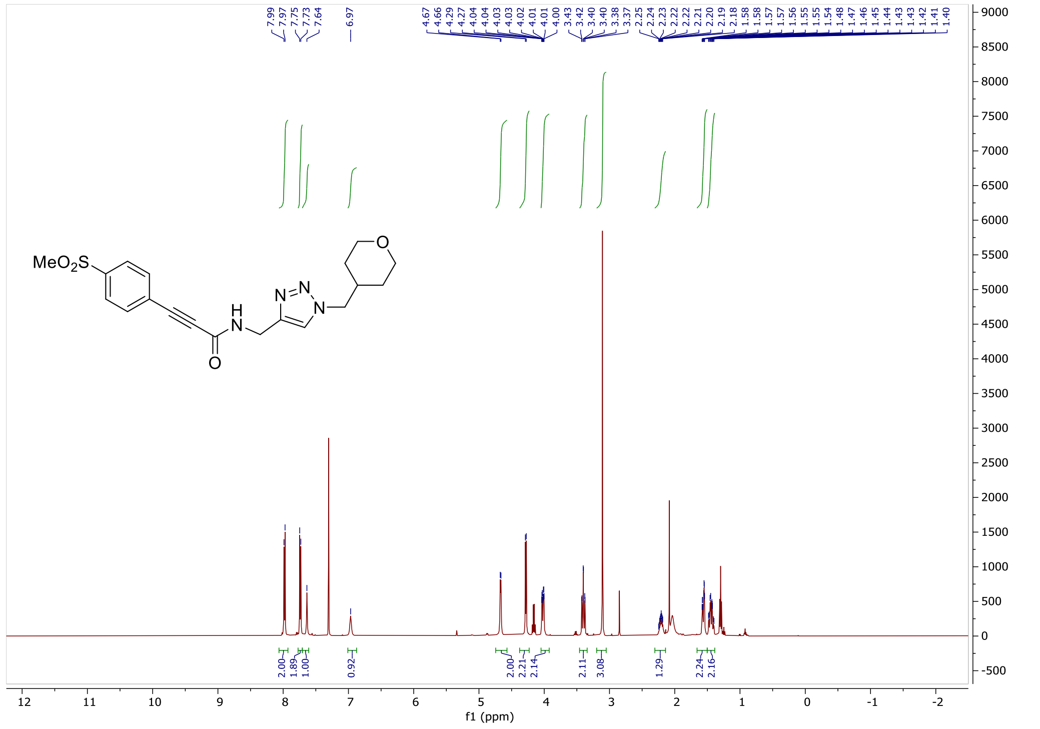

^{13}C NMR (150 MHz, $CDCl_3$) Spectrum of Compound **18**

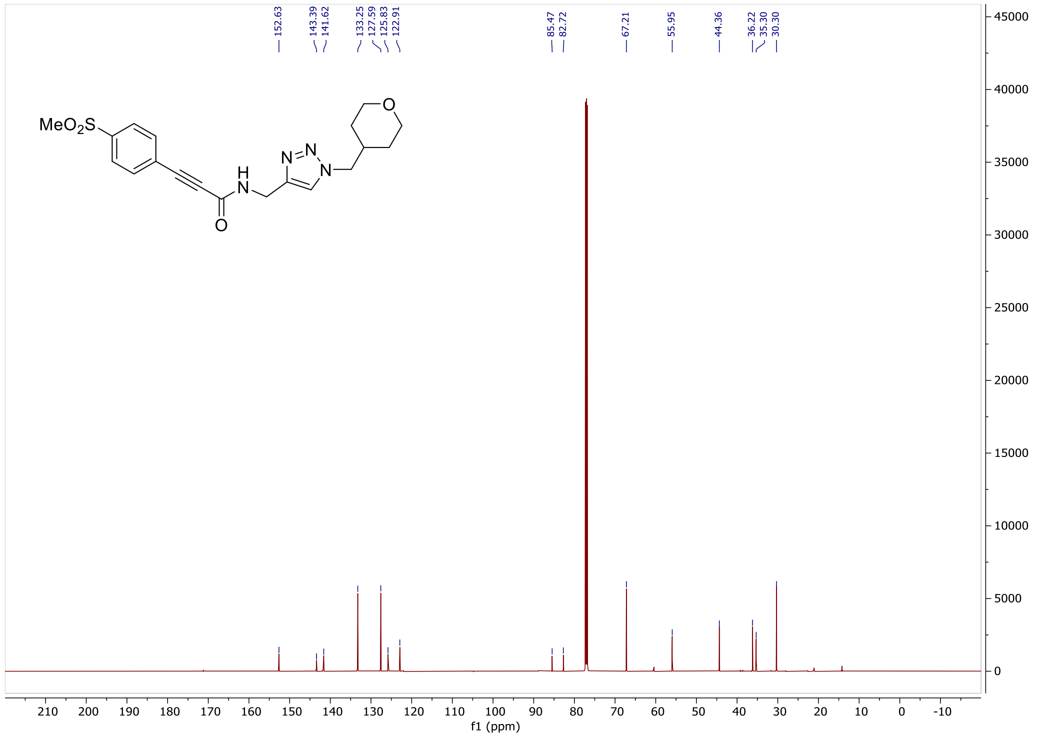

^{1}H NMR (500 MHz, $CDCl_3$) Spectrum of Compound **19A**

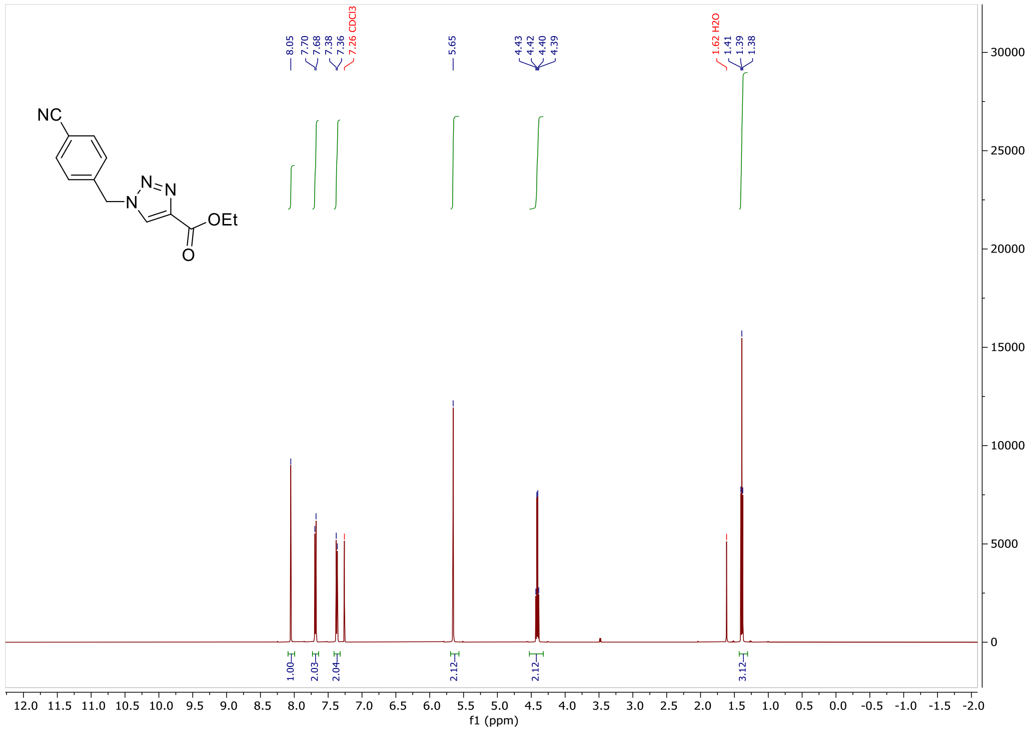

^{13}C NMR (125 MHz, $CDCl_3$) Spectrum of Compound **19A**

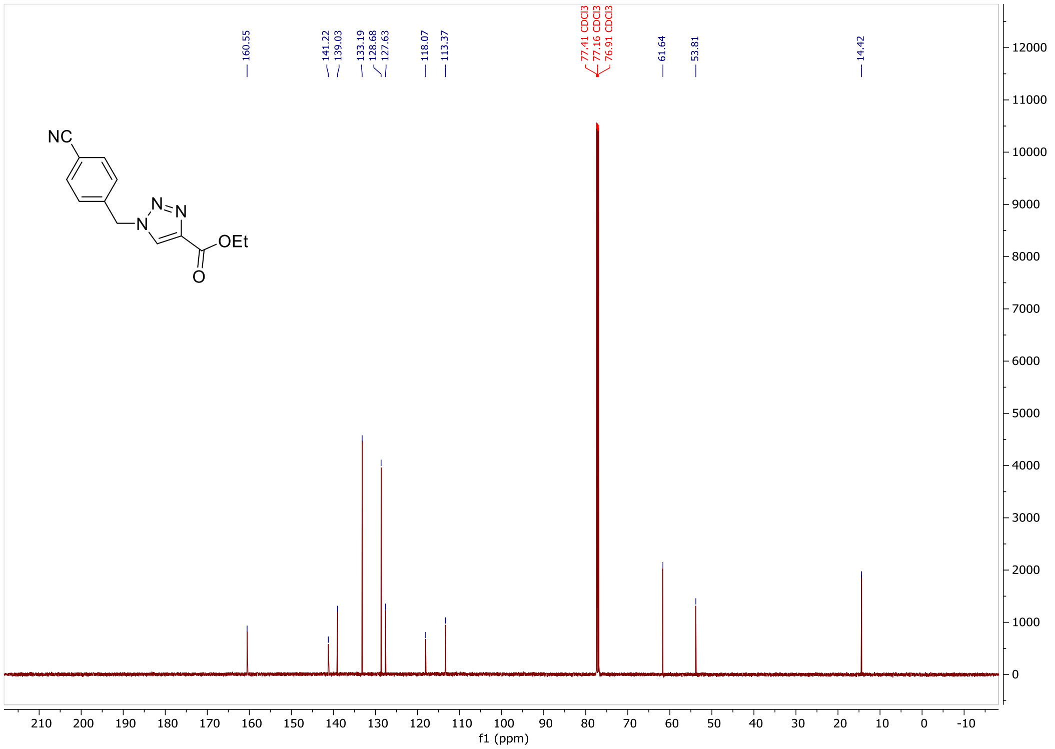

^{1}H NMR (500 MHz, DMSO-d_6) Spectrum of Compound **20A**

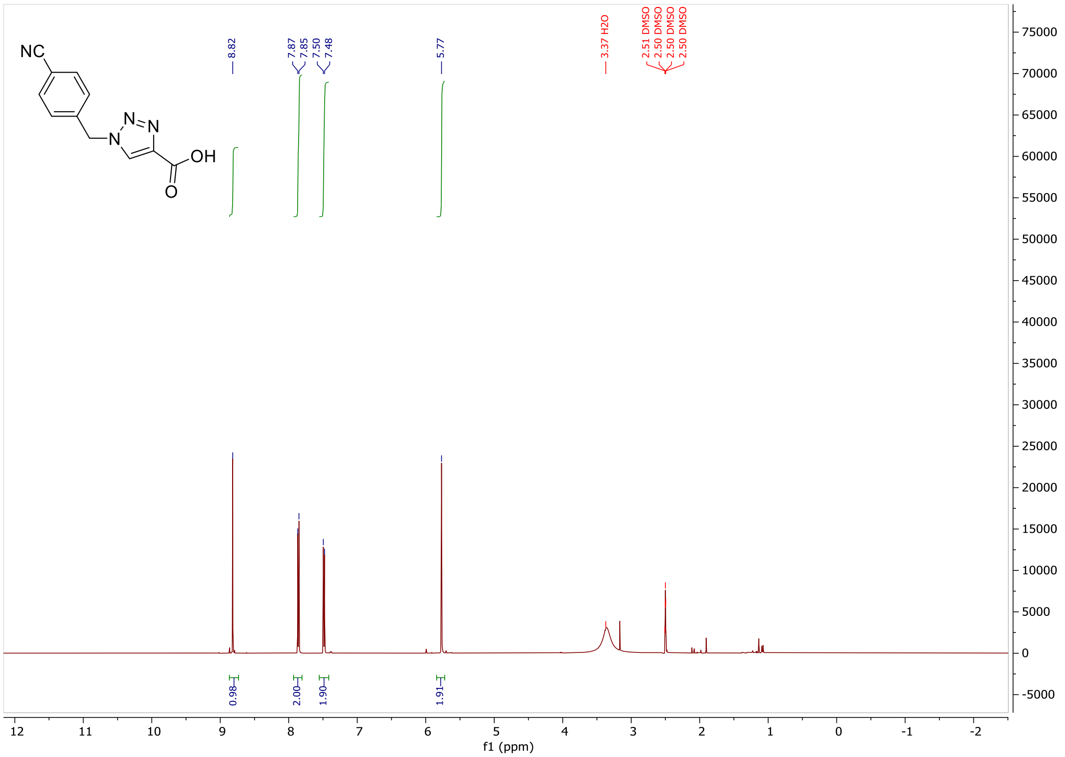

^{13}C NMR (125 MHz, DMSO-d_6) Spectrum of Compound **20A**

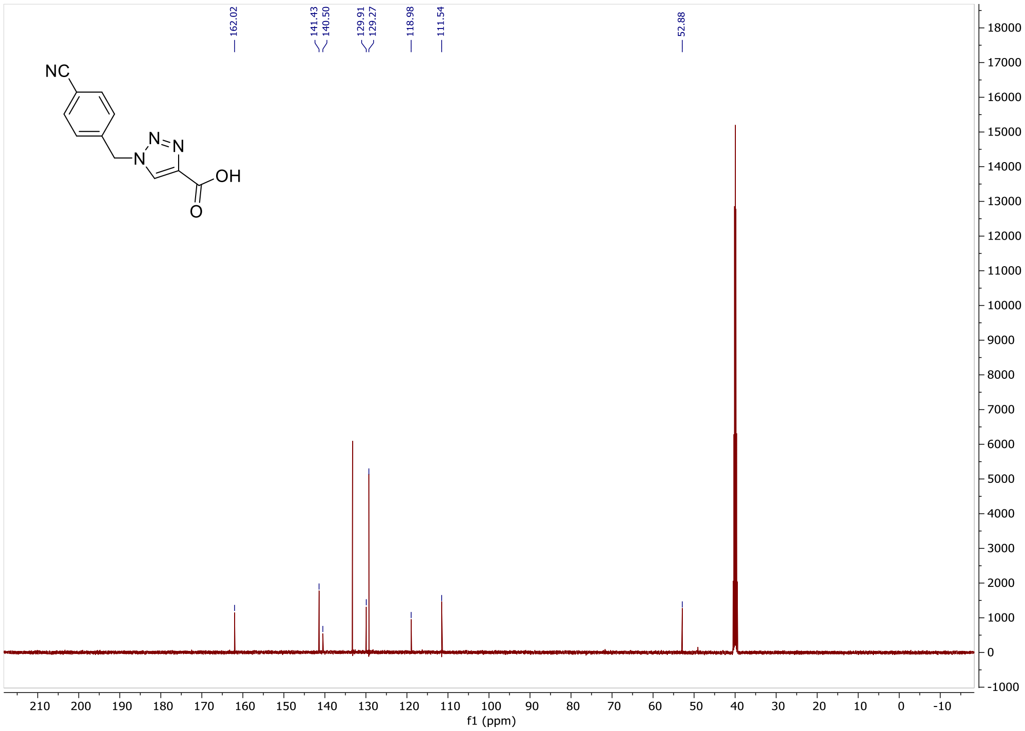

^{1}H NMR (500 MHz, $CDCl_3$) Spectrum of Compound **21A**

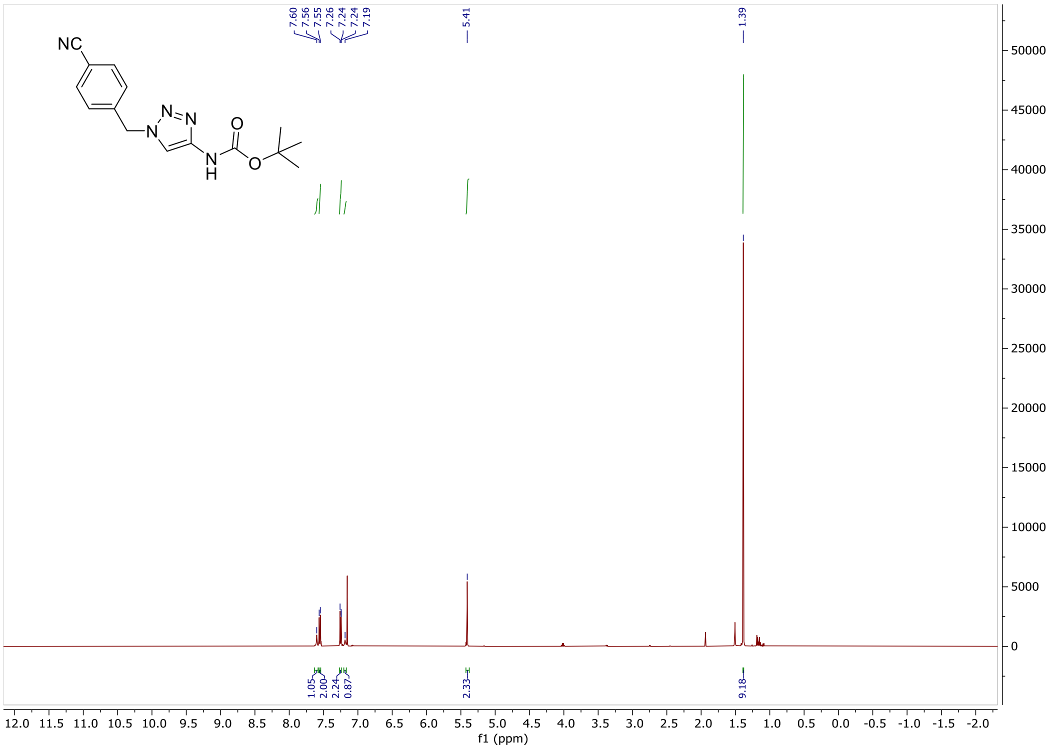

^{13}C NMR (125 MHz, $CDCl_3$) Spectrum of Compound **21A**

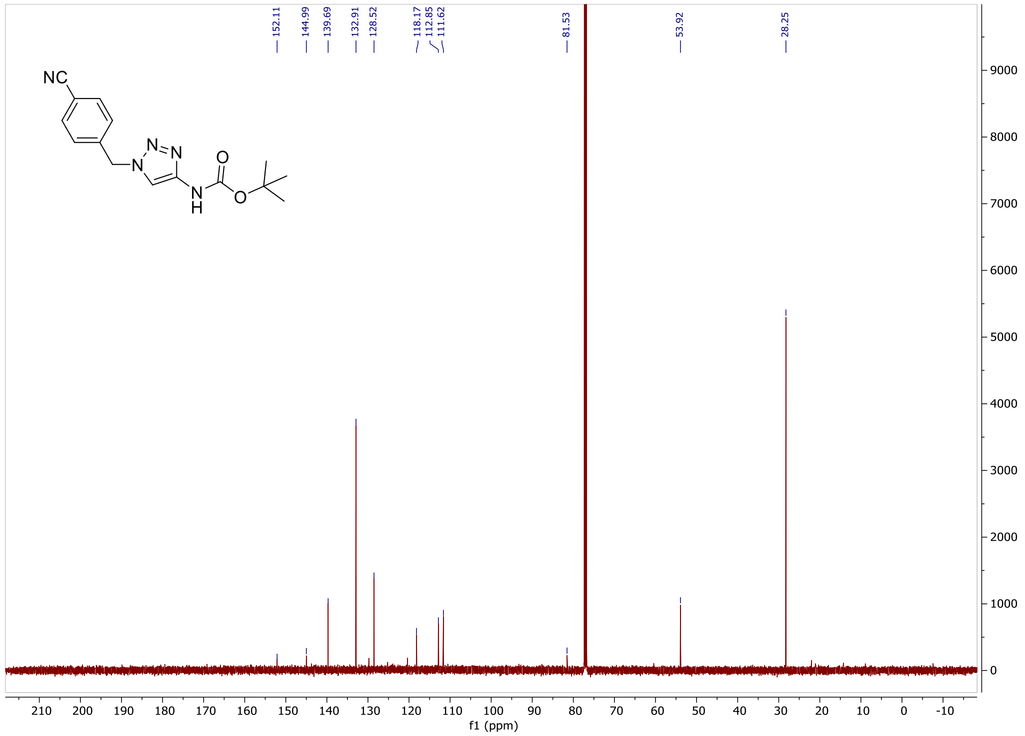

^{1}H NMR (500 MHz, DMSO-d_6) Spectrum of Compound **22A**

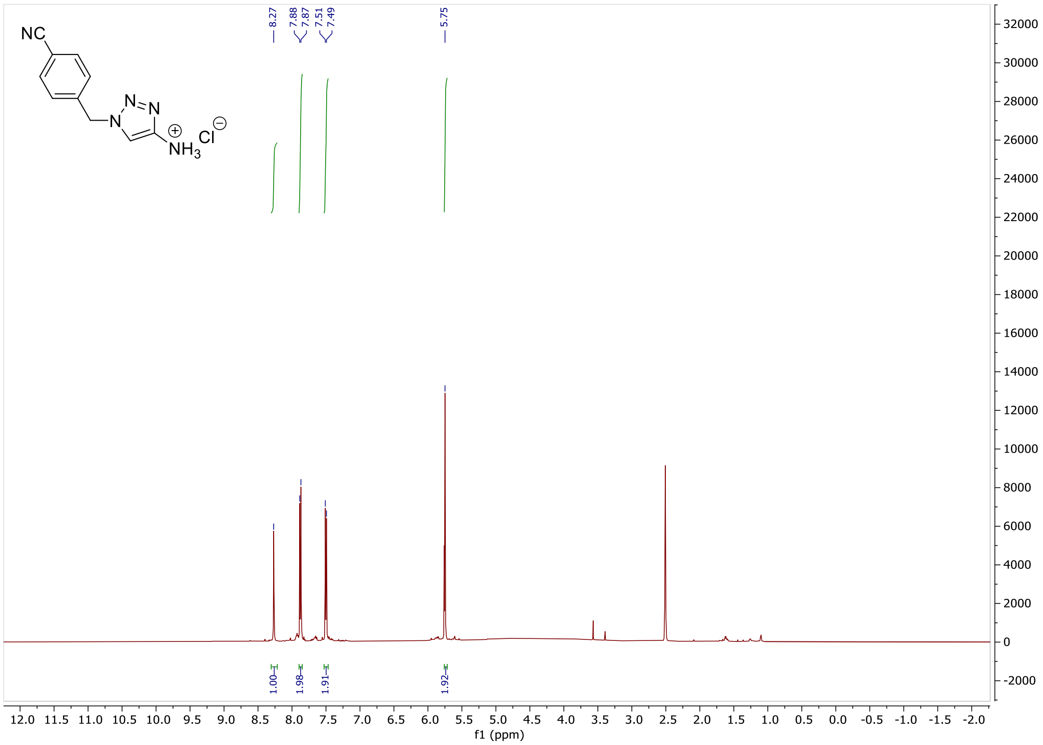

^{13}C NMR (125 MHz, DMSO-d_6) Spectrum of Compound **22A**

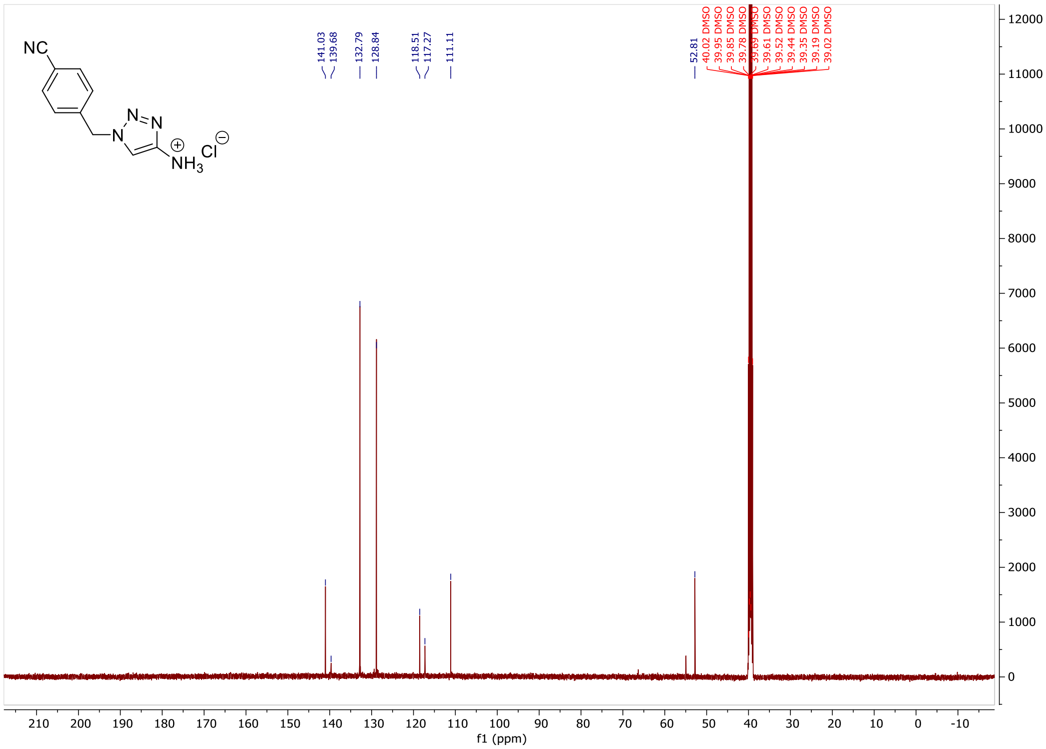

^{1}H NMR (500 MHz, DMSO-d_6) Spectrum of Compound **23A**

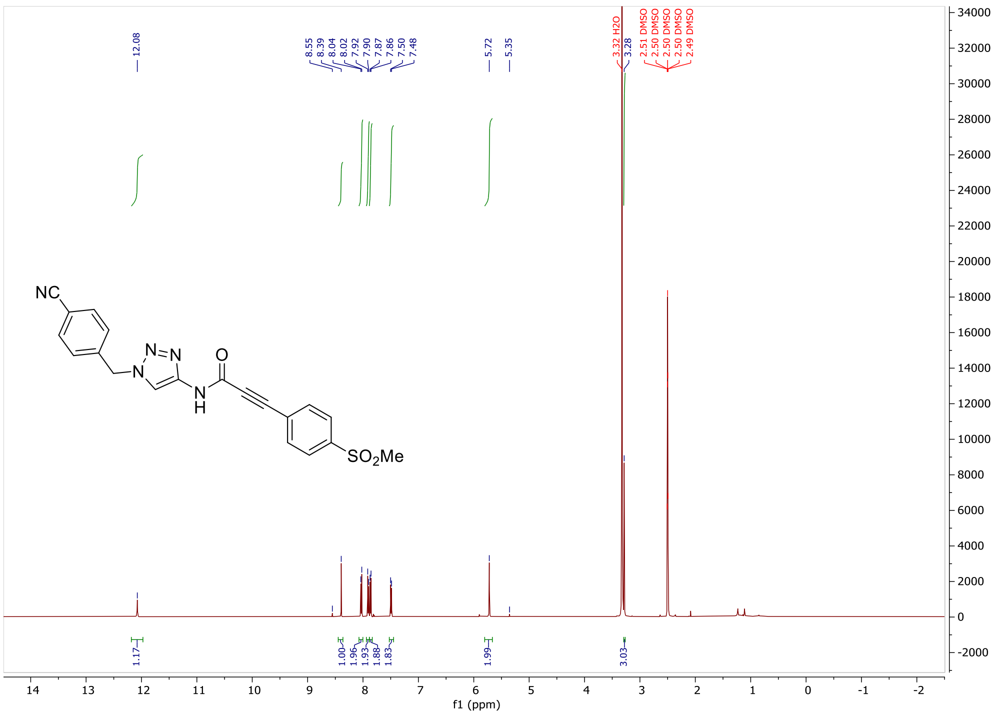

^{13}C NMR (150 MHz, DMSO-d_6) Spectrum of Compound **23A**

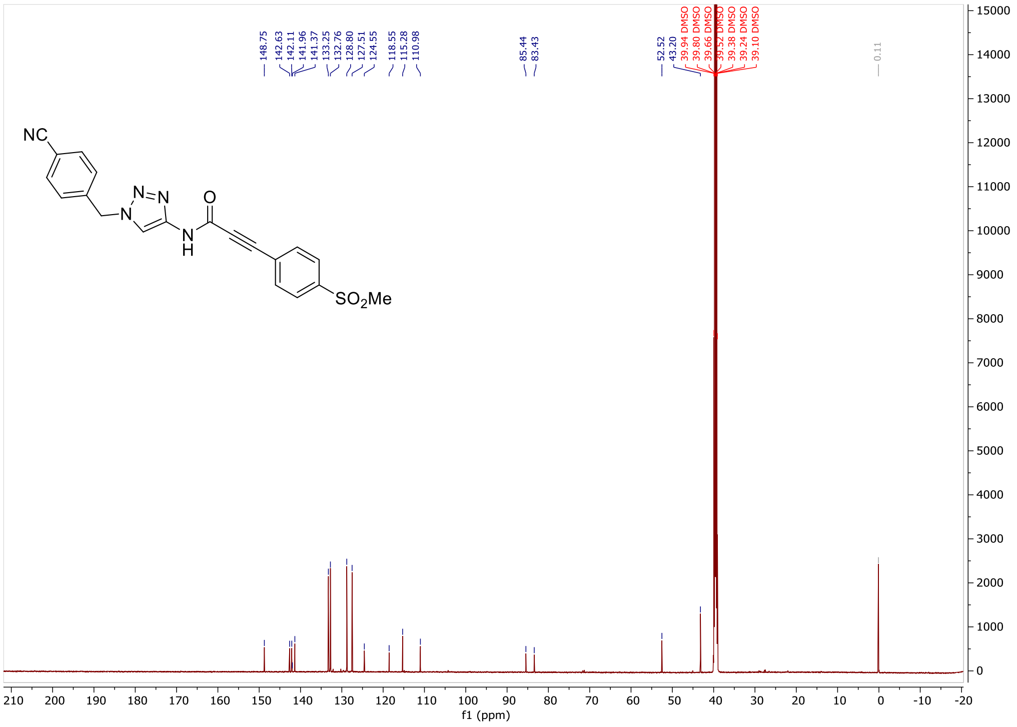

^{1}H NMR (500 MHz, $CDCl_3$) Spectrum of Compound **19B**

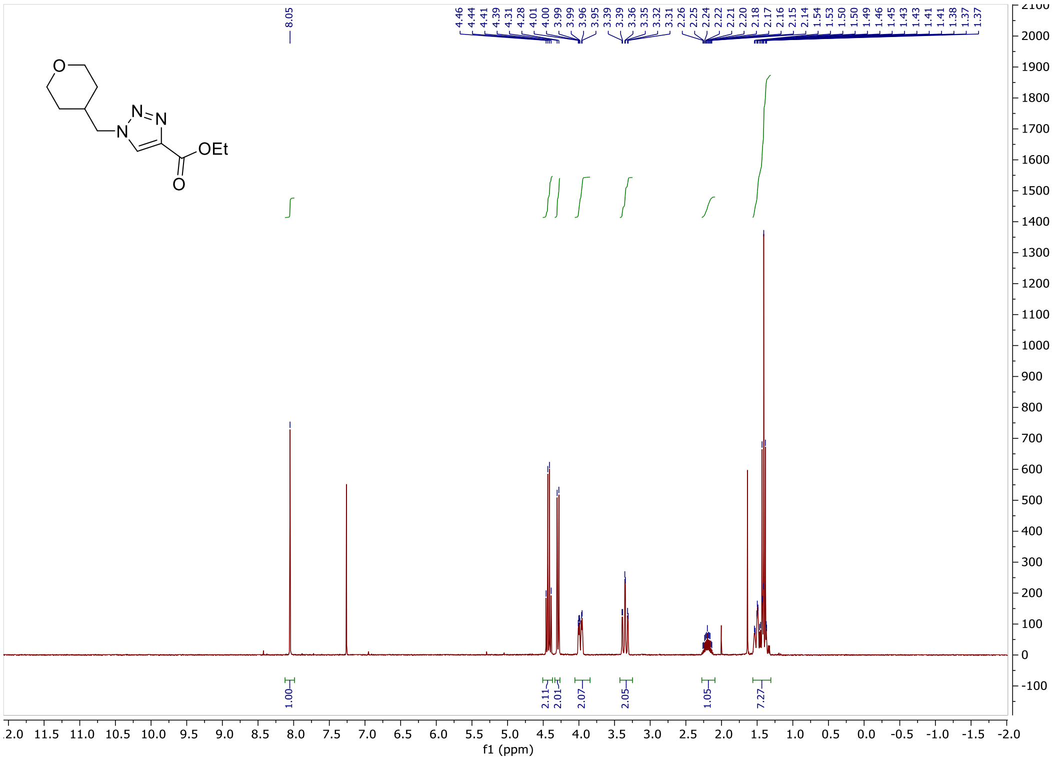

^{13}C NMR (125 MHz, $CDCl_3$) Spectrum of Compound **19B**

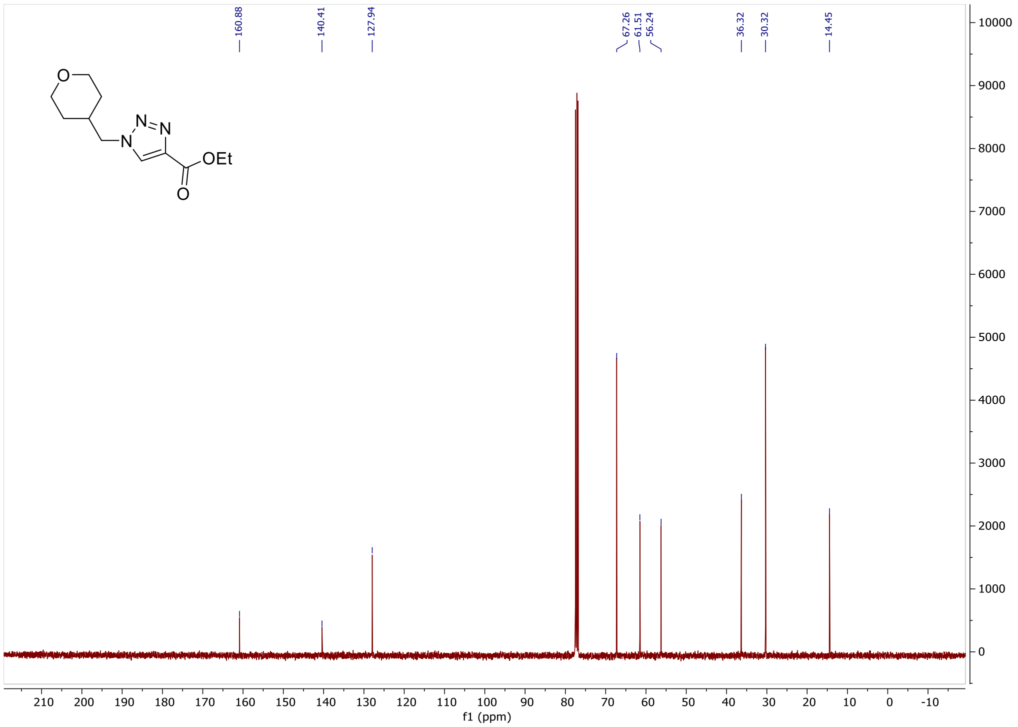

^{1}H NMR (500 MHz, DMSO-d_6) Spectrum of Compound **20B**

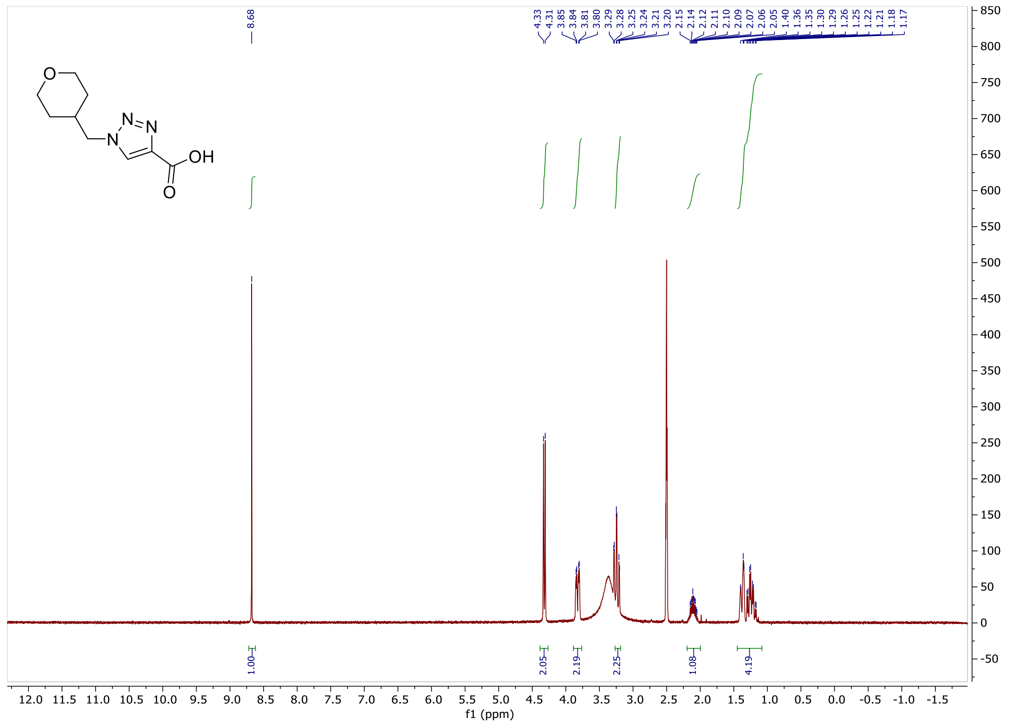

^{13}C NMR (125 MHz, DMSO-d_6) Spectrum of Compound **20B**

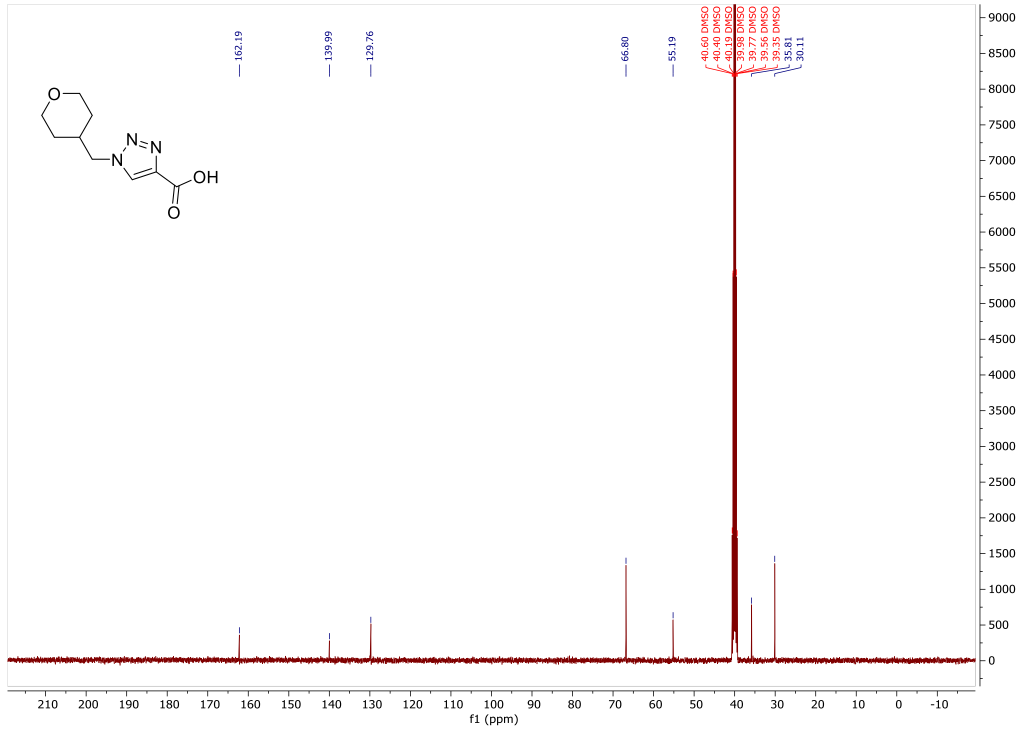

^{1}H NMR (600 MHz, $CDCl_3$) Spectrum of Compound **21B**

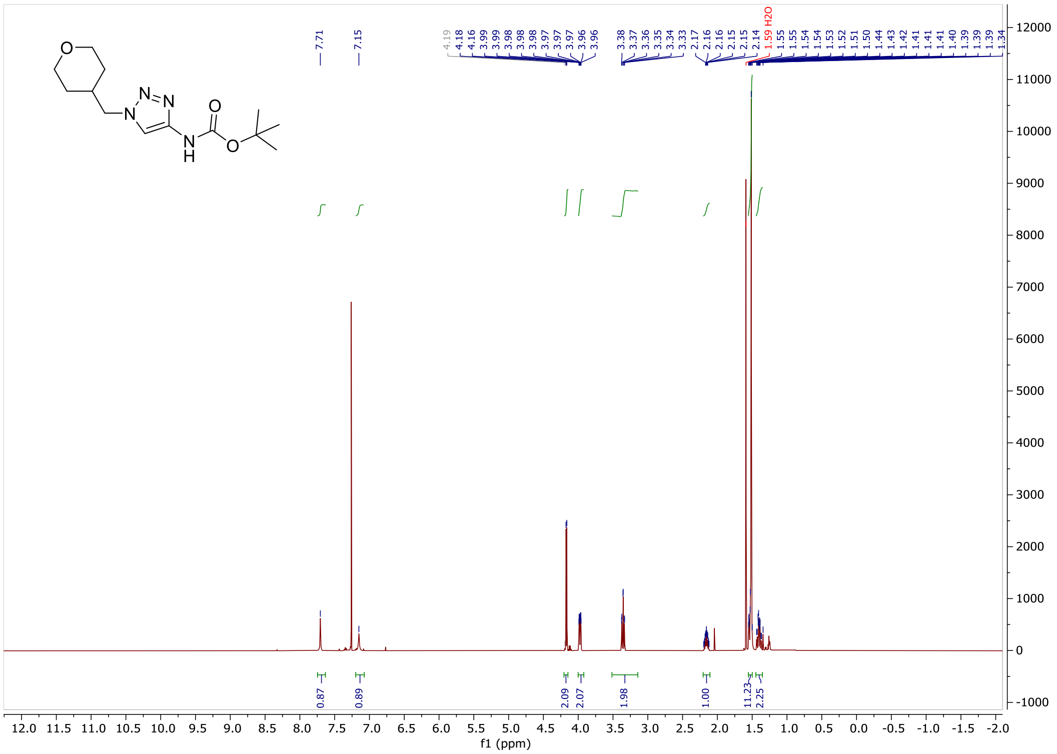

^{13}C NMR (150 MHz, $CDCl_3$) Spectrum of Compound **21B**

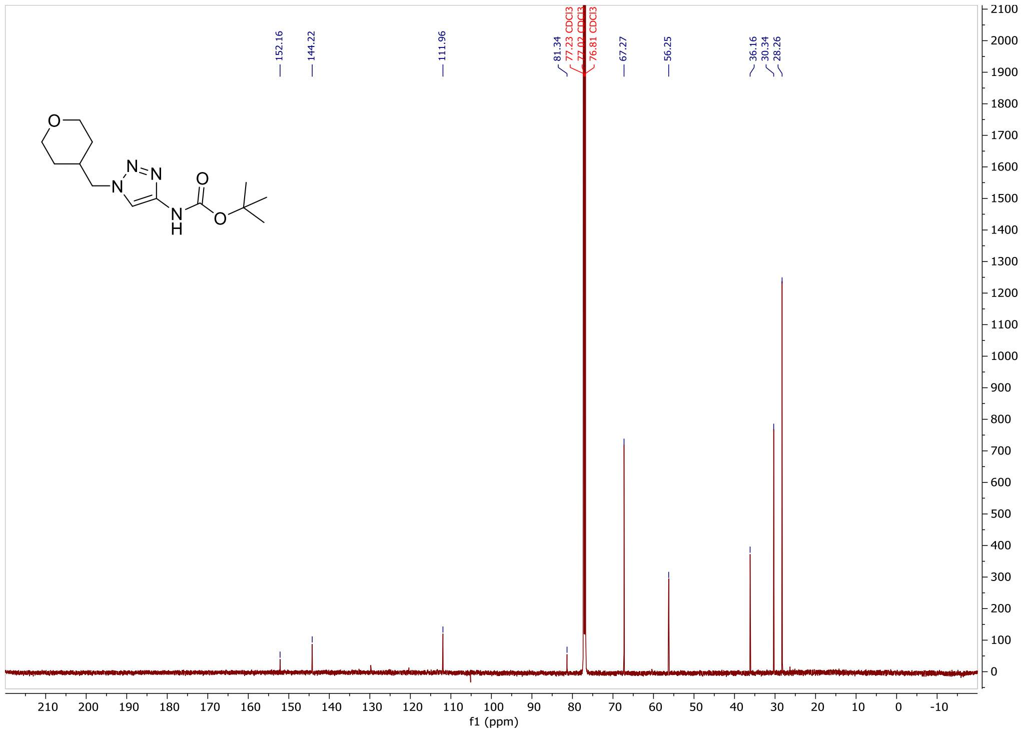

^{1}H NMR (500 MHz, DMSO-d_6) Spectrum of Compound **22B**

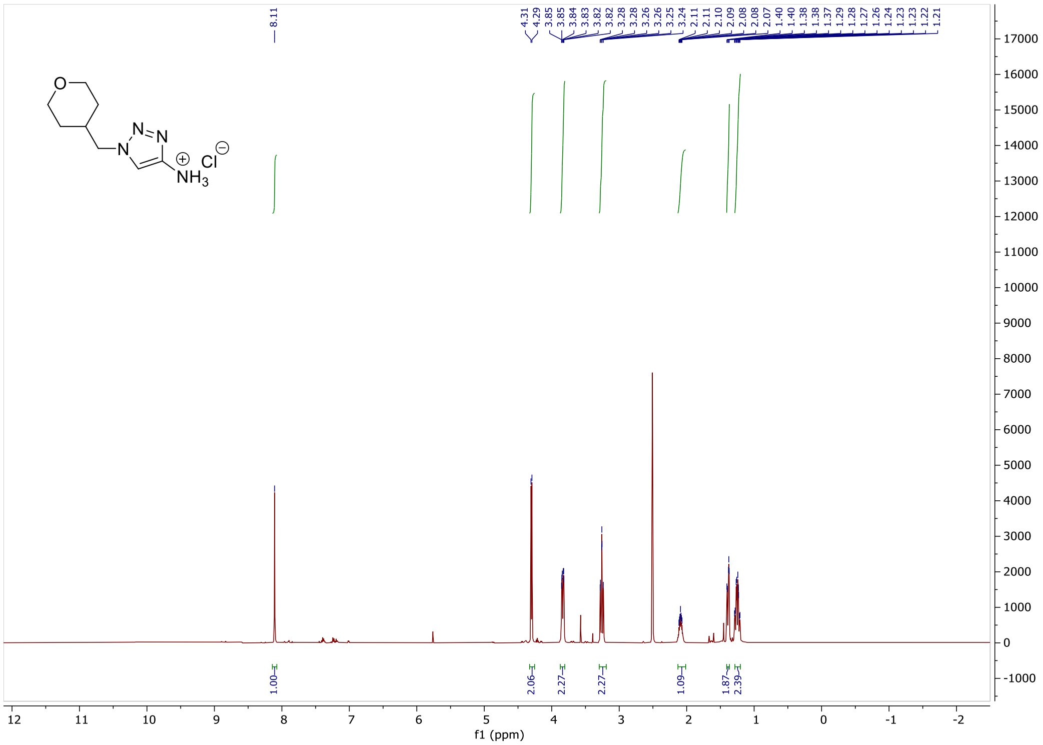

^{13}C NMR (125 MHz, DMSO-d_6) Spectrum of Compound **22B**

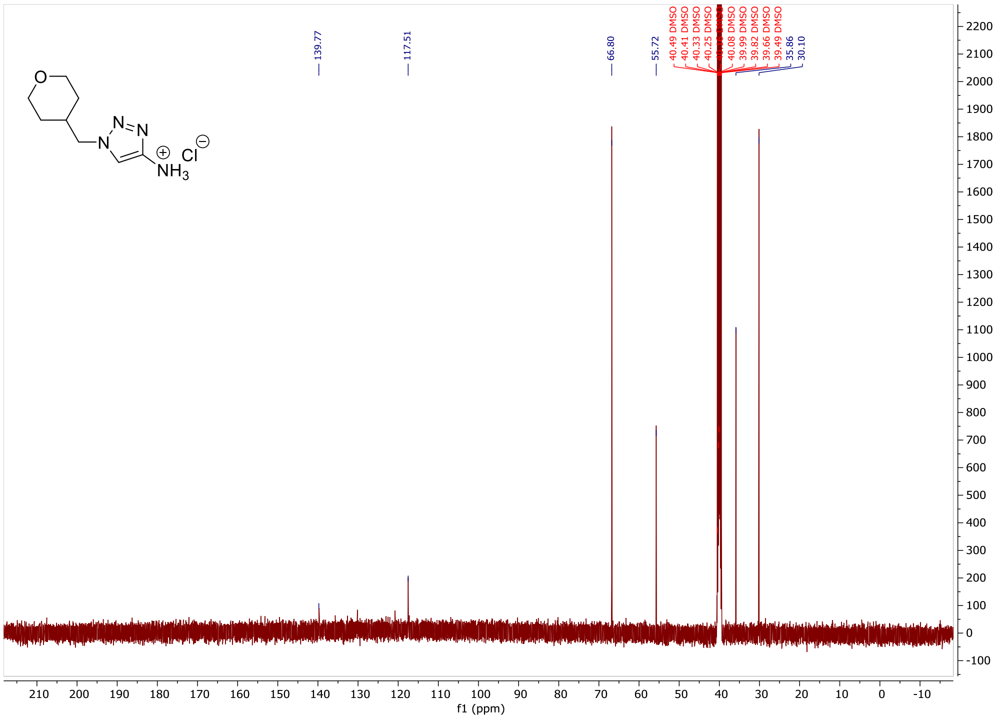

^{1}H NMR (600 MHz, DMSO-d_6) Spectrum of Compound **23B**

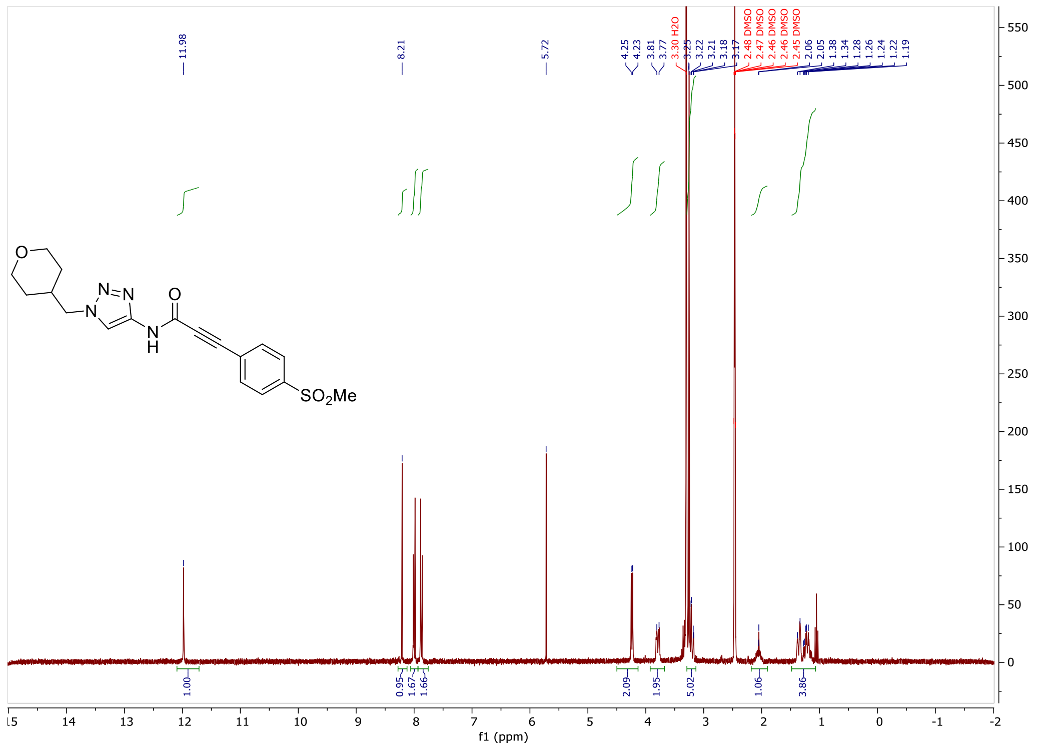

^{13}C NMR (150 MHz, DMSO-d_6) Spectrum of Compound **23B**

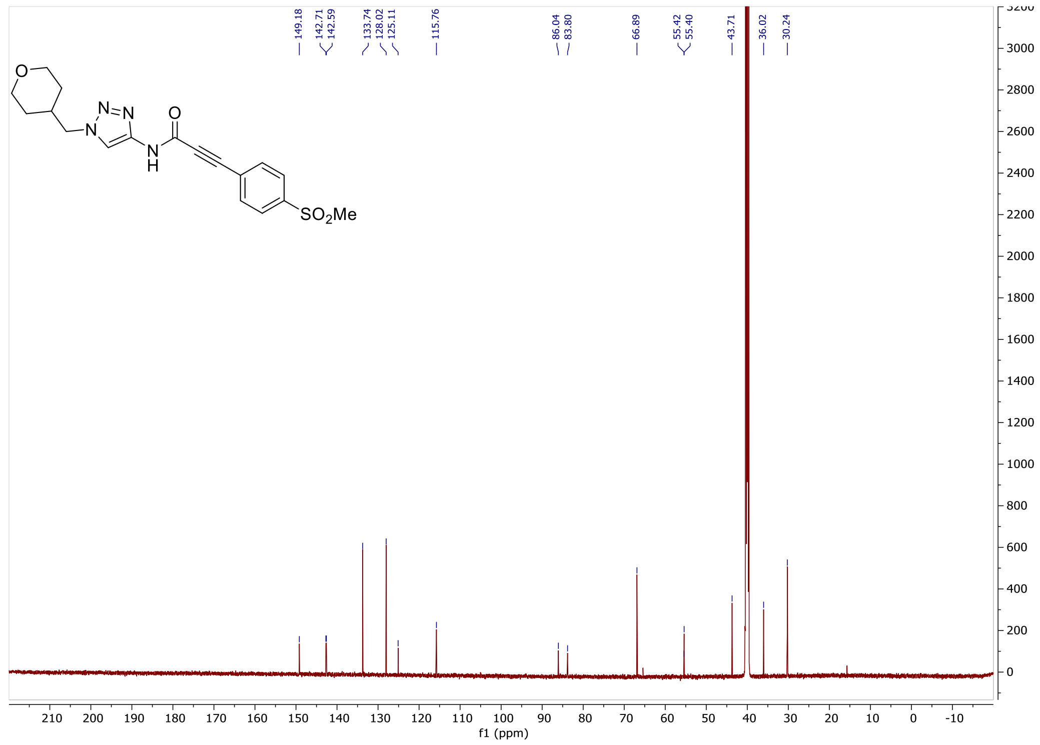

^{1}H NMR (600 MHz, DMSO-d_6) Spectrum of Compound **23C**

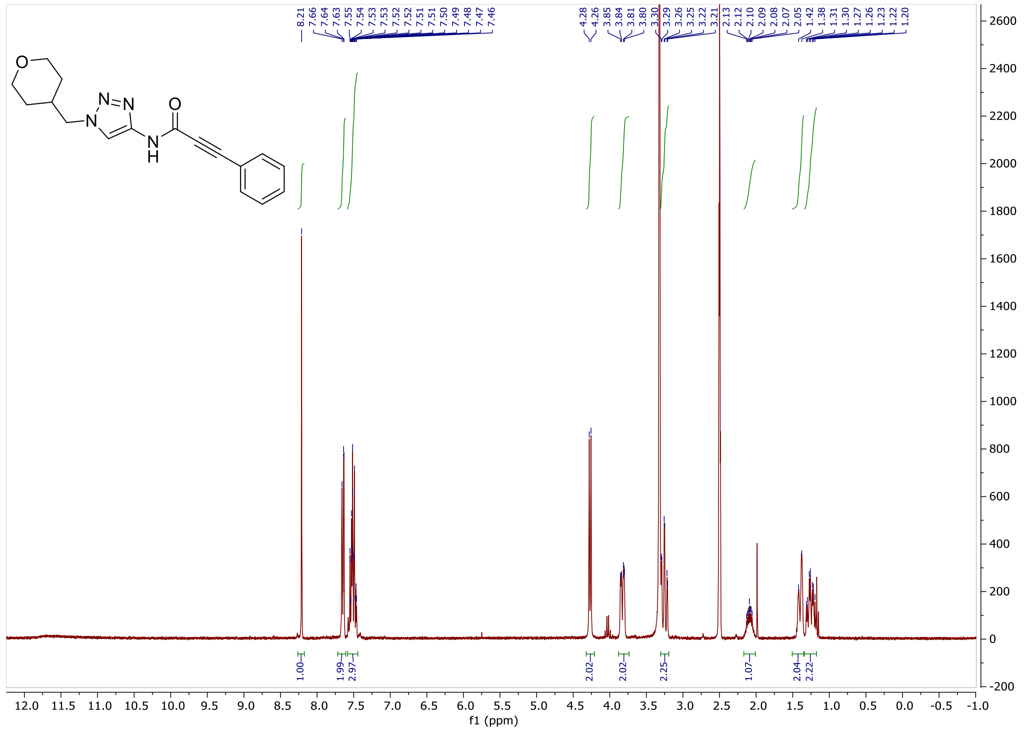

^{13}C NMR (150 MHz, DMSO-d_6) Spectrum of Compound **23C**

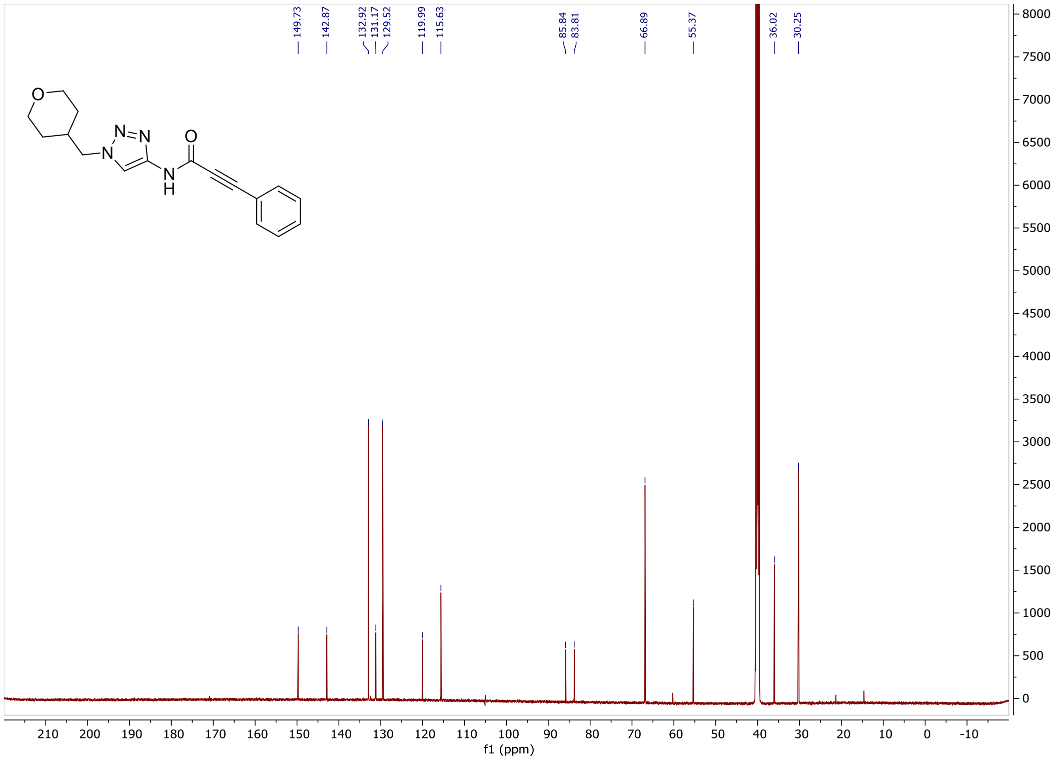

^{1}H NMR (600 MHz, $CDCl_3$) Spectrum of Compound **23D**

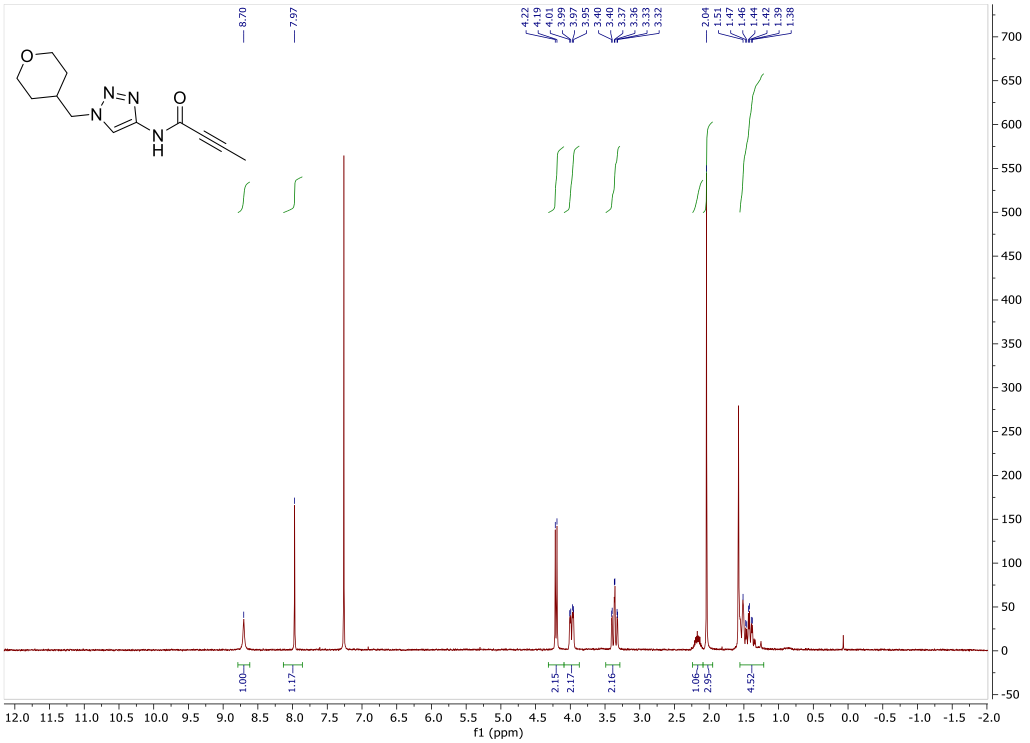

^{13}C NMR (150 MHz, $CDCl_3$) Spectrum of Compound **23D**

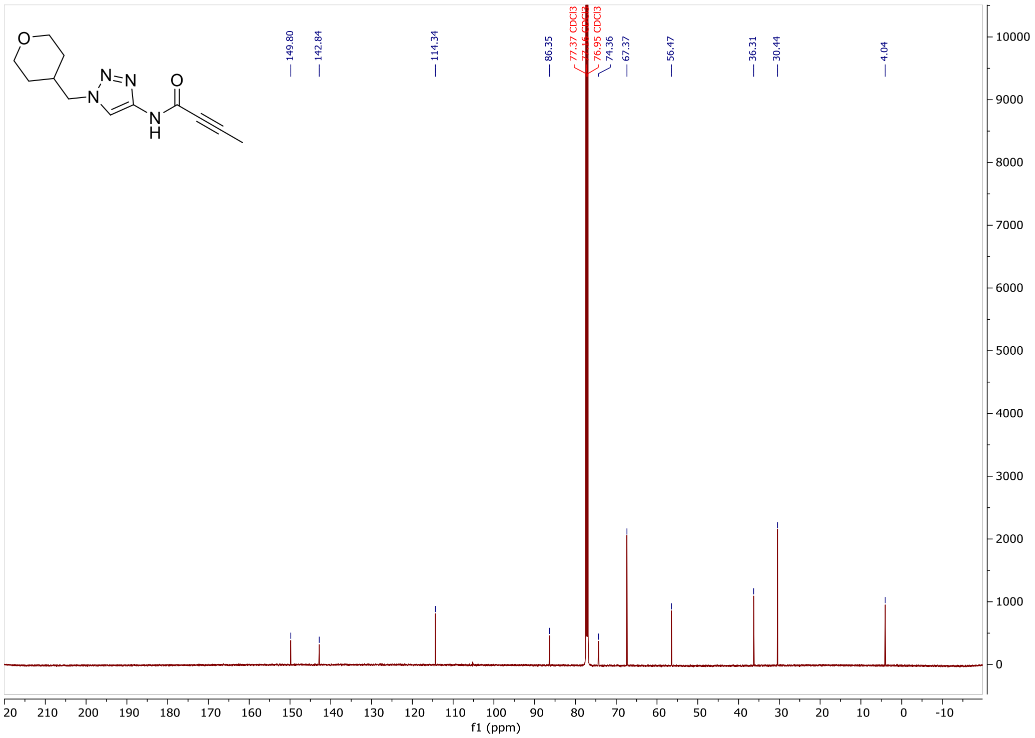

^{1}H NMR (600 MHz, $CDCl_3 + CD_3OD$) Spectrum of Compound **23E**

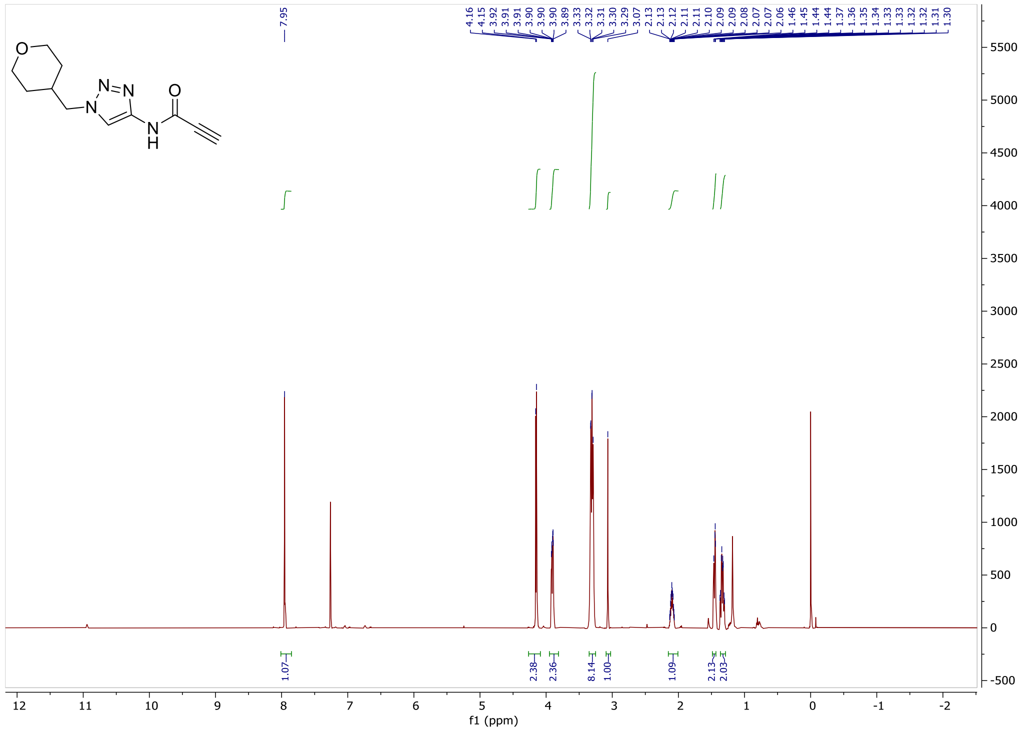

^{13}C NMR (150 MHz, $CDCl_3 + CD_3OD$) Spectrum of Compound **23E**

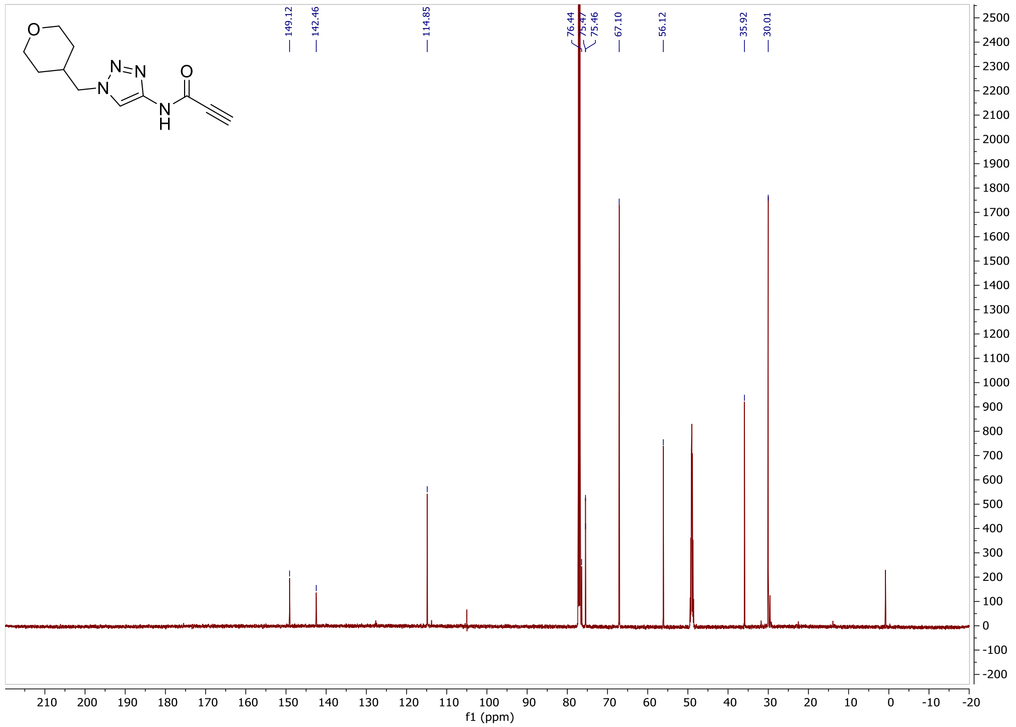

^{1}H NMR (300 MHz, $CDCl_3$) Spectrum of Compound **26**

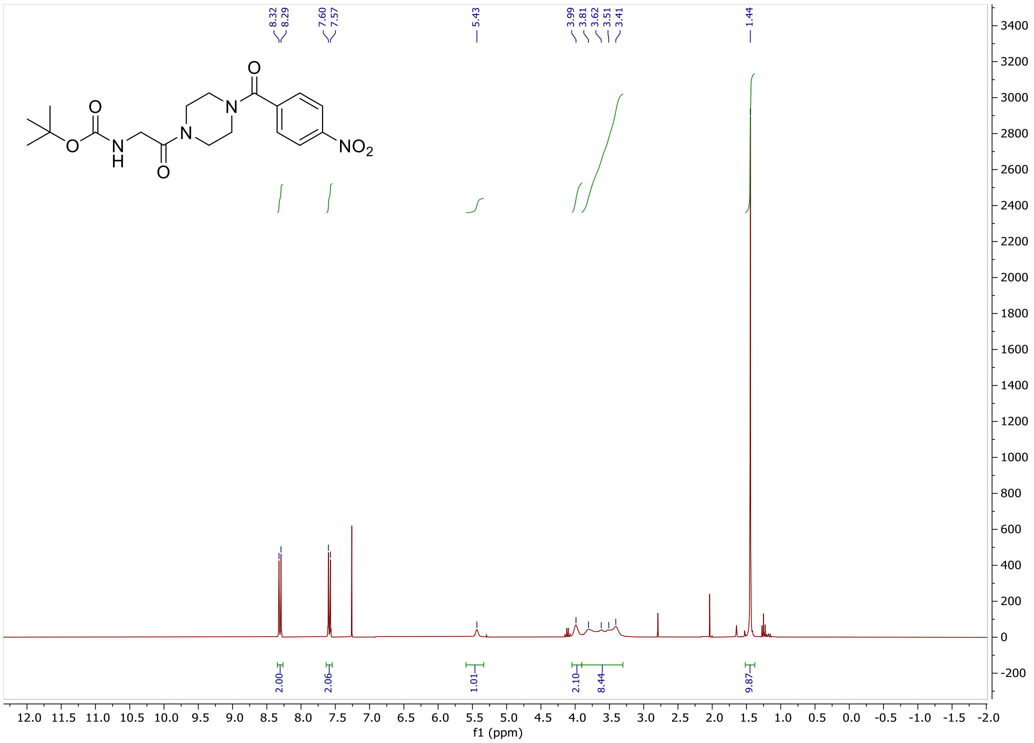

^{13}C NMR (75 MHz, $CDCl_3$) Spectrum of Compound **26**

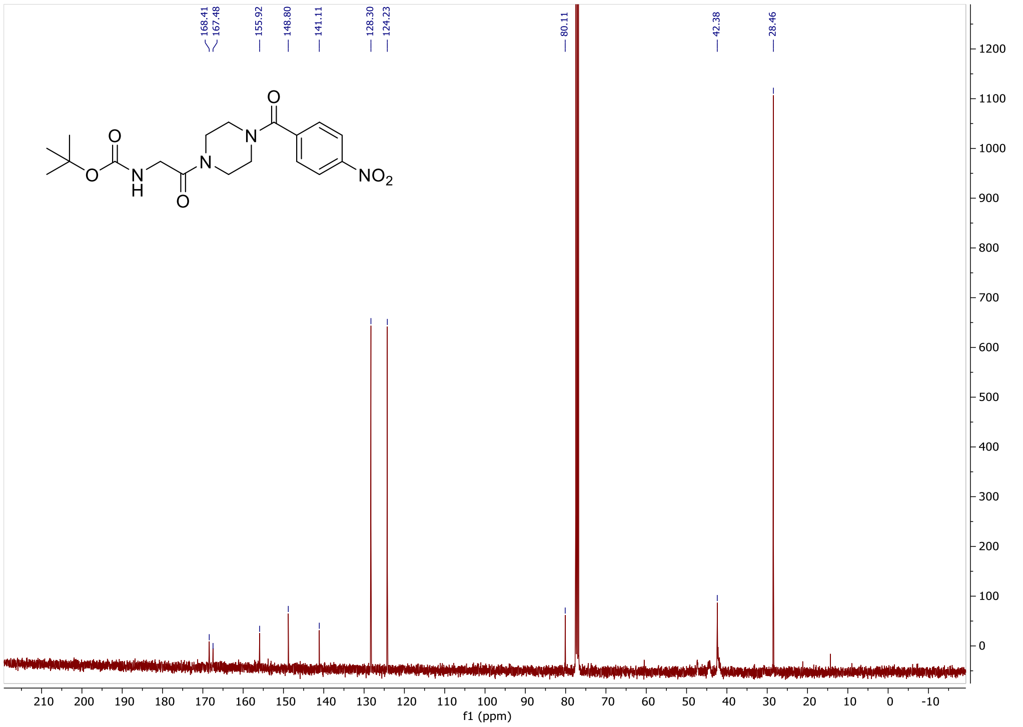

^{1}H NMR (300 MHz, DMSO-d_6) Spectrum of Compound **27**

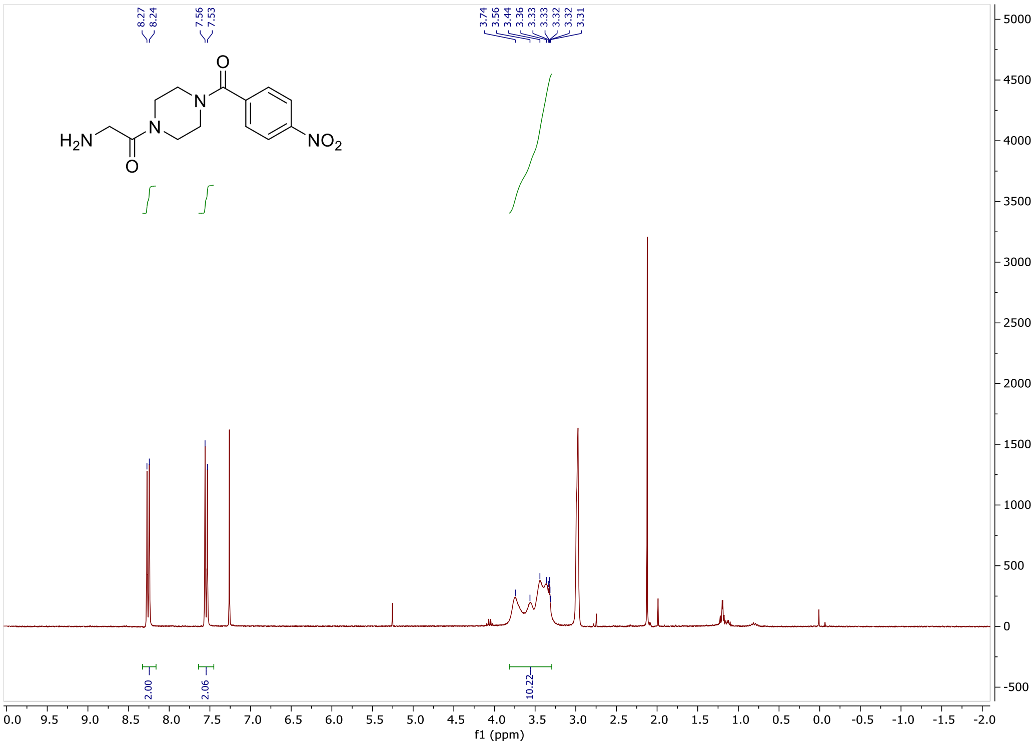

^{13}C NMR (75 MHz, DMSO-d_6) Spectrum of Compound **27**

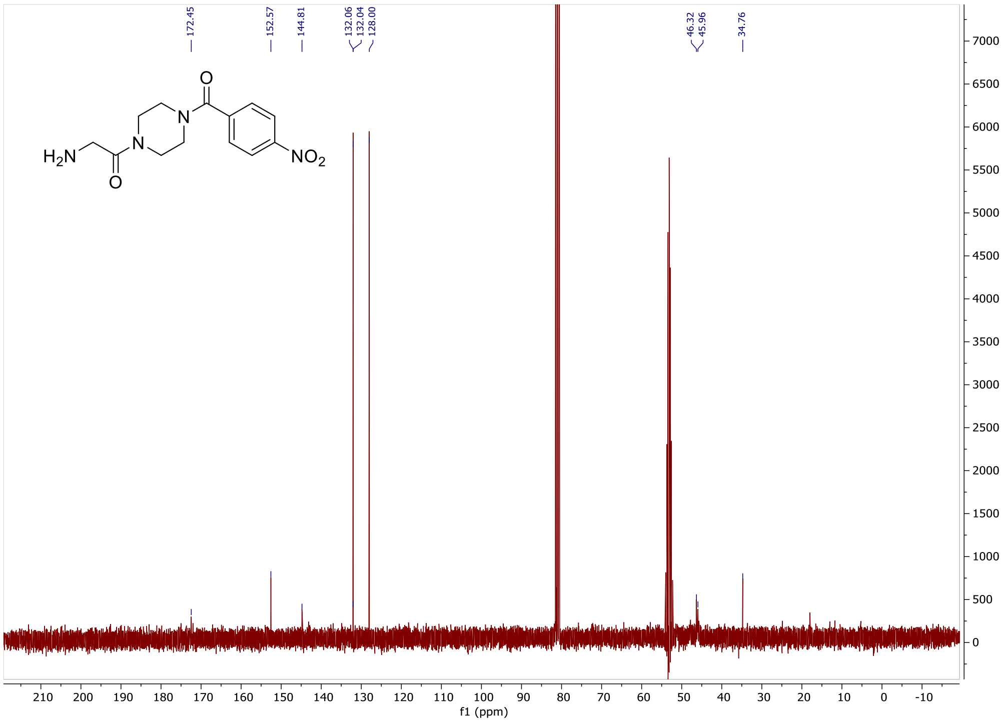

^{1}H NMR (300 MHz, $CDCl_3$) Spectrum of Compound **28**

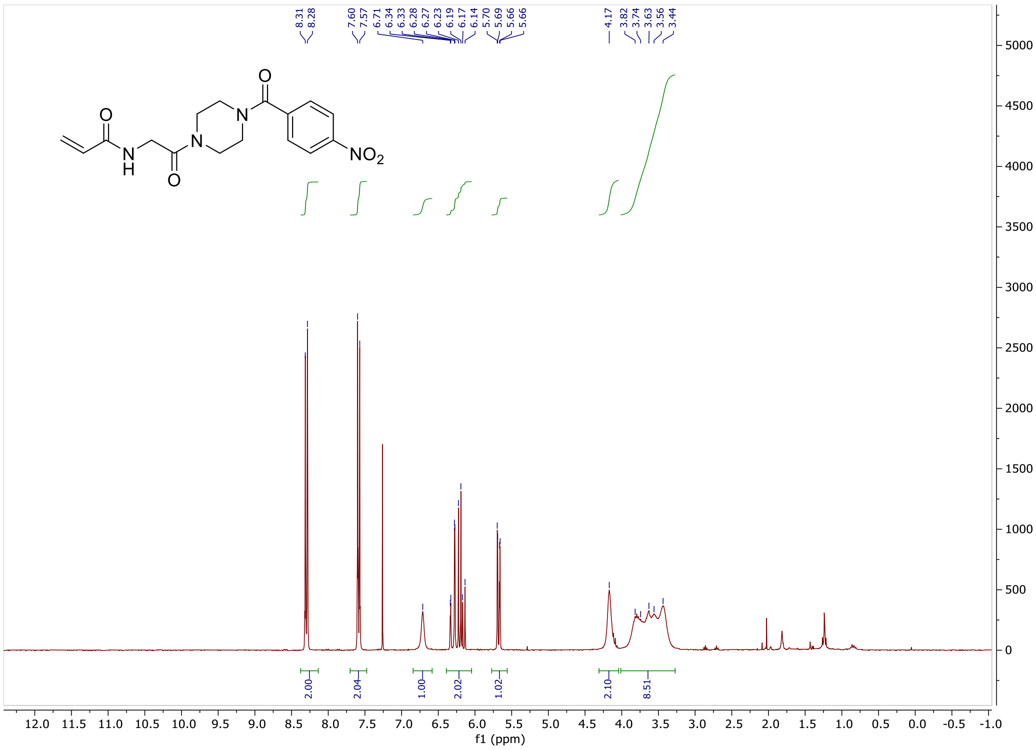

^{13}C NMR (75 MHz, $CDCl_3$) Spectrum of Compound **28**

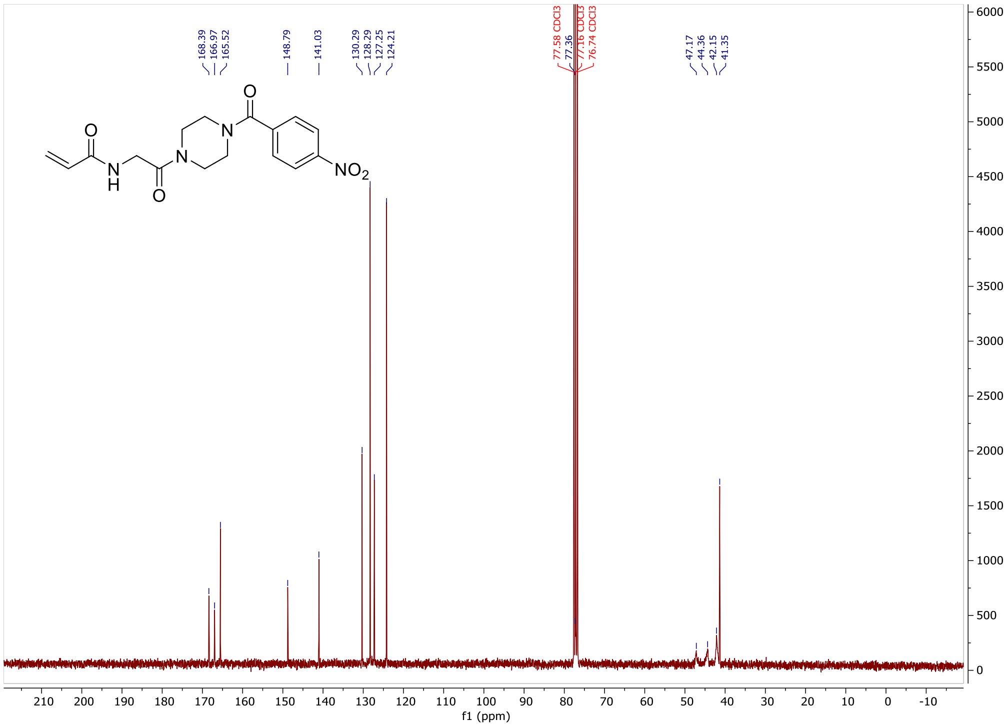

^{1}H NMR (300 MHz, $CDCl_3$) Spectrum of Compound **31**

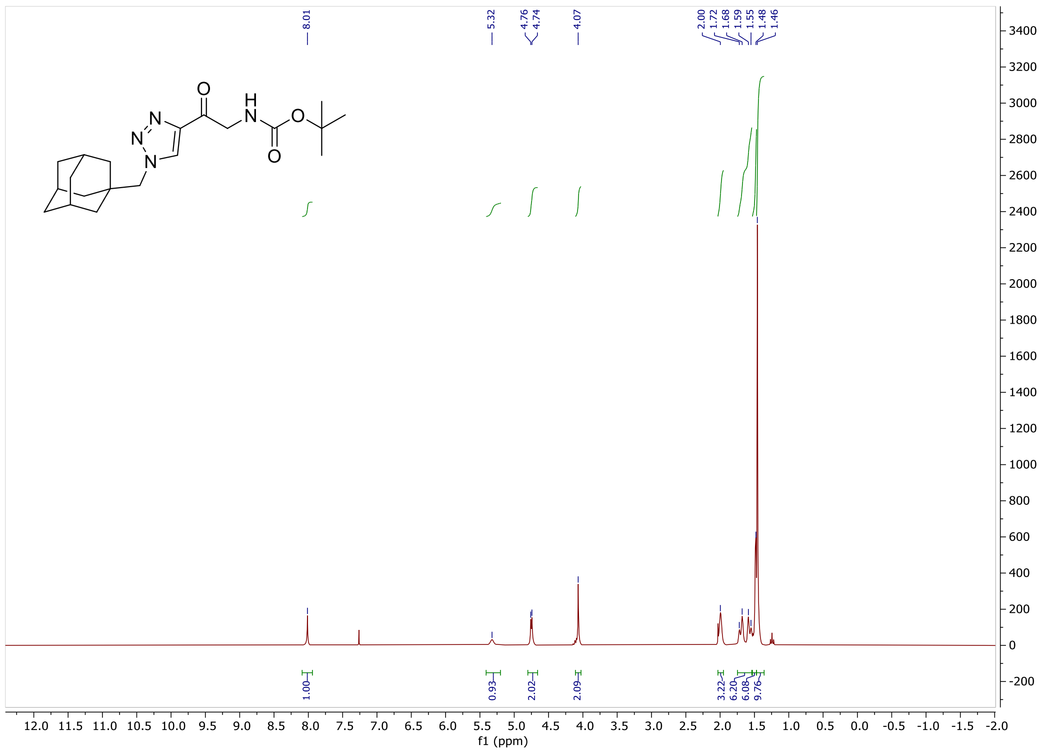

^{13}C NMR (75 MHz, $CDCl_3$) Spectrum of Compound **31**

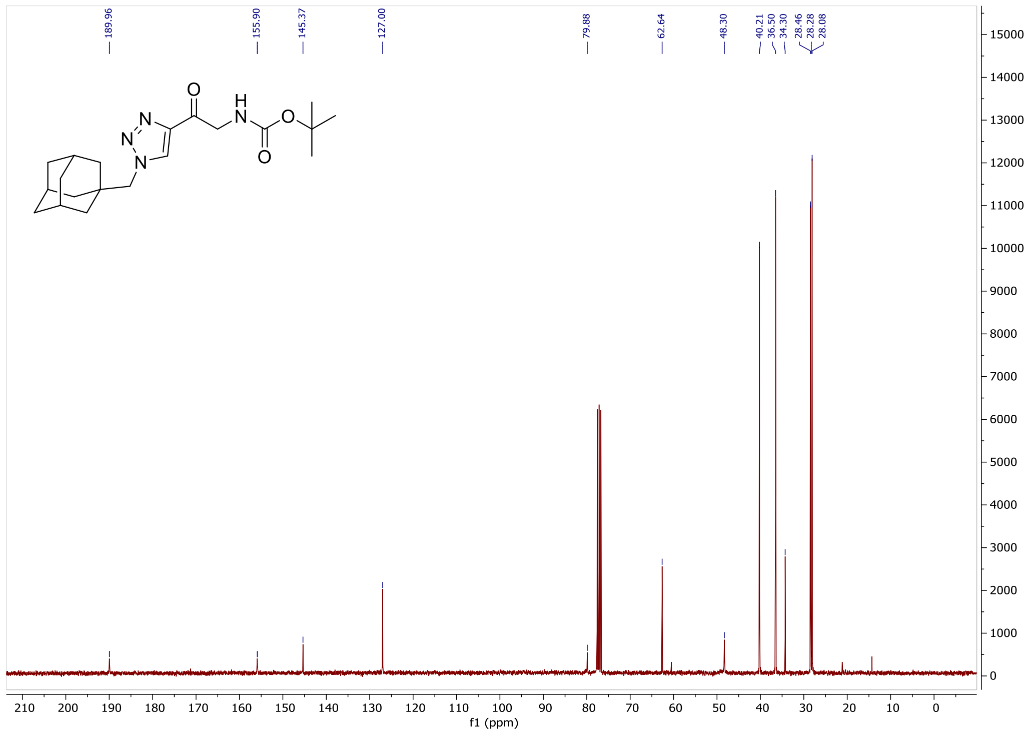

^{1}H NMR (300 MHz, CD_3OD) Spectrum of Compound **32**

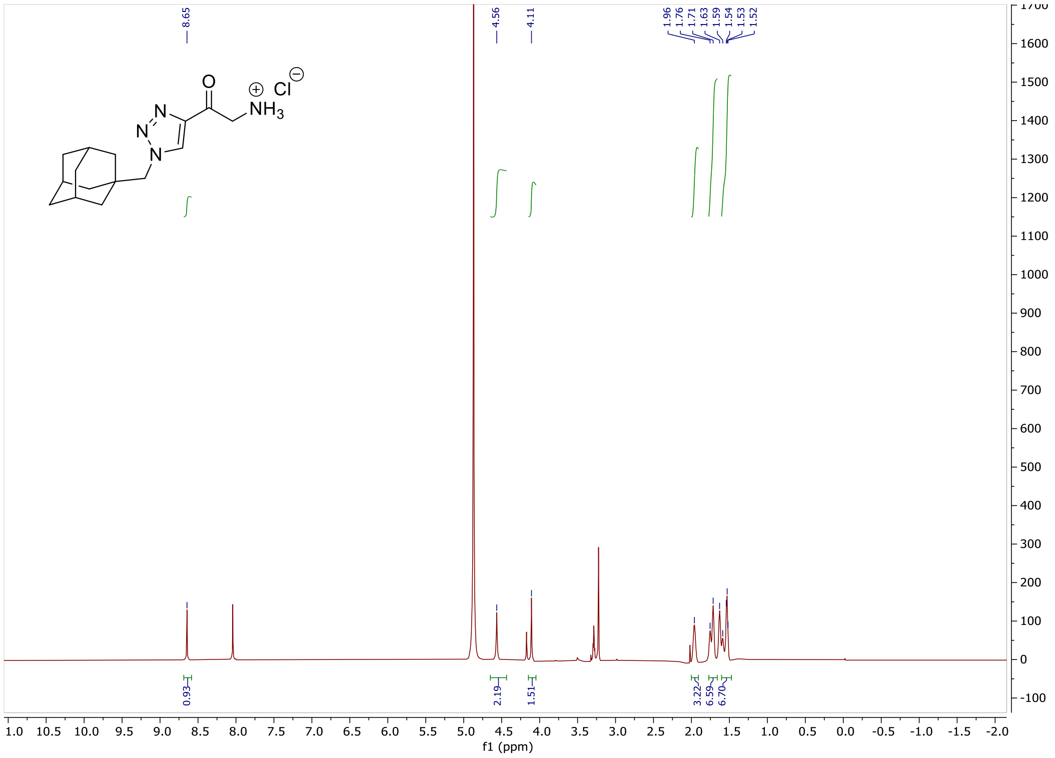

^{13}C NMR (75 MHz, CD_3OD) Spectrum of Compound **32**

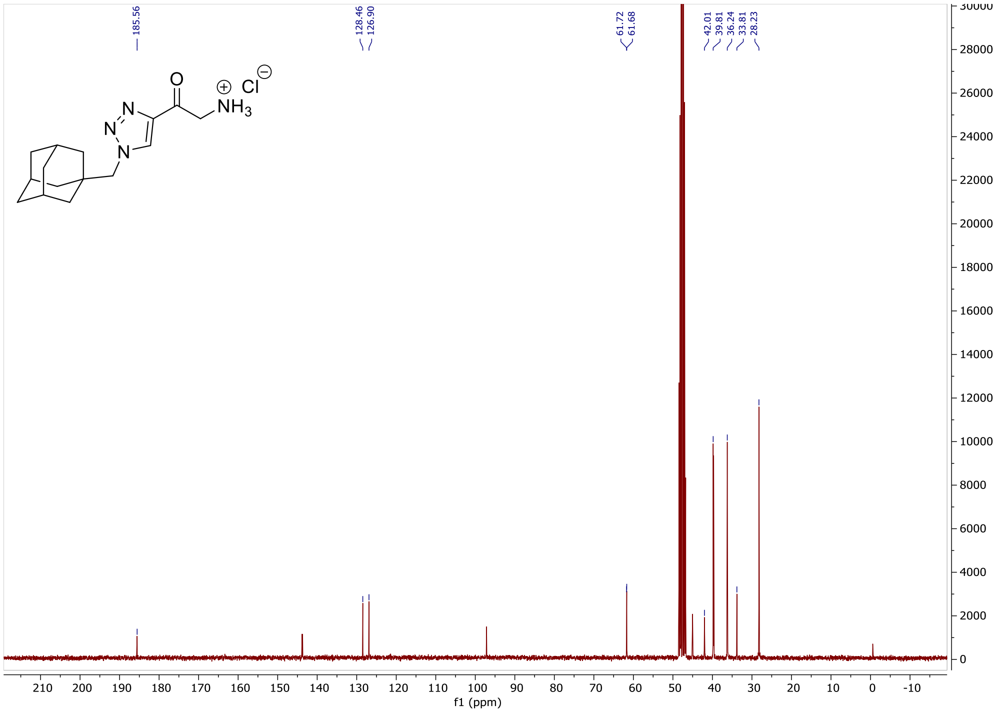

^{1}H NMR (300 MHz, $CDCl_3$) Spectrum of Compound **33**

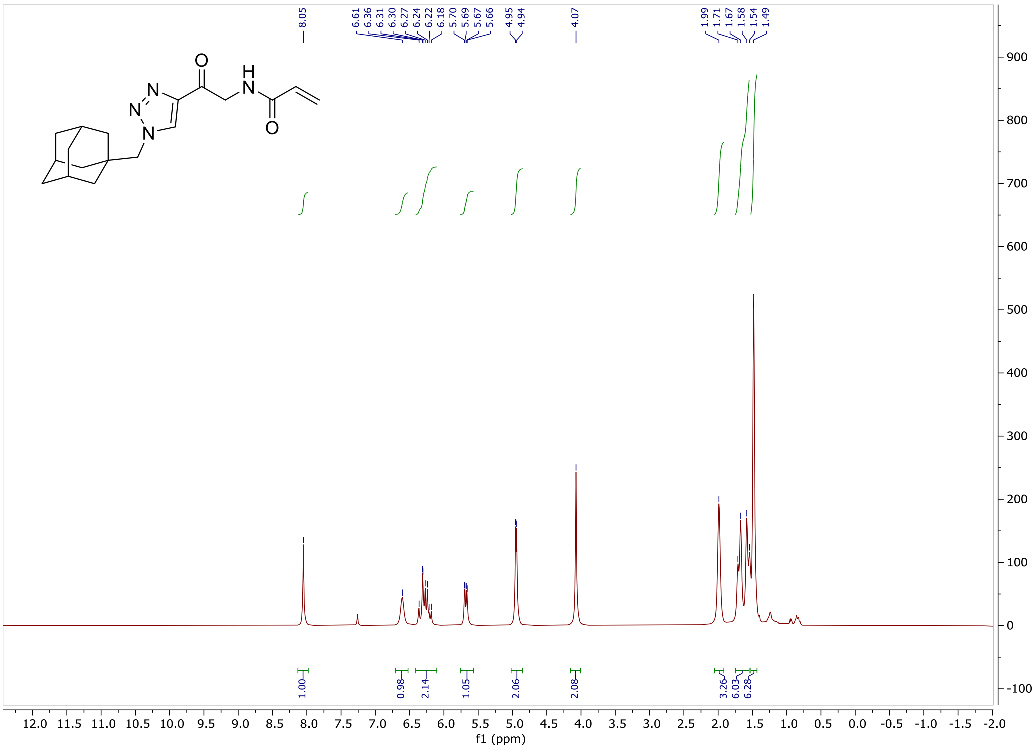

^{13}C NMR (75 MHz, $CDCl_3$) Spectrum of Compound **33**

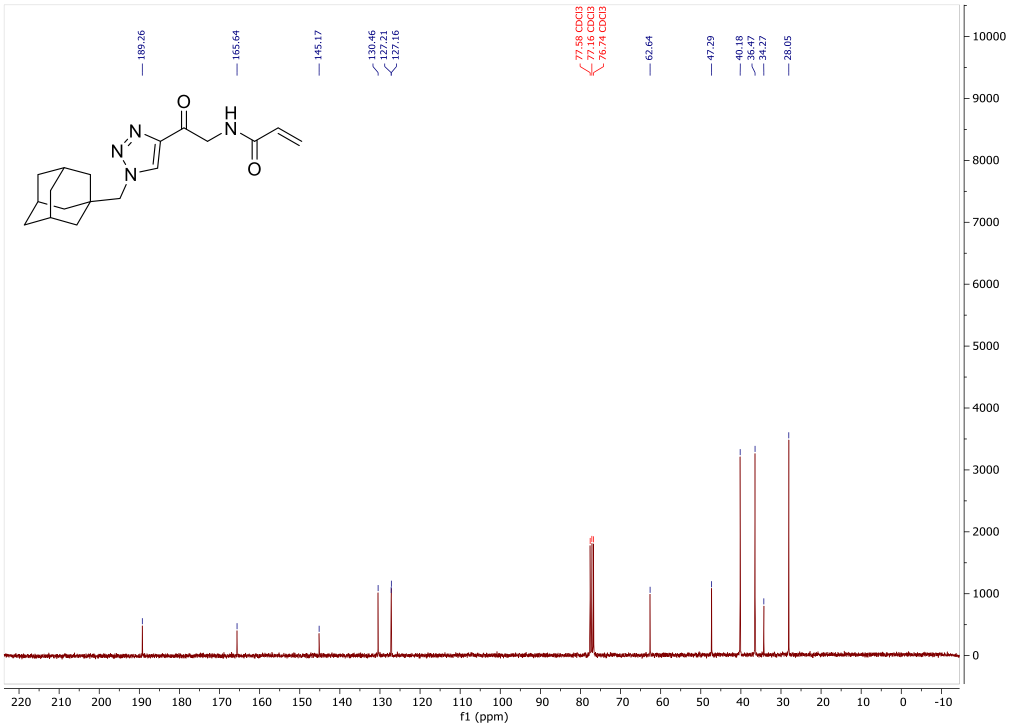

^{1}H NMR (300 MHz, $CDCl_3$) Spectrum of Compound **36**

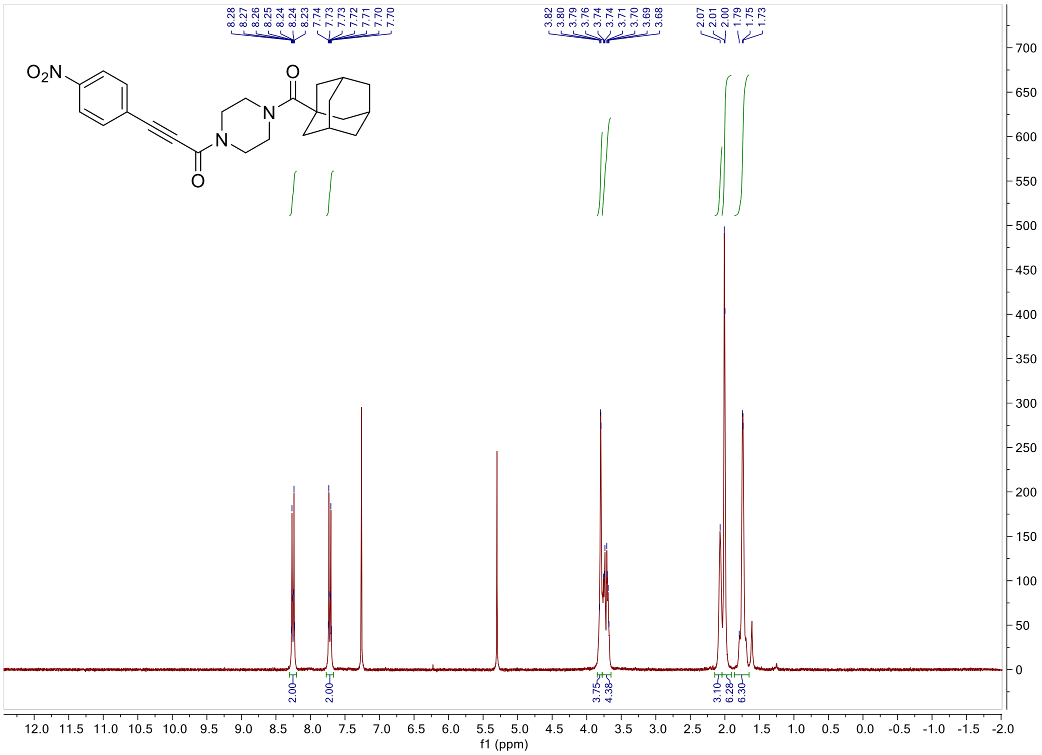

^{13}C NMR (75 MHz, $CDCl_3$) Spectrum of Compound **36**

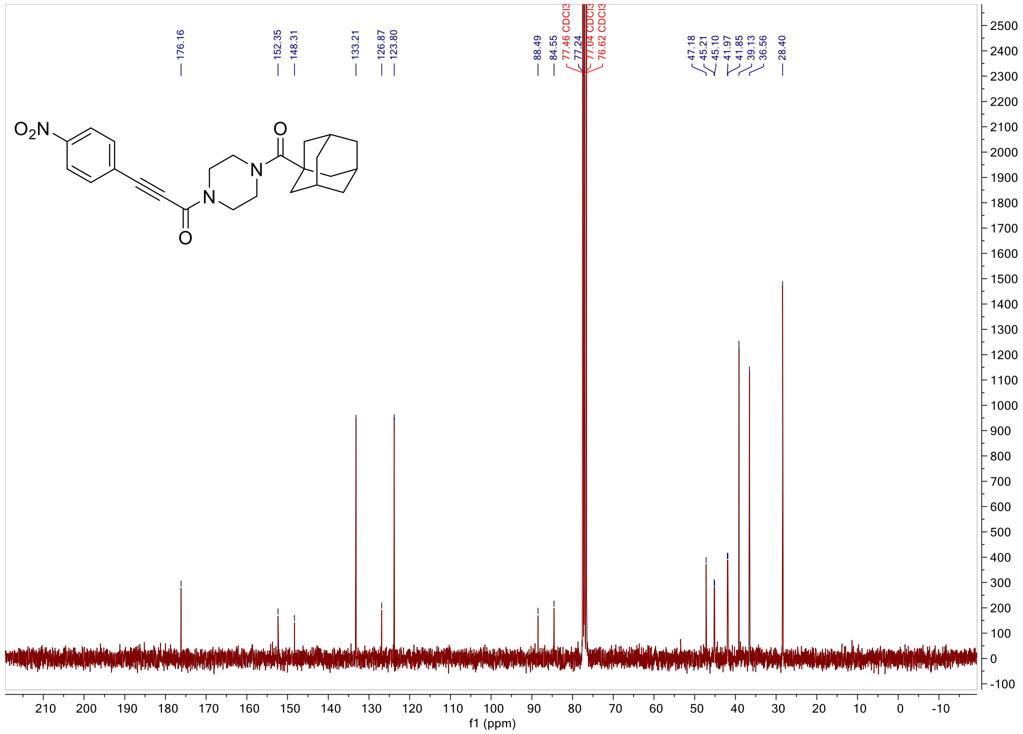

^{1}H NMR (300 MHz, $CDCl_3$) Spectrum of Compound **38**

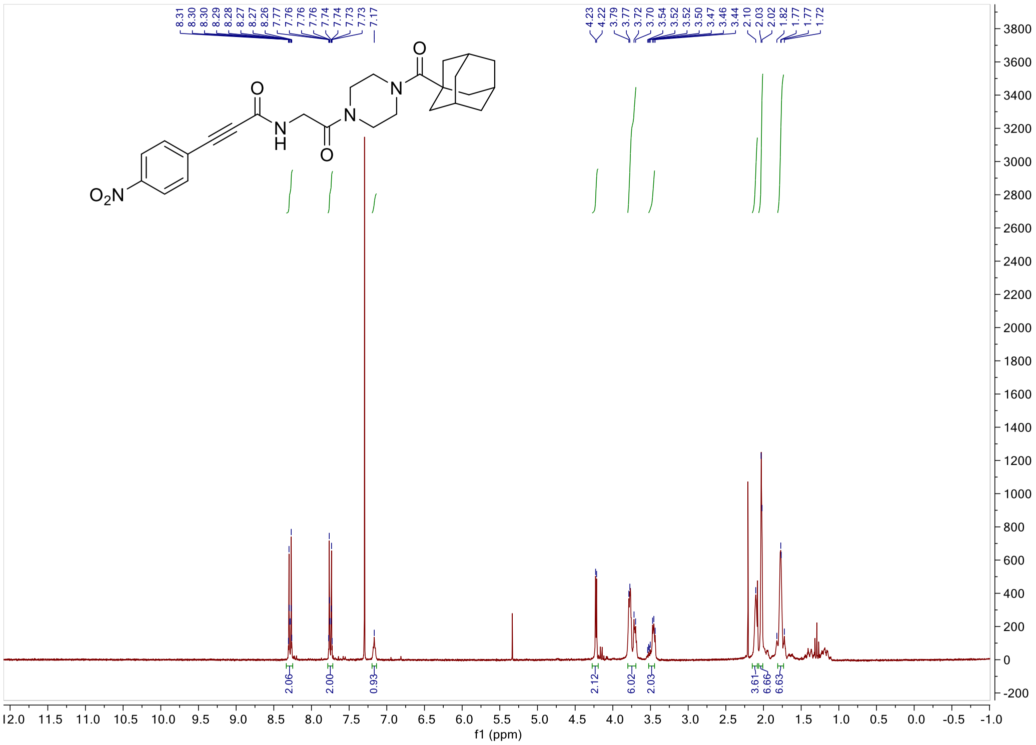

^{13}C NMR (75 MHz, $CDCl_3$) Spectrum of Compound **38**

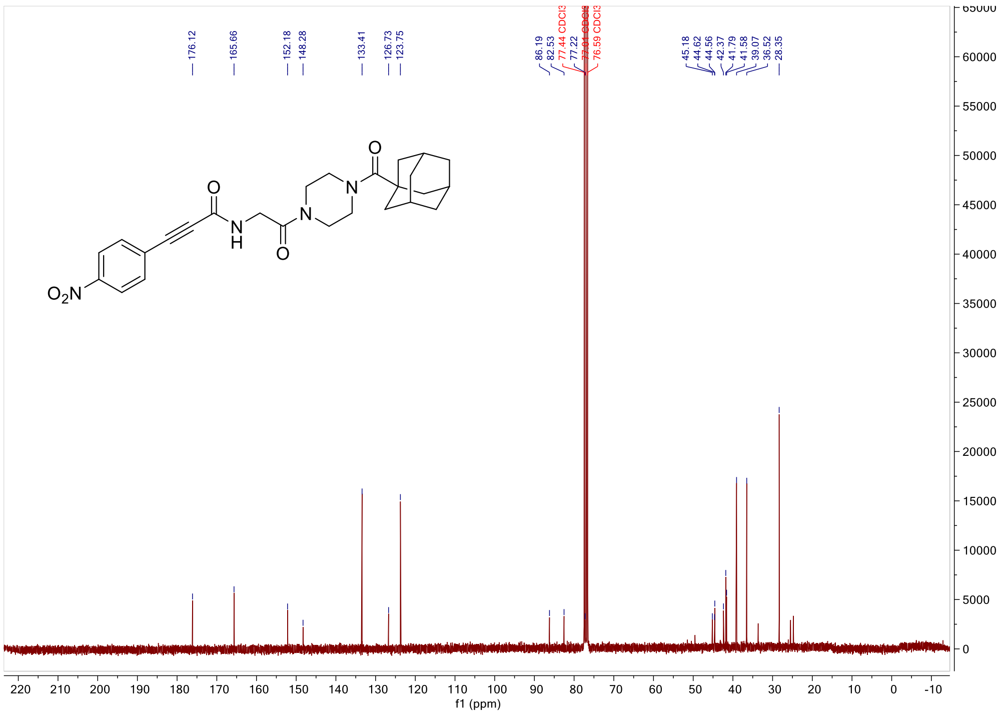

^{1}H NMR (300 MHz, $CDCl_3$) Spectrum of Compound **40**

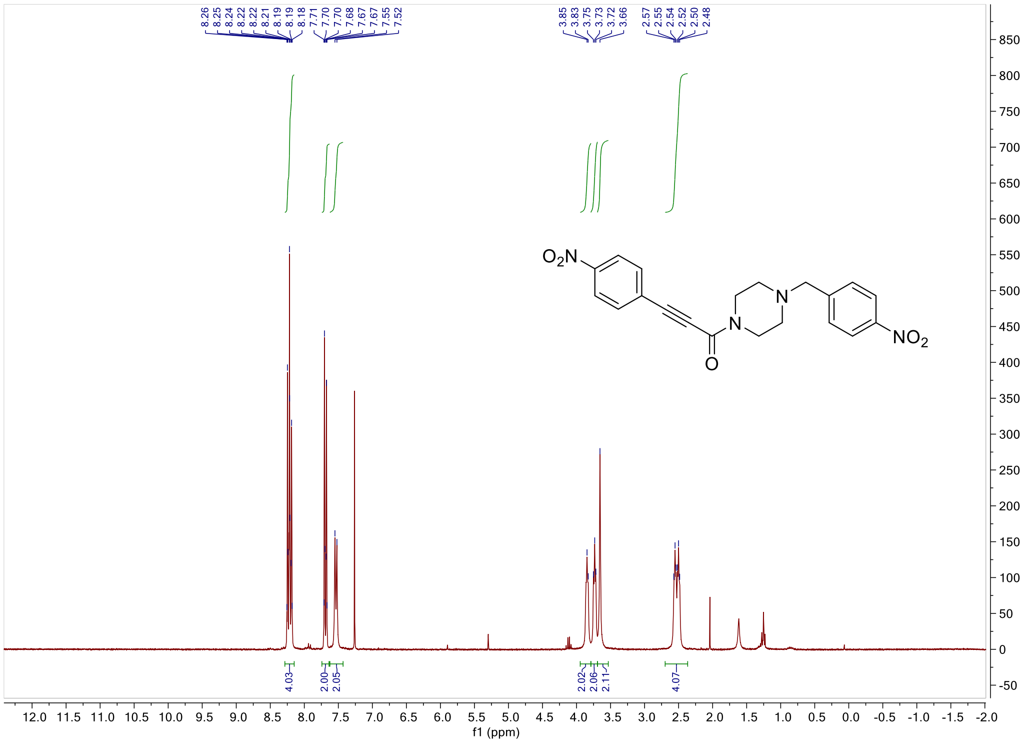

^{13}C NMR (75 MHz, $CDCl_3$) Spectrum of Compound **40**

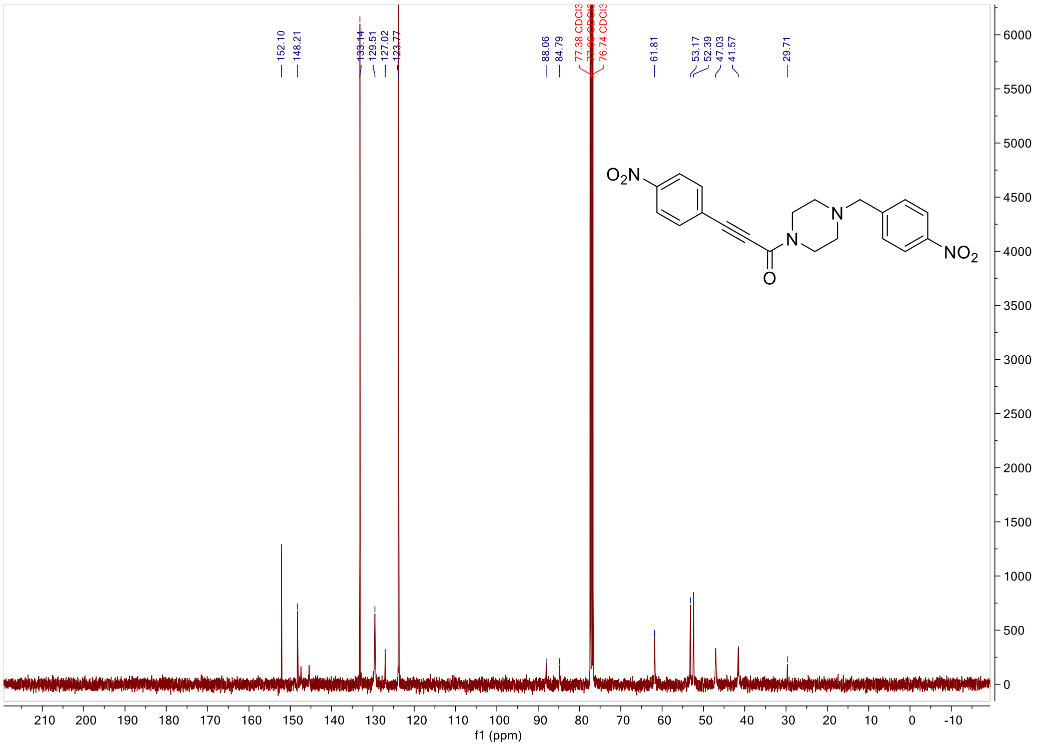

Supplement: MD-016-D5MD00777A-s001 [file MD-016-D5MD00777A-s001.pdf]
